# Total Synthesis of the Proposed Structure of (−)-Novofumigatamide, Isomers Thereof, and Analogues. Part I

**DOI:** 10.1021/acs.joc.2c01227

**Published:** 2022-09-22

**Authors:** Patricia García-Domínguez, Paula Lorenzo, Rosana Álvarez, Angel R. de Lera

**Affiliations:** CINBIO, Universidade de Vigo, 36310 Vigo, Spain

## Abstract

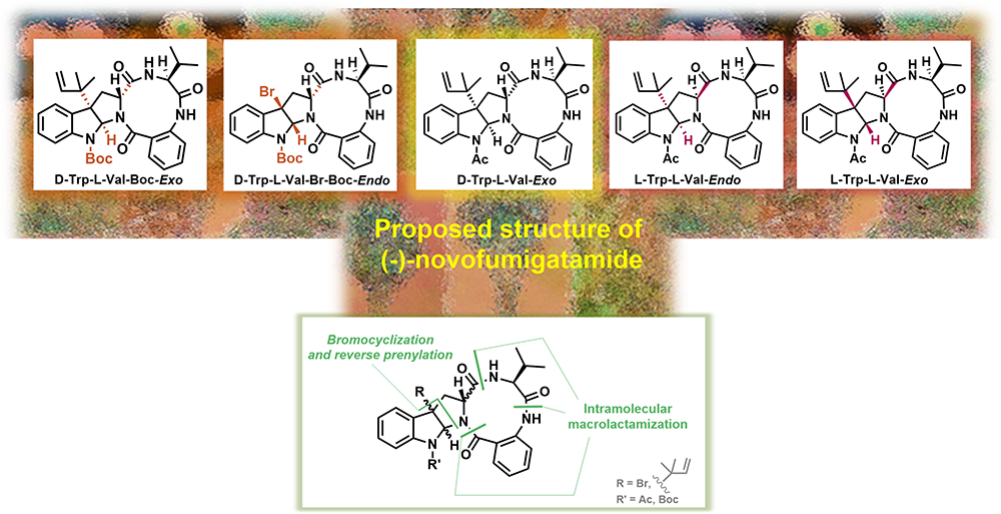

The total synthesis of the suggested structure of (−)-novofumigatamide, a natural product containing a C3-reverse prenylated *N*-acetyl-*exo*-hexahydropyrrolo[2,3-*b*]indole motif fused to a 10-membered ring lactam, was achieved using the macrolactam formation in advance of a diastereoselective bromocyclization and reverse prenylation steps. Since the NMR data of the synthetic sample did not match those of the natural product, the *endo*-bromo precursor of a *N*-Boc analogue and additional diastereomers derived from l-Trp were also synthesized. Five alternative synthetic routes, which differed in the order of final key steps used for the construction of the 10-membered ring lactam and the hexahydropyrrolo[2,3-*b*]indole framework within the polycyclic skeleton and also in the amide bond selected for the ring-closing of the macrolactam, were thoroughly explored. Much to our dismay, the lack of spectroscopic correlations between the proposed structure of natural (−)-novofumigatamide and the synthetic products suggested a different connectivity between the atoms. Additional synthetic efforts to assemble alternative structures of the natural product and isomers thereof (see accompanying paper; DOI: 10.1021/acs.joc.2c01228) further highlighted the frustrating endeavors toward the identification of a natural product.

## Introduction

The hexahydropyrrolo[2,3-*b*]indole skeleton bearing a reverse-prenyl group at the C3α position is a structural motif present in a large number of tryptophan-derived alkaloids, mainly in those isolated from fungi and other microorganisms.^1,2^ These naturally occurring compounds display a broad structural diversity and a wide array of biological activities, which make them particularly appealing from a synthetic point of view.

**Figure 1:**
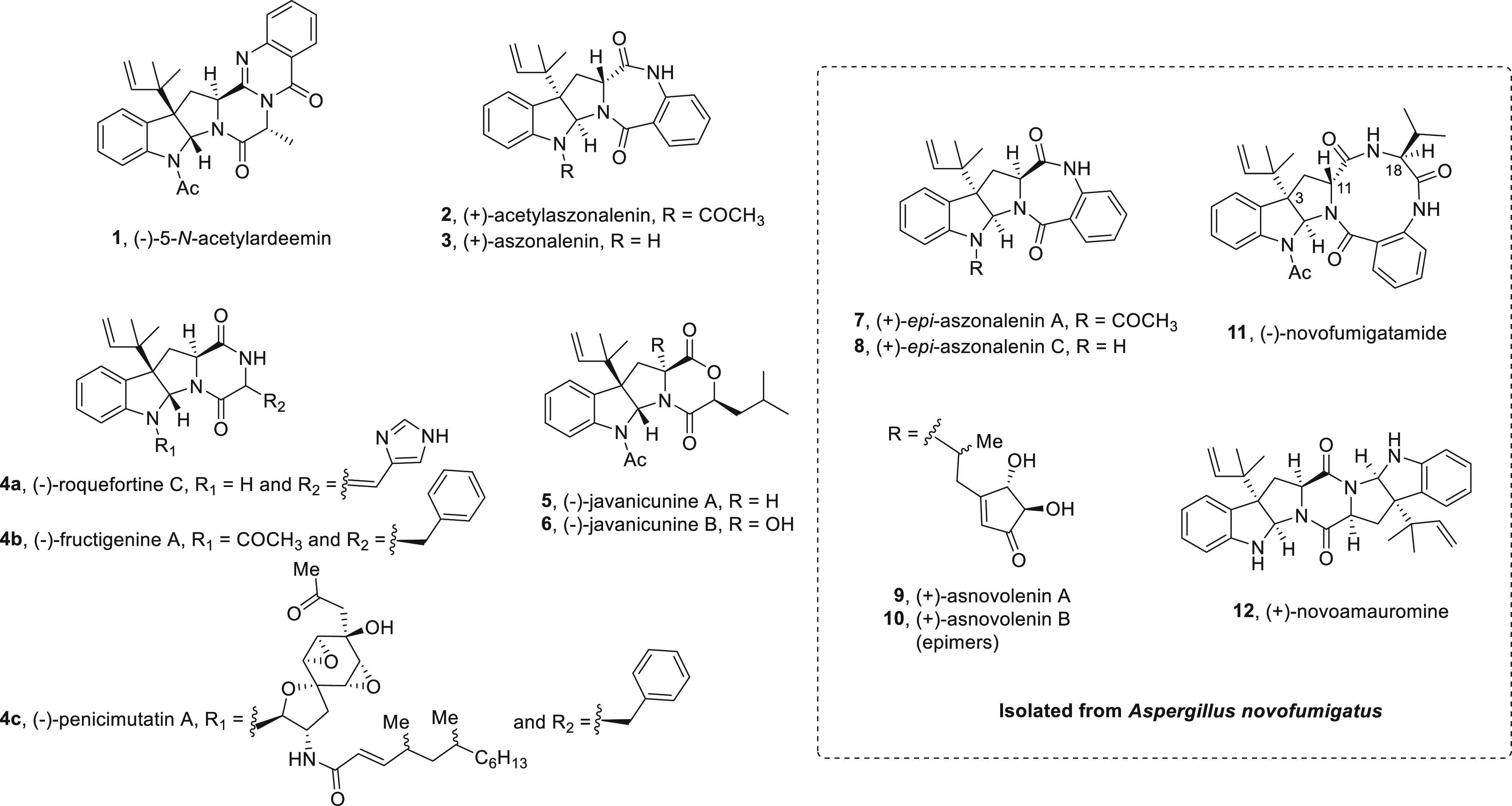
Representative reverse-prenylated pyrroloindoline alkaloids. On the dashed box, those isolated from *Aspergillus novofumigatus.*

Outstanding examples of this sort of secondary metabolites contain the pyrroloindoline unit fused to a diketopiperazine, such as (−)-5-*N*-acetylardeemin (**1**), (−)-roquefortine C (**4a**), (−)-fructigenine A (**4b**), (−)-penicimutatin A (**4c**), and (+)-novoamauromine (**12**) (Figure 1),^3−16^ or to a diketomorpholine ring, as in (−)-javacunine A (**5**) and (−)-javacunine B (**6**) (Figure 1).^17,18^ Less common molecular skeletons possess the pyrroloindoline moiety connected to a benzodiazepinedione framework, as in the family of aszonalenin alkaloids (**2** and **3**, Figure 1),^19−21^ the related congeners *epi*-aszonalenins (**7** and **8**, Figure 1),^22^ and the most recently reported asnovolenines (**9** and **10**, Figure 1).^23^

From the methanolic extract of the CBS117520 strain of the fungus *Aspergillus novofumigatus* cultivated on rice, Hosoe and co-workers isolated in 2010 a new cyclotripeptide, termed (−)-novofumigatamide (**11**), together with other previously identified natural products.^24^ NMR spectroscopic data revealed the presence of an *exo*-hexahydropyrrolo[2,3-*b*]indole motif fused to a 10-membered ring lactam, an unprecedented structural feature in these secondary metabolites, which in turn should be generated by the condensation of a valine and an anthranilic acid fragments. In addition, this new reverse prenylated alkaloid contains an acetyl group at the indole nitrogen. The combination of NMR spectroscopy and Marfey’s degradative analysis^25^ allowed us to puzzle out the relative and absolute configurations of (−)-novofumigatamide (**11**), which was determined to have its stereochemical origin on d-tryptophan and l-valine amino acids.

Despite the fact that (−)-novofumigatamide (**11**) did not show antifungal or antiproliferative activities against some specific fungal strains or cancer cell lines, respectively, its structural resemblance to known compounds with relevant biological activities^10,11,15,22,23,26−28^ and the long-standing interest and experience of our group in this family of alkaloids^29−32^ encouraged us to address the total synthesis of this natural product. The final aim of our synthetic research project was to corroborate the structure of (−)-novofumigatamide (**11**) and obtain enough of this and related compounds for further biological studies. Herein, we present the successful total synthesis of the proposed structure of (−)-novofumigatamide (**11**) and several diastereomers and analogues of this putative natural product structure.

## Results and Discussion

**Scheme 1:**
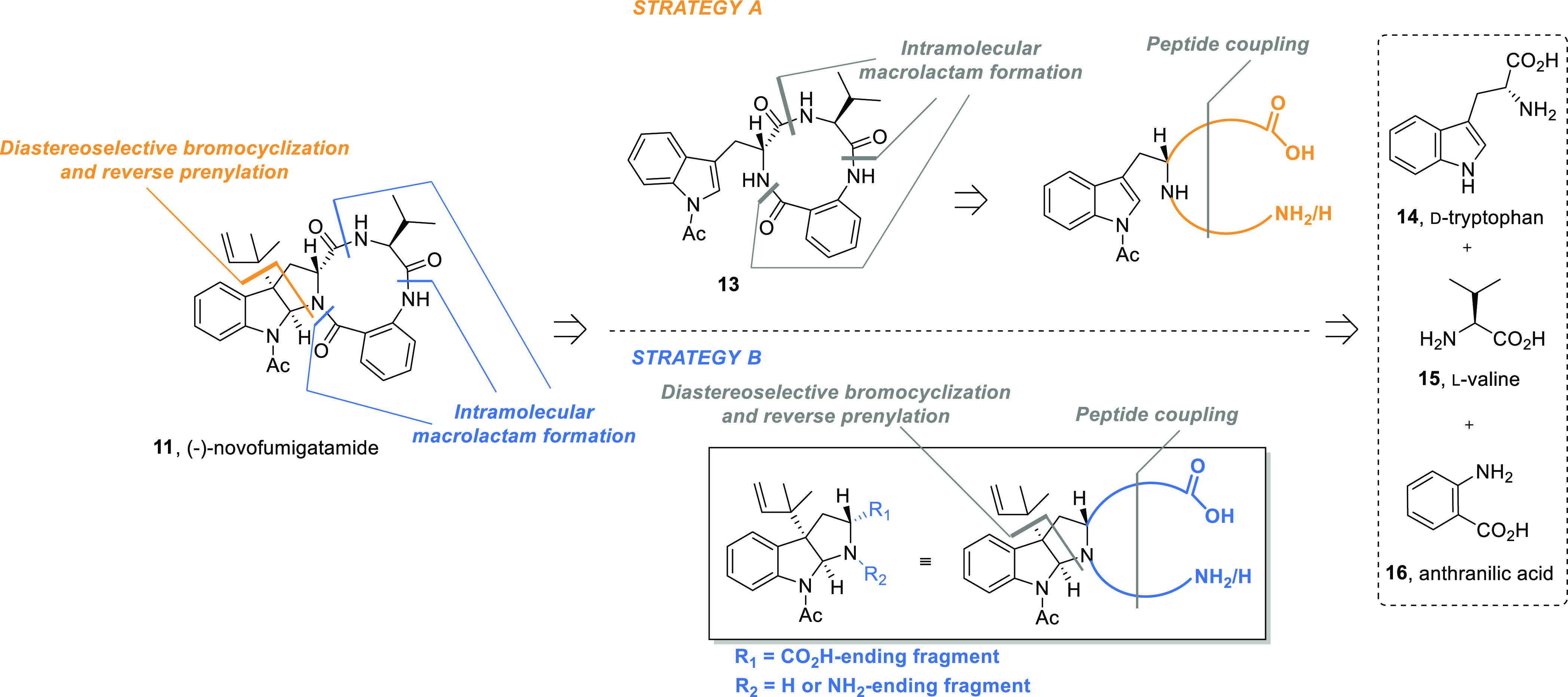
General Synthetic Strategies toward (−)-Novofumigatamide (**11**)

At the outset, two general synthetic strategies toward this naturally occurring compound were envisioned. In a first group of approaches, consecutive diastereoselective bromocyclization and alkylation (reverse prenylation) reactions were proposed as the final key transformations for the construction of (−)-novofumigatamide (**11**) (Scheme 1, strategy A). In a second series of alternative approaches, a challenging intramolecular macrolactam formation, which would be achieved through the formation of different amide bonds within the 10-membered ring macrolactam, was postponed to the last step of the synthesis (Scheme 1, strategy B). The intermediates obtained along these two general disconnections would be prepared in different ways according to the key reactions selected for the assembly of the whole molecular skeleton. In that manner, intermediates arising from type A strategies would be accessible by means of an intramolecular macrolactam formation, whereas the immediate precursors of type B strategies would allow further diversification since either an amide-bond formation or a bromocyclization–alkylation dual sequence could be selected to build these frameworks. Thus, the synthetic routes that we planned to explore were named according to the order of the last key steps of the synthesis. Eventually, unprotected d-tryptophan (**14**), l-valine (**15**), and anthranilic acid (**16**) or different protected derivatives thereof were selected as starting materials for all the routes explored in this work.

### Type A Strategies toward the Proposed Structure of (−)-Novofumigatamide (**11**) and *N*-Boc Analogue *Exo*-**39**

#### Route A.1

**Scheme 2:**
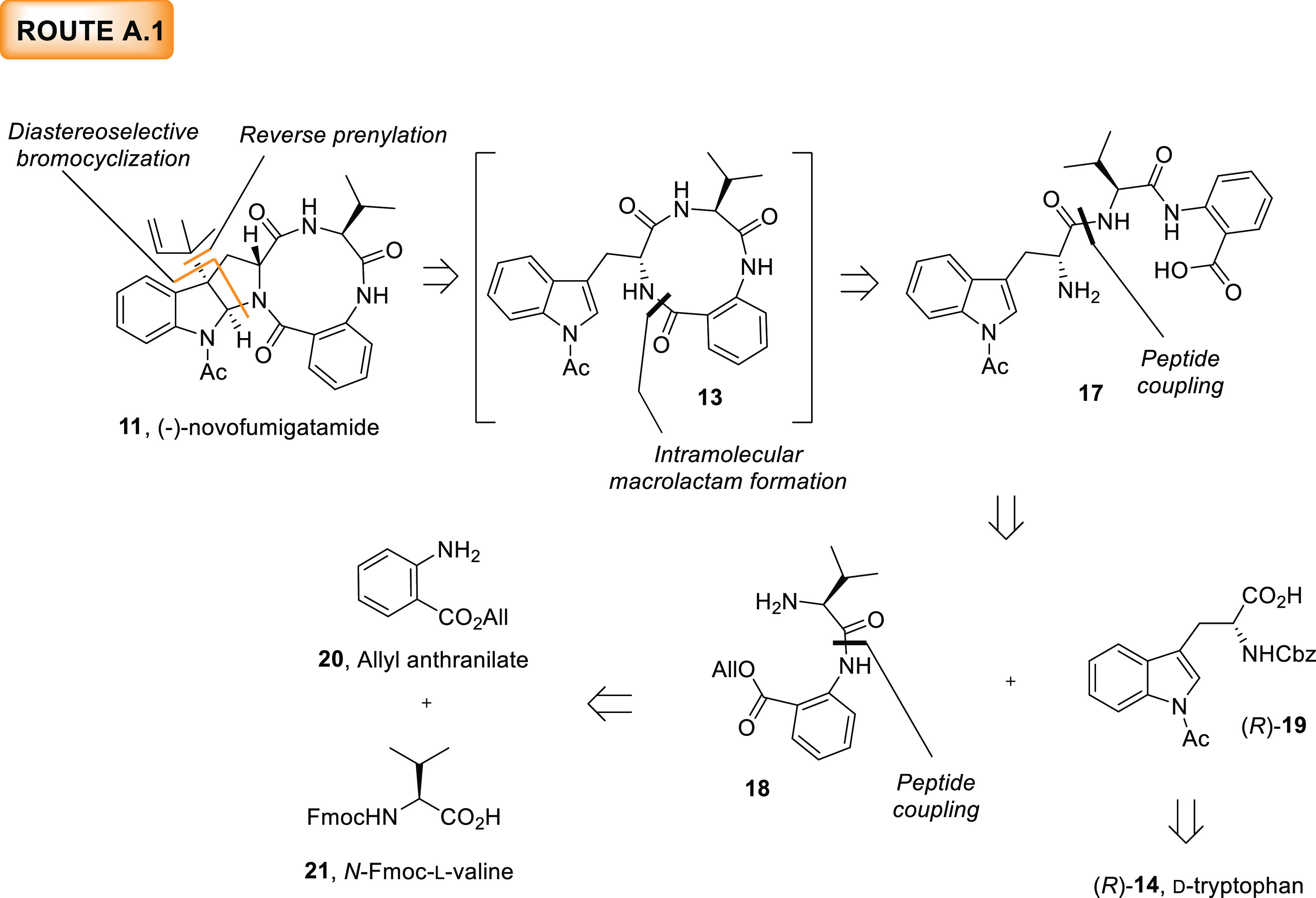
Retrosynthetic Analysis for the Proposed Structure of (−)-Novofumigatamide (**11**) Following Route A.1

We initiated our investigation with a synthetic route from the group of strategies A. According to the retrosynthetic analysis for route A.1 outlined in Scheme 2, the installation of the reverse prenyl group was postponed to be the last step of the synthesis.^33,34^ The assembly of the skeleton of (−)-novofumigatamide (**11**) would be achieved *via* a sequential macrolactamization and a diastereoselective bromocyclization,^35^ which would allow for the construction of the hexahydropyrrolo[2,3-*b*]indole skeleton without isolation of the presumably unstable macrolactam intermediate (**13**). The acyclic macrolactam precursor (**17**) could be traced back to appropriate condensations of commercially available d-tryptophan (**14**), allyl anthranilate (**20**), and *N*-Fmoc-l-valine (**21**) amino acid units.

**Scheme 3:**
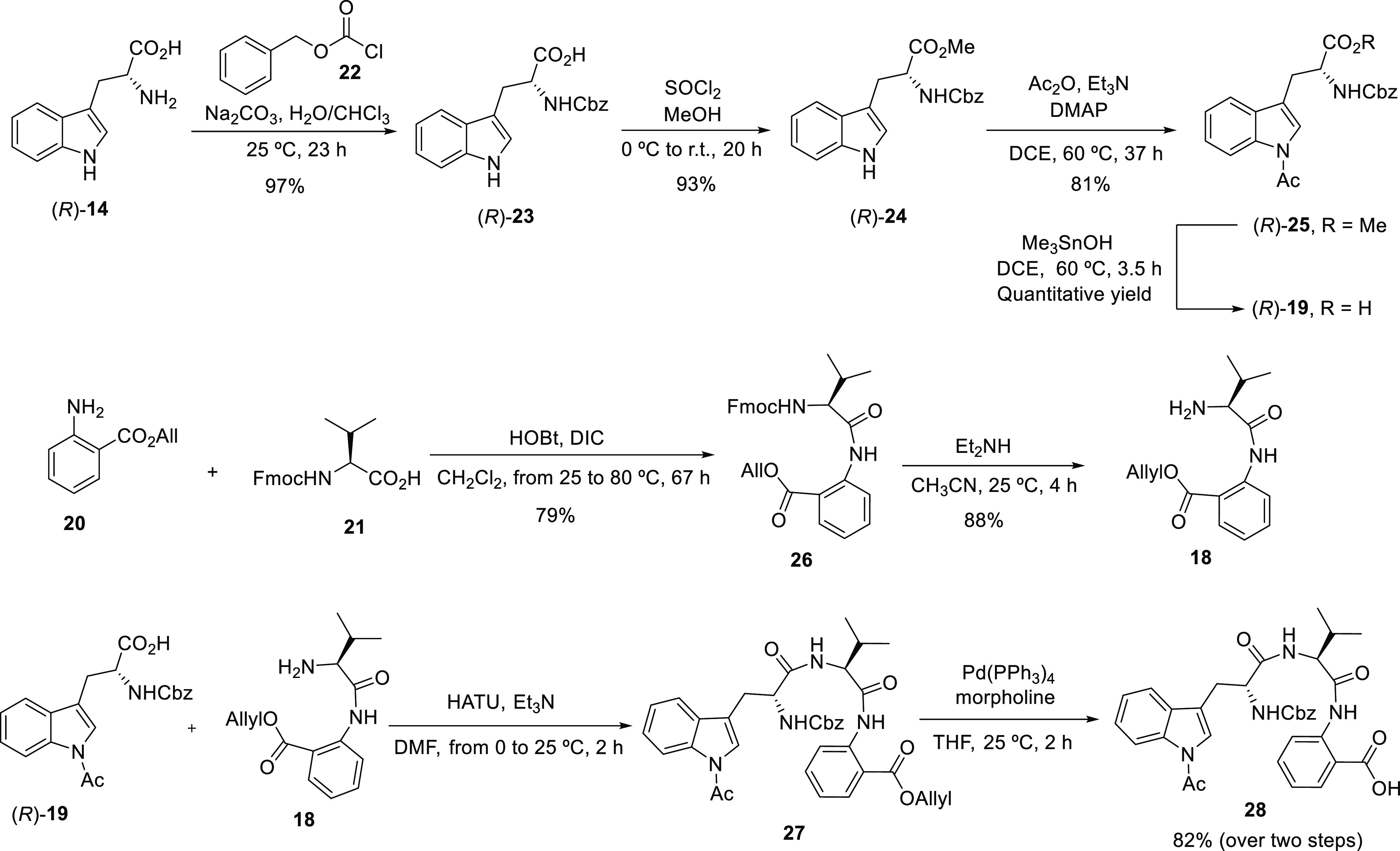
Synthesis of Acyclic Fragment **28**, the Precursor of the Proposed Structure of (−)-Novofumigatamide (**11**) Following Route A.1

**Scheme 4:**
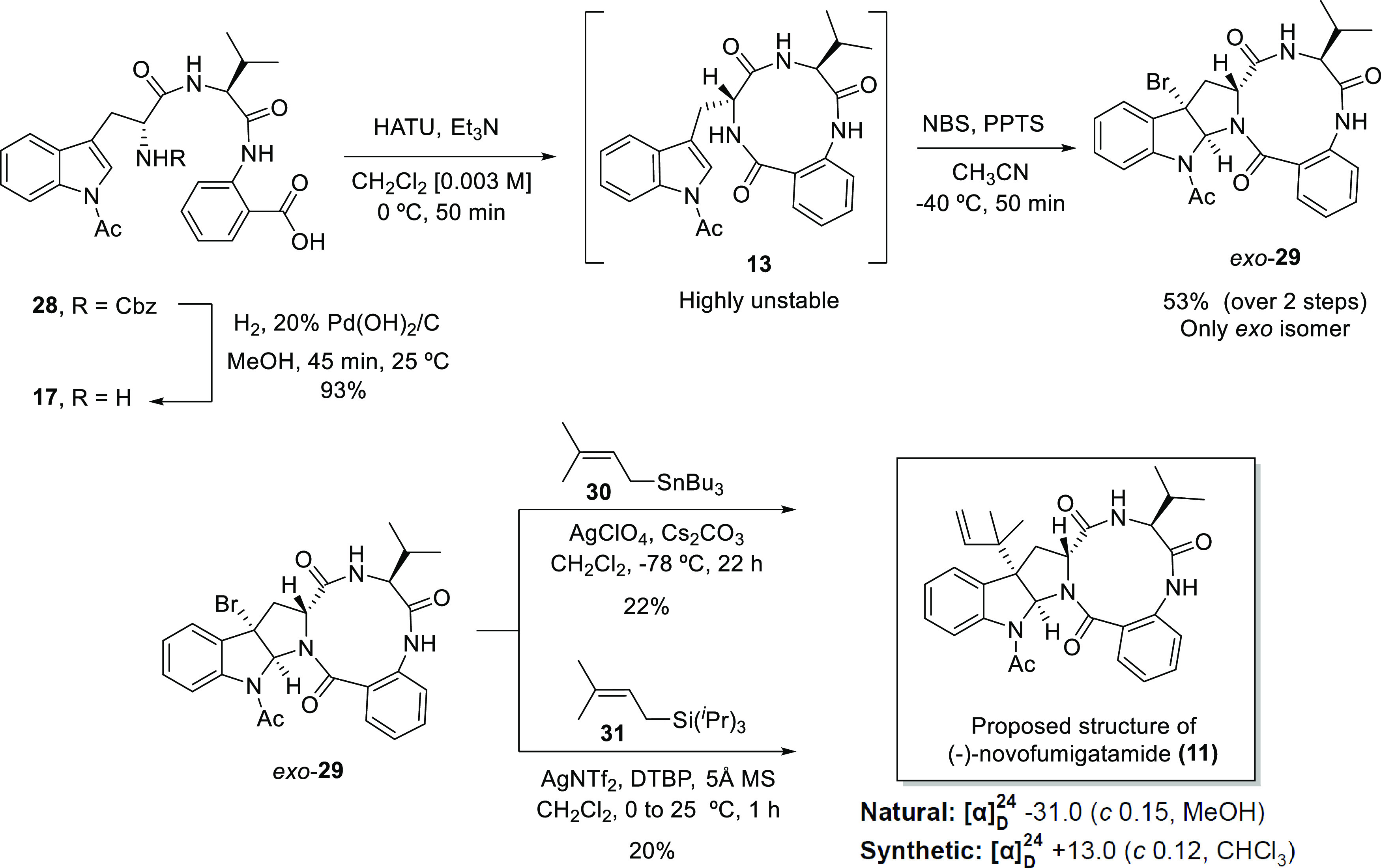
Key Steps on the Total Synthesis of the Proposed Structure of (−)-Novofumigatamide (**11**) Following Route A.1

Schemes 3 and 4 outline the synthetic sequence optimized for the synthesis of the proposed structure of (−)-novofumigatamide (**11**) following route A.1. The total synthesis of this putative natural product structure began with the preparation of main fragments **18** and (*R*)-**19**. The tryptophan derivative (*R*)-**19** was efficiently synthesized in four steps starting from commercially available d-tryptophan (**14**). Cbz-protection of the amine using benzyl chloroformate (**22**) and Na_2_CO_3_, formation of the methyl ester, and acetylation of the indole nitrogen with acetic anhydride under DMAP catalysis^36^ afforded the fully protected tryptophan derivative (*R*)-**25** in good overall yield. The hydrolysis of the latter using a classical saponification protocol with LiOH and a THF/H_2_O solvent system led to the simultaneous deprotection of the *N*-acetyl group due to its lability under basic conditions. Therefore, this transformation was attained using trimethyl tin hydroxide at 60 °C in DCE,^37^ which afforded the desired fragment (*R*)-**19** in quantitative yield. The preparation of the dipeptide unit **18** with the free amine group as required to merge with the carboxylate tryptophan derivative was envisioned as a two-step sequence: first, the coupling between commercially available allyl anthranilate (**20**) and *N*-Fmoc-l-valine (**21**) in the presence of HOBt and DIC as coupling reagents to provide the fully protected dipeptide **26** (79% yield), and then the *N*-Fmoc deprotection in the presence of diethylamine (88% yield).

With the two fragments **18** and (*R*)-**19** in hand, optimization of the reaction parameters was carried out on the subsequent condensation in the presence of HATU and Et_3_N. After adjustment of the reaction time and the equivalents of the reagents, full conversion to the acyclic intermediate **27** was achieved with the use of DMF as the solvent at 25 °C. However, as the resulting acyclic product **27** turned out to be rather unstable, moderate yields were obtained after cumbersome chromatographic purifications. Due to the problems encountered, the following deprotection of the allyl ester moiety by treatment with Pd(PPh_3_)_4_ and morpholine in THF was accomplished with the crude mixture, and only a final purification of the deprotected intermediate **28**, which was obtained in a combined 82% yield, was performed. Since the presence of rotamers in all the acyclic and cyclic intermediates complicated NMR signal assignment, from this point of the synthetic route onward, NMR spectroscopic data had to be recorded at high temperatures (*T* ≥ 323 K) with the aim of observing sharply defined peaks.

During our investigations, we realized the importance of having highly pure substrates in order to achieve successful transformations in some of the reactions of the sequence. This was the case for the removal of the *N*-Cbz group on **28**. Although hydrogenation in the presence of Pd/C (10%) led to the recovery of the starting material, the use of Pearlman’s catalyst (20% Pd(OH)_2_/C)^38,39^ with a highly pure substrate precursor (**28**) allowed us to isolate the fully deprotected amino acid intermediate **17** in 93% yield (Scheme 4). This compound was insoluble in most organic solvents, which precluded its purification by column chromatography. The purity of the intermediates was particularly crucial in the next two steps of the synthesis, the most challenging transformations of the synthetic route. Macrolactam formation from intermediate **17** was accomplished using high dilution conditions to favor the intramolecular reaction. Although different coupling reagents (HATU, HBTU, HOBt, EDC, COMU), bases (Et_3_N, DIPEA), solvent systems, temperatures and reaction times were employed, the desired product could not be isolated. With the use of our standard conditions for amide formation promoted by HATU and Et_3_N, the macrolactam formation proceeded very fast within the first 1 h of reaction; however, attempts to purify the product were unsuccessful. Likewise, efforts to preserve the integrity of the desired product in the crude mixture by keeping it overnight in a freezer (−30 °C) were fruitless since the presence of decomposition products was also observed. Given these results, it became obvious that a fast conversion of unstable macrolactam **13** to the next intermediate of the synthetic route was mandatory. Thus, the crude arising from the macrolactam formation was immediately used in the subsequent diastereoselective bromocyclization,^35^ which was also subjected to optimization through screening a variety of conditions (see the SI for further details).^40−42^ It was concluded that a sequential macrolactam formation and diastereoselective bromocyclization in the presence of NBS and PPTS in CH_3_CN at −30 °C was the optimal procedure to build the bromohexahydropyrrolo[2,3-*b*]indole skeleton *exo*-**29**, the direct precursor of the proposed structure of (−)-novofumigatamide (**11**). To our delight, the *exo* diastereomer (*ex*o-**29**) was obtained as a single product. The whole process is fast (100 min overall reaction time), occurs at low temperatures, mainly to avoid decomposition of the macrolactam (temperature can even be lowered to −30 °C without affecting the conversion), and proceeds with high diastereoselectivity (Scheme 4). To the best of our knowledge, this represents the first example of a diastereoselective bromocyclization achieved through the nucleophilic attack of an amide embedded in a macrolactam ring.

The final installation of the reverse prenyl group on *exo*-**29** was achieved through the silver-promoted Friedel–Crafts alkylation developed by Qin *et al.*(33) This methodology requires the use of a silver salt and a base to generate a carbocation, which is trapped *in situ* by a prenyl tributylstannane nucleophile (**30**). When *ex*o**-29** was treated with AgClO_4_ and Cs_2_CO_3_ in CH_2_Cl_2_ at −78 °C, in the presence of nucleophile **30**, the desired final product (−)-novofumigatamide (**11**) was obtained in a low yield but with an excellent *exo* diastereoselectivity, which was determined by the preferred *cis*-fusion of the tricyclic system. An alternative alkylation protocol using instead tri*iso*propylprenyl silane (**31**) as a nucleophile and DTBP as a base in the presence of silver bis(trifluoromethylsulfonyl)imide (AgNTf_2_) took place with a similar yield and selectivity (Scheme 4).^34^ To our surprise, neither the spectroscopic data nor the optical rotation of the synthetic material matched those reported for the natural compound.^24^

**Scheme 5:**
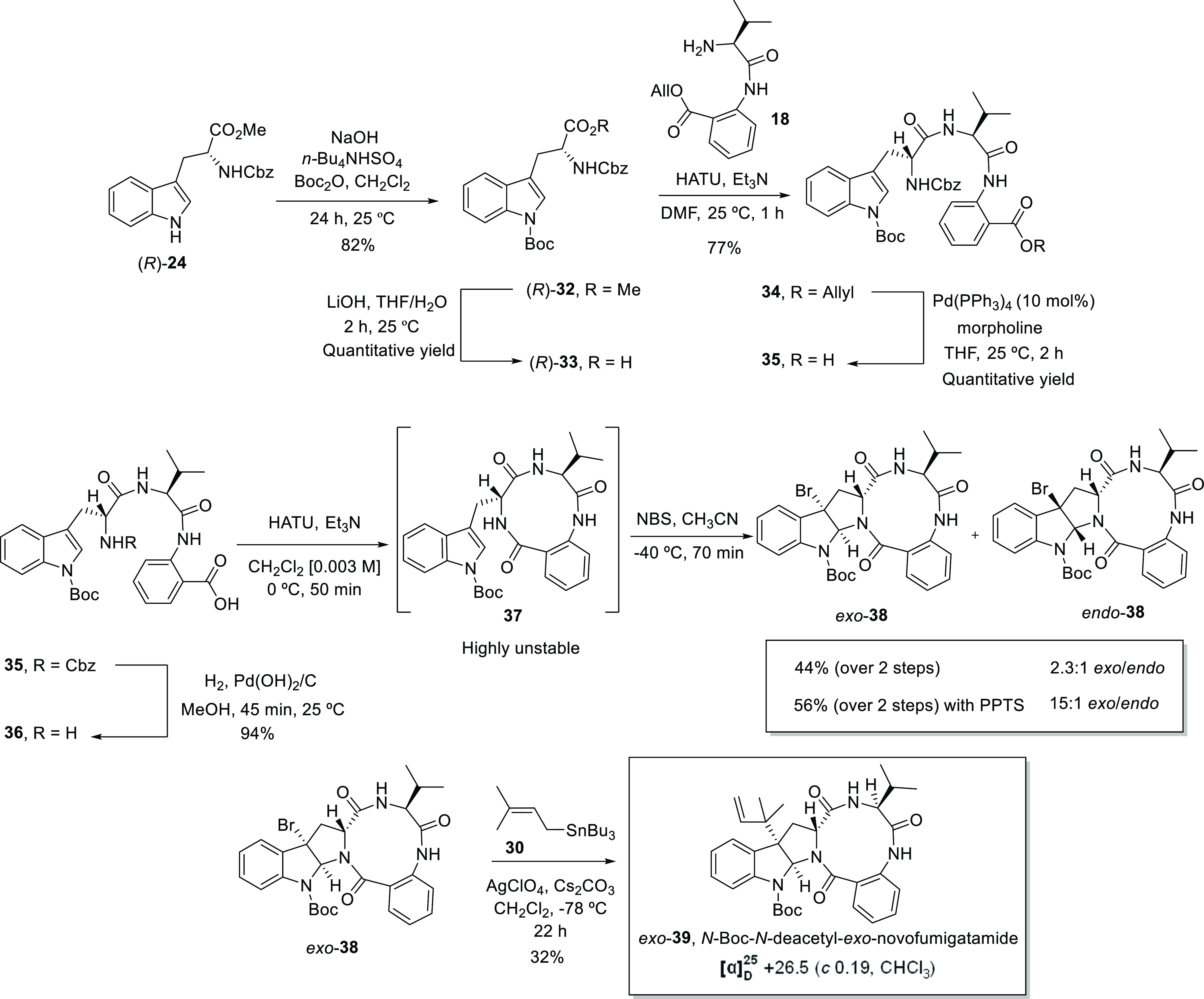
Total Synthesis of the *N*-Boc-*N*-deacetyl-*exo*-Novofumigatamide Analogue *exo*-**39**

In parallel to the synthesis of the proposed structure of (−)-novofumigatamide (**11**), *N*-Boc-protected structural analogue *exo*-**39** was also prepared (Scheme 5). The development of a synthetic approach toward this new target aided to optimizing different synthetic steps, common to the routes to both compounds, and to discarding alternative relative and absolute configurations of the natural product, as explained below. *N*-Protected tryptophan carboxylic acid (*R*)-**33** was prepared in two steps from common intermediate (*R*)-**24**. Boc-protection of the indole nitrogen on **24** by reaction with Boc_2_O under phase-transfer catalytic conditions furnished the fully protected tryptophan derivative (*R*)-**32**, which was subsequently exposed to classical saponification conditions to obtain the free carboxylic acid on (*R*)-**33**. Condensation of fragments (*R*)-**33** and dipeptide **18** and the consecutive removal of the *O*-allyl and *N*-Cbz protecting groups to afford **36** were performed following the conditions depicted in Scheme 3, which allowed us to obtain the corresponding intermediates **34**, **35**, and **36** in yields ranging from very good to excellent. As expected, the two-step macrolactam formation–bromocyclization sequence furnished brominated precursor **38** as a 15:1 mixture of *exo*/*endo* diastereomers in 56% yield over the two steps. Moreover, the same experimental procedure performed in the absence of PPTS resulted in a 2.3:1 *exo*/*endo* diastereomeric mixture, which facilitated the isolation and full characterization of the *endo*-**38** product. Although the use of bromine led to the isolation of the *endo* diastereomer as the major product, a concomitant decrease in the yield was obtained due to the formation of decomposition byproducts (see the SI for further details). To complete the synthesis, a diastereoselective reverse prenylation using the protocol described above converted the *exo*-**38** into the *N*-Boc-*N*-deacetyl-*exo*-novofumigatamide product (*exo*-**39**) in a slightly higher yield than the corresponding *N*-acetylated analogue (**11**). Comparatively, the *N*-Boc-protected intermediates are less reactive and more stable than their *N*-acetylated counterparts, which accounts for the higher yields observed in their preparation. For instance, *N*-Boc-intermediate **36** turned out to be much less reactive than the *N*-acylated analogue **17** towards macrolactam formation since this reaction did not proceed at temperatures lower than 0 °C, whereas the same process with **17** could be attained at −30 °C (data not shown). Regardless of this difference, *N*-Boc and *N*-acetyl intermediates share key common features. As noticed above, the presence of rotamers complicated ^1^H NMR analysis, and high temperatures and/or the use of DMSO-*d_6_* as deuterated solvent were required to observe sharp NMR signals. This was also the case for the cyclic intermediates and remarkably for the final products, namely, synthetic (−)-novofumigatamide (**11**) and *N*-Boc-*N*-deacetyl-*exo*-novofumigatamide (*exo*-**39**). The acquisition of the NMR spectra in CDCl_3_ or DMSO-*d_6_* as deuterated solvents at 298 K showed the presence of two major rotamers, whereas at higher temperatures (328 or 343 K, respectively), broad peaks were observed for some of the key signals (see the SI). This result is in striking contrast to the original spectra recorded and kindly provided by Hosoe and coworkers (data not shown),^24^ which showed well-defined NMR peaks in both solvents at 298 K.

**Figure 2:**
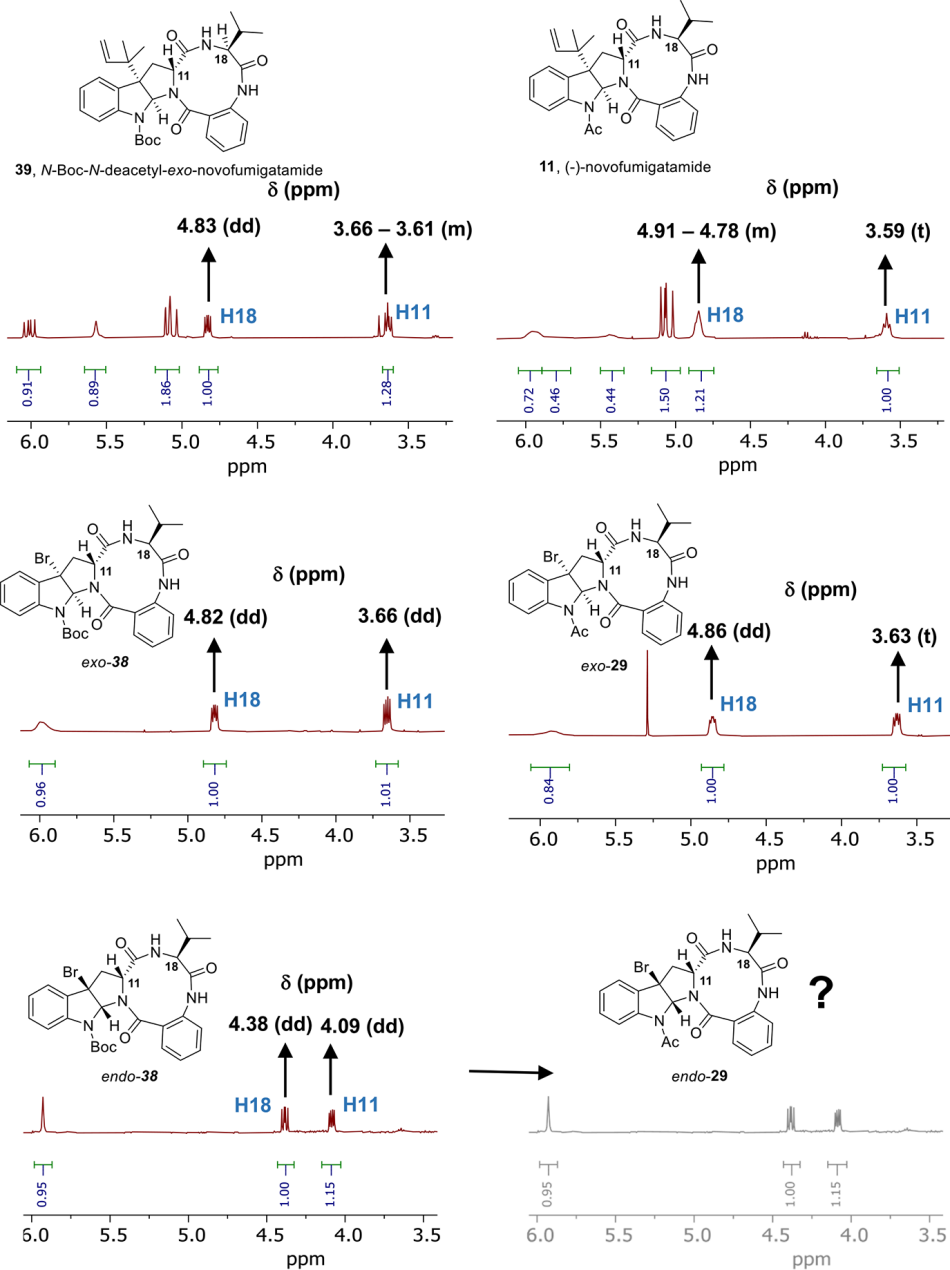
Comparison of the ^1^H NMR (collected at 323 K or 328 K) spectra of the bromo-precursors *exo*-**30**, *exo*-**38**, *endo*-**38**, and the final products (−)-novofumigatamide (**11**) and *N*-Boc-*N*-deacetyl-*exo*-novofumigatamide (**39**).

**Scheme 6:**
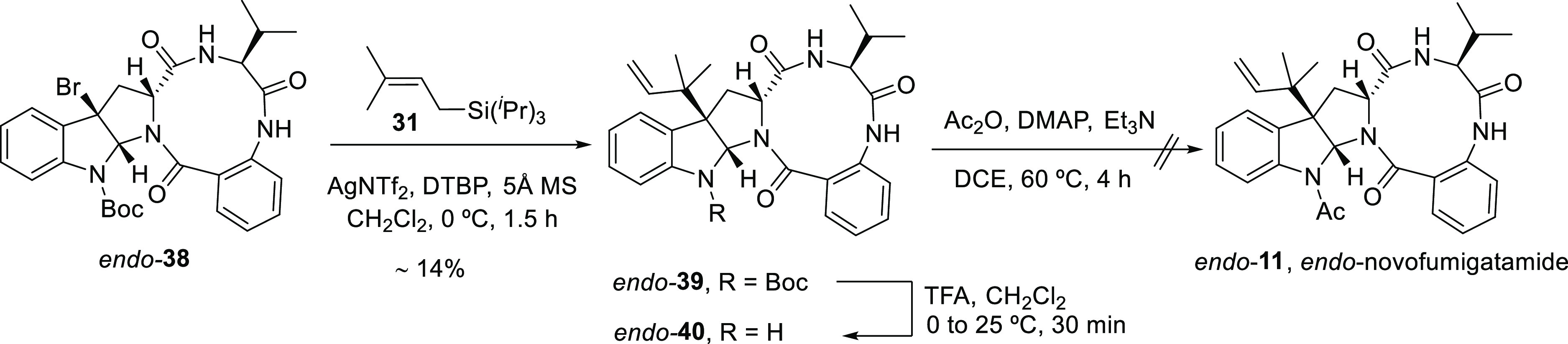
Synthetic Approach toward *endo*-Novofumigatamide (*endo*-**11**)

Comparison between the ^1^H NMR data of the final *exo*-products **11** and **39** and their brominated precursors revealed interesting features. The chemical shifts of the key proton signals at the α-enolizable positions, corresponding to H11 and H18 (Figure 1) in natural (−)-novofumigatamide (**11**), did not significantly change when a *N*-Boc or a *N*-acetyl group are present in the indole nitrogen. In accordance, the signals corresponding to H11 and H18 in (−)-novofumigatamide (**11**) and *N*-Boc-*N*-deacetyl-*exo*-novofumigatamide *exo*-**39**, both *exo* isomers, are almost identical multiplets placed at δ_H11_ ∼ 3.7–3.5 ppm and δ_H18_ ∼ 4.9–4.7 ppm, respectively (Figure 2). Likewise, the replacement of the bromine atom in *ex*o-**30** and *ex*o-**38** precursors by a reverse prenyl group to give rise to the final products **11** and **39** did not significantly alter the chemical shifts of the ^1^H NMR signals corresponding to H11 and H18. On the other hand, when we focused our attention on the corresponding H11 and H18 signals for the *endo*-**38** diastereomer, we noticed the important displacement of δ ∼ 0.5 ppm on the chemical shift of both protons with respect to the *exo*-**38** diastereomer. Hence, δ_H11_ and δ_H18_ values and their relative position in the ^1^H NMR spectrum represent very characteristic indicators of the stereochemistry of the products and, therefore, could be used as diagnostic signals for the straightforward assignment of the configuration of intermediates and final products. If the experimental evidence that chemical shifts of both α-enolizable protons remain unchanged after Br/prenyl or *N*-Boc/*N*-acetyl replacement is extrapolated to the *endo* isomers, then *endo*-**38** should not be a precursor of the natural product, whose synthesis could be envisioned from this bromo precursor following installation of a reverse-prenyl group, *N*-Boc deprotection, and acetylation of the indole nitrogen. Despite this negative evidence, the latter route toward *endo*-novofumigatamide (*endo*-**11**) was attempted (Scheme 6). *Endo*-bromo precursor **38** or the corresponding carbocation formed after treatment with a silver salt proved to be more unstable than those of the corresponding *exo* diastereomer since reverse prenylation using the conditions described above by Hidetoshi and co-workers^34^ led to the isolation of a highly impure fraction of the desired product *endo*-**39**, which was subsequently treated with TFA to afford the *N*-Boc-deprotected intermediate *endo*-**40**. The crude mixture of the previous reaction was submitted to the standard acetylation protocol, but a complex mixture of compounds was obtained, suggesting that all *endo* intermediates are more unstable, and likely less reactive, than the corresponding *exo* isomers. This result is in accordance with recent reports, where the problems encountered in the *N*-acylation of some tetracyclic *endo*-compounds have been proposed to rest on the sterically crowded environment at the N1-position of these diastereoisomers.^43^ As expected, prenylated *endo*-**39** showed similar chemical shifts for H11 and H18 than brominated precursor *endo*-**38**, thus confirming the trend observed for the *exo* isomers. Assuming that the previous observations are general, the replacement of the *N*-Boc in *endo*-**39** by an *N*-acetyl group should not provide the natural product, in which the signals for H11 and H18 appear at δ ∼ 5.22 ppm and δ ∼ 4.43 ppm, respectively.

As mentioned in the introductory paragraph, natural products bearing a hexahydropyrrolo[2,3-*b*]indole skeleton, with either *exo* or *endo* relative configurations, fused with additional cyclic structures, have been isolated and characterized. An analysis of the chemical shifts of the α-enolizable hydrogen of the tryptophan units in these molecules reveals that the H-*exo* protons fall within a typical range of δ ∼ 3.5–4.0 ppm, whereas H-*endo* protons are pulled downfield to chemical shifts higher than δ ∼ 4 ppm. These trends were corroborated in our brominated precursors and final products. According to these average values, natural (−)-novofumigatamide (**11**) would fit into the *endo* range given the chemical shift at δ ∼ 4.43 ppm assigned to H11. Further evidence supporting this hypothesis is related to the fact that all metabolites isolated from *Aspergillus novofumigatus* are l-tryptophan-derived *endo* diastereomers, as is the case of *epi*-aszonalenins A-C,^22^ novoamauromine,^14^ and asnovolenins.^23^ All these data made us tentatively assign the relative configuration of the hexahydropyrrolo[2,3-*b*]indole framework of natural (−)-novofumigatamide as *endo* arising from l-tryptophan. This conclusion suggests that (−)-novofumigatamide might be obtained through the incorporation of a l-valine residue at some point of the biosynthetic pathway toward *epi*-aszonalenins. The absolute configuration of l-valine in natural (−)-novofumigatamide has been determined by Marfey’s analysis,^25^ whereas the configuration of the remaining chiral centers of the molecule has been established on the basis of 1D and 2D NMR spectroscopic data. Taking into account the reliability of Marfey’s method,^25^ we envisioned that the absolute configuration of the stereocenter arising from the tryptophan unit could have been incorrectly assigned.

### Route A.1 and Route B.1 toward the *Exo and Endo* Diastereomers of l-Trp-Novofumigatamide (l-Trp-**11**)

**Scheme 7:**
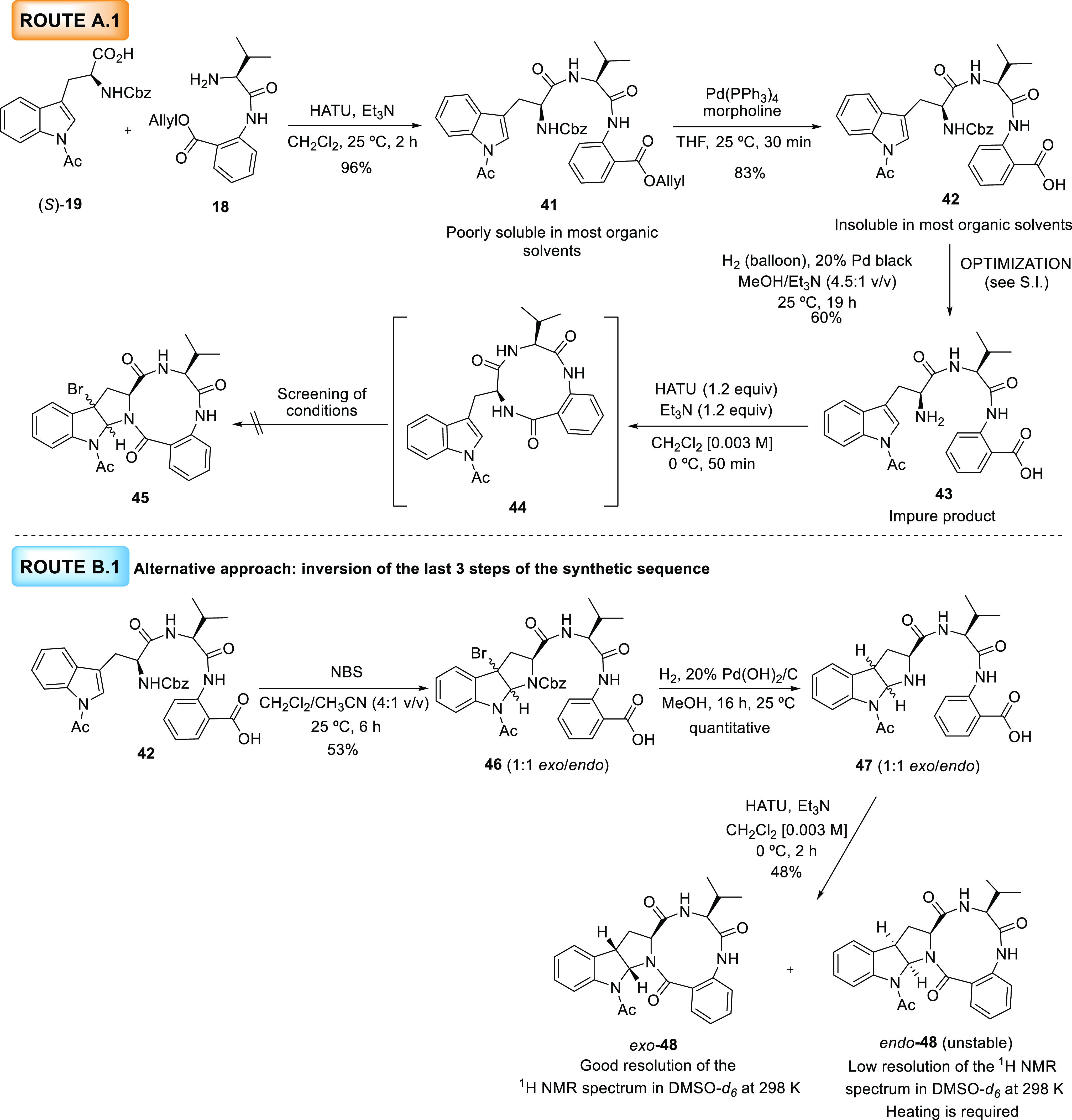
Synthetic Approaches toward l-Trp-Novofumigatamide (l-Trp-**11**)

Then, we set out to prepare a new diastereomer of (−)-novofumigatamide. Scheme 7 illustrates the synthetic sequence toward the diastereomer of this natural product using l-valine and l-tryptophan as starting chiral substrates and following the same transformations depicted in Schemes 3 and 4. Surprisingly, the different solubility shown by the intermediates of this synthetic route with respect to their diastereomeric counterparts dramatically altered their reactivity, as demonstrated below. Condensation of dipeptide **18** and the enantiomeric tryptophan derivative (*S*)-**19** using the previously optimized conditions furnished acyclic intermediate **41**, which was poorly soluble in most organic solvents and, therefore, difficult to purify by column chromatography in a large-scale synthesis. The subsequent *O*-allyl deprotection upon treatment with Pd(PPh_3_)_4_ and morpholine proceeded efficiently. However, the corresponding carboxylic acid **42** was insoluble in most organic solvents, particularly in MeOH, which was the solvent employed for the following *N*-Cbz deprotection step in the presence of Pearlman’s catalyst. Foreseeably, this fact caused the full recovery of the starting material. The failure of the previous step forced us to screen new conditions to achieve this transformation (see the SI for further details). The hydrogenation in the presence of palladium black (20%) in a mixture of MeOH and Et_3_N as the solvent afforded the desired fully unprotected product **43** in a 60% yield, although as a moderately pure compound. As demonstrated during the development of the route toward the proposed structure of d-Trp-novofumigatamide (d-Trp-**11**), the high purity of the fully unprotected acyclic compound was crucial to obtain satisfactory results in the ensuing macrolactam formation and bromocyclization reactions. Macrolactam formation with **43** using the standard conditions afforded the desired macrolactam **44**, which proved to be even less stable than the diastereomeric counterpart **13**, since several decomposition byproducts were already observed along the reaction course. Likely due to the instability of this macrolactam, the bromocyclization led only to complex mixtures of compounds after several attempts under different reaction conditions (see the SI for further details). In view of these drawbacks, the order of the last three steps of the synthetic sequence was altered, the macrolactam formation was postponed to the last step of the synthesis, and the bromocyclization was performed in advance of the *N*-Cbz deprotection (Scheme 7, route B.1). To address the lack of solubility of the *N*-Cbz protected acyclic tripeptide **42**, acetonitrile was added to the bromocyclization reaction and the temperature was increased to 25 °C with respect to the standard conditions; nevertheless, these changes did not help solubilize this intermediate. To our surprise, the solubility was not relevant since the reaction reached completion and the *exo*/*endo* products (**46**) were obtained in a 1:1 ratio, although in moderate yield (53%). Since the separation of the *exo* and *endo* products by column chromatography was cumbersome at this stage, the remaining steps were performed with this mixture of diastereomers. Unfortunately, simultaneous reduction of the C–Br bond and *N*-Cbz deprotection occurred under hydrogenation conditions. The macrolactam formation was performed with the reduced *exo*/*endo* derivatives **47** using the standard conditions, and both diastereomers (*endo*-**48** and *exo*-**48**) were separated and characterized at this final step. As observed for other *exo*/*endo* pairs, stability and NMR signal resolution of both diastereomers differed considerably since *exo*-**48** could be characterized in DMSO-*d_6_* at 298 K, whereas *endo*-**48**, which required higher temperatures to observe defined NMR peaks, could not be fully characterized due to its instability.

### Additional Type B Strategies toward the *Exo and Endo* Diastereomers of l-Trp-Novofumigatamide (l-Trp-**11**)

#### Route B.2

**Scheme 8:**
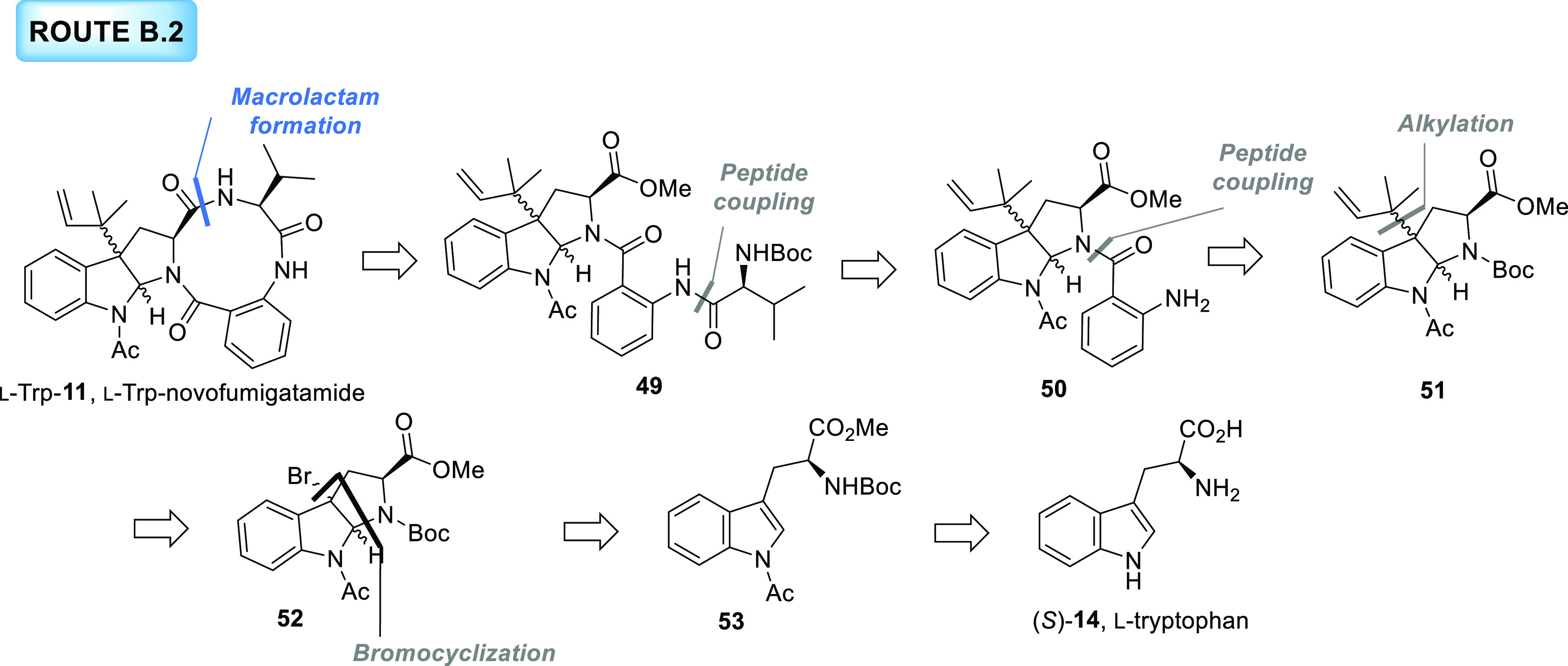
Retrosynthetic Analysis for Route B.2 toward the Proposed Structure of l-Novofumigatamide (l-Trp-**11**)

The failure of the previous two synthetic routes prompted us to explore new alternatives. As a first option, we focused our attention on a synthetic approach from the group of strategies B. In our new retrosynthetic proposal, the intramolecular macrolactam formation through the formation of the amide bond between the tryptophan and the valine units was selected as key and last transformation (Scheme 8). The acyclic intermediate **49** resulting from this disconnection would be prepared from the hexahydropyrrolo[2,3-*b*]indole derivative **51** after the sequential coupling reactions in the required order with valine and anthranilic acid units. The construction of the hexahydropyrrolo[2,3-*b*]indole framework, in turn, would occur through a diastereoselective bromocyclization from the corresponding tryptophan derivative **53**. Importantly, the *N*-Cbz protecting group on the tryptophan unit was replaced by a *N*-Boc group to avoid the problems found in the previous route.

**Scheme 9:**
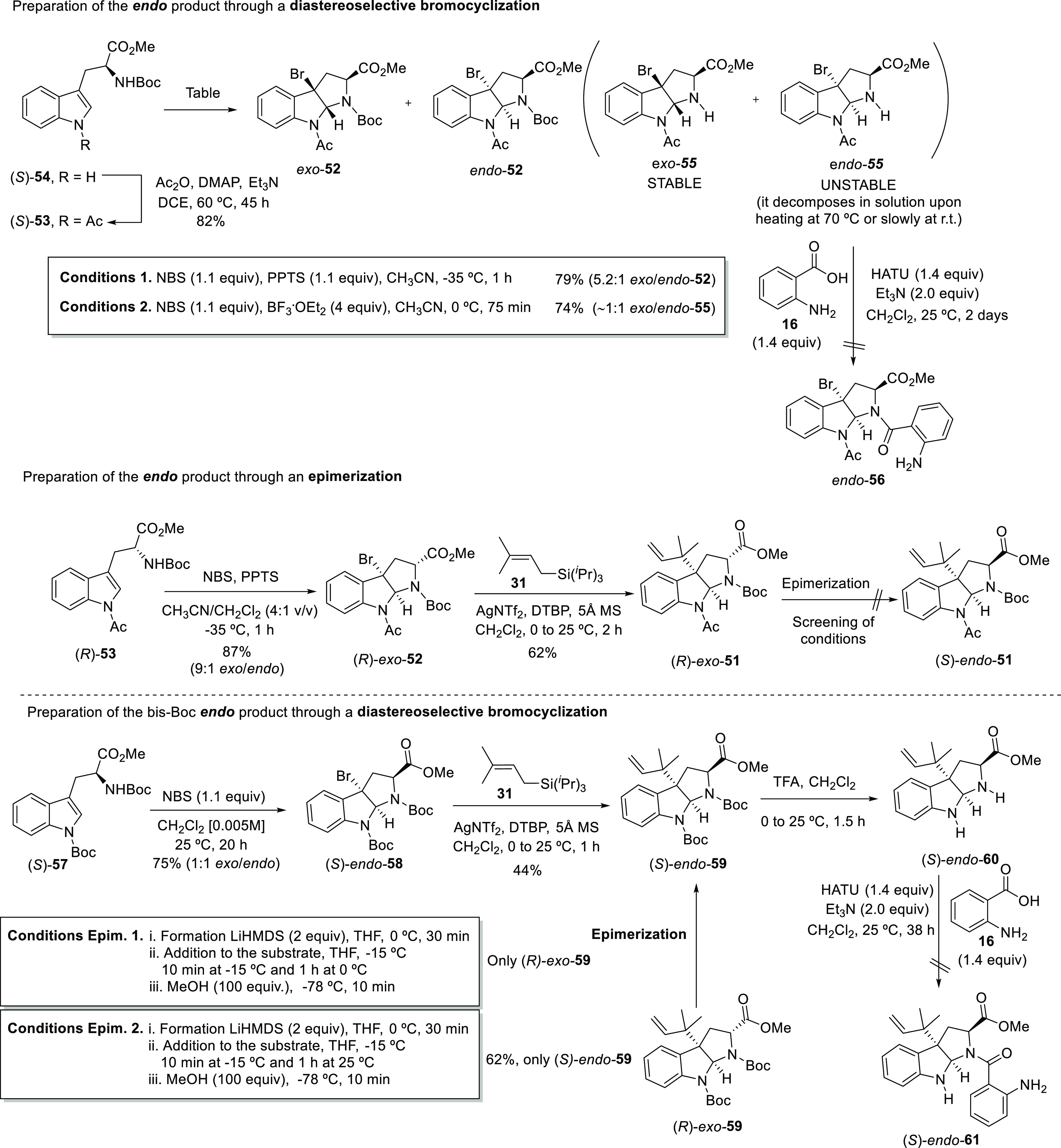
Synthetic Approach toward l-Trp-Novofumigatamide (l-Trp-**11**) Following Route B.2

The new synthetic sequence commenced with the preparation of the tryptophan derivative **53** from precursor **54**(44) using the acetylation conditions described above (Scheme 9). The conclusions drawn concerning the correct configuration of the natural product made us focus our efforts on the preparation of the *endo* diastereomer (l-Trp-*endo*-**11**). A literature search revealed that for some particular substrates, the control of reaction conditions allows us to perform diastereoselective bromocyclizations biased toward the *endo* diastereomer.^42^ Nevertheless, although several conditions were examined on our starting material **53** (see the SI for further details), the *endo*-**52** isomer could not be obtained as a major product. By using a classical procedure in the presence of NBS and PPTS in CH_3_CN at −35 °C for 1 h, the corresponding 3a-bromo-hexahydropyrrolo[2,3-*b*]indole **52** was obtained as a 5.2:1 mixture of *exo*/*endo* products, which were difficult to separate by column chromatography. The addition of boron trifluoride etherate has been reported to increase the *endo*/*exo* ratio for some substrates.^45,46^ However, in our case, the addition of 4 equivalents of this Lewis acid resulted in the bromocyclization and a subsequent deprotection of the *N*-Boc group as an undesired reaction, which generated the unprotected hexahydropyrrolo[2,3-*b*]indole **55** as an almost equimolar mixture of *endo* and *exo* diastereomers. As observed before, the two diastereomers showed different stabilities, the *endo* product being less stable, since it decomposed in solution immediately upon heating or upon standing at ambient temperature for several hours. As the unexpected *N*-Boc-deprotection left the pyrrolidine nitrogen ready to be coupled, the following condensation with anthranilic acid **16** employing the standard conditions described by Carreira and co-workers with Et_3_N and HATU was carried out.^47^ Unfortunately, only a mixture of decomposition products and recovered starting material was obtained from the reaction mixture after 2 days of reaction. The lack of nucleophilicity of the pyrrolidine nitrogen atom in *endo* diastereomers of hexahydropyrrolo[2,3-*b*]indoles has been recently attributed to a deactivation through an n_N_ → σ_C-N_* interaction, which is less pronounced or inexistent in the *exo* counterparts.^48^ We also hypothesized that the electronegativity of the bromine atom in C3a could be playing a role in the lower reactivity of this nitrogen atom.

*Endo* diastereomers of hexahydropyrrolo[2,3-*b*]indoles have also been selectively prepared through an epimerization of the enolizable Cα-position of the corresponding *exo* isomers, a transformation that occurs through a base-promoted generation of an enolate and a subsequent kinetic protonation at low temperature. Such a process usually delivers the thermodynamic *endo* products for simpler analogous compounds with hexahydropyrrolo[2,3-*b*]indole frameworks.^49−51^ The failure to promote the direct diastereoselective bromocyclization of tryptophan derivative **53** prompted us to try this classical indirect methodology to obtain the *endo*-pyrrolidinoindoline **52**. This alternative required the preparation of enantiomer (*R*)-**53** and its subsequent diastereoselective bromocyclization to obtain the *exo* product as the major compound. With this purpose in mind, conditions 1 depicted in Scheme 9 (for enantiomer *S*-**53**) were carried out with a different mixture of solvents (CH_3_CN/CH_2_Cl_2_ 4:1 *v/v*), which allowed us to increase the *exo*/*endo* ratio to 9:1. As described in the literature, treatment of bromo-hexahydropyrrolo[2,3-*b*]indoles with a base generates an enolate that immediately evolves to give rise to a cyclopropylazetoindoline.^52^ Thus, the reverse prenyl group was introduced at this stage of the synthesis in order to block this position. Using the standard protocol with AgNTf_2_, the desired product (*R*)-*exo*-**51** was obtained in 62% yield. The epimerization of this product was first attempted using lithium *bis*(trimethylsilyl)amide (LiHMDS)^53^ as a base at −15 °C and further quenching with MeOH at −78 °C. Nevertheless, only the *exo* product was recovered from the reaction mixture. The increase of the equivalents of base or the temperature, the modification of reaction times, or the use of lithium di*iso*propyl amide (LDA) as an alternative base did not furnish the *endo* product (see the SI for further details). To test whether the *N*-acetyl group was playing a role in this result, the bis-*N*-Boc-(*S*)-*endo*-**59** analogue was prepared using the same two synthetic routes, namely, diastereoselective bromocyclization–alkylation or epimerization, already described for the acetylated analogues. In the particular case of this starting material, the diastereoselective bromocyclization using the high dilution conditions reported by Oguri and coworkers for the same substrate (*S*)-**57**^54^ led to a 1:1 mixture of the *endo*/*exo* isomers of (*S*)-**58**, which were separated by column chromatography. Then, the (*S*)-*endo*-**58** diastereomer was reverse-prenylated to give (*S*)-*endo*-**59** with a moderate yield. On the other hand, epimerization of bis-*N*-Boc-(*R*)-*exo*-**59** using the standard conditions reported in the literature and tried previously with the acetylated analogue failed to provide the desired (*S*)-*endo*-**59** product (Conditions Epimerization 1, Scheme 9). However, treatment of the starting material with LiHMDS at ambient temperature for 1 h led to the isolation of the desired isomer in 62% yield and as a single product (Conditions Epimerization 2, Scheme 9).^55^ The low yields obtained in this reaction could be attributed to the lower stability of the *endo* products, as mentioned above. Remarkably, the same conditions applied to (*R*)-*exo*-**51** did not furnish the corresponding *endo* product (see the SI), which confirmed the influence of the protecting groups on both nitrogen atoms in the performance of the epimerization process. With (*S*)-*endo*-**59** at hand, the removal of both *N*-Boc groups upon treatment with TFA at 0 °C delivered the corresponding NH-pyrrolidinoindoline product (*S*)-*endo*-**60** in a quantitative transformation. The crude of this reaction was subjected to the conditions described by Carreira and Ruchti for the coupling of hexahydropyrrolo[2,3-*b*]indole units with anthranilic acid and used before with *endo*-**55**.^47^ Unfortunately, after 38 h of reaction time, only traces of the desired product were detected by injection of aliquots in HPLC-MS. The lack of reactivity of *endo* compounds **55** and **60** toward the condensation with anthranilic acid **16** and the good results observed by Carreira and Ruchti for the condensation with (*R*)-*exo*-**60** under the same reaction conditions suggest that the substituent at the C3a bridge does not have an effect on the reactivity, but there are rather steric or electronic features of the *endo* diastereomer responsible for the failure of this condensation (*vide supra*: removal of the electronic density from the pyrrolidine nitrogen through a n_N_ → σ_C-N_* interaction^48^). On the other hand, the fact that the coupling of similar *endo*-products has been achieved for the condensation with *N*-Boc-valine,^54^ and as described by our own group, for the condensation with *N*-Boc-leucine^30^ with *endo* but not with *exo* diastereomers definitively suggests that a combination of features related with the substrate (group at position C3a – acetate, bromine, reverse prenyl, or a hexahydropyrrolo[2,3-*b*]indole unit – and/or relative configuration) and the coupling partner determines the final result. The lack of success in the condensation of *endo* diastereomers of pyrrolidinoindoline units discouraged us to continue with these synthetic routes.

#### Route B.3

**Scheme 10:**
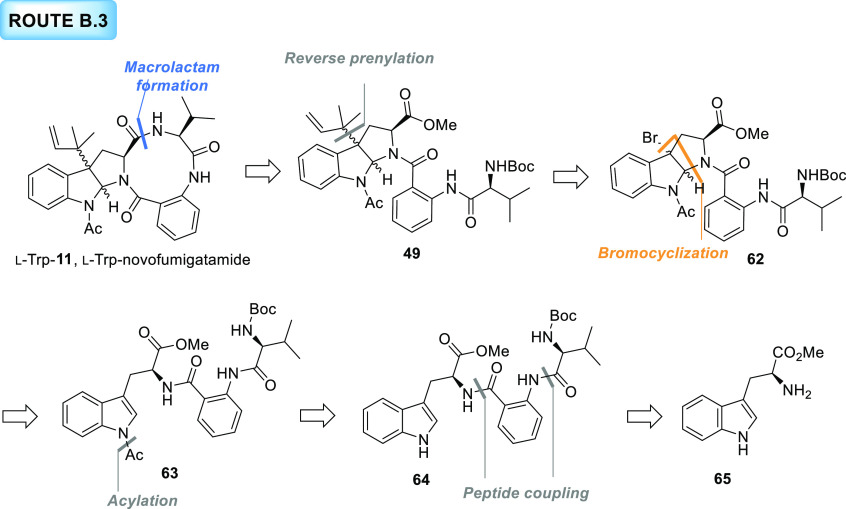
Retrosynthetic Analysis for the Proposed Structure of l-Trp-Novofumigatamide (l-Trp-**11**) Following Route B.3

Thus, we set out to investigate a new route toward the proposed structure of (−)-novofumigatamide after considering that it could be derived from l-tryptophan (Scheme 10, Route B.3). As in route B.2 (Scheme 8), the formation of the amide bond between the tryptophan and the valine units was envisioned as the last step of the synthesis from the acyclic precursor **49**, which in turn would be obtained through a diastereoselective bromocyclization and alkylation from acyclic intermediate **63**. Finally, the latter fragment already bearing all the units present in the natural product would be obtained after the consecutive couplings of valine and anthranilic acid fragments with l-tryptophan methyl ester (**65**).

**Scheme 11:**
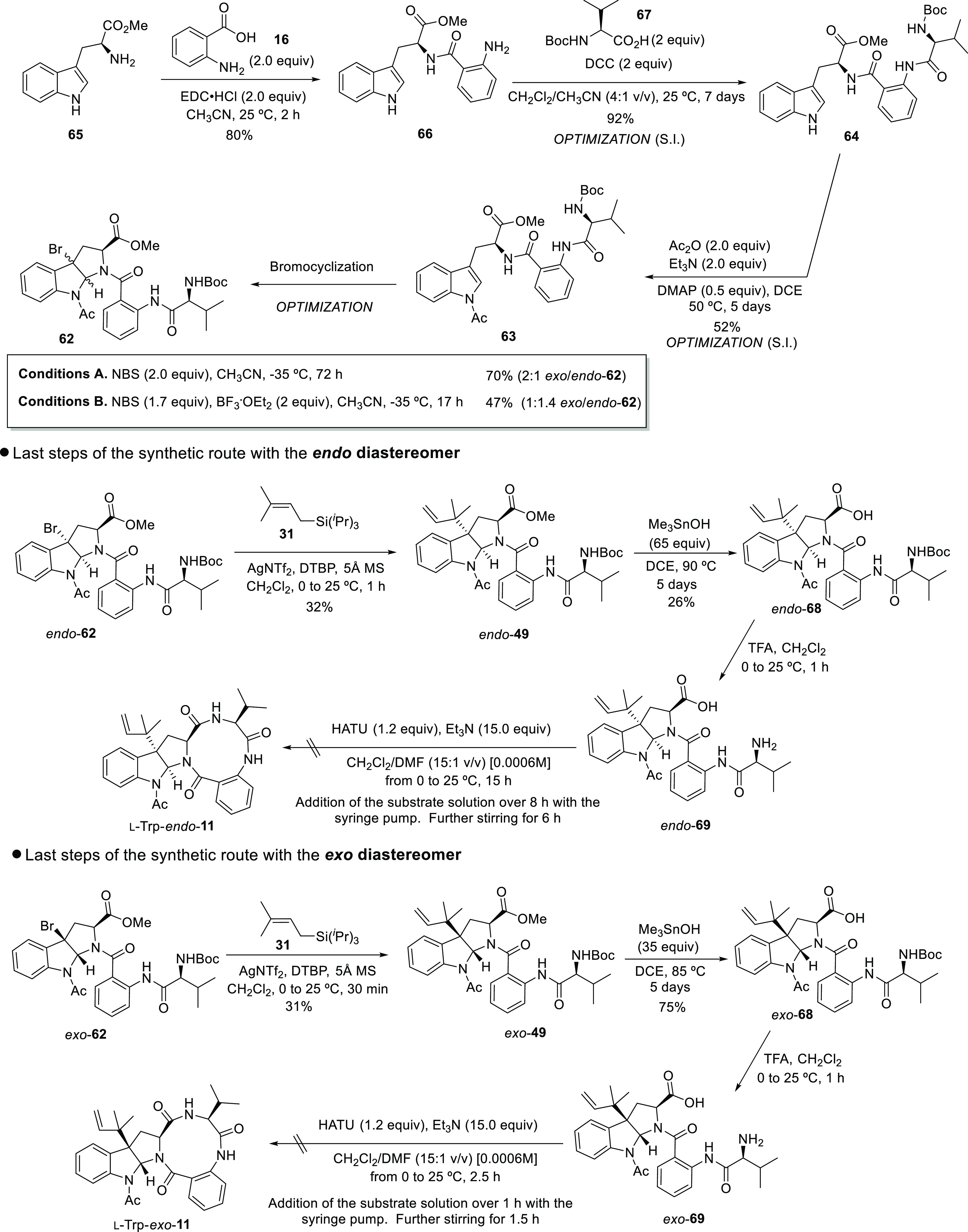
Synthetic Approach toward l-Trp-Novofumigatamide (l-Trp-**11**) Following Route B.3

The new synthetic sequence began with the condensation of l-tryptophan methyl ester **65** and anthranilic acid **16** in the presence of EDC as the coupling agent,^56^ which provided the desired product **66** after 2 h of reaction in 80% yield (Scheme 11). The subsequent assembly of the previous fragment with commercially available *N*-Boc-valine **67** required portionwise addition of the reagents (DCC and the valine derivative) over a long period of time in order to achieve optimal results (see the SI for further information on the optimization of this reaction). Unfortunately, incorporation of an acetyl group on the indole nitrogen of **64** turned out to also be a cumbersome transformation that was subjected to a screening of reaction conditions taking as a starting point the procedure used above for the acetylation of (*R*)-**24**. The selectivity was the major challenge in the acetylation of this nitrogen atom, as proven by the formation of secondary or decomposition byproducts, which complicated the purification of the product and led to a concomitant decrease in the yield. The reaction progress was monitored by injection of aliquots in HPLC-MS, which showed the formation of these secondary byproducts, as those derived from the double acylation of the starting molecule, and several decomposition products derived from the long reaction times at high temperatures. In this transformation, portionwise addition of the reagents over a period of several days was also required in order to obtain a moderate 52% yield. With the acetylated product **63** at hand, the diastereoselective bromocyclization was performed, and given the low stability of the product, attributed again to the presence of the labile *N*-acetyl group, different reaction conditions were tested (see the SI). Reaction conditions A and B depicted in Scheme 11 were developed in order to obtain either the *exo*-**62** or the *endo*-**62** products, respectively, as major compounds. As observed before, the addition of boron trifluoride etherate produced a higher proportion of the *endo*-**62** product, which was hypothesized to be the precursor of the correct structure of natural novofumigatamide. The low stability of products **62** and/or the starting material **63** led to complex reaction mixtures and impure products, even after separation and purification by column chromatography.

The remaining steps of the synthetic sequence were carried out separately with each diastereomer. First, the installation of the prenyl group on *endo*-**62** was achieved following the protocol described by Tokuyama and co-workers (using prenyltri*iso*propylsilane **31** as a nucleophile and AgNTf_2_ as silver salt), which seemed to be the most appropriate methodology to accomplish this transformation in complex polycyclic substrates.^34^ Although only a 31% yield was obtained in this transformation, the result is in agreement with the yields reported in the literature for such complex molecules.^34^ Our own experience corroborates this fact since reverse prenylation of comparable substrates with *endo* relative configurations does not occur when prenyltributylstannane **30** was used as the prenyl source, in spite of the highest nucleophilicity of this reagent (part II of this article; DOI: 10.1021/acs.joc.2c01228).(33,34,57) The subsequent hydrolysis of the methyl ester on *endo*-**49** following the method described by Nicolaou *et al*.^37^ turned out to be more challenging than expected. As observed with similar substrates during the development of this project (part II of this article; DOI: 10.1021/acs.joc.2c01228),^57^ the standard conditions for this reaction (5 equivalents of Me_3_SnOH and 60 °C of reaction temperature) did not provide any conversion to the product. The increase of the temperature to 80–90 °C and progressive addition of equivalents of trimethyltin hydroxide allowed us to observe the formation of the product, although the reaction rate dropped after addition of 20 equivalents of the reagent. To avoid the poisoning of the reaction mixture with the excess of the reagent, an intermediate workup was performed and the reaction was immediately set up again with the crude mixture. After repeating this process several times, the *endo*-**68** product could be isolated in 26% yield. The subsequent TFA-promoted *N*-Boc removal occurred with full conversion after 1 h of reaction time at 0 °C, but given the high polarity of *endo*-**69** and the inherent difficulties associated with its purification, the crude was immediately used in the next step after evaporation of the solvent.

Intramolecular macrolactam formation is a usually challenging transformation, which depends upon intrinsic structural features of the precursor substrate, including relative configuration, conformational preferences (presence of turn inducers, hydrogen bonds, etc.), and/or number of atoms of the macrocycle to be formed.^58−60^ Not all the preorganization of the acyclic substrate, determined by these conformational or configurational factors, assures the success of the cyclization process. It is also of pivotal importance to select the appropriate ring-closing reaction or, in the case of polypeptides, the location where the carboxylic acid and amine groups will be tethered. All these specific features of the reacting polypeptide make difficult to predict in advance the efficiency of the process. Most often, high-dilution conditions are used to favor the macrolactam formations versus intermolecular reactions leading to oligomeric products. One option to achieve low concentrations of the open-chain precursors is through a slow addition of the reagents with a syringe pump. Following a protocol of this type, slow addition of the HATU-activated acyclic precursor *endo*-**69** to a large volume of solvent containing the remaining reagents was performed.^61^ Nevertheless, although this protocol was successful for the preparation of more strained macrocycles studied in this research program (part II of this article; DOI: 10.1021/acs.joc.2c01228);^57^ in this case, it only led to a complex mixture of decomposition products.

The same synthetic sequence was also carried out with *exo*-**62**. Although the reverse prenylation gave access to the following intermediate of the synthetic route (*exo*-**49**) in a similar yield than that of the *endo*-**49** diastereoisomer, the hydrolysis of the methyl ester to provide *exo*-**68** proceeded more smoothly, since intermediate workups were not required and only half of the equivalents of Me_3_SnOH were required to achieve full conversion. Moreover, the product could be isolated in a satisfactory 75% yield. The different behavior shown by the *exo*- and *endo*-**49** products toward the hydrolysis demonstrates the great influence of the relative configuration of the hexahydropyrrolo[2,3-*b*]indole framework and the steric bulk in the proximity of the ester on this transformation, the *endo* diastereomer being less prone to reacting. The following *N*-Boc deprotection in the presence of TFA afforded the corresponding amino acid *exo*-**69**. The disappointing results obtained for the macrolactam formation of *endo*-**69**, likely due to long reaction times and/or the lack of reactivity of this substrate, encouraged us to accomplish the final ring-closing reaction in shorter reaction times, for both the addition with the syringe pump and the further stirring. Although the product was detected in trace amounts, the reaction crude showed a large number of decomposition byproducts.

#### Route B.4

**Scheme 12:**
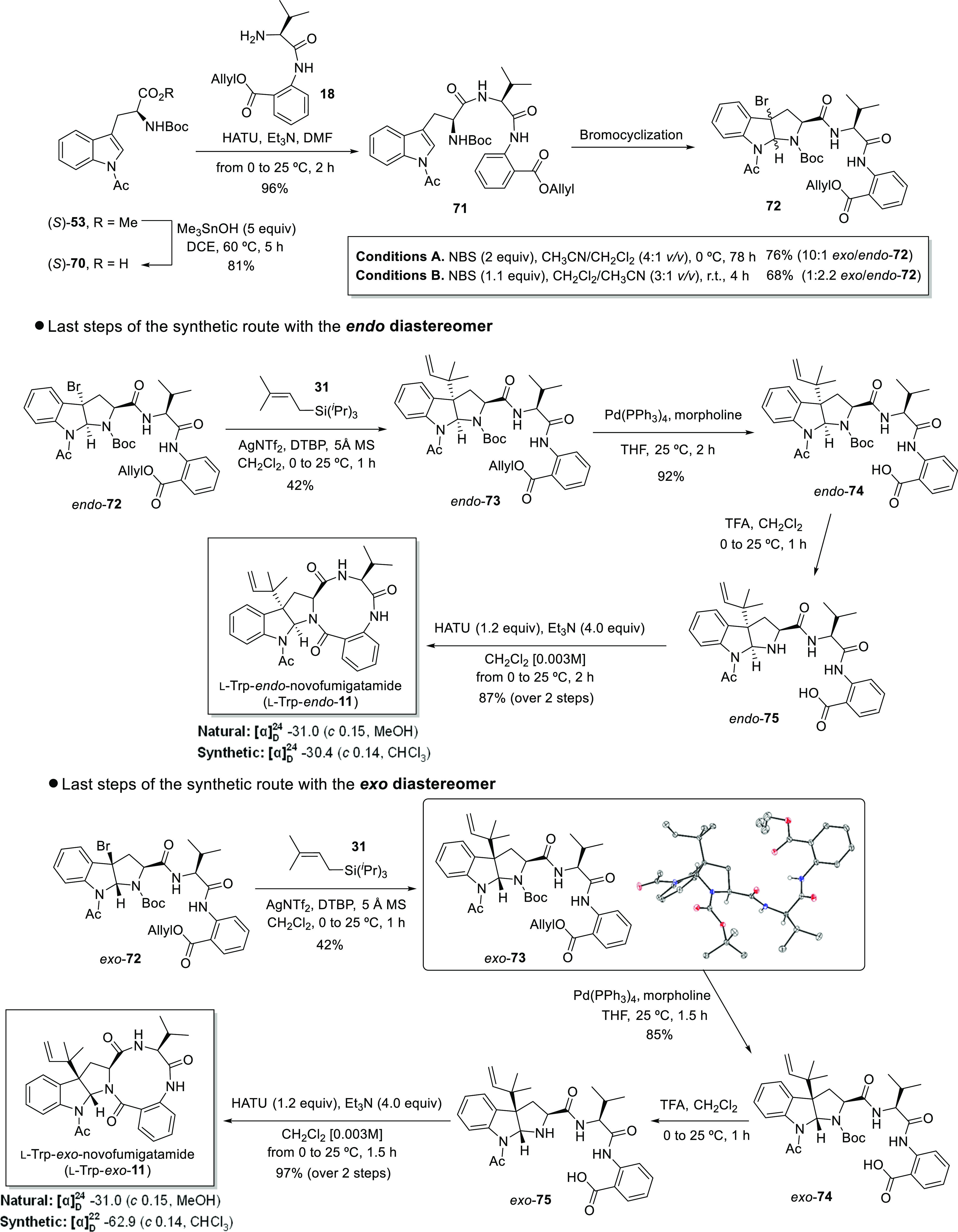
Synthetic Approach toward l-Trp-Novofumigatamide (l-Trp-**11**) Following Route B.4 The ORTEP diagram is represented with the ellipsoids drawn at 50% probability level.

The development of route B.3 was troublesome in several aspects, such as the purification and the stability of the intermediates, the lack of efficiency of the reactions, and the low yields achieved for the isolated products. Furthermore, the lack of reactivity of the final acyclic precursors toward the cyclization process was difficult to rationalize considering that the anthranilate residue is a turn-inducing element that might facilitate the proximity of both reaction sites.^62^ All these drawbacks made us abandon this route and focus our attention back to route B.1. Reconsidering the reasons of the failure of this route, we envisioned that the replacement of the *N*-Cbz group by a *N*-Boc protecting groups could be a simple and efficient way to shortcut the problems encountered during the removal of the *N*-Cbz under reducing conditions. In addition, the order of the last five steps was inverted with respect to route B.1, and this new alternative was named route B.4 (Scheme 12).

We embarked on the new synthetic route (Scheme 12) by first hydrolyzing the methyl ester of (*S*)-**53** under the standard conditions. The subsequent condensation of this fragment with the NH_2_-dipeptide **18** led to acyclic tripeptide **71** in good overall yield. To continue with route B.4, and unlike route B.1, we decided to postpone the deprotection of the carboxylic acid to a late stage of the synthesis since bromocyclizations with fully protected intermediates are easy to handle and usually result in higher yields. Throughout the study presented herein, the challenge to selectively produce *endo* diastereomers as major products or, at least, with a meaningful *endo*/*exo* ratio, has been underlined. The bromocyclization reaction has proven to be highly dependent on the structure of the starting substrate, and it is therefore difficult to make a prediction in advance. When acyclic precursor **71** was treated with 2 equivalents of NBS in a 4:1 *v/v* CH_3_CN/CH_2_Cl_2_ solvent mixture at 0 °C, the desired bromopyrrolidinoindoline **72** was isolated in good yield and with very good selectivity toward the *exo* isomer. The use of a 1:3 *v/v* CH_3_CN/CH_2_Cl_2_ solvent mixture at ambient temperature biased the reaction toward the *endo* product, but only with a moderate 1:2.2 *exo/ endo* diastereomeric ratio. For both sets of conditions, the use of mixture of solvents aided in the solubility of the starting material **71**, particularly at low temperatures. Surprisingly, when CH_3_CN was used as the major solvent, the addition of 2 equivalents of NBS and longer reaction times were required to reach full conversion. After separation of both diastereomers **72** by column chromatography, the remaining steps of route B.4 were carried out independently with each diastereomer. When both diastereomers **72** were subjected to reverse prenylation under the standard conditions, the corresponding alkylated products **73** were obtained in a significant 42% yield, which is higher than the yields observed previously for similar substrates. We were able to obtain suitable crystals for X-ray diffraction analysis, which unequivocally confirmed the identity of *exo*-**73** (Scheme 12). The ensuing *O*-allyl deprotection was accomplished in the presence of Pd(PPh_3_)_4_ and morpholine, which led to the corresponding products **74** in excellent yields in both cases. Then, the subsequent deprotection of the *N*-Boc group proceeded smoothly for both diastereoisomers in the presence of TFA. As in route B.3, the crudes of the previous deprotection reactions were immediately used in the final step of the synthesis to avoid problems related with the purification of such polar intermediates and the concomitant loss of yield. As demonstrated in route B.1, this sort of intermediates should cyclize readily without the need of slow reagent additions or more diluted reaction conditions. The same protocol applied to this route led to the formation of novofumigatamide diastereoisomers l-Trp-*endo*-**11** and l-Trp-*exo*-**11** in excellent yields. Unfortunately, the NMR spectroscopic data of the synthesized products were again inconsistent with those reported in the literature for the natural compound. The presence of rotamers in the ^1^H NMR spectra of both diastereomers recorded at 298 K, also observed for diastereomers d-Trp-**11**, seems to indicate that the correct structure of this natural product is a more constrained molecule or, alternatively, a putative structure with a preferred conformation fixed through intramolecular H-bonding interactions. Some NMR experiments conducted in CDCl_3_ at variable temperatures with the aim of confirming the latter hypothesis were unfruitful. Surprisingly, the ^1^H NMR data for l-Trp-*exo*-**11** is almost identical to the corresponding spectrum for d-Trp-*exo*-**11**. Likewise, the key signals for H11 and H18 in the *N*-Boc-*endo*-**38** analogue are similar to the signals for the equivalent protons in l-Trp-*endo*-**11**. Remarkably, the sign of the specific optical rotation for both l-Trp-novofumigatamide (**11**) synthetic analogues matched those reported for the natural product, which may be confirming the stereochemical origin of the natural product on l-tryptophan.

## Conclusions

**Figure 3:**
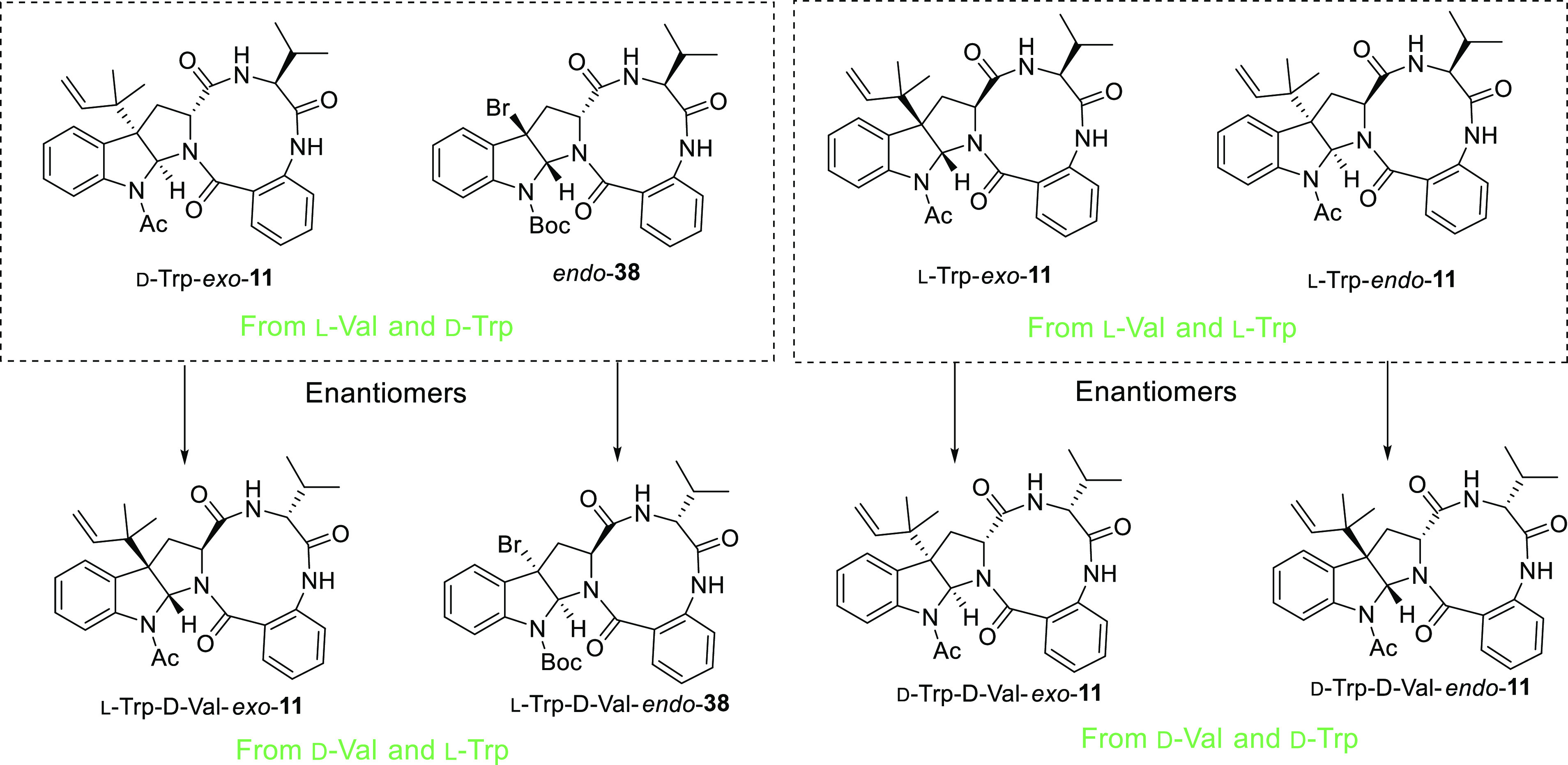
Synthetic diastereomers of (−)-novofumigatamide (**11**) and bromo-*N*-Boc-*N*-deacetylnovofumigatamide precursor (*endo*-**38**) prepared in this work, and their enantiomers.

In view of the results presented herein, a deep structural revision of the molecular skeleton of (−)-novofumigatamide (**11**) is required. Considering the *cis*-fusion on hexahydropyrrolo[2,3-*b*]indole frameworks as the only relative configuration allowed, eight stereoisomeric products (four diastereomers and their corresponding enantiomers) can be drawn if the connectivity between the atoms on the original structural proposal is maintained (Figure 3).^24^ During this work, we have been able to prepare three of these diastereomers (d-Trp-*exo*-**11,**l-Trp-*exo*-**11**, l-Trp-*endo*-**11**) and the bromo precursor of a *N*-Boc analogue derived from d-Trp, namely, *endo*-**38**, in which key signals for H11 and H18 would likely not differ from the corresponding signals of a putative prenylated and *N*-acetylated derivative (*vide supra*). None of the NMR data of the synthetic products matched those of the natural product, which led us to conclude that the correct structure of (−)-novofumigatamide must show a different connectivity between the atoms. Five synthetic routes, which differed in the final key steps used for the construction of the polycyclic skeleton of the natural product, were studied. In route A.1, a macrolactam formation followed by a diastereoselective bromocyclization was selected as last steps of the synthesis and allowed assembly of the proposed structure of the natural product. Following the same synthetic protocol, the *N*-Boc-*N*-deacetyl-*exo* analogue (**39**) and an *endo*-bromo precursor (*endo*-**38**) were also prepared. Unexpectedly, the same route or a slightly modified version (route B.1) was not suitable for the preparation of *exo* and *endo* diastereomeric products arising from l-tryptophan since the reduced compounds **48** were obtained instead. In a last attempt to access the l-Trp-*exo*-**11** and l-Trp-*endo*-**11** diastereomers, the final macrolactam formation was achieved through the formation of the amide bond between the tryptophan and the anthranilic acid units (route B.4). The latter synthetic sequence turned out to be the most efficient among all the routes explored since it gave rise to stable intermediates and final products with high yields and satisfactory *exo*/*endo* selectivities.

## Supporting Information Available

General information, synthesis and characterization of substrates, optimization of reaction conditions, comparative tables of the spectroscopic data of the natural and the synthetic products, spectra collection and HPLC-MS traces of the compounds synthesized, X-ray crystallographic data and references (PDF)
