# Supplementary material for: Total Synthesis of the Proposed Structure of (−)-Novofumigatamide, Isomers Thereof, and Analogues. Part I
Source: J Org Chem. 2022 Sep 22;87(19):12510–27. doi: 10.1021/acs.joc.2c01227 (PMC9552234; doi:10.1021/acs.joc.2c01227)
Supplement: Supplementary file 1 — jo2c01227_si_001.pdf [file jo2c01227_si_001.pdf]

Supporting Information

Total Synthesis of the Proposed Structure of (-)-Novofumigatamide, Isomers Thereof and Analogues. Part I

Patricia García-Domínguez,* Paula Lorenzo, Rosana Álvarez, and Angel R. de Lera*

CINBIO, Universidade de Vigo, 36310 Vigo, Spain

Table of contents

1. General information S1

2. Synthesis and characterization of substrates. Optimization of reaction conditions S3

3. Comparative tables of the spectroscopic data of the natural and the synthetic products S48

4. Spectra collection and HPLC-MS traces S49

5. X-Ray structures S118

6. References S120

1. General information

$CHCl_3$ was used from commercial sources as analytical reagent-grade solvent or dried according to published methods and distilled before use if required. THF, CH_2Cl_2, DMF, CH_3CN and MeOH were dried using a solvent purification system (Puresolv^{TM}, Innovative Technology). Alternatively, Aldrich MeOH, DMF and CH_3CN 99.8% packaged under argon in a resealable Sure/Seal^{TM} bottle were used. DCE (1,2-dichloroethane) 99.5%, extra dry, kept over Molecular Sieves and packaged under argon in a resealable AcroSeal® was used. Et_3N and DIPEA were dried by distillation over CaH_2. Alternatively, Et_3N (≥ 99.5%) and DIPEA (99.5%) packaged under argon in a resealable Sure/Seal^{TM} bottle were purchased from Aldrich. Et_2NH was dried by distillation with CaH_2. All other reagents were commercial compounds of the highest purity available, which were used as received unless otherwise noted. All the reactions were carried out under argon, and those not involving aqueous reagents were carried out in oven-dried glassware. All solvents and anhydrous solutions were transferred through syringes and cannulae previously dried in an oven for at least 12 h and stored in a desiccator with KOH. For reactions at low temperatures, ice/water or CO_2/acetone systems were used. For different temperatures, a HaaKe EK90 Immersion Cooler apparatus (-78 to 0 °C) was used. Analytical TLC was performed on aluminium plates with Merck Kieselgel $60F_{254}$ and visualized by UV irradiation (254 nm) or by staining with an ethanolic solution of phosphomolybdic acid or an ethanolic solution of *p*-anisaldehyde. Flash-column chromatography was carried out on Merck Kieselgel 60 (230–400 mesh), Silicycle SiliaFlash® P60 (230-400 mesh) or C18-SiO_2 (Waters, 55–105 µm, 125Å) under pressure or using an automated Teledyne Isco CombiFlash® Rf+ system. HPLC was performed using a Waters 515 Pump, a Waters 600 Controller, and a Waters 2487 Dual λ Absorbance Detector. HPLC-MS system contains the following modules: Waters 2545 pump, Waters 2767 Sample Manager, Waters SFO System Fluidics Organizer, Waters 2898 Photodiode Array Detector, MICROMASS Quattro micro^{TM} API mass spectrometer and Edwards 28 Vacuum Pump.

^{1}H NMR and ^{13}C{^{1}H} NMR spectra were recorded at room temperature or at the indicated temperature (323 K, 328 K, 343 K, 383 K) on a Bruker AVANCE 400 spectrometer operating at 400 and 101 MHz, respectively, with residual protic solvent as the internal reference ($CDCl_3$, δ_H = 7.26 and δ_C = 77.2 ppm; DMSO-d_6, δ_H = 2.5 and δ_C = 39.52 ppm; CD_3OD, δ_H = 3.31 ppm); chemical shifts (δ) are given in parts per million (ppm) and coupling constants (*J*) are given in Hertz (Hz). The proton spectra are reported as follows: δ (multiplicity, coupling constant *J*, number of protons, assignment). The DEPT135 pulse sequence and the standard 2D experiments (HSQC, HMBC, COSY) were used to aid in the assignment of signals in the ^{1}H NMR spectra. HRMS (ESI-TOF) were measured with a Bruker SolariX XR mass spectrometer equipped with a 7T actively shielded magnet. Ions were generated using an Apollo API electrospray ionization

(ESI) source, with a voltage between 1800 and 2200 V (to optimize ionization efficiency) applied to the needle, and a counter voltage of 450 V applied to the capillary. ESI spectra samples were prepared by adding a spray solution of 70:29.9:0.1 (*v/v/v*) MeOH/water/formic acid to a solution of the sample at a *v/v* ratio of 1 to 5% to give the best signal-to-noise ratio. IR spectra were recorded with a JASCO FT/IR-4200 spectrophotometer from a thin film deposited onto NaCl glass or with an ATR module. Specific optical rotations were measured on a JASCO P-1020 or a JASCO P-2000 polarimeters with a Na lamp (glass cell, 3.5 x 100 mm). Melting points were measured in a Stuart Scientific apparatus.

2. Synthesis and characterization of substrates. Optimization of reaction conditions

Optimization of the synthetic route A.1 with the *N*-Boc protecting group

Allyl (*S*)-2-[2'-({[(9*H*-Fluoren-9^{iv}-yl)methoxy]carbonyl}amino)-3'-methylbutanamido]benzoate 26

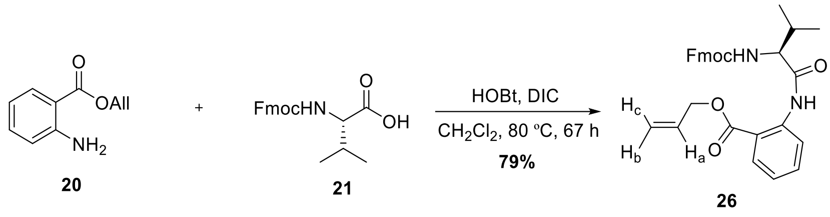

General procedure for amide (peptide) bond formation. Method A. In a Schlenk flask, DIC (0.78 g, 6.21 mmol) was added to a solution of allyl anthranilate **20** (0.89 mL, 1.0 g, 5.64 mmol), *N*-Fmoc protected valine **21** (2.11 g, 6.21 mmol) and HOBt (0.84 g, 6.21 mmol) in CH_2Cl_2 (18.8 mL). The flask was sealed, and the reaction mixture was stirred in a metal heating block at 80 ºC for 67 h. The solution was allowed to reach room temperature and the solvent was evaporated. The residue was purified by trituration with MeOH (3 x 15 mL) to afford 2.23 g (79% yield) of the titled compound as a white solid. **M.p.**: 171-173 ºC (toluene). **^{1}H NMR** (400 MHz, $CDCl_3$) δ 8.73 (dd, J = 8.5, 1.1 Hz, 1H, ArH), 8.08 (dd, J = 8.0, 1.6 Hz, 1H, ArH), 7.77 (d, J = 7.5 Hz, 2H, ArH), 7.67 (d, J = 7.3 Hz, 1H, ArH), 7.64 (d, J = 7.5 Hz, 1H, ArH), 7.57 (t, J = 7.8 Hz, 1H, ArH), 7.44 – 7.35 (m, 2H, ArH), 7.37 – 7.27 (m, 2H, ArH), 7.12 (t, J = 7.6 Hz, 1H, ArH), 5.97 (ddt, J_{ac} = 17.2, J_{ab} =10.4, $J_{a\text{-}CH2O}$ = 5.7 Hz, 1H, $OCH_2C\underline{H}_a{=}CH_2$), 5.48 (d, J = 8.5 Hz, NH), 5.36 (app. dq, J_{ca} = 17.2, J_{cb} = 1.5 Hz, 1H, $OCH_2CH{=}C\underline{H}_cH$), 5.27 (dd, J_{ba} = 10.4, J_{bc} =1.5 Hz, 1H, $OCH_2CH{=}C\underline{H}_bH$), 4.74 (d, $J_{CH2O\text{-}Ha}$ = 5.1 Hz, 2H, $O\underline{CH_2}CH{=}CH_2$), 4.54 – 4.19 (m, 4H, $OC\underline{H_2}C\underline{H}Ar_2$ + $H_{2'}$), 2.42 – 2.28 (m, 1H, $C\underline{H}(CH_3)_2$), 1.07 (d, J = 6.8 Hz, 3H, $CH(C\underline{H_3})_2$), 1.00 (d, J = 6.8 Hz, 3H, $CH(C\underline{H_3})_2$) ppm. **^{13}C{^{1}H} NMR** (101 MHz, $CDCl_3$) δ 170.5 (s), 167.9 (s), 156.6 (s), 144.2 (s), 143.9 (s), 141.4 (s, 2x), 141.1 (s), 134.8 (d), 131.7 (d), 131.0 (d), 127.8 (d, 2x), 127.2 (d, 2x), 125.4 (d), 125.3 (d), 123.1 (d), 120.6 (d), 120.1 (d, 2x), 118.9 (t), 115.5 (s), 67.4 (t), 66.0 (t), 61.7 (d), 47.4 (d), 31.4 (d), 19.5 (q), 17.7 (q) ppm. **IR** (neat): ν 3330 (w, N-H), 3282 (w, N-H), 3042 (w, C-H), 2962 (w, C-H), 2871 (w, C-H), 1718 (m, C=O), 1666 (s, C=O), 1526 (s), 1449 (s), 1287 (s), 1249 (s) cm^{-1}. **HRMS** (ESI-TOF) *m/z*: $[M + H]^+$ calcd. for $C_{30}H_{31}N_2O_5$, 499.2228; found, 499.2222. $[\alpha]_D^{21}$ –36 (c 0.17, DMSO).

Allyl (*S*)-2-(2'-Amino-3'-methylbutanamido)benzoate 18

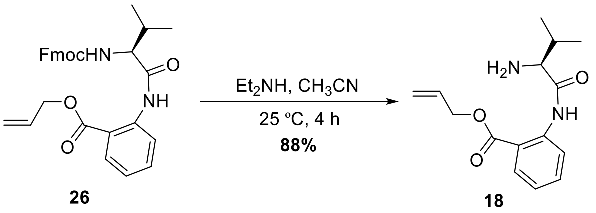

To a solution of *N*-Fmoc protected dipeptide **26** (3.84 g, 7.70 mmol) in CH_3CN (154 mL) Et_2NH was added dropwise (15.92 mL, 11.26 g, 154.0 mmol) and the reaction mixture was stirred for 16 h at room temperature. The solvent was removed under reduced pressure and the residue was purified by flash column chromatography (CombiFlash® Rf+ system, 80 g silica gel, gradient from 90:10 to 60:40 *v/v* hexane/EtOAc, flow rate = 60 mL/min) to afford 1.87 g (88% yield) of the titled compound as a colourless oil. **^{1}H NMR** (400 MHz, $CDCl_3$) δ 8.80 (dd, *J* = 8.5, 1.0 Hz, 1H, ArH), 8.09 (dd, *J* = 8.0, 1.7 Hz, 1H, ArH), 7.55 (td, *J* = 8.2, 1.7 Hz, 1H, ArH), 7.14 – 7.07 (m, 1H, ArH), 6.17 – 5.93 (m, 1H, $OCH_2C\underline{H}{=}CH_2$), 5.47 – 5.38 (m, 1H, $OCH_2CH{=}C\underline{H_2}$), 5.35 – 5.29 (m, 1H, $OCH_2CH{=}C\underline{H_2}$), 4.91 – 4.73 (m, 2H, $OC\underline{H_2}CH{=}CH_2$), 3.43 (d, *J* = 4.3 Hz, 1H, $H_{2'}$), 2.46 – 2.28 (m, 1 H, $C\underline{H}(CH_3)_2$), 1.06 (d, *J* = 6.9 Hz, 3H, $CH(C\underline{H_3})_2$), 0.92 (d, *J* = 6.9 Hz, $CH(C\underline{H_3})_2$) ppm. **^{13}C{^{1}H} NMR** (101 MHz, $CDCl_3$) δ 174.1 (s), 167.1 (s), 140.9 (s), 134.3 (d), 131.8 (d), 130.8 (d), 122.4 (d), 120.4 (d), 118.4 (t), 115.6 (s), 65.6 (t), 61.6 (d), 31.4 (d), 19.6 (q), 16.3 (q) ppm. **IR** (NaCl): ν 3399 (w, N-H), 3300 – 3200 (br, N-H), 2960 (m, C-H), 2933 (m, C-H), 2873 (m, C-H), 1691 (s, C=O), 1582 (s), 1514 (s), 1447 (s), 1364 (m), 1296 (s), 1254 (s), 1164 (m), 1143 (m), 1085 (s) cm^{-1}. **HRMS** (ESI-TOF) *m/z*: $[M + H]^+$ calcd. for $C_{15}H_{21}N_2O_3$, 277.1547; found, 277.1537. $[\alpha]_D^{24}$ –29 (*c* 1.45, $CHCl_3$).

(*R*)-2-Phenylmethoxycarbonylamino-3-(indole-3'-yl)propanoic Acid (*R*)-23

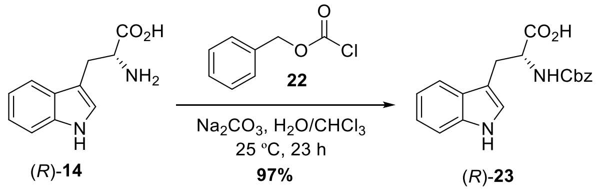

A solution of Na_2CO_3 (1.06 g, 9.79 mmol) in water (5.7 mL) was added to a suspension of D-tryptophan (*R*)-**14** (2.0 g, 9.79 mmol) in $CHCl_3$ (98 mL) at 25 ºC. The resulting biphasic mixture was then cooled down to 0 ºC, and benzyl chloroformate (1.4 mL, 1.67 g, 9.79 mmol) and Na_2CO_3 (5 mL, 2.0 M in H_2O) were sequentially added. After 23 h of vigorous stirring at 25 ºC, the reaction mixture was diluted with water, the organic layer was separated, and the aqueous layer was washed with $CHCl_3$ (3x). The aqueous layer was treated with a 10% HCl aqueous solution to pH 5-6 to give a white solid that was filtered and washed with diethyl ether to afford 3.2 g (97% yield) of the titled compound. Alternatively, the acidified aqueous solution could be extracted

with EtOAc (3x) and the combined organic layers dried over anhydrous Na_2SO_4 and concentrated under reduced pressure. The spectroscopic data matched those previously reported.[1-2]

^{1}H-NMR (400 MHz, CD_3OD) δ 7.56 (d, J = 8.0 Hz, 1H, ArH), 7.38 – 7.20 (m, 6H, ArH), 7.12 – 7.04 (m, 2H, ArH), 6.99 (t, J = 7.4 Hz, 1H, ArH), 5.10 – 4.97 (m, 2H, C<u>H</u>$_2$Ph), 4.55 – 4.46 (m, 1H, H_2), 3.37 – 3.32 (m, 1H, H_{3A}), 3.15 (dd, J = 14.7, 8.1 Hz, 1H, H_{3B}) ppm. $[\alpha]_D^{22}$ +7 (c 0.59, MeOH).

Methyl (*R*)-2-Phenylmethoxycarbonylamino-3-(indole-3'-yl)propanoate (*R*)-24

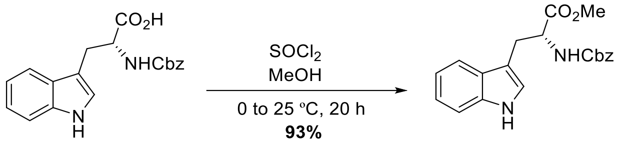

(*R*)-**23** (*R*)-**24**

Thionyl chloride (0.52 mL, 0.84 g, 7.09 mmol) was added to a solution of *N*-Cbz-protected D-tryptophan (*R*)-**23** (2.0 g, 5.91 mmol) in MeOH (74 mL) at 0 ºC and the resulting mixture was stirred for about 15 min. It was then allowed to warm at room temperature and stirred overnight at 25 ºC. The solution was concentrated under reduced pressure and diluted with $CHCl_3$ (32 mL). A 10% $NaHCO_3$ aqueous solution was added, and the mixture was transferred to a separation funnel. If the pH of the resulting aqueous phase was 8, it was extracted with $CHCl_3$ (3x). If this was not the case, more 10% aqueous $NaHCO_3$ was added before the extraction. The combined organic layers were dried over anhydrous Na_2SO_4 and concentrated under reduced pressure to afford 1.94 g (93% yield) of the title compound as a slightly yellow foam that was used in the next step without further purification. Alternatively, the residue could be purified by flash column chromatography (CombiFlash® Rf+ system, 80 g silica gel, gradient from 80:20 to 60:40 *v/v* hexane/EtOAc, flow rate = 60 mL/min) with the concomitant decrease in the yield. The spectroscopic data matched those previously reported.[2-3]

^{1}H-NMR (400 MHz, $CDCl_3$) δ 8.06 (s, 1H, NH), 7.52 (d, J = 7.9 Hz, 1H, ArH), 7.41 - 7.27 (m, 6H, ArH), 7.19 (t, J = 7.6 Hz, 1H, ArH), 7.10 (t, J = 7.5 Hz, 1H, ArH), 6.96 (d, J = 2.3 Hz, 1H, ArH), 5.37-5.24 (m, N<u>H</u>Cbz), 5.19 – 4.99 (m, 2H, C<u>H</u>$_2$Ph), 4.77 - 4.68 (m, 1H, H_2), 3.68 (s, 3H, CO_2C<u>H$_3$</u>), 3.41-3.20 (m, 2H, 2H_3) ppm. $[\alpha]_D^{26}$ -30 (c 1.76, $CHCl_3$).

Methyl (*R*)-3-[1'-(*tert*-Butoxycarbonyl)indole-3'-yl]-2-(phenylmethoxycarbonylamino)propanoate (*R*)-32

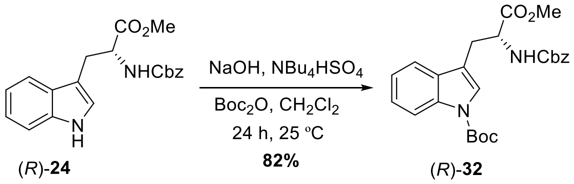

(*R*)-**24** (*R*)-**32**

Powdered sodium hydroxide (1.24 g, 30.9 mmol) was added to a solution of the methyl ester of Cbz-protected D-tryptophan derivative (*R*)-**24** (3.63 g, 10.3 mmol) and tetrabutylammonium hydrogen sulfate (0.18 g, 0.52 mmol) in CH_2Cl_2 (103 mL). The mixture was stirred for 30 min at 25 ºC. Di-*tert*-butyldicarbonate (3.37 g, 15.45 mmol) was then added and the mixture was stirred for 24 h. It was filtered through a pad of Celite® and the filtrate was evaporated *in vacuo*. The residue was purified by flash column chromatography (CombiFlash® Rf+ system, 120 g silica gel, gradient from 90:10 to 60:40 *v/v* hexane/EtOAc, flow rate = 85 mL/min) to afford 3.8 g (82% yield) of the titled compound as a colourless foam.The spectroscopic data matched those previously reported.[4]

H-NMR (400.16 MHz, $CDCl_3$) δ 8.11 (d, *J* = 7.3 Hz, 1H, ArH), 7.47 (d, *J* = 7.8 Hz, 1H, ArH), 7.42 – 7.29 (m, 7H, ArH), 7.24 – 7.17 (m, 1H, ArH), 5.36 (d, *J* = 7.8 Hz, 1H, N<u>H</u>Cbz), 5.17 – 5.06 (m, 2H, C<u>H</u>$_2$Ph), 4.78 – 4.69 (m, 1H, H_2), 3.69 (s, 3H, CO_2C<u>H</u>$_3$), 3.31 – 3.18 (m, 2H, $2H_3$), 1.66 (s, 9H, $CO_2{}^t$<u>Bu</u>) ppm. $[\alpha]_D^{23}$ -36 (*c* 1.22, $CHCl_3$).

(*R*)-3-[1’-(*tert*-Butoxycarbonyl)indole-3’-yl]-2-(phenylmethoxycarbonylamino)propanoic Acid (*R*)-33

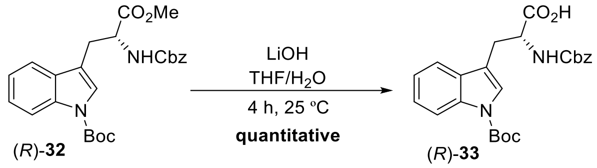

General procedure for the hydrolysis of esters. Method A. The tryptophan derivative (*R*)-**32** (3.8 g, 8.4 mmol) was dissolved in a THF/H_2O mixture (168 mL, 1:1 *v/v*) and the resulting mixture was cooled down to 0 ºC. LiOH (3.02 g, 126.0 mmol) was added, and the reaction was allowed to warm to room temperature and stirred for 4 h. The reaction was acidified with a 1 M aqueous HCl solution and extracted with EtOAc (3x). The combined organic layers were washed with a saturated aqueous solution of NaCl, dried over anhydrous Na_2SO_4 and concentrated *in vacuo*. The residue was purified by flash column chromatography (CombiFlash® Rf+ system, 120 g silica gel, gradient from 95:5 to 90:10 *v/v* CH_2Cl_2/MeOH, flow rate = 85 mL/min) to obtain 3.68 g (quantitative yield) of the titled compound as a white solid. **M.p.**: > 250 ºC dec. (hexane/CH_2Cl_2/MeOH). **^{1}H NMR** (400 MHz, DMSO-d_6, 343 K) δ 8.02 (d, *J* = 8.2 Hz, 1H, ArH), 7.62 (d, *J* = 7.8 Hz, 1H, ArH), 7.50 (s, 1H, ArH), 7.38 – 7.11 (m, 7H, ArH), 5.00 (d, *J* = 12.8 Hz, 1H, OC<u>H</u>$_2$Ph), 4.95 (d, *J* = 12.8 Hz, 1H, OC<u>H</u>$_2$Ph), 4.26 (app. td, *J* = 8.0, 4.7 Hz, 1H, H_2), 3.25 (dd, *J* = 14.5, 4.6 Hz, 1H, H_{3A}), 3.02 (dd, *J* = 14.8, 8.3 Hz, 1H, H_{3B}), 1.62 (s, 9H, CO_2tBu) ppm. **^{13}C{^{1}H} NMR** (101 MHz, DMSO-d_6, 343 K) δ 173.9 (s), 155.3 (s), 148.8 (s), 136.8 (s), 134.4 (s), 130.4 (s), 127.8 (d, 3x), 127.2 (d), 127.0 (d), 123.6 (d), 123.4 (d), 121.9 (d), 118.9 (d), 117.2 (s), 114.2 (d), 83.0 (s), 64.9 (t), 54.8 (d), 27.4 (q, 3x), 27.1 (t) ppm. **IR** (NaCl): ν 3500 -3200 (br,

COO-H), 2976 (w, C-H), 2926 (w, C-H), 2854 (w, C-H), 1728 (s, C=O), 1585 (s), 1452 (s), 1372 (s), 1257 (s), 1159 (s), 1089 (m) cm^{-1}. **HRMS** (ESI-TOF) *m/z*: $[M + H]^+$ calcd. for $C_{24}H_{27}N_2O_6$, 439.1864; found, 439.1860. $[\alpha]_D^{19}$ –5.9 (*c* 0.2, $CHCl_3$).

Allyl (2'*S*,2''*R*)-2-(2'-{3''-[1'''-(*tert*-Butoxycarbonyl)indole-3'''-yl]-2''-(phenylmethoxycarbonylamino)propanamido}-3'-methylbutanamido)benzoate 34

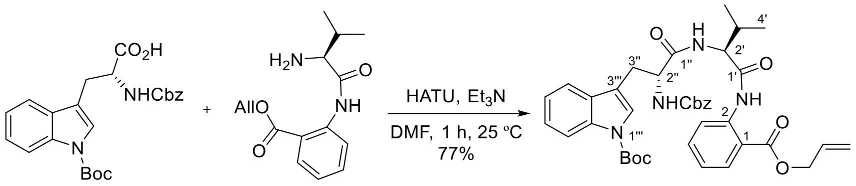

(*R*)-**33** **18** **34**

General procedure for amide (peptide) bond formation. Method B. To a cooled (0 ºC) solution of the indole derivative (*R*)-**33** (0.21 g, 0.47 mmol) and dipeptide **18** (0.13 g, 0.47 mmol) in DMF (6.4 mL) were added Et_3N (0.1 mL, 0.07 g, 0.71 mmol) and HATU (0.27 g, 0.71 mmol) and the mixture was stirred for 1 h at 25 ºC. Water (10 mL) was added to the reaction mixture, the layers were separated, and the aqueous layer was extracted with EtOAc (3x). The combined organic layers were washed with a saturated aqueous solution of NaCl (3x), dried over anhydrous Na_2SO_4 and the solvent was evaporated. The residue was purified by flash column chromatography (silica gel, 80:20 *v/v* hexane/EtOAc) to afford 0.26 g (77% yield) of the titled compound as a white solid. **^{1}H NMR** (400 MHz, DMSO-d_6, 343 K) δ 10.78 (s, 1H, CONH), 8.38 (dd, *J* = 8.4, 1.2 Hz, 1H, ArH), 8.22 (d, *J* = 7.9 Hz, 1H, NH), 8.04 (dt, *J* = 8.3, 0.9 Hz, 1H, ArH), 7.97 (dd, *J* = 7.9, 1.5 Hz, 1H, ArH), 7.70 (ddd, *J* = 7.8, 1.3, 0.7 Hz, 1H, ArH), 7.61 (ddd, *J* = 8.7, 7.3, 1.7 Hz, 1H, ArH), 7.55 (s, 1H, ArH), 7.37 – 7.15 (m, 8H, ArH), 6.03 (ddt, *J* = 17.2, 10.5, 5.6 Hz, 1H, $OCH_2C\underline{H}=CH_2$), 5.38 (app. dq, *J* = 17.2, 1.6 Hz, 1H, $OCH_2CH=C\underline{H}_{trans}H$), 5.27 (app. dq, *J* = 10.5, 1.4 Hz, 1H, $OCH_2CH=C\underline{H}_{cis}H$), 5.05 – 4.90 (m, 2H, $OC\underline{H_2}Ph$), 4.85 – 4.74 (m, 2H, $OC\underline{H_2}CH=CH_2$), 4.65 (td, *J* = 8.5, 5.7 Hz, 1H, $H_{2''}$), 4.21 (dd, *J* = 8.0, 6.2 Hz, 1H, $H_{2'}$), 3.16 (dd, *J* = 14.7, 5.3 Hz, 1H, $H_{3A''}$), 3.04 (dd, *J* = 14.7, 8.7 Hz, 1H, $H_{3B''}$), 2.27 – 2.10 (m, 1H, $C\underline{H}(CH_3)_2$), 1.62 (s, 9H, CO_2tBu), 0.89 (d, *J* = 6.8 Hz, 3H, $CH(C\underline{H_3})_2$), 0.88 (d, *J* = 6.8 Hz, 3H, $CH(C\underline{H_3})_2$) ppm. **^{13}C{^{1}H} NMR** (101 MHz, DMSO-d_6, 343 K) δ 171.4 (s), 169.5 (s), 166.2 (s), 155.2 (s), 148.7 (s), 139.2 (s), 136.6 (s), 134.5 (s), 133.6 (d), 131.9 (d), 130.1 (d), 130.0 (s), 127.8 (d), 127.2 (d), 126.9 (d), 123.8 (d), 123.7 (d), 122.9 (d), 121.9 (d), 120.6 (d), 119.0 (d), 117.9 (t), 117.0 (s), 116.1 (s), 114.2 (d), 83.1 (s), 65.1 (t, 2x), 59.6 (d), 54.5 (d), 29.4 (d), 27.5 (t), 27.4 (q, 3x), 18.7 (q), 17.5 (q) ppm. **IR** (neat): ν 3500 - 3200 (br, N-H), 2970 (w, C-H), 2934 (w, C-H), 1729 (s, C=O), 1689 (s, C=O), 1589 (m), 1526 (s), 1451 (s), 1371 (s), 1258 (s), 1160 (s), 1087 (s) cm^{-1}. **HRMS** (ESI-TOF) *m/z*: $[M + H]^+$ calcd. for $C_{39}H_{45}N_4O_8$, 697.3232; found, 697.3212. $[\alpha]_D^{24}$ +7 (*c* 0.46, $CHCl_3$).

(2'*S*,2''*R*)-2-(2'-{3''-[1'''-(*tert*-Butoxycarbonyl)indole-3'''-yl]-2''-(phenylmethoxycarbonylamino)propanamido}-3'-methylbutanamido)benzoic Acid 35

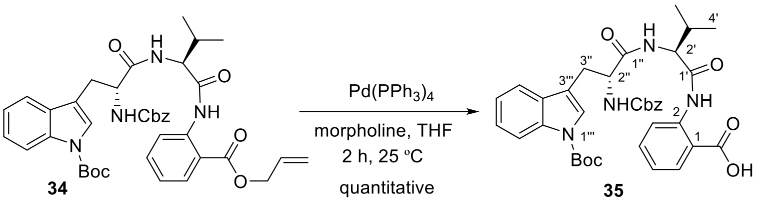

General procedure for the deprotection of allyl esters. To a stirred solution of the allyl ester **34** (1.58 g, 2.27 mmol) and $Pd(PPh_3)_4$ (0.26 g, 0.23 mmol) in THF (119 mL), morpholine (0.4 mL, 0.4 g, 4.54 mmol) was added dropwise and the resulting mixture was stirred for 2 h at room temperature. The reaction mixture was diluted with EtOAc, and the organic layer was washed with a 1M aqueous HCl solution (until acid pH of the resulting aqueous phase is reached) and a saturated aqueous solution of NaCl (2x), then dried over anhydrous Na_2SO_4 and the solvent evaporated. The residue was purified by flash column chromatography (CombiFlash® Rf+ system, 80 g silica gel, gradient from 97.5:2.5 to 90:10 *v/v* CH_2Cl_2/MeOH, flow rate = 60 mL/min) to afford 1.49 g (quantitative yield) of the titled compound as a yellow solid. **^{1}H NMR** (400 MHz, DMSO-d_6, 343 K) δ 8.52 – 8.46 (m, 1H, ArH), 8.13 (d, *J* = 8.4 Hz, 1H, NH), 8.07 – 7.97 (m, 2H, ArH), 7.80 – 7.69 (m, 1H, ArH), 7.67 – 7.42 (m, 1H, ArH), 7.42 – 7.12 (m, 8H, ArH), 7.06 – 6.98 (m, 1H, ArH), 5.02 – 4.89 (m, 2H, OC<u>H</u>$_2$Ph), 4.64 – 4.51 (m, 1H, $H_{2''}$), 4.28 – 4.17 (m, 1H, $H_{2'}$), 3.18 (dd, *J* = 14.7, 5.1 Hz, 1H, $H_{3A''}$), 3.00 (dd, *J* = 14.7, 9.2 Hz, 1H, $H_{3B''}$), 2.23 – 2.07 (m, 1H, C<u>H</u>$(CH_3)_2$), 1.62 (s, 9H, CO_2*t*Bu), 0.88 (d, *J* = 6.9 Hz, 3H, CH(C<u>H</u>$_3$)$_2$), 0.85 (d, *J* = 6.8 Hz, 3H, CH(C<u>H</u>$_3$)$_2$) ppm. **^{13}C{^{1}H} NMR** (101 MHz, DMSO-d_6, 343 K) δ 171.1 (s), 170.4 (s), 169.0 (s), 155.6 (s), 148.8 (s), 140.0 (s), 136.7 (s), 134.5 (s), 131.0 (d), 130.8 (d), 130.2 (s), 127.9 (d, 3x), 127.3 (d), 127.0 (d), 123.9 (d, 2x), 122.1 (d), 121.6 (d), 119.3 (d), 118.5 (d), 116.5 (s), 114.3 (d), 83.2 (s), 65.2 (t), 59.6 (d), 54.7 (d), 30.2 (d), 27.5 (q, 3x), 27.5 (t), 18.9 (q), 17.8 (q) ppm. **IR** (NaCl): ν 3500 – 3200 (br, COO-H), 3061 (w, C-H), 2971 (w, C-H), 2933 (w, C-H), 1731 (s, C=O), 1688 (s, C=O), 1657 (s, C=O), 1589 (s), 1512 (s), 1451 (s), 1387 (s), 1257 (m), 1158 (m), 1089 (m) cm^{-1}. **HRMS** (ESI-TOF) *m/z*: $[M + H]^+$ calcd. for $C_{36}H_{41}N_4O_8$, 657.2919; found, 657.2898. $[\alpha]_D^{21}$ –17.3 (*c* 0.27, $CHCl_3$).

REMARK: one ^{13}C{^{1}H} NMR signal singlet is missing.

(2'*S*,2''*R*)-2-(2'-{-2''-Amino-3''-[1'''-(*tert*-butoxycarbonyl)indole-3'''-yl]propanamido}-3'-methylbutanamido)benzoic Acid 36

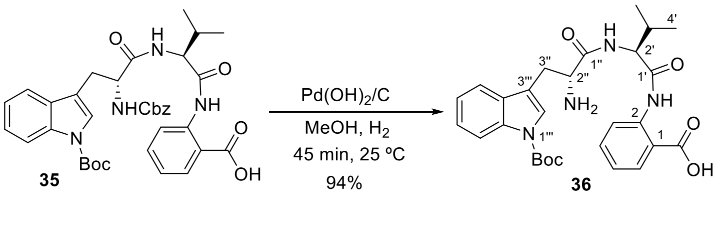

General procedure for Cbz deprotection. $Pd(OH)_2/C$ (0.96 g, 6.85 mmol) was added to a solution of Cbz-protected amine **35** (1.54 g, 2.34 mmol) in MeOH (236 mL) under argon atmosphere. After the resulting mixture was purged with H_2 gas, it was stirred for 4 h. The reaction mixture was filtered over Celite® and the solvent was evaporated. The yellow solid obtained (1.15 g, 94% yield) was used in the next step without further purification. **^{1}H NMR** (400 MHz, DMSO-d_6, 343 K) δ 8.66 (br s, 1H, NH), 8.40 (d, J = 8.2 Hz, 1H), 8.05 (d, J = 8.2 Hz, 1H, ArH), 7.97 (d, J = 7.8 Hz, 1H, ArH), 7.69 (d, J = 7.8 Hz, 1H, ArH), 7.68 – 7.49 (m, 1H, ArH + O=PPh_3 residue), 7.38 – 7.18 (m, 3H, ArH), 6.98 (t, J = 7.5 Hz, 1H, ArH), 4.46 (t, J = 7.5 Hz, 1H, $H_{2''}$), 4.15 – 4.09 (m, 1H, $H_{2'}$), 3.28 – 3.15 (m, 2H, $2H_{3''}$), 2.39 – 2.25 (m, 1H, $C\underline{H}(CH_3)_2$), 1.63 (s, 9H, CO_2tBu), 0.74 (d, J = 6.8 Hz, 3H, $CH(C\underline{H}_3)_2$), 0.55 (d, J = 6.6 Hz, 3H, $CH(C\underline{H}_3)_2$) ppm. **^{13}C{^{1}H} NMR** (101 MHz, DMSO-d_6, 343 K) δ 169.6 (s), 169.0 (s), 168.4 (s), 148.6 (s), 139.2 (s), 134.6 (s), 130.8 (d), 129.8 (d), 129.7 (s), 124.5 (s), 124.0 (d, 2x), 122.0 (d), 121.4 (d), 118.9 (d), 118.3 (d), 114.3 (s), 114.2 (d), 83.2 (s), 58.9 (d), 52.3 (d), 27.9 (d), 27.4 (q, 3x), 26.0 (t), 18.4 (q), 16.4 (q) ppm. **IR** (neat): ν 3300-3000 (br, O-H, N-H), 2965 (w, C-H), 2930 (w, C-H), 1732 (m, C=O), 1656 (m, C=O), 1587 (m), 1506 (m), 1446 (m), 1367 (s), 1304 (m), 1256 (m), 1155 (m), 1085 (m), 1043 (w), 1019 (w), 934 (w), 833 (w), 749 (s), 698 (w), 665 (w) cm^{-1}. **HRMS** (ESI-TOF) *m/z*: $[M + H]^+$ calcd. for $C_{28}H_{35}N_4O_6$, 523.2551; found, 523.2534. $[\alpha]_D^{24}$ –97.6 (*c* 0.11, MeOH).

REMARK: this compound is contaminated with trace amounts of triphenylphosphine oxide, a by-product arising from the deprotection of the allyl ester with $Pd(PPh_3)_4$ (a residue from the previous step of the synthetic route). This was corroborated by the presence of a peak at δ 29.35 ppm in the ^{31}P NMR spectrum and accounts for the excess of signals in the ^{1}H NMR and ^{13}C{^{1}H} NMR spectra. The high polarity and insolubility of this compound precluded a further purification by column chromatography.

Bromo-precursor of the Boc analogue of the synthetic novofumigatamide 38

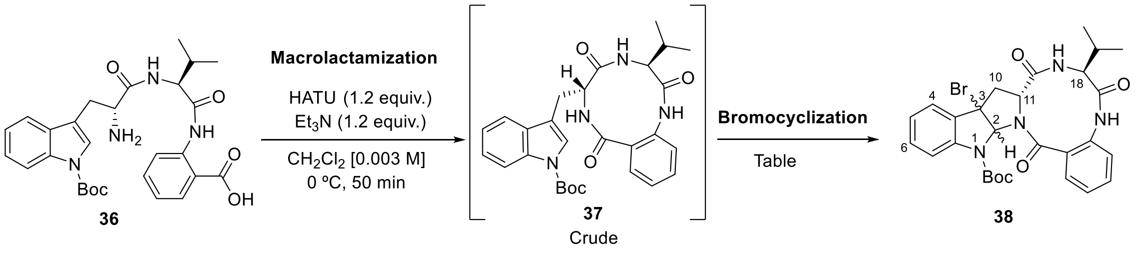

The atom numbering from the original publication of the isolation of the natural product is used.[5]

Entry	Reaction conditions BROMOCYCLIZATION[6-8]	*Exo/Endo* ratio	Yield (over 2 steps)
**1**	NBS, CH_3CN, -40 ºC, 70 min	2.3:1	44%
**2**	NBS, PPTS, CH_3CN, -40 ºC, 70 min	15:1	56%

Entry	Reaction conditions BROMOCYCLIZATION[6-8]	*Exo/Endo* ratio	Yield (over 2 steps)
**3**	NBS, CH_2Cl_2, -30 ºC, 50 min	5:1 + trace amounts of other diastereomers	~50%
**4**	Br_2 (4.8 equiv.), CH_2Cl_2, 0 ºC, 10 min	2:1	~53%
**5**	Br_2 (4.8 equiv.), CH_2Cl_2, -30 ºC, 25 min	1:2	~27%
**6**	Br_2 (1.5 equiv.), CH_2Cl_2, -30 ºC, 25 min	4.3:1	33%
**7**	Br_2 (2.4 equiv.), CH_2Cl_2, 0 ºC (very slow addition of the solution of Br_2 in CH_2Cl_2 over a period of 15 min)	1.6:1	33%

General procedure for the macrolactamization. To a cooled (0 ºC) solution of the tryptophan derivative **36** (25.0 mg, 0.05 mmol) in CH_2Cl_2 (16.0 mL) were added Et_3N (8.0 μL, 6.0 mg, 0.06 mmol) and HATU (22.0 mg, 0.06 mmol). After stirring the mixture at 0 ºC for 50 min, H_2O was added, the layers were separated, and the aqueous layer was extracted with CH_2Cl_2 (3x). The combined organic layers were dried (anhydrous Na_2SO_4) and the solvent was evaporated. The residue was immediately used in the next step without further purification.

General procedure for the bromocyclization with *N*-bromosuccinimide (Method A. Entry 2). To a stirred solution of the macrolactam **37** (0.05 mmol) in CH_3CN at **-** 40 ºC, NBS (9.0 mg, 0.05 mmol) and PPTS (12.0 mg, 0.05 mmol) were added. The reaction mixture was stirred at this temperature until completion of the reaction as judged by TLC. The mixture was diluted with EtOAc, and the organic layer was washed with H_2O (2x). The organic layer was dried over anhydrous Na_2SO_4, and the solvent was concentrated. The residue was purified by flash column chromatography (silica gel, gradient from 80:20 to 60:40 *v/v* hexane/EtOAc) to afford 15.3 mg (53% yield) of the *exo*-**38** isomer and 1 mg (3% yield) of the *endo*-**38** isomer, both as white solids (56% yield, over two steps).

General procedure for the bromocyclization with bromine (Method B. Entry 4). To a solution of the macrolactam **37** (48.0 mg, 0.10 mmol) in CH_2Cl_2 (1.2 mL) at 0 ºC, a solution of bromine in CH_2Cl_2 (0.46 mL, 1M) was added dropwise. The reaction mixture was stirred at this temperature for 10 min. A saturated aqueous solution of $Na_2S_2O_3$ was added to the reaction mixture. Once the red color dissipated, the mixture was diluted with EtOAc and the organic layer was washed with a saturated aqueous solution of $NaHCO_3$ (2 x), water (2 x) and brine. The organic layer was dried over anhydrous Na_2SO_4, and the solvent was evaporated. The residue was purified by flash column chromatography (silica gel, 70:30 *v/v* hexane/EtOAc) to afford 0.03 g (53% yield) of the titled compound as a mixture of diastereomers (a 2:1 ratio of the *exo*/*endo*-**38** diastereomers was observed in the ^{1}H NMR of the crude).

***Exo*-Br-precursor-Boc-Novofumigatamide (*exo*-38). ^{1}H NMR** (400 MHz, $CDCl_3$, 328 K) δ 8.20 (d, *J* = 7.8 Hz, 1H, ArH), 7.83 (t, *J* = 7.7 Hz, 1H, ArH), 7.65 (d, *J* = 8.0 Hz, 1H, ArH), 7.64-7.53 (m, 1H, HSQC, ArH), 7.54 (t, *J* = 7.6 Hz, 1H, ArH), 7.41 (d, *J* = 7.6 Hz, 1H, ArH), 7.33 –

7.20 (m, 1H, ArH), 7.08 (t, J = 7.5 Hz, 1H, ArH), 5.98 (br s, 1H, H_2), 4.82 (dd, J = 9.4, 5.2 Hz, 1H, H_{18}), 3.66 (dd, J = 10.6, 5.4 Hz, 1H, H_{11}), 3.09 (dd, J = 12.6, 5.4 Hz, 1H, H_{10A}), 2.93 (dd, J = 12.6, 10.6 Hz, 1H, H_{10B}), 2.50 – 2.36 (m, 1H, C$\underline{H}$(CH_3)$_2$), 1.63 (s, 9H, CO_2*t*Bu), 1.04 (d, J = 6.8 Hz, 3H, CH(C$\underline{H_3}$)$_2$), 0.98 (d, J = 6.9 Hz, 3H, CH(C$\underline{H_3}$)$_2$) ppm. **^{13}C{^{1}H} NMR** (101 MHz, $CDCl_3$) δ (mixture of rotamers; data for the major rotamer) 171.9 (s), 161.3 (s), 159.0 (s), 152.2 (s), 146.1 (s), 140.9 (s, HMBC), 136.7 (d), 132.8 (s, HMBC), 130.7 (d), 128.8 (d, 2x), 127.2 (d), 125.4 (d), 124.1 (d), 117.3 (s), 115.1 (d), 87.7 (d), 83.0 (s, HMBC), 61.8 (s, HMBC), 60.5 (d), 56.4 (d), 49.9 (t), 31.5 (d), 28.7 (q, 3x), 19.6 (q), 17.8 (q) ppm. **HRMS** (ESI-TOF) *m/z*: $[M + H]^+$ calcd. for $C_{28}H_{32}{}^{79}BrN_4O_5$, 583.1551; found, 583.1532. **IR** (NaCl): ν 3300 – 3100 (br, N-H), 2969 (m, C-H), 2930 (m, C-H), 1763 (m, C=O), 1684 (s, C=O),1646 (m, C=O), 1604 (m, C=O), 1521 (m), 1481 (m), 1373 (m), 1278 (m), 1254 (m),1160 (m), 1110 (w), 1086 (w), 1040 (w), 1012 (w), 850 (w), 755 (s) cm^{-1}. $[\alpha]_D^{23}$ +44.1 (*c* 0.19, $CHCl_3$).

***Endo*-Br-precursor-Boc-Novofumigatamide (*endo*-38).** **^{1}H NMR** (400 MHz, $CDCl_3$, 328 K) δ 8.21 (dd, J = 7.9, 1.4 Hz, 1H, ArH), 7.81 (ddd, J = 8.0, 7.3, 1.5 Hz, 1H, ArH), 7.62 – 7.49 (m, 2H, ArH), 7.47 (d, J = 8.0 Hz, 1H, ArH), 7.35 – 7.28 (m, 1H, ArH), 6.84 (t, J = 7.6 Hz, 1H, ArH), 6.74 – 6.66 (m, 1H, ArH), 5.93 (s, 1H, H_2), 4.38 (dd, J = 9.0, 6.4 Hz, 1H, H_{18}), 4.09 (dd, J = 8.8, 4.1 Hz, 1H, H_{11}), 3.26 (dd, J = 13.6, 4.0 Hz, 1H, H_{10A}), 3.18 – 3.05 (m, 1H, H_{10B}), 2.18 – 2.06 (m, 1H, C$\underline{H}$(CH_3)$_2$), 1.65 (s, 9H, CO_2*t*Bu), 0.89 (d, J = 5.7 Hz, 3H, CH(C$\underline{H_3}$)$_2$), 0.87 (d, J = 5.6 Hz, 3H, CH(C$\underline{H_3}$)$_2$) ppm. **^{13}C{^{1}H} NMR** (101 MHz, $CDCl_3$, 328 K) δ 172.0 (s), 160.4 (s), 158.9 (s), 146.0 (s), 140.3 (s, HMBC), 136.6 (d), 130.1 (d), 128.8 (d), 128.5 (d), 127.5 (d), 124.9 (d), 123.6 (d), 117.4 (s), 115.1 (d), 88.4 (d), 82.5 (s, HMBC), 62.8 (s, HMBC), 60.9 (d), 57.0 (d), 46.8 (t), 32.3 (d), 28.6 (q, 3x), 19.1 (q), 18.2 (q) ppm. **HRMS** (ESI-TOF) *m/z*: $[M + H]^+$ calcd. for $C_{28}H_{32}{}^{79}BrN_4O_5$, 583.1551; found, 583.1538. $[\alpha]_D^{25}$ –96.2 (*c* 0.14, MeOH).

REMARK: this compound decomposes during characterization in $CDCl_3$ solution at 328 K. Low resolution of the signals is observed when the ^{1}H NMR spectrum was recorded at 298 K. Two ^{13}C{^{1}H} NMR signals are missing.

***Exo*-*N*-Boc-*N*-deacetylnovofumigatamide (*exo*-39)**

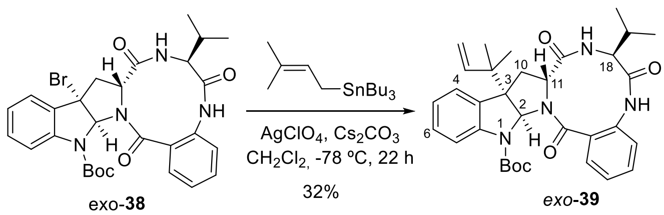

exo-**38** *exo*-**39**

General procedure for the reverse prenylation with tributyl(3-methyl-2-butenyl)stannane. Method A. To a solution of the *exo*-Br-precursor-Boc-novofumigatamide (*exo*-**38**) (32.0 mg, 55.0 μmol), Cs_2CO_3 (27.0 mg, 82.0 μmol) and tributyl(3-methyl-2-butenyl)stannane[9-10] **30** (0.03 g, 82.0 μmol) in CH_2Cl_2 (0.8 mL) at −78 °C was added $AgClO_4$ (23.0 mg, 0.11 mmol). After stirring

at −78 °C for 22 h, the reaction was quenched with a saturated aqueous solution of NH_4Cl, and the mixture warmed up to room temperature. The layers were separated, and the aqueous layer was extracted with CH_2Cl_2 (3x). The combined organic layers were dried over anhydrous Na_2SO_4, filtered and the solvent was evaporated. The residue was purified by flash column chromatography (silica gel, gradient from 80:20 to 50:50 hexane/EtOAc). A second purification (CombiFlash® Rf+ system, 13 g C18 silica gel, gradient from 70:30 *v/v* CH_3CN/H_2O to CH_3CN 100%, flow rate = 30 mL/min) was required to remove the tin salts by-products and obtain a pure sample of *N*-Boc-protected synthetic novofumigatamide *exo*-**39** (0.01 g, 32%) as a white solid. **^{1}H NMR** (400 MHz, $CDCl_3$, 328 K) δ 8.21 (dd, *J* = 7.9, 1.5 Hz, 1H, ArH), 7.81 (ddd, *J* = 8.0, 7.3, 1.6 Hz, 1H, ArH), 7.63 – 7.49 (m, 3H, ArH), 7.26 – 7.16 (m, 2H, ArH), 6.97 (td, *J* = 7.5, 1.1 Hz, 1H, ArH), 6.01 (dd, *J* = 17.4, 10.8 Hz, 1H, $C(CH_3)_2C\underline{H}=CH_2$), 5.57 (br s, 1H, H_2), 5.13 – 5.00 (m, 2H, $C(CH_3)_2CH=C\underline{H_2}$), 4.83 (dd, *J* = 9.3, 5.3 Hz, 1H, H_{18}), 3.66 – 3.61 (m, 1H, H_{11}), 2.45 – 2.35 (m, 1H, $C\underline{H}(CH_3)_2$), 2.36 – 2.25 (m, 2H, $2H_{10}$), 1.63 (s, 9H, CO_2*t*Bu), 1.11 (s, 3H, $C(C\underline{H_3})_2CH=CH_2$), 1.10 (s, 3H, $C(C\underline{H_3})_2CH=CH_2$), 1.04 (d, *J* = 6.8 Hz, 3H, $CH(C\underline{H_3})_2$), 0.99 (d, *J* = 6.9 Hz, 3H, $CH(C\underline{H_3})_2$) ppm. **^{13}C{^{1}H} NMR** (101 MHz, $CDCl_3$, 328 K) δ 173.8 (s), 161.6 (s), 159.0 (s), 146.2 (s), 144.3 (d), 142.7 (s, HMBC), 136.6 (d), 133.9 (s, HMBC), 128.8 (d), 128.7 (d), 128.6 (d), 127.0 (d), 125.5 (d), 122.8 (d), 117.4 (s), 114.6 (d), 114.0 (t), 82.2 (s, HMBC, $CO_2\underline{C}(CH_3)_3$), 81.3 (d, C_2), 61.7 (s, HMBC, C_3), 60.4 (d, C_{11}), 56.2 (d, C_{18}), 41.5 (s, $\underline{C}(CH_3)_2CH=CH_2$), 41.2 (t, C_{10}), 31.8 (d, $\underline{C}H(CH_3)_2$), 28.8 (q, $CO_2C(\underline{C}H_3)_3$), 23.8 (q, $C(\underline{C}H_3)_2CH=CH_2$), 22.9 (q, $C(\underline{C}H_3)_2CH=CH_2$), 19.6 (q, $CH(\underline{C}H_3)_2$), 17.8 (q, $CH(\underline{C}H_3)_2$) ppm. **IR** (NaCl): ν 3500–3200 (br, N–H), 3080 (w, C-H), 2967 (s, C-H), 2926 (s, C-H), 2873 (m, C-H), 2852 (m, C-H), 1764 (s, C=O), 1682 (s, C=O), 1643 (s, C=O), 1607 (m), 1514 (m), 1484 (s), 1369 (s), 1286 (m), 1252 (s), 1162 (s), 1137 (s), 1039 (m), 1008 (m), 920 (w), 865 (w), 754 (s) cm^{-1}. **HRMS** (ESI-TOF) *m/z*: $[M + H]^+$ calcd. for $C_{33}H_{40}N_4O_5$, 573.3071; found, 573.3069. $[\alpha]_D^{25}$ +26.5 (*c* 0.19, $CHCl_3$).

REMARK[1]: One ^{13}C{^{1}H} NMR signal is missing.

REMARK[2]: some solubility issues were found when MeOH was used as solvent to measure the specific optical rotations for some intermediates and final products. Remarkably, the first final product synthesized, the proposed structure of novofumigatamide (**11**), was not fully soluble in this solvent, which prompted us to use $CHCl_3$ as solvent to measure this physical property for this and the subsequent final products.

Preparation of synthetic novofumigatamide from D-Trp and L-Val following route A.1

Methyl (*R*)-3-(1'-Acetylindole-3'-yl)-2-(phenylmethoxycarbonylamino)propanoate (*R*)-25

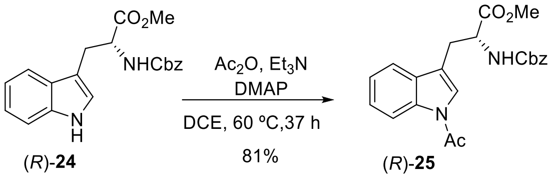

General procedure for the acetylation of amines. Tryptophan derivative (*R*)-**24** (2.86 g, 8.11 mmol) and 4-dimethylaminopyridine (DMAP) (0.2 g, 1.62 mmol) were added to a Schlenk flask and dissolved in DCE (81.2 mL). Triethylamine (1.7 mL, 1.23 g, 12.17 mmol) and acetic anhydride (2.3 mL, 2.48 g, 24.34 mmol) were added, the tube was sealed, and the reaction mixture was stirred in a metal heating block at 60 ºC. After 20 h at 60 ºC, another portion of DMAP (0.2 g, 1.62 mmol), triethylamine (1.7 mL, 1.23 g, 12.17 mmol) and acetic anhydride (1.15 mL, 1.24 g, 12.17 mmol) was added and the mixture was further stirred for 17 h. EtOAc and a saturated aqueous solution of NH_4Cl were added, the layers were separated, and the aqueous layer was extracted with EtOAc (3x). The combined organic layers were dried over anhydrous Na_2SO_4, and the solvent was evaporated. The residue was purified by flash column chromatography (silica gel, 70:30 *v/v* hexane/EtOAc) to afford 2.58 g (81% yield) of the titled compound as a white solid. **M.p.**: 119-121 ºC (hexane/CH_2Cl_2/MeOH). **^{1}H NMR** (400 MHz, $CDCl_3$) δ 8.34 (d, *J* = 7.9 Hz, 1 H, ArH), 7.41 (d, *J* = 7.7 Hz, 1H, ArH), 7.31 – 7.22 (m, 6H, ArH), 7.21 – 7.10 (m, 2H, ArH), 5.48 (d, *J* = 8.1 Hz, 1H, N<u>H</u>Cbz), 5.09 (d, *J* = 12.3 Hz, 1H, OC$\underline{H_{2A}}$Ph), 5.00 (d, *J* = 12.3 Hz, 1H, OC$\underline{H_{2B}}$Ph), 4.75 – 4.65 (m, 1H, H_2), 3.63 (s, 3H, $CO_2C\underline{H_3}$), 3.22 (dd, *J* = 14.9, 5.3 Hz, 1H, H_{3A}), 3.12 (dd, *J* = 15.0, 6.3 Hz, 1H, H_{3B}), 2.43 (s, 3H, $COC\underline{H_3}$) ppm. **^{13}C{^{1}H} NMR** (101 MHz, $CDCl_3$) δ 172.0 (s), 168.4 (s), 155.8 (s), 136.3 (s), 135.8 (s), 130.4 (s), 128.6 (d, 2x), 128.3 (d), 128.1 (d, 2x), 125.5 (d), 123.7 (d), 123.5 (d), 118.7 (d), 116.9 (s), 116.7 (d, 2x), 67.0 (t), 53.8 (d), 52.6 (q), 28.0 (t), 24.0 (q) ppm. **IR** (NaCl): ν 3308 (w, N-H), 3111 (w, C-H), 3031 (w, C-H), 2948 (w, C-H), 1737 (s, C=O), 1697 (s, C=O), 1687 (s, C=O), 1547 (m), 1525 (m), 1455 (m), 1391 (m), 1283 (m), 1227 (s), 1193 (m), 1172 (m), 1149 (m), 1064 (m), 1006 (s) cm^{-1}. **HRMS** (ESI-TOF) *m/z*: $[M + H]^+$ calcd. for $C_{22}H_{23}N_2O_5$, 395.1602; found, 359.1601. $[\alpha]_D^{24}$ –33.9 (*c* 0.5, $CHCl_3$).

Methyl (*S*)-3-(1'-Acetylindole-3'-yl)-2-(phenylmethoxycarbonylamino)propanoate (*S*)-25: [α]D20 +36 (*c* 2.4, $CHCl_3$).[4]

(*R*)-3-(1'-Acetylindole-3'-yl)-2-(phenylmethoxycarbonylamino)propanoic Acid (*R*)-19

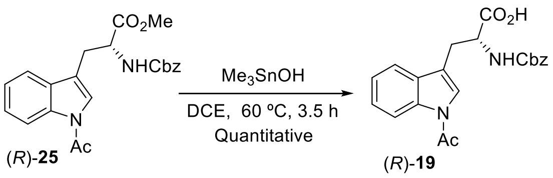

General procedure for the hydrolysis of esters. Method B. Methyl ester (*R*)-**25** (0.68 g, 1.72 mmol) was dissolved in DCE (17.2 mL) and, after addition of trimethyltin hydroxide (1.55 g, 8.6 mmol), the mixture was stirred in a metal heating block at 60 ºC for 3.5 h. After completion of the reaction, the mixture was concentrated *in vacuo*, and the residue was taken up in ethyl acetate. The organic layer was washed with a 5% aqueous solution of HCl (3x), and brine, dried over anhydrous Na_2SO_4 and the solvent was removed *in vacuo*. The residue was purified by flash column chromatography (silica gel, 90:10 *v/v* CH_2Cl_2/MeOH) to afford 0.65 g (quantitative yield) of the titled compound as a white solid. **M.p.**: 168-170 ºC (hexane/CH_2Cl_2/MeOH). **^{1}H NMR** (400 MHz, DMSO-d_6, 343 K) δ 8.27 (d, *J* = 8.1 Hz, 1H, ArH), 7.60 (d, *J* = 7.7 Hz, 1H, ArH), 7.54 (s, 1H, ArH), 7.34 – 7.17 (m, 7H, ArH), 6.74 (br s, 1H, NH), 5.01 (d, *J* = 12.7 Hz, 1H, $OC\underline{H_{2A}}Ph$), 4.94 (d, *J* = 12.7 Hz, 1H, $OC\underline{H_{2B}}Ph$), 4.21 (app. td, *J* = 7.5, 4.9 Hz, 1H, H_2), 3.23 (dd, *J* = 14.7, 4.5 Hz, 1H, H_{3A}), 3.03 (dd, *J* = 14.7, 7.6 Hz, 1H, H_{3B}), 2.53 (s, 3H, $COC\underline{H}_3$) ppm. **^{13}C{^{1}H} NMR** (101 MHz, DMSO-d_6, 343 K) δ 173.8 (s), 168.3 (s), 155.2 (s), 136.9 (s), 134.8 (s), 130.7 (s), 127.8 (d), 127.2 (d), 127.0 (d), 124.0 (d), 123.9 (d), 122.6 (d), 118.8 (d), 118.3 (s), 115.4 (d), 64.9 (t), 55.0 (d), 27.3 (t), 23.3 (q) ppm. **IR** (neat): ν 3500-3100 (br, O-H), 3039 (w, C-H), 2969 (w, C-H), 1687 (s, C=O), 1585 (m), 1539 (m), 1447 (m), 1388 (m), 1328 (m), 1248 (s), 1219 (s), 1144 (w), 1038 (s), 934 (w), 882 (w), 744 (s), 695 (m), 650 (m) cm^{-1}. **HRMS** (ESI-TOF) *m/z*: $[M + H]^+$ calcd. for $C_{21}H_{21}N_2O_5$, 381.1445; found, 381.1434. $[\alpha]_D^{26}$ –4.5 (*c* 0.14, MeOH).

(*S*)-3-(1'-Acetylindole-3'-yl)-2-(phenylmethoxycarbonylamino)propanoic Acid (*S*)-19: $[\alpha]_D^{22}$ +6.7 (*c* 0.14, MeOH).

(2'*S*,2''*R*)-2-{2'-[3''-(1'''-Acetylindole-3'''-yl)-2''-(phenylmethoxycarbonylamino)propanamido]-3'-methylbutanamido}benzoic Acid 28

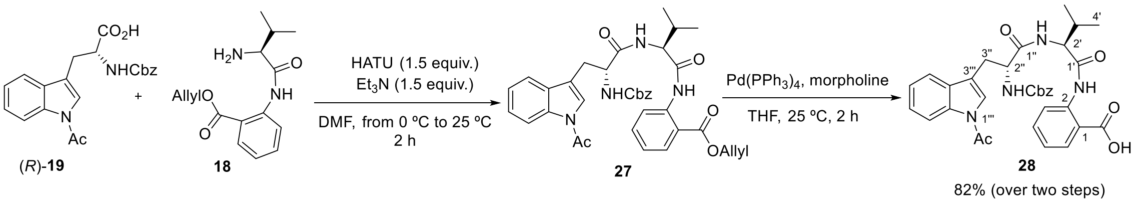

Following the general procedure described above for amide (peptide) bond formation (Method B), the reaction of tryptophan derivative (*R*)-**19** (1.1 g, 2.89 mmol), dipeptide **18** (0.8 g, 2.89 mmol), HATU (1.65 g, 4.34 mmol) and Et_3N (0.6 mL, 0.44 g, 4.34 mmol) in DMF (39 mL) at 25 ºC afforded, after 2 h of reaction time, a residue that was used in the next step without further purification.

To a stirred solution of the previous residue (2.89 mmol) and $Pd(PPh_3)_4$ (0.33 g, 0.29 mmol) in THF (152 mL), morpholine (0.5 mL, 0.5 g, 5.78 mmol) was added dropwise and the mixture was stirred for 30 min at 25 ºC. The reaction mixture was diluted with EtOAc, and the organic layer was washed with 1M aqueous solution of HCl (2x) and brine (2x), then dried over anhydrous Na_2SO_4 and the solvent was evaporated. The residue was purified by flash column

chromatography (CombiFlash® Rf+ system, 120 g silica gel, gradient from 98:2 to 90:10 *v/v* CH_2Cl_2/MeOH, flow rate = 85 mL/min) to afford 1.43 g (82% yield, over two steps) of the titled compound as a white solid. **^{1}H NMR** (400 MHz, DMSO-d_6, 343 K) δ 8.48 (d, *J* = 8.3 Hz, 1H, ArH), 8.29 (d, *J* = 8.0 Hz, 1H, ArH), 8.06 – 7.95 (m, 2H, ArH), 7.71 (d, *J* = 7.6 Hz, 1H, ArH), 7.66 (s, 1H, ArH), 7.44-7.37 (m, 1H, ArH), 7.35 – 7.18 (m, 6H, ArH), 7.05 (t, *J* = 7.5 Hz, 1H, ArH), 5.05 – 4.89 (m, 2H, OC<u>H</u>$_2$Ph), 4.69 – 4.54 (m, 1H, $H_{2''}$), 4.28 – 4.14 (m, 1H, $H_{2'}$), 3.21 (dd, *J* = 15.4, 5.1 Hz, 1H, $H_{3A''}$), 3.02 (dd, *J* = 14.8, 8.8 Hz, 1H, $H_{3B''}$), 2.53 (s, 3H, COC<u>H</u>$_3$), 2.23 – 2.05 (m, 1H, C<u>H</u>(CH_3)$_2$), 0.87 (d, *J* = 6.8 Hz, 3H, CH(C<u>H</u>$_3$)$_2$), 0.85 (d, *J* = 6.8 Hz, 3H, CH(C<u>H</u>$_3$)$_2$) ppm. **^{13}C{^{1}H} NMR** (101 MHz, DMSO-d_6, 343 K) δ 171.0 (s), 169.8 (s), 169.0 (s), 168.3 (s), 155.4 (s), 139.9 (s), 136.6 (s), 134.8 (s), 131.0 (d), 130.8 (d), 130.2 (s), 127.8 (d), 127.2 (d), 126.9 (d), 124.5 (d), 124.2 (d), 122.7 (d), 121.6 (d), 118.8 (d), 118.7 (d), 117.2 (s), 115.4 (d), 65.2 (t), 59.5 (d), 54.6 (d), 29.9 (d), 27.4 (t), 23.2 (q), 18.8 (q), 17.6 (q) ppm. **HRMS** (ESI-TOF) *m/z*: [M + H]$^+$ calcd. for $C_{33}H_{35}N_4O_5$, 599.2500; found, 599.2476. **IR** (NaCl): ν 3500–3100 (br, O-H, N-H), 2961 (w, C-H), 2925 (w, C-H), 1677 (m, C=O), 1658 (m, C=O), 1643 (m, C=O), 1588 (m), 1510 (s), 1503 (s), 1450 (s), 1386 (s), 1330 (m), 1291 (m), 1248 (m), 1224 (m), 1165 (m), 1144 (m), 1084 (m), 1042 (m), 1011 (m), 936 (m), 900 (m), 846 (m), 746 (s), 697 (s) cm^{-1}. **$[\alpha]_D^{23}$** +29.7 (*c* 0.15, $CHCl_3$).

REMARK: one ^{13}C{^{1}H} NMR signal is missing.

(2’*S*,2’’*R*)-2-{2’-[-3’’-(1’’’-Acetylindole-3’’’-yl)-2’’-aminopropanamido]-3’-methylbutanamido}benzoic Acid 17

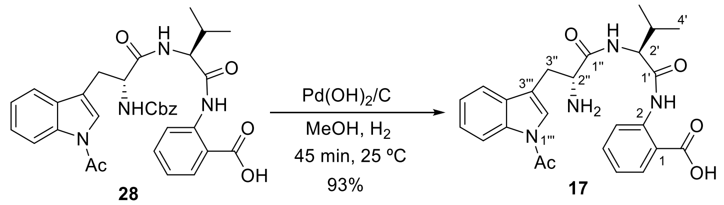

Following the general procedure described above for Cbz deprotection, the reaction of Cbz-protected amine **28** (0.49 g, 0.82 mmol), $Pd(OH)_2$/C (0.38 g, 2.4 mmol) in MeOH (82 mL) under hydrogen atmosphere afforded, after 45 min of reaction time, a white solid (0.36 g, 93% yield) which was used in the next step without further purification. **^{1}H NMR** (400 MHz, DMSO-d_6, 343 K) δ 8.37 (d, *J* = 8.2 Hz, 1H, ArH), 8.30 (d, *J* = 7.8 Hz, 1H, ArH), 7.94 (d, *J* = 7.9 Hz, 1H, ArH), 7.72 (s, 1H, ArH), 7.65 (d, *J* = 7.3 Hz, 1H, ArH), 7.38 – 7.22 (m, 3H, ArH), 6.96 (t, *J* = 7.5 Hz, 1H, ArH), 4.25 (t, *J* = 7.6 Hz, 1H, $H_{2''}$), 4.15 (d, *J* = 4.3 Hz, 1H, $H_{2'}$), 3.21 (dd, *J* = 14.3, 8.5 Hz, 1H, $H_{3A''}$), 3.05 (dd, *J* = 14.3, 6.3 Hz, 1H, $H_{3B''}$), 2.61 (s, 3H, $COCH_3$), 2.35 – 2.23 (m, 1H, C<u>H</u>(CH_3)$_2$), 0.75 (d, *J* = 6.9 Hz, 3H, CH(C<u>H</u>$_3$)$_2$), 0.57 (d, *J* = 6.9 Hz, 3H, CH(C<u>H</u>$_3$)$_2$) ppm. **^{13}C{^{1}H} NMR** (101 MHz, DMSO-d_6, 343 K) δ 170.4 (s), 169.0 (s), 168.5 (s), 168.4 (s), 139.3 (s), 134.9 (s), 130.8 (d), 129.9 (s), 129.7 (d), 124.8 (d), 124.7 (s), 124.4 (d), 122.7 (d), 121.4 (d), 118.7 (d),

118.2 (d), 115.7 (s), 115.5 (d), 58.8 (d), 52.7 (d), 28.1 (d), 26.8 (t), 23.2 (q), 18.4 (q), 16.5 (q) ppm. **HRMS** (ESI-TOF) *m/z*: $[M + H]^+$ calcd. for $C_{25}H_{29}N_4O_5$, 465.2132; found, 465.2115. **IR** (neat): ν 3500-3000 (br, O-H, N-H), 2962 (w, C-H), 2927 (w, C-H), 1671 (m, C=O), 1585 (m), 1502 (s), 1444 (s), 1371 (s), 1297 (m), 1249 (m), 1221 (m), 1143 (w), 1038 (w), 934 (w), 830 (w), 752 (s), 703 (m), 660 (m) cm^{-1}. $[\alpha]_D^{24}$ –123.9 (*c* 0.09, MeOH).

***Exo*-Br-novofumigatamide (*exo*-29)**

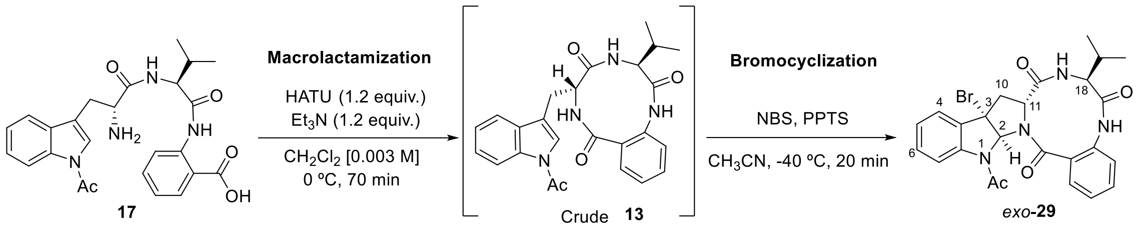

Following the general procedure described above for the macrolactamization, the reaction of the tryptophan derivative **17** (25 mg, 54 μmol), Et_3N (9.0 μL, 7.0 mg, 65.0 μmol) and HATU (25.0 mg, 65.0 μmol) in CH_2Cl_2 (18 mL) at 0 ºC for 70 min, afforded a residue that was immediately used in the next step without further purification.

Following the general procedure described above for the bromocyclization with *N*-bromosuccinimide (Method A), the reaction of macrolactam **13** (54 μmol), NBS (10.0 mg, 54.0 μmol) and PPTS (14.0 mg, 54.0 μmol) in CH_3CN (0.9 mL) at – 40 ºC for 20 min afforded, after purification by flash column chromatography (silica gel, 60:40 *v/v* hexane/EtOAc), 15.0 mg (53% yield, over two steps) of the *exo* isomer (*exo*-**29)** as a white solid and as a single product. **^{1}H NMR** (400 MHz, $CDCl_3$, 323 K) δ 8.20 (ddd, *J* = 7.9, 1.5, 0.4 Hz, 1H, ArH), 7.81 (ddd, *J* = 8.1, 7.3, 1.6 Hz, 1H, ArH), 7.62 (d, *J* = 8.1 Hz, 1H, ArH), 7.57 – 7.49 (m, 2H, ArH), 7.47 (d, *J* = 7.7 Hz, 1H, ArH), 7.33 (ddd, *J* = 8.4, 7.5, 1.4 Hz, 1H, ArH), 7.17 (td, *J* = 7.6, 1.0 Hz, 1H, ArH), 5.92 (br s, 1H, H_2), 4.86 (dd, *J* = 9.2, 5.3 Hz, 1H, H_{11}), 3.63 (dd, *J* = 10.1, 5.8 Hz, 1H, H_{18}), 3.24 – 3.05 (m, 1H, H_{10A}), 2.95 (dd, *J* = 12.8, 10.1 Hz, 1H, H_{10B}), 2.47 – 2.30 (m, 4H, $C\underline{H}(CH_3)_2$ + $COC\underline{H_3}$), 1.04 (d, *J* = 6.8 Hz, 3H, $CH(C\underline{H_3})_2$), 0.99 (d, *J* = 6.9 Hz, 3H, $CH(C\underline{H_3})_2$) ppm. **^{13}C{^{1}H} NMR** (101 MHz, $CDCl_3$, 323 K) δ (mixture of rotamers; data for the major rotamer) 171.4 (s), 169.2 (s), 161.2 (s), 158.91 (s) 145.9 (s), 141.4 (s), 136.8 (d), 135.2 (d, HSQC), 132.7 (s, HMBC), 130.9 (d), 128.9 (d), 128.8 (d), 127.2 (d), 125.3 (d), 124.9 (d), 117.2 (s), 88.3 (d), 62.9 (s, HMBC), 60.8 (d), 56.6 (d), 48.8 (t), 31.7 (d), 24.0 (q, HSQC), 19.5 (q), 17.8 (q) ppm. **HRMS** (ESI-TOF) *m/z*: $[M + H]^+$ calcd. for $C_{25}H_{26}{}^{79}BrN_4O_4$, 525.1132; found, 525.1126. **IR** (NaCl): ν 3400-3000 (br, N-H), 2966 (w, C-H), 2930 (w, C-H), 1763 (m, C=O), 1712 (s, C=O), 1677 (s, C=O), 1604 (m, C=O), 1523 (m), 1474 (m), 1392 (m), 1286 (m), 1249 (m), 1177 (m), 756 (s) cm^{-1}. $[\alpha]_D^{21}$ +44.3 (*c* 0.22, $CHCl_3$).

REMARK: the presence of rotamers, even at high temperatures, makes the interpretation cumbersome. High temperatures induce decomposition of the compound, which complicates the interpretation of the spectra.

Synthetic novofumigatamide 11

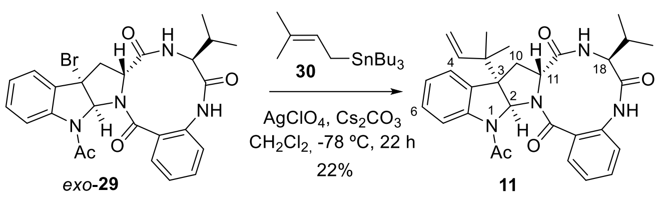

Reverse prenylation with tributyl(3-methyl-2-butenyl)stannane 30. Following the general procedure described above for the reverse prenylation with tributyl(3-methyl-2-butenyl)stannane **30** (Method A), the reaction of bromopyrroloindoline *exo*-**29** (0.06 g, 0.11 mmol), Cs_2CO_3 (0.06 g, 0.17 mmol), prenyl tributylstannane **30** (0.06 g, 0.17 mmol) and $AgClO_4$ (0.05 g, 0.23 mmol) in CH_2Cl_2 (1.7 mL) at −78 °C for 22 h afforded, after purification by flash column chromatography (silica gel, gradient from 80:20 to 50:50 *v/v* hexane/EtOAc), synthetic novofumigatamide **11** (13.0 mg, 22%) as a white solid.

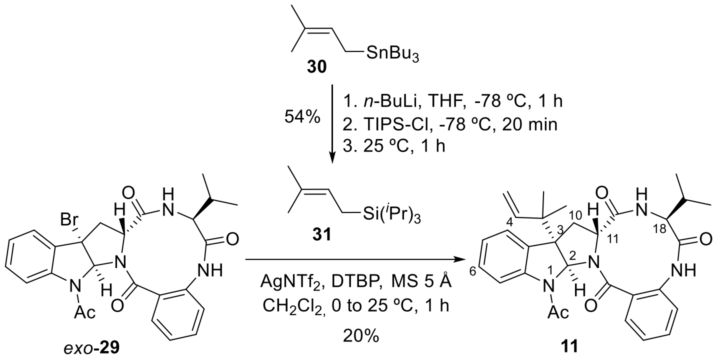

Synthesis of Triisopropyl(3-methyl-2-butenyl)silane 31.[11-13] To a cooled (- 78 ºC) stirred solution of tributyl(3-methyl-2-butenyl)stannane **30** (0.55 g, 1.54 mmol) in THF (14 mL), *n*-butyllithium (0.81 mL, 1.9 M in hexane, 1.54 mmol) was dropwise added over a period of 5 min and the mixture was stirred for 1 h at this temperature. Then, TIPSCl (0.3 mL, 0.27 g, 1.4 mmol) was added dropwise at -78 ºC and the resulting mixture was stirred for 20 min. The solution was allowed to reach room temperature and, after 1 h, it was quenched with water, the layers were separated, and the aqueous layer was extracted with Et_2O (3x). The combined organic layers were dried over anhydrous Na_2SO_4, and the solvent was evaporated. The residue was purified by flash column chromatography (CombiFlash® Rf+ system, 26 g C18 silica gel, gradient from 100% CH_3CN to 30:70 *v/v* CH_2Cl_2/CH_3CN, flow rate = 35 mL/min) to afford 0.17 g (54%) of the title compound as a colourless oil. The spectroscopic data matched those reported in the literature.[11-13]

General procedure for the reverse prenylation with triisopropyl(3-methyl-2-butenyl)silane 31. Method B. To a cooled (0 ºC) stirred solution of brominated precursor *exo*-**29** (0.03 g, 0.06 mmol), freshly activated 5Å MS (0.34 g), DTBP (19.0 μL, 16.0 mg, 0.09 mmol) and triisopropyl(3-methyl-2-butenyl)silane **31** (0.02 g, 0.09 mmol) in CH_2Cl_2 (1.1 mL) was added $AgNTf_2$ (0.05 g, 0.11 mmol). After stirring for one minute, the resulting mixture was allowed to warm up to room temperature, stirred for 15 min and then quenched with a saturated aqueous solution of $NaHCO_3$. The mixture was extracted with CH_2Cl_2 (3x) and the combined layers were washed with H_2O, filtered through a pad of Celite® and concentrated under reduced pressure. The residue was purified by flash column chromatography (silica gel, 50:50 *v/v* hexane/EtOAc) to afford 6 mg (20%) of the titled compound as a white solid. **^{1}H NMR** (400 MHz, $CDCl_3$, 298 K) δ 8.20 (ddd, *J* = 7.9, 1.6, 0.6 Hz, 1H, ArH), 7.83 (ddd, *J* = 8.1, 7.3, 1.6 Hz, 1H, ArH), 7.63 – 7.48 (m, 2H, ArH), 7.38 – 7.19 (m, 2H, ArH), 7.18 – 7.03 (m, 2H, ArH), 6.06 – 5.85 (m, 1H, $C(CH_3)_2C\underline{H}=CH_2$), 5.78 (s, 0.5 H, $0.5H_2$), 5.42 (s, 0.5 H, $0.5H_2$), 5.09 (d, *J* = 10.8 Hz, 1H, $C(CH_3)_2CH=C\underline{H}_{cis}H$), 5.04 (dd, *J* = 17.4, 1.2 Hz, 1H, $C(CH_3)_2CH=C\underline{H}_{trans}H$), 4.81 – 4.76 (m, 1H, H_{18}), 3.68 – 3.50 (m, 1H, H_{11}), 2.52 – 2.21 (m, 6H, $COC\underline{H_3}$ + $2H_{10}$ + $C\underline{H}(CH_3)_2$), 1.10 (s, 3H, $C(C\underline{H_3})_2CH=CH_2$), 1.04 – 0.84 (m, 9H, $C(C\underline{H_3})_2CH=CH_2$ + $CH\underline{(CH_3)_2}$) ppm. **^{13}C{^{1}H} NMR** (101 MHz, $CDCl_3$, 298 K) δ 173.8 (s, rotamer: 173.0), 169.6 (s, rotamer: 168.6), 161.4 (s, rotamer: 161.1), 159.1 (s, rotamer: 159.0), 146.0 (s, rotamer: 145.8), 143.9 (d), 143.1 (s, HBMC rotamer: 141.9), 136.9 (d, rotamer: 136.8), 136.0 (s, HMBC rotamer: 133.5), 129.0 (d, rotamers: 128.9, 128.84, 128.78, 128.7, 128.6), 127.1 (d, rotamers: 127.0, 126.7), 125.3 (d), 124.2 (d), 123.8 (d), 117.3 (d), 117.2 (s, rotamer: 117.1), 114.3 (t, rotamer: 114.2), 113.6 (d), 82.0 (d, rotamer: 81.2), 63.7 (s, rotamer: 60.6, 60.5), 61.2 (d, rotamer: 60.2), 56.2 (d, rotamer: 55.9), 41.3 (s, rotamer: 41.1), 41.0 (t, rotamer: 40.0), 31.8 (d, rotamer: 31.6), 25.1 (q, rotamers: 24.2, 24.0), 23.5 (q, $C(\underline{C}H_3)_2$, rotamer: 23.4), 22.8 (q, $C(\underline{C}H_3)_2$), rotamer: 22.7), 19.6 (q, $CH(\underline{C}H_3)_2$), 17.8 (q, $CH(\underline{C}H_3)_2$, rotamer: 17.7) ppm. **^{1}H NMR** (400 MHz, **DMSO-*d*$_6$, 343 K**) δ 8.13 – 8.09 (m, 1H, ArH), 8.09 – 7.88 (m, 2H, ArH), 7.66 – 7.57 (m, 2H, ArH), 7.33 (d, *J* = 7.6 Hz, 1H, ArH), 7.22 (t, *J* = 7.8 Hz, 1H, ArH), 7.12 – 7.01 (m, 1H, ArH), 5.97 (dd, *J* = 17.2, 10.8 Hz, 1H, $C(CH_3)_2C\underline{H}=CH_2$), 5.53 (br s, 1H, H_2), 5.08 – 4.97 (m, 2H, $C(CH_3)_2CH=C\underline{H_2}$), 4.60 – 4.52 (m, 1H, H_{18}), 4.25 – 4.15 (m, 1H, H_{11}, rotamers HSQC: 3.51-3.44 (m), 3.43 – 3.33 (m)), 2.35 – 2.03 (m, 6H, $COC\underline{H_3}$ + $2H_{10}$ + $C\underline{H}(CH_3)_2$), 1.03 (s, 3H, $C(C\underline{H_3})_2CH=CH_2$), 1.00 – 0.82 (m, 9H, $C(C\underline{H_3})_2CH=CH_2$ + $CH\underline{(CH_3)_2}$) ppm. **^{13}C{^{1}H} NMR** (101 MHz, **DMSO-*d*$_6$, 343 K**) δ 171.8 (s), 168.6 (s), 160.6 (s), 158.2 (s), 145.1 (s), 144.2 (d, rotamer: 144.1), 143.2 (s, HMBC), 136.6 (d), 133.3 (s, HMBC), 130.7 (d, rotamer), 128.4 (d), 127.6 (d), 127.5 (d), 126.2 (d), 125.0 (d), 122.5 (d), 116.3 (s), 115.0 (d), 113.0 (t), 81.6 (d), 63.6 (s, HMBC), 59.64 (d, rotamers HSQC: 59.6, 59.4), 56.9 (d), 40.7 (s, HMBC), 40.4 (t), 30.1 (d), 23.6 (q, rotamer: 23.5), 23.1 (q), 22.1 (q), 18.8 (q, rotamer: 18.7), 17.8 (q) ppm. **HRMS** (ESI-TOF) *m/z*: $[M + H]^+$ calcd. for $C_{30}H_{35}N_4O_4$,

515.2653; found, 515.2646. **IR** (NaCl): ν 3400-3000 (br, N-H), 3068 (w, C-H), 2964 (m, C-H), 2929 (m, C-H), 2871 (w, C-H), 1762 (s, C=O), 1766 (s, C=O), 1607 (m), 1590 (m), 1508 (s), 1479 (s), 1397 (s), 1284 (m), 1257 (m), 1220 (m), 1135 (m), 1038 (m), 1008 (m), 756 (s) cm^{-1}. $[\alpha]_D^{24}$ +13.0 (*c* 0.12, $CHCl_3$).

REMARKS: the instability of the synthetic novofumigatamide makes the characterization at high temperature not appropriate. Nevertheless, spectra at room temperature showed mixture of rotamers with many signals. ^1H NMR spectra recorded in $CDCl_3$, at 328 K and in DMSO-d_6, at 298 K are included also in section **4. Spectra collection and HPLC-MS traces**.

Synthetic *endo*-novofumigatamide (***endo*-39**)

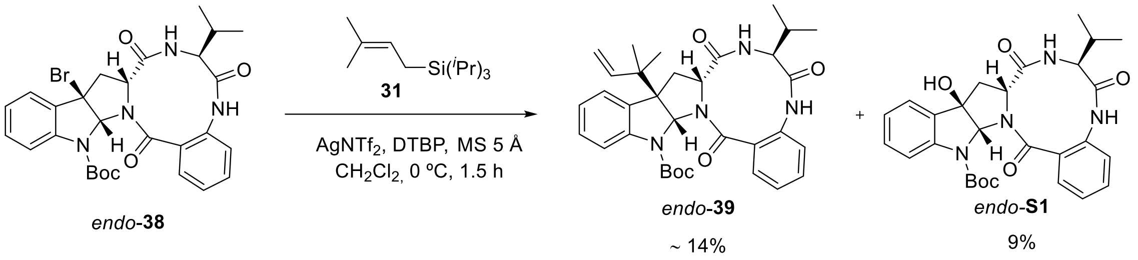

Following the general procedure described above for the reverse prenylation with triisopropyl(3-methyl-2-butenyl)silane (Method B), the reaction of *endo*-bromopyrroloindoline *endo*-**38** (14.0 mg, 24.0 μmol), prenyl triisopropyl silane (8.0 mg, 0.04 mmol) freshly activated 5Å MS (0.14 g), DTBP (8.0 μL, 7 mg, 0.04 mmol) and $AgNTf_2$ (0.02 g, 0.05 mmol) in CH_2Cl_2 (0.5 mL) at room temperature for 1 h afforded, after purification by flash column chromatography (silica gel, 70:30 *v/v* hexane/EtOAc), 2 mg of the desired compound *endo*-**39** (14%) in an impure form and 1.3 mg of the OH-*endo*-**S1** by-product (9%).

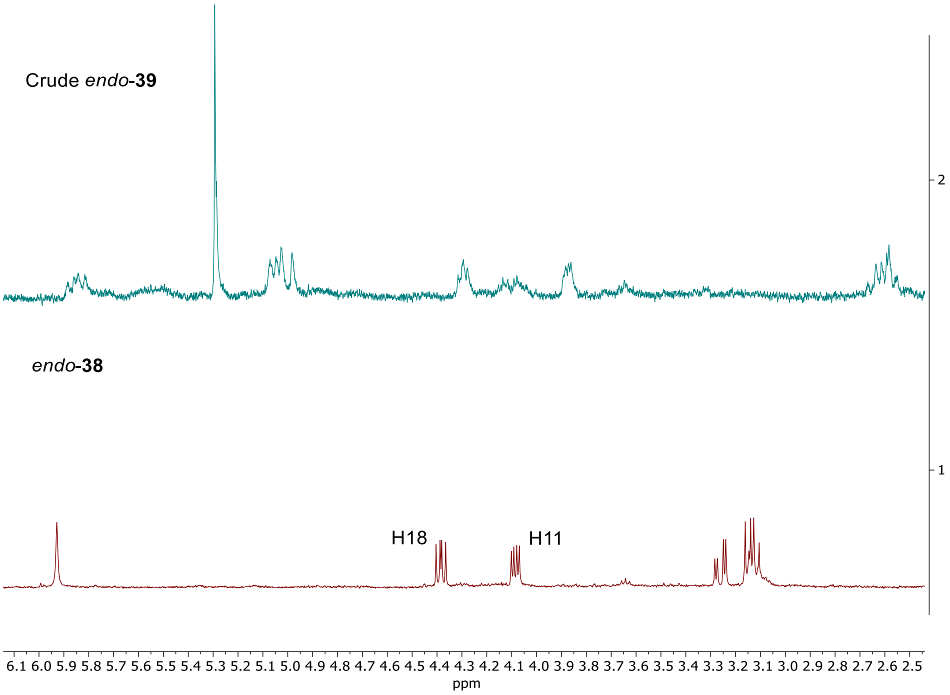

Preparation of synthetic (-)-novofumigatamide from L-Trp and L-Val following route A.1

Allyl (2'*S*,2''*S*)-2-{2'-[3''-(1'''-Acetylindole-3'''-yl)-2''-(phenylmethoxycarbonylamino)propanamido]-3'-methylbutanamido}benzoate 41

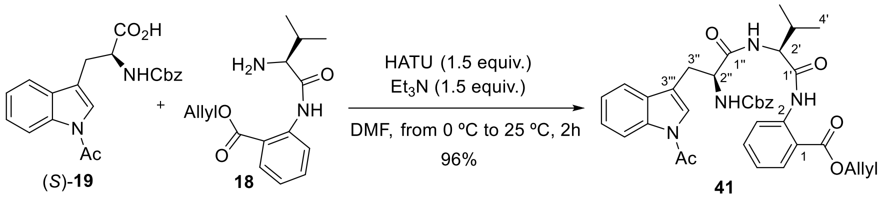

Following the general procedure described above for amide (peptide) bond formation (Method B), the reaction of tryptophan derivative (*S*)-**19** (1.5 g, 3.94 mmol), dipeptide **18** (1.09 g, 3.94 mmol), HATU (2.25 g, 5.92 mmol) and Et_3N (0.82 mL, 0.6 g, 5.92 mmol) in DMF (53.3 mL) at 25 ºC afforded, after 2 h reaction time and work-up, a residue that was purified by trituration with Et_2O (2x) and H_2O (2x) to give the desired compound (2.42 g, 96%) as a white solid. **M.p.**: 212-214 ºC (hexane/CH_2Cl_2/MeOH). **^{1}H NMR** (400 MHz, $CDCl_3$, 333 K) δ 8.59 (d, *J* = 8.5 Hz, 1H, ArH), 8.31 (d, *J* = 8.1 Hz, 1H, ArH), 8.04 (d, *J* = 7.9 Hz, 1H, ArH), 7.59 (d, *J* = 7.6 Hz, 1H, ArH), 7.53 (t, *J* = 7.9 Hz, 1H, ArH), 7.37 – 7.16 (m, 8H, ArH), 7.09 (t, *J* = 7.7 Hz, 1H, ArH), 6.45 (d, *J* = 8.1 Hz, 1H, CON<u>H</u>), 6.04 – 5.90 (m, 1H, $OCH_2C\underline{H}=CH_2$), 5.52 (d, *J* = 7.9 Hz, 1H, N<u>H</u>Cbz), 5.36 (d, *J* = 17.3 Hz, 1H, $OCH_2CH=C\underline{H}_{trans}H$), 5.27 (d, *J* = 10.5 Hz, 1H, $OCH_2CH=C\underline{H}_{cis}H$), 5.19-5.04 (m, 2H, $OC\underline{H_2}Ph$), 4.78 – 4.58 (m, 3H, $OC\underline{H_2}CH=CH_2$ + $H_{2''}$), 4.43 – 4.34 (m, 1H, $H_{2'}$), 3.33 – 3.12 (m, 2H, $2H_{3''}$), 2.40 (s, 3H, $COCH_3$), 2.26 – 2.09 (m, 1H, $C\underline{H}(CH_3)_2$), 0.90 (d, *J* = 6.8 Hz, 3H, $CH(C\underline{H_3})_2$), 0.85 (d, *J* = 6.9 Hz, 3H, $CH(C\underline{H_3})_2$) ppm. **^{13}C{^{1}H} NMR** (101 MHz, $CDCl_3$, 333 K) δ 171.1 (s), 169.7 (s), 168.4 (s), 168.0 (s), 156.2 (s), 141.1 (s), 136.5 (s), 136.2 (s), 134.9 (d), 131.8 (d), 131.1 (d), 130.5 (s), 128.7 (d), 128.4 (d), 128.2 (d), 125.5 (d), 124.4 (d), 123.8 (d), 123.2 (d), 120.7 (d), 119.0 (t), 118.9 (d), 117.3 (s), 116.9 (d), 115.6 (s), 67.4 (t), 66.1 (t), 60.2 (d), 55.2 (d), 31.4 (d), 28.2 (t), 23.8 (q), 19.3 (q), 17.9 (q) ppm. **HRMS** (ESI-TOF) *m/z*: $[M + H]^+$ calcd. for $C_{36}H_{39}N_4O_7$, 639.2813; found, 639.2805. **IR** (neat): ν 3300-3100 (br, O-H, N-H), 2964 (w, C-H), 2927 (w, C-H), 1688 (m, C=O), 1640 (s, C=O), 1590 (w, C=O), 1525 (s), 1447 (m), 1386 (m), 1338 (m), 1246 (s), 1139 (m), 1081 (m), 1047 (m), 1011 (m), 932 (m), 853 (w), 804 (w), 744 (s), 692 (m), 664 (m) cm^{-1}. $[\alpha]_D^{24}$ +10.9 (*c* 0.13, DMF).

(2'*S*,2''*S*)-2-{2'-[3''-(1'''-Acetylindole-3'''-yl)-2''-(phenylmethoxycarbonylamino)propanamido]-3'-methylbutanamido}benzoic Acid 42

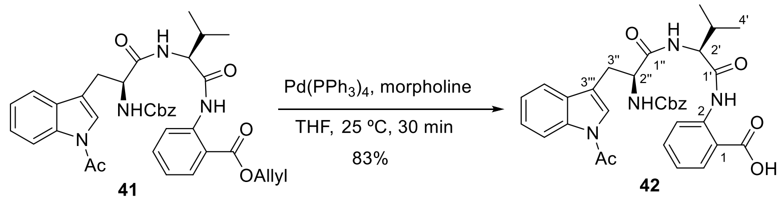

Following the general procedure described above for deprotection of allyl esters, the reaction of **41** (2.22 g, 3.48 mmol), $Pd(PPh_3)_4$ (0.4 g, 0.35 mmol) and morpholine (0.61 mL, 0.61 g, 6.95 mmol) in THF (183 mL) at 25 ºC for 30 min afforded, after purification by trituration with CH_2Cl_2 (2x), the desired compound (1.73 g, 83%) as a beige solid. **^{1}H NMR** (400 MHz, DMSO-d_6, 343 K) δ 8.47 (d, *J* = 8.4 Hz, 1H, ArH), 8.27 (d, *J* = 8.1 Hz, 1H, ArH), 8.12 (d, *J* = 7.6 Hz, 1H, NH), 7.99 (d, *J* = 7.7 Hz, 1H, ArH), 7.70 (d, *J* = 7.6 Hz, 1H, ArH), 7.66 – 7.41 (m, 2H, ArH), 7.36 – 7.20 (m, 7H, ArH), 7.16 (t, *J* = 7.6 Hz, 1H, ArH), 5.09 – 4.89 (m, 2H, OC$\underline{H_2}$Ph), 4.68 – 4.55 (m, 1H, $H_{2''}$), 4.23 (t, *J* = 7.0 Hz, 1H, $H_{2'}$), 3.24 (dd, *J* = 14.9, 5.1 Hz, 1H, $H_{3A''}$), 3.01 (dd, *J* = 14.9, 9.1 Hz, 1H, $H_{3B''}$), 2.50 (s, COC$\underline{H_3}$), 2.31 – 2.10 (m, 1H, C$\underline{H}$(CH_3)$_2$), 1.00 – 0.93 (m, 6H, CH$\underline{(CH_3)_2}$) ppm. **^{13}C{^{1}H} NMR** (101 MHz, DMSO-d_6, 343 K) δ 171.4 (s), 169.5 (s), 168.8 (s), 168.3 (s), 155.4 (s), 139.9 (s), 136.6 (s), 134.8 (s), 133.5 (d), 130.7 (d), 130.2 (s), 127.8 (d), 127.2 (d), 127.0 (d), 124.4 (d), 124.1 (d), 122.6 (d), 122.5 (d), 119.9 (d), 118.9 (d), 117.1 (s), 116.7 (s), 115.4 (d), 65.1 (t), 59.9 (d), 54.0 (d), 29.7 (d), 26.8 (t), 23.1 (q), 18.7 (q), 17.8 (q) ppm. **HRMS** (ESI-TOF) *m/z*: $[M + H]^+$ calcd. for $C_{33}H_{35}N_4O_7$, 599.2500; found, 599.2489. **IR** (neat): ν 3400-3100 (br, O-H, N-H), 2965 (w, C-H), 1690 (m, C=O), 1638 (s, C=O), 1597 (m, C=O), 1522 (s), 1446 (m), 1356 (m), 1338 (m), 1289 (m), 1224 (m), 1172 (m), 1138 (m), 1081 (m), 1024 (m), 745 (s), 696 (m), 639 (m) cm^{-1}. **$[\alpha]_D^{21}$** +12.0 (*c* 0.34, DMF).

Cbz deprotection of the acyclic intermediate

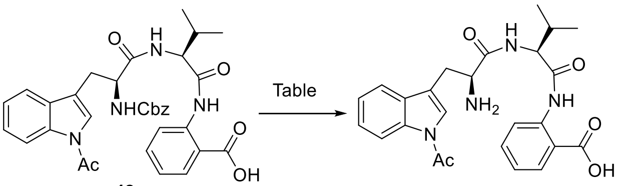

42 Insoluble in most organic solvents

43

Entry	Reaction conditions	Yield
**1**	$Pd(OH)_2$/C (20%), MeOH [0.01M], H_2 (balloon), r.t., 26 h	SM
**2**	$Pd(OH)_2$/C (20%), MeOH/THF (1:1) [0.0052M], H_2 (balloon), r.t., 4 days[14]	SM + decomposition
**3**	$Pd(OH)_2$/C (20%), MeOH/Et_3N (3:1) [0.035M], H_2 (balloon), r.t., 21 h[15]	decomposition + isomerization
**4**	$Pd(OH)_2$/C (20%), MeOH/Et_3N (23:1) [0.035M], H_2 (balloon), r.t., 8 h	1:1.2 SM/product
**5**	Pd black (20%), MeOH/Et_3N (4.5:1) [0.025M], H_2 (balloon), r.t. 19 h purification by filtration through a short pad of silica gel	**60%***
**6**	**Entry 2** with H_2 (25 bar), 17 h	Decomposition
**7**	**Entry 6** with MeOH as solvent	SM
**8**	Li, naphthalene, THF, 0 ºC, 3.5 h[16]	Decomposition

***** Slightly impure sample

Macrolactamization and bromocyclization

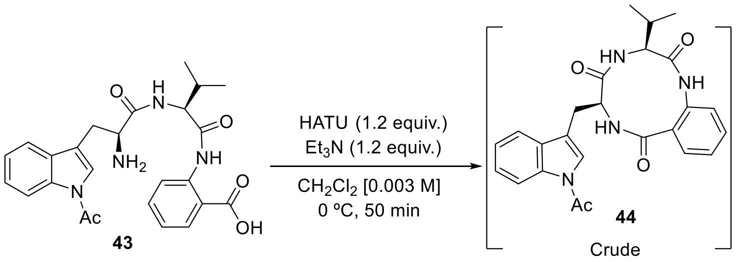

Entry	Reaction conditions	Yield
1	NBS, CH_2Cl_2, - 30 ºC, 50 min	Decomposition
2	NBS, CH_2Cl_2, - 30 ºC, 20 min	Decomposition
3	NBS, PPTS, CH_3CN, - 35 ºC, 5 min	Decomposition
4	NBS, CH_2Cl_2, - 50 ºC, 20 min<br>NO WORKUP for the macrolactamization, just addition of NBS at -50 ºC	Decomposition
5	NBS, CH_2Cl_2, - 50 ºC, 20 min<br>Normal WORKUP for the macrolactamization, but the solvent was not evaporated: the temperature was cooled down to -50 ºC and NBS was added	Decomposition

Bromo-precursor of the synthetic novofumigatamide from L-Trp (*exo*-46 and *endo*-46)

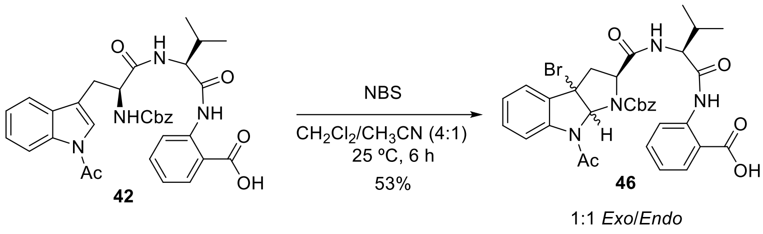

Following the general procedure described above for the bromocyclization with *N*-bromosuccinimide (Method A), the reaction of the acyclic intermediate **42** (0.18 g, 0.3 mmol) and NBS (54.0 mg, 0.3 mmol) in CH_2Cl_2/CH_3CN (15 mL, 4:1 *v/v*) at room temperature for 6 h afforded, after purification by flash column chromatography (silica gel, gradient from 95:5 to 90:10 *v/v* CH_2Cl_2/MeOH), 0.11 g (53% yield) of a 1:1 mixture of *exo*/*endo*-**46** isomers, which was used in the next step without separation of both diastereomer. A HPLC trace is shown in section "**4. Spectra collection and HPLC-MS traces**".

H-precursor of the synthetic novofumigatamide from L-Trp 47

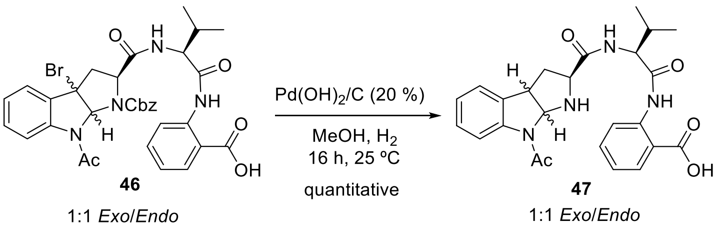

Following the general procedure described above for Cbz deprotection, the reaction of the *exo*/*endo* mixture of isomers of the Cbz-protected derivative **46** (0.05 g, 0.07 mmol), $Pd(OH)_2/C$ (0.02 g, 0.11 mmol) in MeOH (7 mL) under hydrogen atmosphere afforded, after 19 h of reaction

time, a mixture of *exo*/*endo* isomers as a beige solid (0.03 g, quantitative yield), which was used in the next step without separation of both diastereomers. A HPLC trace is shown in section "**4. Spectra collection and HPLC-MS traces**".

REMARK: the presence of the free carboxylic acid moiety makes the separation of both isomers in the last two reactions very challenging. Different conditions in normal and C18-reverse phase silica gel were attempted without success.

H-Synthetic novofumigatamide 48

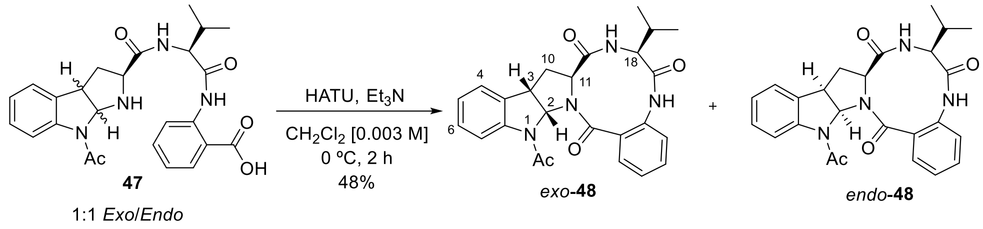

Following the general procedure described above for the macrolactamization, the reaction of the *exo*/*endo* mixture of isomers of the tryptophan derivative **47** (16.0 mg, 34.0 μmol), Et_3N (6.0 μL, 4.0 mg, 41.0 μmol) and HATU (16.0 mg, 41.0 μmol) in CH_2Cl_2 (11.5 mL) at 0 ºC for 1 h, followed by purification of the residue by flash column chromatography (silica gel, gradient from 50% hexane/EtOAc to 100% EtOAc), afforded 4.4 mg (29% yield) of the H-*exo*-**48** isomer and 2.9 mg (19% yield) of the H-*endo*-**48** isomer, both as white solids. Due to the low stability of the isomers and their rapid decomposition, a further purification by flash column chromatography (silica gel, 97:3 *v/v* CH_2Cl_2/MeOH) was required. The *endo* diastereomer could not be fully characterized due to its higher instability at the temperature required to observe a good resolution on the NMR spectra.

H-*Exo*-**48.** ^{1}**H NMR** (400 MHz, DMSO-d_6, 298 K) δ 8.54 (d, *J* = 8.0 Hz, NH), 8.13 (dd, *J* = 8.2, 1.5 Hz, 1H, ArH), 8.02 (d, *J* = 8.0 Hz, 1H, ArH), 7.95 (ddd, *J* = 8.3, 7.4, 1.6 Hz, 1H, ArH), 7.68 – 7.60 (m, 2H, ArH), 7.32 (d, *J* = 7.4 Hz, 1H, ArH), 7.18 (t, *J* = 7.7 Hz, 1H, ArH), 7.05 (t, *J* = 7.3 Hz, 1H, ArH), 5.87 (d, *J* = 7.4 Hz, 1H, H_2), 4.52 (app. t, *J* = 7.6 Hz, 1H, H_{18}), 4.01 (app. t, *J* = 7.8 Hz, 1H, H_3), 3.55 – 3.41 (m, 1H, H_{11}), 2.39 – 2.30 (m, 1H, H_{10A}), 2.27 (s, 3H, CO$\underline{CH_3}$), 2.25 – 2.12 (m, 1H, C$\underline{H}$$(CH_3)_2$), 2.02 – 1.90 (m, 1H, H_{10B}), 0.94 (d, *J* = 6.8 Hz, 3H, CH(C$\underline{H_3}$)$_2$), 0.93 (d, *J* = 6.8 Hz, 3H, CH(C$\underline{H_3}$)$_2$) ppm. ^{13}**C{^{1}H} NMR** (101 MHz, DMSO-d_6, 298 K) δ 172.2 (s), 169.7 (s), 160.9 (s), 158.8 (s), 145.4 (s), 142.9 (s), 137.0 (d), 132.6 (s), 128.9 (d), 128.0 (d), 127.6 (d), 126.6 (d), 124.8 (d), 123.4 (d), 116.7 (s), 115.3 (d), 80.0 (d), 58.6 (d), 57.5 (d), 45.8 (d), 39.8 (t), 30.2 (d), 23.9 (q), 19.1 (q), 18.2 (q) ppm. **HRMS** (ESI-TOF) *m/z*: $[M + H]^+$ calcd. for $C_{25}H_{27}N_4O_4$, 447.2027; found, 447.2025. $[\alpha]_D^{21}$ –68.1 (*c* 0.06, $CHCl_3$).

H-*Endo*-**48.** ^{1}**H NMR** (400 MHz, DMSO-d_6, 343 K) δ 8.10 (d, *J* = 7.7 Hz, 1H, ArH), 8.00 – 7.90 (m, 2H, ArH), 7.67 – 7.56 (m, 2H, ArH), 7.19 (d, *J* = 7.6 Hz, 1H, ArH), 7.10 (t, *J* = 7.7 Hz, 1H,

ArH), 6.94 (t, J = 7.6 Hz, 1H, ArH), 5.89 (d, J = 7.7 Hz, 1H, H_2), 4.19 (dd, J = 8.5, 5.5 Hz, 1H), 4.03 – 3.94 (m, 1H), 3.92 – 3.84 (m, 1H), 2.60 – 2.43 (m, 2H), 2.40 (s, 3H, $COCH_3$), 1.89 – 1.77 (m, 1H, $C\underline{H}(CH_3)_2$), 0.61 (d, J = 6.7 Hz, 3H, $CH(C\underline{H_3})_2$), 0.56 (d, J = 6.7 Hz, 3H, $CH(C\underline{H_3})_2$) ppm. **HRMS** (ESI-TOF) *m/z*: $[M + H]^+$ calcd. for $C_{25}H_{27}N_4O_4$, 447.2027; found, 447.2025. $[\alpha]_D^{22}$ –32.7 (c 0.03, $CHCl_3$).

<u>Preparation of synthetic (-)-novofumigatamide from L-Trp and L-Val following route B.2</u>

Methyl (*S*)-2-(*tert*-Butoxycarbonyl)amino-3-(indole-3'-yl)propanoate (*S*)-54

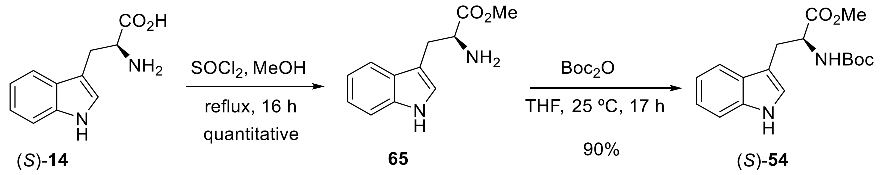

To a cooled (0 ºC) solution of thionyl chloride (3.4 mL, 5.52 g, 46.0 mmol,) in MeOH (40 mL) L-tryptophan (*S*)-**14** was added (4.0 g, 20.0 mmol). A reflux condenser was then fitted to the round-bottomed flask and the reaction mixture was stirred under refluxing conditions in a metal heating block for 16 h. The solvent was removed under reduced pressure to obtain the corresponding hydrochloride. The residue was neutralized with a saturated aqueous solution of $NaHCO_3$, and the aqueous layer was extracted with EtOAc (3x). The combined organic layers were dried (anhydrous Na_2SO_4), filtered, and the solvent evaporated to give 4.36 g (quantitative yield) of the titled compound as a brown foam.[17]

Di-*tert*-butyl dicarbonate (1.9 g, 9.12 mmol) was added to a solution of L-tryptophan methyl ester **65** (1.66 g, 7.59 mmol) in THF (19.0 mL) under argon atmosphere. The solution was stirred for 17 h at 25 ºC and the solvent was concentrated. The residue was purified by flash column chromatography (silica gel, 50:50 *v/v* hexane/EtOAc) to obtain 2.15 g (90% yield) of the titled compound (*S*)-**54** as a white solid. The spectroscopic data matched those previously reported.[18]

^{1}H-NMR (400 MHz, $CDCl_3$) δ 8.36 (br s, 1H, NH), 7.58 (d, J = 7.9 Hz, 1H, ArH), 7.34 (d, J = 8.0 Hz, 1H, ArH), 7.24 – 7.15 (m, 1H, ArH), 7.18 – 7.11 (m, 1H, ArH), 6.96 (s, 1H, ArH), 5.13 (d, J = 8.1 Hz, 1H, NH), 4.74-4.59 (m, 1H, H_2), 3.57 (s, 3H, $CO_2\underline{CH_3}$), 3.39-3.20 (m, 2H, $2H_3$), 1.44 (s, 9H, $CO_2\underline{^tBu}$) ppm. $[\alpha]_D^{20}$ +47.5 (c 0.05, $CHCl_3$).[18]

Methyl (*S*)-2-(*tert*-Butoxycarbonyl)amino-3-(1'-acetylindole-3'-yl)propanoate (*S*)-53

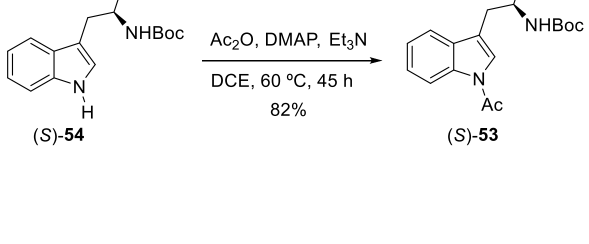

Following the general procedure described above for the acetylation of amines, the reaction of tryptophan derivative (*S*)-**54** (1.0 g, 3.14 mmol), 4-dimethylaminopyridine (DMAP) (0.08 g, 0.63 mmol), acetic anhydride (0.89 mL, 0.96 g, 9.42 mmol) and triethylamine (0.66 mL, 0.48 g, 4.71 mmol) in DCE (31.4 mL) at 60 ºC (metal heating block) for 45 h afforded, after purification by flash column chromatography (CombiFlash® Rf+ system, 80 g silica gel, gradient from 80:20 to 20:80 *v/v* hexane/EtOAc, flow rate = 60 mL/min), 0.93 g (82% yield) of the titled compound, as a white solid. The spectroscopic data matched those previously reported.[4]

^{1}H-NMR (400 MHz, $CDCl_3$, 323 K) δ 8.41 (d, *J* = 8.2 Hz, 1H), 7.50 (d, *J* = 7.7 Hz, 1H), 7.36 (ddd, *J* = 8.4, 7.2, 1.4 Hz, 1H), 7.31 – 7.24 (m, 2H), 5.09 (s, 1H, NH), 4.76 – 4.56 (m, 1H, H_2), 3.70 (s, 3H, $CO_2C\underline{H_3}$), 3.27 (dd, *J* = 14.9, 5.5 Hz, 1H, H_{3A}), 3.21 – 3.07 (m, 1H, H_{3B}), 2.60 (s, 3H, $CO\underline{CH_3}$), 1.42 (s, 9H, $CO_2{}^t\underline{Bu}$) ppm. $[\alpha]_D^{20}$ +272 (*c* 0.7, $CHCl_3$).[4]

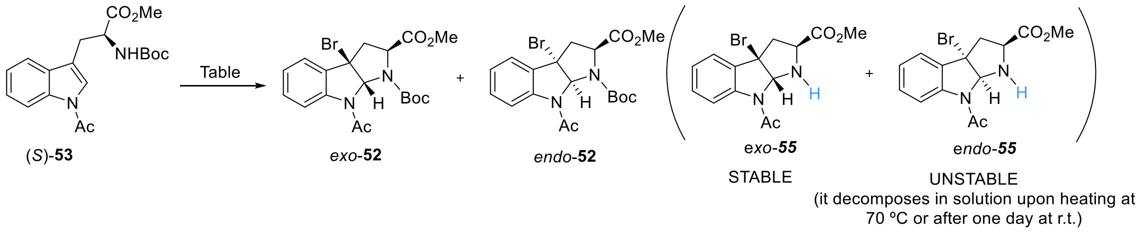

Entry	Conditions	Comments	Yield
**1**	NBS (1.1 equiv.)<br>CH_2Cl_2 [0.005M], r.t., 22 h<br>High dilution<br>(*ChemBioChem* **2019**, *20*, 1273)[19]	CH_2Cl_2 is not a suitable solvent for this reaction since its use leads to greater decomposition rates of the SM and/or the products	66%<br>(3:1 *exo*/endo)
**2**	NBS (1.1 equiv.), CH_3CN<br>r.t., 80 min	-	79% (2.4:1 *exo*/*endo*)<br>*exo* and *endo* products are difficult to separate
**3**	NBS (1.1 equiv.), PPTS (1.1equiv.)<br>CH_3CN, -35 ºC, 1 h	-	79% (5.2:1 *exo*/*endo*)
**4**	NBS (1.1 equiv.), $BF_3{\cdot}OEt_2$ (2 equiv.), CH_3CN, 0 ºC, 70 min<br>(*J. Am. Chem. Soc.* **2016**, *138*, 1057)[20]	-	78% deprotected products<br>(41% *endo* and 37% *exo*)<br>6% protected products<br>(2:1 *exo*/*endo*)
**5**	NBS (1.1 equiv.), $BF_3{\cdot}OEt_2$ (**4 equiv.**), CH_3CN, 0 ºC, 75 min<br>**As entry 4 but with 4 equiv. of $BF_3{\cdot}OEt_2$**	The use of a larger excess of $BF_3{\cdot}OEt_2$ is aimed at obtaining the deprotected products as the only products of the reaction. As expected, no protected products were obtained	74% deprotected products<br>(34% *endo* and 40% *exo*)
**6**	NBS (1.1 equiv.), $BF_3{\cdot}OEt_2$ (2 equiv.), CH_3CN, **-30 ºC**, 12 h<br>**As entry 4 but at -30 ºC to avoid Boc deprotection**<br>(*Org. Lett.* **2019**, *21*, 4205)[21]	The deprotected products were obtained as major compounds	47% deprotected products<br>(18% *endo* and 29% *exo*)<br>28% protected products<br>(2:1 *exo*/*endo*)

Methyl (2*S*,3a*S*,8a*S*)-8-Acetyl-3a-bromo-1-(*tert*-butoxycarbonyl)-(1,2,3,3a,8,8a)-hexahydropyrrolo[2,3-*b*]indole-2- carboxylate (*exo*-52) and Methyl (2*S*,3a*R*,8a*R*)-8-Acetyl-3a-bromo-1-(*tert*-butoxycarbonyl)-(1,2,3,3a,8,8a)-hexahydropyrrolo[2,3-*b*]indole-2-carboxylate (*endo*-52)

Following the general procedure described above for the bromocyclization with *N*-bromosuccinimide (Method A), the reaction of tryptophan derivative (*S*)-**53** (0.05 g, 0.14 mmol) and NBS (0.03 g, 0.15 mmol) in CH_3CN (1.4 mL) at 25 ºC for 80 min afforded, after purification by flash column chromatography (CombiFlash® Rf+ system, 12 g silica gel, gradient from 85:15 to 75:25 *v/v* hexane/EtOAc, flow rate = 30 mL/min), 0.05 (79% yield) of a 2.4:1 mixture of *exo*/*endo*-**52** isomers. These isomers were separated by HPLC (Waters Prep Nova-Pak HR Silica 60 Å, 6 µm, 19 x 300 mm, 90:10 *v/v* hexane/EtOAc, 5 mL/min).

Endo-**52**. **^{1}H NMR** (400 MHz, DMSO-d_6, 343 K) δ 7.78 (d, *J* = 8.1 Hz, 1H, ArH), 7.47 (ddd, *J* = 7.7, 1.4, 0.6 Hz, 1H, ArH), 7.37 – 7.30 (m, 1H, ArH), 7.13 (td, *J* = 7.5, 1.1 Hz, 1H, ArH), 6.28 (s, 1H, H_{8a}), 4.54 (dd, *J* = 9.1, 1.5 Hz, 1H, H_2), 3.32 – 3.15 (m, 2H, 2H_3), 3.10 (s, 3H, $CO_2C\underline{H_3}$), 2.54 (s, 3H, $COCH_3$), 1.37 (s, 9H, $C(CH_3)_3$) ppm. **^{13}C{^{1}H} NMR** (101 MHz, DMSO-d_6, 343 K) δ 169.8 (s), 169.6 (s), 152.3 (s), 141.5 (s), 132.5 (s), 130.2 (d), 124.4 (d), 123.7 (d), 118.4 (d), 84.7 (d), 80.6 (d), 61.4 (s), 59.4 (d), 51.4 (q), 41.2 (t), 27.6 (q), 23.1 (q) ppm. **HRMS** (ESI-TOF) *m/z*: $[M + H]^+$ calcd. for $C_{19}H_{24}{}^{79}BrN_2O_5$, 439.0863; found, 439.0864. **IR** (NaCl): ν 2976 (m, C-H), 2952 (m, C-H), 2933 (m, C-H), 1761 (s, C=O), 1712 (s, C=O), 1682 (s, C=O), 1476 (s), 1398 (s), 1370 (s), 1342 (s), 1283 (s) 1164 (s), 756 (s) cm^{-1}. $[\alpha]_D^{23}$ +68.4 (*c* 0.2, $CHCl_3$).

Exo-**52.**[22] **^{1}H NMR** (400 MHz, DMSO-d_6, 343 K) δ 7.82 (d, *J* = 8.1 Hz, 1H, ArH), 7.71 – 7.65 (m, 1H, ArH), 7.44 – 7.35 (m, 1H, ArH), 7.23 (td, *J* = 7.5, 1.1 Hz, 1H, ArH), 6.26 (s, 1H, H_{8a}), 3.81 (dd, *J* = 9.9, 6.8 Hz, 1H, H_2), 3.72 (s, 3H, $CO_2C\underline{H_3}$), 3.63 – 3.55 (m, 1H, H_{3A}), 2.70 (dd, *J* = 12.9, 9.9 Hz, 1H, H_{3B}), 2.52 (s, 3H, $COCH_3$), 1.30 (s, 9H, $C(CH_3)_3$) ppm. **^{13}C{^{1}H} NMR** (101 MHz, DMSO-d_6, 343 K) δ 170.7 (s), 169.5 (s), 151.4 (s), 140.4 (s), 132.5 (s), 130.4 (d), 124.9 (d), 123.7 (d), 118.3 (d), 84.0 (d), 81.2 (s), 60.5 (s), 59.1 (d), 52.0 (q), 39.7 (t), 27.4 (q), 23.3 (q) ppm. **HRMS** (ESI-TOF) *m/z*: $[M + H]^+$ calcd. for $C_{19}H_{24}{}^{79}BrN_2O_5$, 439.0863; found, 439.0869. **IR** (NaCl): ν 2979 (w, C-H), 2954 (w, C-H), 2933 (w, C-H), 1753 (m, C=O), 1714 (s, C=O), 1684 (s, C=O), 1476 (m), 1400 (m), 1368 (m), 1340 (m), 1322 (m), 1283 (m), 1167 (s), 754 (m) cm^{-1}. $[\alpha]_D^{22}$ –146.4 (*c* 0.2, $CHCl_3$).

Methyl (2*S*,3a*S*,8a*S*)-8-Acetyl-3a-bromo-(1,2,3,3a,8,8a)-hexahydropyrrolo[2,3-*b*]indole-2-carboxylate (*exo*-55) and Methyl (2*S*,3a*R*,8a*R*)-8-Acetyl-3a-bromo-(1,2,3,3a,8,8a)-hexahydropyrrolo[2,3-*b*]indole-2-carboxylate (*endo*-55)

General procedure for the bromocyclization with *N*-bromosuccinimide (Method C). To a stirred solution of the tryptophan derivative (*S*)-**53** (0.05 g, 0.14 mmol) in CH_3CN (6.5 mL) at 0

ºC, $BF_3 \cdot OEt_2$ (0.07 mL, 0.08 g, 0.56 mmol) was added slowly, and the reaction mixture was stirred vigorously at 0 ºC for 20 min. In a separate flask, a solution of NBS (0.03 g, 0.15 mmol) in CH_3CN (0.7 mL) was cooled down to 0 ºC and then slowly cannulated to the reaction mixture. After 45 min at 0 ºC, a saturated aqueous solution of $Na_2S_2O_3$ was added and the reaction mixture was allowed to reach to room temperature. EtOAc was added, the layers were separated, and the aqueous layer was extracted with CH_2Cl_2 (3 x). The combined organic layers were dried over anhydrous Na_2SO_4, and the solvent was removed under reduced pressure. The residue was purified by flash column chromatography (CombiFlash® Rf+ system, 24 g silica gel, gradient from 30:70 *v/v* hexane/EtOAc to 100% EtOAc, flow rate = 35 mL/min) to afford 18.8 mg (40% yield) of the *exo*-**55** isomer and 15.9 mg (34% yield) of the *endo*-**55** isomer, both as yellow oils (74% overall yield).

Exo-**55**. **^{1}H NMR** (400 MHz, DMSO-d_6, 308 K) δ 8.03 (d, J = 8.1 Hz, 1H, ArH), 7.58 (d, J = 7.8, 1H, ArH), 7.37 – 7.28 (m, 1H, ArH), 7.15 (td, J = 7.6, 1.1 Hz, 1H, ArH), 5.93 (s, 1H, H_{8a}), 4.35 (br s, NH), 3.66 (s, 3H, $CO_2C\underline{H_3}$), 3.42 (dd, J = 10.3, 5.5 Hz, 1H, H_2), 3.19 (dd, J = 12.6, 5.3 Hz, 1H, H_{3A}), 2.68 (dd, J = 12.3, 10.7 Hz, 1H, H_{3B}), 2.29 (s, 3H, $COCH_3$) ppm. **^{13}C{^{1}H} NMR** (101 MHz, DMSO-d_6, 308 K) δ 171.5 (s), 169.3 (s), 141.4 (s), 132.3 (s), 130.4 (d), 125.1 (d), 124.4 (d), 116.0 (d), 87.7 (d), 64.6 (s), 58.8 (d), 52.1 (q), 48.1 (t), 23.7 (q) ppm. **HRMS** (ESI-TOF) *m/z*: $[M + H]^+$ calcd. for $C_{14}H_{16}{}^{79}BrN_2O_3$, 339.0339; found, 339.0338. **IR** (NaCl): ν 3400-3200 (br, N-H), 2953 (w, C-H), 1740 (s, C=O), 1668 (s, C=O), 1479 (s), 1464 (m), 1437 (m), 1393 (s), 1357 (m), 1280 (m), 1251 (m), 1218 (m), 1135 (m), 758 (m) cm^{-1}. $[\alpha]_D^{25}$ –87.8 (*c* 0.12, $CHCl_3$).

Endo-**55**. **^{1}H NMR** (400 MHz, DMSO-d_6, 298 K) δ 7.97 (d, J = 8.0 Hz, 1H), 7.47 (dd, J = 7.6, 1.4 Hz, 1H), 7.32-7.23 (m, 1H), 7.09 (td, J = 7.6, 1.2 Hz, 1H), 5.87 (s, 1H, H_{8a}), 4.08 (dd, J = 8.3, 2.9 Hz, 1H, H_2), 3.15 (s, 3H, $CO_2C\underline{H_3}$), 3.12 (dd, J = 13.2, 3.0 Hz, 1H, H_{3A}), 2.98 (dd, J = 13.2, 8.3 Hz, 1H, H_{3B}), 2.31 (s, 3H, $COCH_3$) ppm. **^{13}C{^{1}H} NMR** (101 MHz, DMSO-d_6, 298 K) δ 172.3 (s), 169.2 (s), 141.3 (s), 132.8 (s), 130.3 (d), 125.0 (d), 124.0 (d), 116.1 (d), 88.2 (d), 64.9 (s), 59.3 (d), 51.5 (q), 46.9 (t), 23.9 (q) ppm. **HRMS** (ESI-TOF) *m/z*: $[M + H]^+$ calcd. for $C_{14}H_{16}{}^{79}BrN_2O_3$, 339.0339; found, 339.0342. **IR** (NaCl): ν 3400-3100 (br, N-H), 3006 (w, C-H), 2950 (w, C-H), 1738 (s, C=O), 1716 (s, C=O), 1666 (s), 1600 (m), 1479 (s), 1464 (m), 1393 (s), 1286 (m), 1215 (m), 1172 (m), 1133 (m), 758 (m) cm^{-1}. $[\alpha]_D^{24}$ +49.0 (*c* 0.14, $CHCl_3$).

Methyl (2*S*,3a*S*,8a*S*)-8-Acetyl-3a-(3'-methylbut-1-en-3'-yl)-1-(*tert*-butoxycarbonyl)-(1,2,3,3a,8,8a)-hexahydropyrrolo[2,3-*b*]indole-2- carboxylate ((*R*)-*exo*-51)

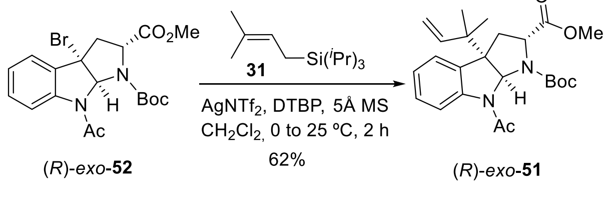

Following the general procedure described above for the reverse prenylation with triisopropyl(3-methyl-2-butenyl)silane (Method B), the reaction of *exo*-bromopyrroloindoline (*R*)-*exo*-**52** (0.25 g, 0.57 mmol), prenyl triisopropyl silane **31** (0.19 g, 0.85 mmol), freshly activated 5Å MS (3.22 g), DTBP (0.19 mL, 0.16 g, 0.85 mmol) and $AgNTf_2$ (0.32 g, 0.74 mmol) in CH_2Cl_2 (11.4 mL) at room temperature for 2 h afforded, after purification by flash column chromatography (CombiFlash® Rf+ system, 40 g silica gel, gradient from 90:10 to 80:20 *v/v* hexane/EtOAc, flow rate = 40 mL/min), the desired compound (*R*)-*exo*-**51** (0.15 g, 62% yield) as a white solid. **^{1}H NMR** (400 MHz, DMSO-d_6, 343 K) δ 7.76 (s, 1H, ArH), 7.44 – 7.37 (m, 1H, ArH), 7.30 (td, *J* = 7.9, 1.2 Hz, 1H, ArH), 7.15 (td, *J* = 7.5, 1.0 Hz, 1H, ArH), 5.94 – 5.76 (m, 2H, H_{8a} + $C(CH_3)_2C\underline{H}=CH_2$), 5.14 – 4.98 (m, 2H, $C(CH_3)_2CH=C\underline{H_2}$), 3.75 – 3.58 (m, 4H, H_2 + $CO_2C\underline{H_3}$), 2.62 (dd, *J* = 12.8, 7.0 Hz, 1H, H_{3A}), 2.44 (s, 3H, $COC\underline{H_3}$), 2.24 (dd, *J* = 12.8, 10.0 Hz, 1H, H_{3B}), 1.29 (s, 9H, $C(C\underline{H_3})_3$), 1.04 (s, 3H, $C(C\underline{H_3})_2CH=CH_2$), 0.85 (s, 3H, $C(C\underline{H_3})_2CH=CH_2$) ppm. **^{13}C{^{1}H} NMR** (101 MHz, DMSO-d_6, 343 K) δ 172.0 (s), 168.9 (s), 151.9 (s), 143.2 (d), 141.9 (s), 133.1 (s), 128.2 (d), 124.7 (d), 123.7 (d), 118.0 (d), 113.7 (t), 80.4 (s), 79.3 (d), 60.9 (s), 59.0 (d), 51.7 (q), 39.7 (s), 33.5 (t), 27.4 (q, 3 x C), 23.3 (q), 22.6 (q), 21.7 (q) ppm. **HRMS** (ESI-TOF) *m/z*: $[M + H]^+$ calcd. for $C_{24}H_{33}N_2O_5$, 429.2384; found, 429.2396. **IR** (NaCl): ν 2978 (w, C-H), 1753 (m, C=O), 1712 (s, C=O), 1674 (m, C=O), 1478 (m), 1402 (m), 1367 (m), 1342 (m), 1284 (m), 1201 (m), 1172 (m), 756 (m) cm^{-1}. $[\alpha]_D^{20}$ +107.3 (*c* 0.13, $CHCl_3$).

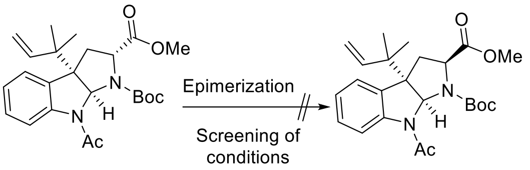

(R)-exo-**51** *(S)-endo*-**51**

Entry	Conditions	Comments	Yield
**1**	1. Formation **LiHMDS (2 equiv.)**, THF, 0 ºC, 30 min<br>2. Addition to the substrate, THF, -15 ºC<br>15 min at -15 ºC and 1 h at 0 ºC<br>3. MeOH **(1.2 equiv.)**, -78 ºC, 10 min.	Light yellow solution during the formation of the enolate	Only *exo* product (traces of *endo* product)
**2**	1. Formation **LDA (2 equiv.)**, THF, 0 ºC, 30 min<br>2. Addition to the substrate, THF, -15 ºC<br>15 min at -15 ºC and 1 h at 0 ºC<br>3. MeOH **(1.2 equiv.)**, -78 ºC, **30 min.**	Light yellow solution during the formation of the enolate	Only *exo* product (traces of *endo* product)
**3**	1. Formation **LDA (1.4 equiv.)**, THF, 0 ºC, 30 min<br>2. Addition to the substrate, THF, -15 ºC<br>15 min at -15 ºC and 1 h at 0 ºC<br>3. MeOH **(200 equiv.)**, -78 ºC, 30 min.	Yellow solution during the formation of the enolate.	Only *exo* product (**66%** after column chromatography)
**4**	Repetition of **entry 3** in a larger scale and with freshly titrated *n*-BuLi	Light yellow solution during the formation of the enolate	Only *exo* product
**5**	1. Formation **LiHMDS (2 equiv.)**, THF, 0 ºC, 30 min<br>2. Addition to the substrate, THF, **-15 ºC**, **1 h**<br>3. MeOH **(200 equiv.)**, -78 ºC, 10 min.<br>**Repetition of entry 1 with 200 equiv. of MeOH**	As it became evident that the enolate was formed (intense yellow colour) the	Only *exo* product (traces of *endo* product)

		reaction was at -15 °C for 1 h	
**6**	1. Formation **LiHMDS (3 equiv.)**, THF, 0 °C, 30 min<br>2. Addition to the substrate, THF, -15 °C<br>15 min at -15 °C and 1 h at 0 °C<br>3. MeOH (1.2 equiv.), -78 °C, 30 min.<br>**Repetition of entry 1 with 3 equiv. of LiHMDS**	Light yellow solution during the formation of the enolate	Only *exo* product (traces of *endo* product)
**7**	1. Formation **LiHMDS (2 equiv.)**, THF, 0 °C, 30 min<br>2. Addition to the substrate, THF, -15 °C<br>**30 min at -15 °C and 1 h at rt**<br>3. MeOH **(100 equiv.)**, - 78 °C, 10 min.	Yellow solution during the formation of the enolate	Only *exo* product (**58%** after column chromatography)

Methyl (2*S*,3a*R*,8a*R*)-3a-Bromo-1,8-di(*tert*-butoxycarbonyl)-(1,2,3,3a,8,8a)-hexahydropyrrolo[2,3-*b*]indole-2-carboxylate (*endo*-58)

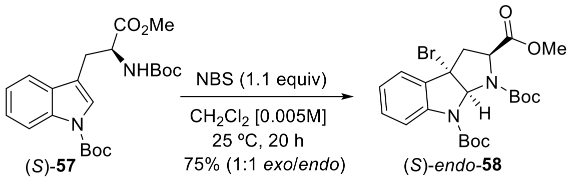

Following the general procedure described above for the bromocyclization with *N*-bromosuccinimide (Method A), the reaction of tryptophan derivative (*S*)-**57** (0.12 g, 0.28 mmol) and NBS (54.0 mg, 0.3 mmol) in CH_2Cl_2 (55.5 mL) at 25 °C for 20 h afforded, after purification by flash column chromatography (CombiFlash® Rf+ system, 24g silica gel, gradient from 95:5 to 90:10 *v/v* hexane/EtOAc, flow rate = 35 mL/min), 52.0 mg of the (*S*)-*exo*-**58** isomer and 52.0 mg of the (*S*)-*endo*-**58** isomer (75% overall yield), both as colourless oil. The spectroscopic data matched those previously reported.[19, 23]

^{1}H-NMR (400 MHz, $CDCl_3$) δ 7.55 (br, 1H, ArH), 7.32 – 7.24 (m, 2H), 7.04 (td, *J* = 7.5, 1.0 Hz, 1H, ArH), 6.44 (s, 1H, H_{8a}), 4.54 (d, *J* = 8.7 Hz, 1H, H_2), 3.27 (d, *J* = 13.0 Hz, 1H, H_{3A}), 3.12 (s, 3H, $CO_2C\underline{H_3}$), 3.07 (dd, *J* = 13.0, 9.3 Hz, 1H, H_{3B}), 1.60 (s, 9H, $C(C\underline{H_3})_3$), 1.46 (br s, 9H, $C(C\underline{H_3})_3$) ppm.

Methyl (2*S*,3a*R*,8a*R*)-3a-(3'-Methylbut-1-en-3'-yl)-1,8-di(*tert*-butoxycarbonyl)-(1,2,3,3a,8,8a)-hexahydropyrrolo[2,3-*b*]indole-2-carboxylate ((*S*)-*endo*-59)

<u>METHOD 1</u>

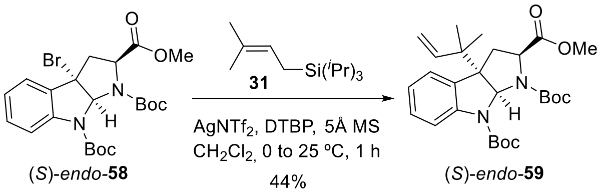

Following the general procedure described above for the reverse prenylation with triisopropyl(3-methyl-2-butenyl)silane (Method B), the reaction of *endo*-bromopyrroloindoline (*S*)-*endo*-**58** (0.04 g, 0.08 mmol), prenyl triisopropyl silane **31** (0.03 g, 0.12 mmol), freshly activated 5Å MS

(0.45 g), DTBP (0.03 mL, 0.02 g, 0.12 mmol) and $AgNTf_2$ (44.0 mg, 0.1 mmol) in CH_2Cl_2 (1.6 mL) at room temperature for 1 h afforded, after purification by flash column chromatography (CombiFlash® Rf+ system, 12 g silica gel, gradient from 95:5 to 80:20 *v/v* hexane/EtOAc, flow rate = 30 mL/min), the desired compound (*S*)-*endo*-**59** (0.02 g, 44% yield) as a white solid. The spectroscopic data matched those previously reported.[24]

^{1}H-NMR (400 MHz, $CDCl_3$) δ 7.41 (br, 1H, ArH), 7.20 (ddd, J = 8.2, 7.4, 1.4 Hz, 1H, ArH), 7.08 (dd, J = 7.7, 1.3 Hz, 1H, ArH), 6.95 (td, J = 7.5, 1.1 Hz, 1H, ArH), 6.15 (s, 1H, H_{8a}), 5.85 (dd, J = 17.3, 10.8 Hz, 1H, $C(CH_3)_2C\underline{H}=CH_2$), 5.13 – 4.90 (m, 2H, $C(CH_3)_2CH=C\underline{H_2}$), 4.49 (br, 1H, H_2), 3.08 (s, 3H, $CO_2C\underline{H_3}$), 2.55 (dd, J = 12.9, 9.0 Hz, 1H, H_{3A}), 2.47 (d, J = 12.8 Hz, 1H, H_{3B}), 1.55 (s, 9H, $C(C\underline{H_3})_3$), 1.46 (br s, 9H, $C(C\underline{H_3})_3$), 1.02 (s, 3H, $C(C\underline{H_3})_2CH=CH_2$), 0.91 (s, 3H, $C(C\underline{H_3})_2CH=CH_2$) ppm.

METHOD 2

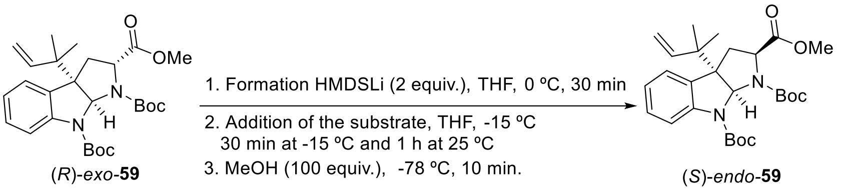

(*R*)-*exo*-**59** (*S*)-*endo*-**59**

To a solution of HMDS (52.0 μL, 0.04 g, 0.25 mmol) in THF (0.8 mL) at -78 ºC, *n*-BuLi (0.09 mL, 2.21 M in hexane, 0.21 mmol) was added and it was stirred for 30 min at 0 ºC. The hexahydropyrroloindole (*R*)-*exo*-**59**[24] (0.05 g, 0.10 mmol) was dissolved in THF (0.4 mL) and cooled down to – 15 ºC. The solution of lithium disilazide was added at this temperature and the reaction mixture was stirred for 30 min and then at room temperature for 1 h. The enolate was quenched by addition of MeOH (0.41 mL, 10.18 mmol) at -78 ºC. After stirring for 10 min, the reaction mixture was warmed to room temperature and diluted with EtOAc. A saturated aqueous solution of NH_4Cl was added and the aqueous layer was extracted with EtOAc (3 x). The combined organic layers were washed with a saturated aqueous solution of NaCl, dried over anhydrous Na_2SO_4 and the solvent evaporated. The residue was purified by flash column chromatography (CombiFlash® Rf+ system, 12 g silica gel, gradient from 95:5 to 90:10 *v/v* hexane/EtOAc, flow rate = 30 mL/min) to afford 31.3 mg (62% yield) of the titled compound as a colourless foam.

***N*-(2-Aminobenzoyl)-L-tryptophan Methyl Ester 66**

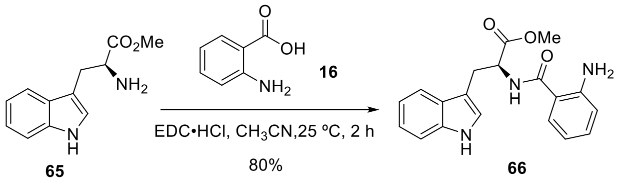

65 **66**

To a mixture of L-tryptophan methyl ester **65** (1.0 g, 4.58 mmol) and EDC·HCl (1.9 g, 9.9 mmol) in CH_3CN (46 mL) at room temperature, was added anthranilic acid **16** (1.24 g, 9.03 mmol) in 10 portions over a period of 100 min. After stirring for an additional 30 min, the solvent was evaporated under reduced pressure. The residue was dissolved in CH_2Cl_2/saturated aqueous solution of Na_2CO_3, the layers were separated, and the aqueous layer was extracted with CH_2Cl_2 (3 x). The combined organic layers were dried over anhydrous Na_2SO_4, and the solvent was evaporated. The residue was purified by flash column chromatography (silica gel, 98:2 *v/v* CH_2Cl_2/MeOH) to afford 1.23 g (80% yield) of the titled compound as a white solid. The spectroscopic data matched those previously reported.[25-26]

^{1}H-NMR (400 MHz, $CDCl_3$) δ 8.23 (br s, 1H, NH), 7.55 (d, *J* = 8.0 Hz, 1H, ArH), 7.38 – 7.33 (m, 1H, ArH), 7.28 – 7.16 (m, 3H, ArH), 7.10 (ddd, *J* = 8.0, 7.0, 1.0 Hz, 1H, ArH), 7.04 (d, *J* = 2.4 Hz, 1H, ArH), 6.93 (d, *J* = 8.3 Hz, 1H), 6.78 – 6.64 (m, 2H, ArH + NH), 5.08 (app. dt, *J* = 7.6, 5.4 Hz, 1H), 3.73 (s, 3H, $CO_2C\underline{H_3}$), 3.46 (dd, *J* = 14.8, 5.3 Hz, 1H, H_{3A}), 3.40 (dd, *J* = 14.8, 5.5 Hz, 1H, H_{3A}) ppm. $[\alpha]_D^{18}$ +72.7 (*c* 1.0, $CHCl_3$).[26]

Methyl (2*S*,2’’*S*)-2-{2’’-[2’’’-(*tert*-Butoxycarbonyl)amino-3’’’-methylbutanamido]benzoyl}amino-3-(indole-3’-yl)propanoate 64

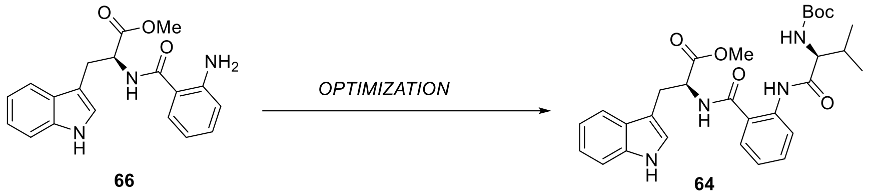

Entry	Conditions	Comments	Yield
**1**	*N*-Boc-L-Val (3.2 equiv.), DCC (3.2 equiv.), CH_2Cl_2/CH_3CN (4:1), r.t., 8 days	*N*-Boc-L-Val and DCC were added portionwise over 8 days. The reaction was followed by HPLC-MS (Scharlau analytic C18, 5 mm, flow = 1 mL/min, CH_3CN/H_2O from 30% to 100% in 20 min). After this reaction time some SM remained unreacted. After column chromatography the product could not be obtained pure.	ND
**2**	*N*-Boc-L-Val (2.0 equiv.), EDC•HCl (2.0 equiv.), CH_3CN, r.t., 5 days	The reaction was followed by HPLC-MS. After 5 days of reaction most of the SM was left	**~21%**
**3**	*N*-Boc-L-Val (1.3 equiv.), DCC (1.5 equiv.), HOBt (0.1 equiv.), CH_3CN, r.t., 8 days	The reaction was followed by HPLC-MS. An epimer of the product and the product with the incorporation of two valines were observed	Desired product: **28%** Epimer: **9%** Product with 2 valines also isolated (impure) in one fraction
**4**	**Repetition of entry 1**, 7 days of reaction time, 2 equiv. of *N*-Boc-L-Val and DCC: ~**0.3 equiv./day**	Its seems the amount of reagents added per day is important for the kinetics of the reaction: if all the equivalents are added at once, no full conversion of the SM is achieved	**92%**

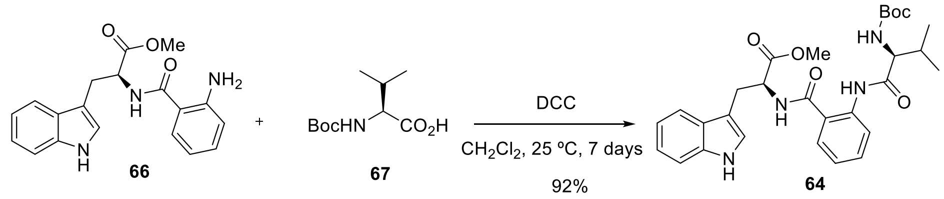

General procedure for amide (peptide) bond formation. Method C. To a solution of the tryptophan derivative **66** (0.5 g, 1.32 mmol) in CH_2Cl_2/CH_3CN (50 mL, 4:1 v/v) at room temperature, *N*-(*tert*-butoxycarbonyl)-L-valine **67** (0.64 g, 2.96 mmol) and DCC (0.61 g, 2.96 mmol) were added in portions over a period of 6 days (0.3 equiv./day). After the addition of the last portion, the mixture was stirred for a further 24 h, filtered through a pad of Celite®, and the solvent was concentrated. The residue was purified by flash column chromatography (CombiFlash® Rf+ system, 40 g silica gel, gradient from 70:30 to 60:40 *v/v* hexane/EtOAc, flow rate = 40 mL/min), to afford 0.73 g (92% yield) of the titled compound as a white solid. **^{1}H NMR** (400 MHz, DMSO-d_6, 343 K) δ 10.71 (s, 1H, NH), 8.84 (d, *J* = 7.3 Hz, 1H, NH), 8.45 (dd, *J* = 8.6, 1.0 Hz, 1H, ArH), 7.76 (dd, *J* = 7.8, 0.9 Hz,1H, ArH), 7.55 (d, *J* = 7.9 Hz, 1H, ArH), 7.49 (ddd, *J* = 8.6, 7.3, 1.5 Hz, 1H, ArH), 7.34 (dt, *J* = 8.1, 0.9 Hz, 1H, ArH), 7.18 (d, *J* = 2.3 Hz, 1H, ArH), 7.13 (td, *J* = 7.6, 1.2 Hz, 1H, ArH), 7.07 (ddd, *J* = 8.2, 7.0, 1.1 Hz, 1H, ArH), 6.99 (ddd, *J* = 7.9, 7.0, 1.1 Hz, 1H, ArH), 7.1-6.8 (br s, NH), 4.75 (ddd, *J* = 8.8, 7.4, 5.7 Hz, 1H, H_2), 3.85-3.71 (br m, 1H, $H_{2'''}$), 3.34 (dd, *J* = 14.8, 5.6 Hz, 1H, H_{3A}), 3.28 (dd, *J* = 14.8, 8.9 Hz, 1H, H_{3A}), 2.18-2.03 (m, 1H, $C\underline{H}(CH_3)_2$), 1.34 (s, 9H, $C(\underline{CH_3})_3$), 0.89 (d, *J* = 6.8 Hz, 6H, $CH(\underline{CH_3})_2$) ppm. **^{13}C{^{1}H} NMR** (101 MHz, DMSO-d_6, 343 K) δ 171.8 (s), 170.4 (s), 168.0 (s), 155.4 (s), 138.6 (s), 136.0 (s), 131.8 (d), 128.0 (d), 126.9 (s), 123.3 (d), 122.2 (d), 120.7 (d), 120.1 (d), 118.2 (d), 117.7 (d), 111.2 (d), 109.6 (s), 78.1 (s), 61.5 (d), 53.6 (d), 51.6 (q), 29.3 (d), 27.9 (q), 26.3 (t), 18.8 (q), 17.8 (q) ppm. **HRMS** (ESI-TOF) *m/z*: $[M + H]^+$ calcd. for $C_{29}H_{37}N_4O_6$, 537.2708; found, 537.2709. **IR** (NaCl): ν 3500-3100 (br, N-H), 3066 (w, C-H), 2969 (w, C-H), 2932 (w, C-H), 1691 (m, C=O), 1648 (m, C=O), 1587 (m), 1517 (s), 1444 (s), 1366 (m), 1167 (m), 753 (s) cm^{-1}. $[\alpha]_D^{25}$ +28.0 (*c* 0.15, $CHCl_3$).

REMARK: one ^{13}C{^{1}H} NMR signal is missing.

Methyl (2*S*,2’’*S*)-2-{2’’-[2’’’-(*tert*-Butoxycarbonyl)amino-3’’’-methylbutanamido]benzoyl}amino-3-(1’-acetylindole-3’-yl)propanoate 63

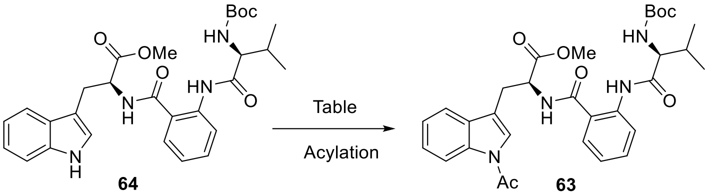

Entry	Conditions	Comments	Yield
**1**	Ac_2O (3 equiv.), Et_3N (3.0 equiv.) DMAP (0.6 equiv.), DCE, 60 ºC, 48 h	The reaction was followed by HPLC-MS (Scharlau analytic C18, 5 mm, flow = 1 mL/min, CH_3CN/H_2O from 30% to 100% in 20 min). The product and the bisacylated product were observed	**~37%**
**2**	Ac_2O (2.5 equiv.), Et_3N (2.5 equiv.) DMAP (1.5 equiv.), DCE, 60 ºC, 72 h Portionwise addition of the reagents	The reaction was followed by HPLC-MS. The product and the bisacylated product were observed	**49%**
**3**	Ac_2O (2.0 equiv.), Et_3N (2.0 equiv.) DMAP (0.5 equiv.), DCE, 50 ºC, 29 h	The reaction was followed by HPLC-MS. The product and the bisacylated product were observed	**43%**
**4**	Ac_2O (2.0 equiv.), Et_3N (2.0 equiv.) DMAP (0.5 equiv.), DCE, 50 ºC, 5 days As entry 3, 5 days reaction time	The reaction was followed by HPLC-MS. The product and the bisacylated product were observed	**52%**
**5**	Ac_2O (2.0 equiv.), Et_3N (2.0 equiv.) THF, 80 ºC, 48 h *(Org. Biomol. Chem.* **2015**, *13*, 8298)[27]	The reaction was followed by HPLC-MS	**33%**
**6**	NaOH, NBu_4HSO_4 AcCl, CH_2Cl_2, from 0 to 25 ºC, 2 h (*Org Lett* **2008,** *10*, 3841)[4]	Extensive decomposition during the reaction	**-**

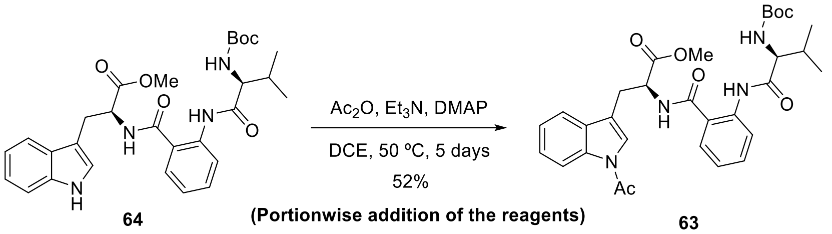

Following the general procedure described above for acetylation of amines, the reaction of the tryptophan derivative **64** (0.1 g, 0.19 mmol), 4-dimethylaminopyridine (DMAP) (11.0 mg, 0.09 mmol), acetic anhydride (35.0 µL, 0.04 g, 0.37 mmol) and triethylamine (0.05 mL, 0.04 g, 0.37 mmol) in DCE (2 mL) at 50 ºC (metal heating block) for 5 days afforded, after purification by flash column chromatography (CombiFlash® Rf+ system, 24 g silica gel, gradient from 70:30 to 60:40 *v/v* hexane/EtOAc, flow rate = 35 mL/min), 27.0 mg of an impure bis-acylated product (~23% yield) and 56.0 mg (52% yield) of the titled compound, both as white solids. **^{1}H NMR** (400 MHz, DMSO-d_6, 343 K) δ 8.96 (d, *J* = 7.5 Hz, 1H, NH), 8.43 (dd, *J* = 8.4, 1.2 Hz, 1H, ArH), 8.29 (d, *J* = 7.6 Hz, 1H, ArH), 7.77 (dd, *J* = 7.9, 1.5 Hz, 1H, ArH), 7.68 (s, 1H, ArH), 7.67 – 7.61 (m, 1H, ArH), 7.50 (ddd, *J* = 8.6, 7.4, 1.5 Hz, 1H, ArH), 7.32 (td, *J* = 7.7, 1.5 Hz, 1H, ArH), 7.27

(td, *J* = 7.4, 1.3 Hz, 1H, ArH), 7.15 (td, *J* = 7.6, 1.2 Hz, 1H, ArH), 7.0-6.8 (br s, 1H, NH), 4.81 (app. td, *J* = 8.2, 5.9 Hz, 1H, H_2), 3.82 – 3.72 (br m, 1H, $H_{2'''}$), 3.68 (s, 3H, $CO_2C\underline{H_3}$), 3.34 (dd, *J* = 14.8, 5.8 Hz, 1H, H_{3A}), 3.28 (dd, *J* = 14.8, 8.6 Hz, 1H, H_{3B}), 2.58 (s, 3H, $COCH_3$), 2.17 – 2.03 (m, 1H, $C\underline{H}(CH_3)_2$), 1.33 (s, 9H, $C(\underline{CH_3})_3$), 0.92-0-84 (m, 6H, $CH(C\underline{H_3})_2$) ppm. **$^{13}C\{^1H\}$ NMR** (101 MHz, DMSO-d_6, 343 K) δ 171.3 (s), 170.4 (s), 168.5 (s), 168.1 (s), 155.5 (s), 138.5 (s), 134.9 (s), 131.9 (d), 129.9 (s), 128.1 (d), 124.5 (d), 124.4 (d), 122.9 (d), 122.3 (d), 120.2 (d), 118.5 (d), 117.0 (s), 115.7 (d), 78.1 (s), 61.5 (d), 52.7 (d), 51.8 (q), 29.4 (d), 27.8 (q), 25.8 (t), 23.4 (q), 18.8 (q), 17.8 (q) ppm. **HRMS** (ESI-TOF) *m/z*: $[M + H]^+$ calcd. for $C_{31}H_{39}N_4O_7$, 579.2813; found, 579.2831. **IR** (NaCl): ν 3500-3200 (br, N-H), 3010 (w, C-H), 2969 (m, C-H), 2933 (w, C-H), 1699 (s, C=O), 1649 (m, C=O), 1587 (m), 1519 (s), 1450 (s), 1388 (m), 1329 (m), 1246 (m), 1224 (m), 1169 (m), 1016 (w), 753 (s) cm^{-1}. $[\alpha]_D^{24}$ +44.5 (*c* 0.18, $CHCl_3$).

REMARK: one $^{13}C\{^1H\}$ NMR signal is missing.

(2*S*,3a*R*,8a*R*,2’*S*)-8-Acetyl-3a-bromo-1-{2’-[2”-(*tert*-butoxycarbonyl)amino-3’’-methylbutanamido]benzoyl}-(1,2,3,3a,8,8a)-hexahydropyrrolo[2,3-*b*]indole-2-carboxylic Acid Methyl Ester (*exo*-62) and (2*S*,3a*S*,8a*S*,2’*S*)-8-Acetyl-3a-bromo-1-{2’-[2”-(*tert*-butoxycarbonyl)amino-3’’-methylbutanamido]benzoyl}-(1,2,3,3a,8,8a)-hexahydropyrrolo[2,3-*b*]indole-2-carboxylic Acid Methyl Ester (*endo*-62)

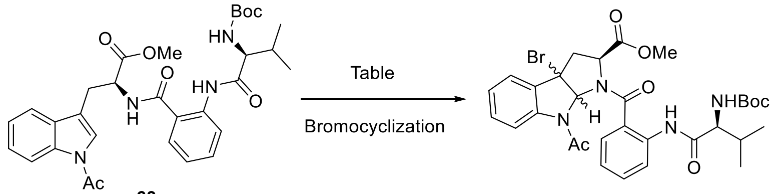

63 62

Entry	Conditions	Comments	Yield
**1**	NBS (1.2 equiv.), CH_3CN, -35 ºC, 15 h	The reaction was followed by HPLC-MS (Scharlau analytic C18, 5 mm, flow = 1 mL/min, CH_3CN/H_2O from 30% to 100% in 20 min).	**22%** *endo* **~44%** *exo* (in the ^1H NMR of the crude the *endo/exo* ratio could not be determined)
**2**	NBS (1.2 equiv.), CH_2Cl_2, -78 ºC, 20 h	Dichloromethane is not a good solvent for the substrates bearing an acetyl group, since it leads to more decomposition of the substrates and products. Nevertheless, CH_3CN has a melting point of -45 ºC, so it cannot be used for the reactions performed at -78 ºC	**34%** *exo* **(74% BRSM)**
**3**	NBS (1.2 equiv.), CH_3CN, 0 ºC, 3 h	The reaction was followed by HPLC-MS	**66%** (3:1 *exo*/*endo*)
**4**	NBS (1.2 equiv.), **toluene**, 0 ºC, 22 h (*Asian J. Org. Chem.* **2017**, *6*, 54)[8]	The reaction was followed by HPLC-MS	**~42%** *exo* **(74% BRSM)**
**5**	NBS (**1.7 equiv.**), CH_3CN, -35 ºC, 24 h	The reaction was followed by HPLC-MS	**60%** (1.7:1 *exo*/*endo*)

**6**	NBS (1.7 equiv.), **BF_3·OEt_2 (2 equiv.)**, CH_3CN, -35 ºC, 17 h	The reaction was followed by HPLC-MS	**47%** (1:1.4 *exo*/*endo*)
**7**	NBS (1.7 equiv.), CH_3CN, -35 ºC, **41 h** **As entry 5 but with 41 h reaction time**	The reaction was followed by HPLC-MS	**66%** (1.4:1 *exo*/*endo*)
**8**	NBS (**2.0 equiv.**), CH_3CN, -35 ºC, **72 h** **Reaction in a 0.5 g scale**	The reaction was followed by HPLC-MS	**70%** (2:1 *exo*/*endo*)

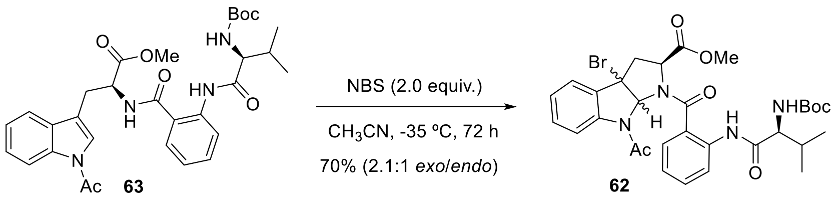

Following the general procedure described above for the bromocyclization with *N*-bromosuccinimide (Method A), the reaction of the tryptophan derivative **63** (0.59 g, 1.03 mmol) with NBS (0.38 g, 2.12 mmol) in CH_3CN (103 mL) at -35 ºC for 72 h afforded, after purification by flash column chromatography (CombiFlash® Rf+ system, 40 g silica gel, gradient from 70:30 to 60:40 *v/v* hexane/EtOAc, flow rate = 40 mL/min), 0.32 g (48% yield) of the *exo*-**62** isomer and 0.15 g (22% yield) of the *endo*-**62** isomer, both as white solids.

Exo-**62.** **^{1}H NMR** (400 MHz, DMSO-d_6, 343 K) δ 9.31 (br s, 1H, NH), 8.17 (d, *J* = 7.7 Hz, 1H, ArH), 7.75 (d, *J* = 7.5 Hz, 1H, ArH), 7.80-7.60 (br, 1H, ArH), 7.54 – 7.37 (m, 2H, ArH), 7.30 (td, *J* = 7.5, 0.8 Hz, 1H, ArH), 7.20– 6.85 (m, 3H, 2 x ArH + NH), 6.48 (s, 1H, H_{8a}), 4.19 (dd, *J* = 10.0, 6.8 Hz, 1H, H_2), 3.90 (app. t, *J* = 7.2 Hz, 1H, $H_{2''}$), 3.70 (dd, *J* = 12.8, 6.7 Hz, 1H, H_{3A}), 3.60-3.40 (br s, 3H, $CO_2C\underline{H_3}$), 2.80 (dd, *J* = 12.9, 10.4 Hz, 1H, H_{3B}), 2.40 – 1.80 (m, 4H, $COCH_3$ + $C\underline{H}(CH_3)_2$), 1.43 (s, 9H, $C(\underline{CH_3})_3$), 0.94 (d, *J* = 6.8 Hz, 6H, $CH(C\underline{H_3})_2$) ppm. **^{13}C{^{1}H} NMR** (101 MHz, DMSO-d_6, 343 K) δ 170.6 (s), 170.1 (s), 169.9 (s), 168.7 (s), 155.6 (s), 140.4 (s), 135.5 (s), 132.6 (s), 131.0 (d), 130.5 (d), 127.4 (d), 125.5 (d), 123.7 (d), 123.4 (d), 121.4 (d), 119.1 (d), 84.1 (d), 78.2 (s), 61.0 (d), 60.2 (s, HMBC), 60.2 (d), 52.3 (q), 39.7 (t), 29.4 (d), 28.0 (q), 21.7 (q), 18.9 (q), 18.0 (q) ppm. **HRMS** (ESI-TOF) *m/z*: $[M + H]^+$ calcd. for $C_{31}H_{38}{}^{79}BrN_4O_7$, 657.1918; found, 657.1917. **IR** (NaCl): ν 3500-3300 (br, N-H), 3009 (w, C-H), 2969 (w, C-H), 2933 (w, C-H), 1740 (m, C=O), 1691 (s, C=O), 1652 (m, C=O), 1585 (w), 1514 (m), 1452 (m), 1394 (m), 1369 (m), 1319 (m), 1285 (m), 1161 (s), 755 (m) cm^{-1}. $[\alpha]_D^{25}$ –107.3 (*c* 0.20, $CHCl_3$).

REMARK: due to the low resolution of the spectra, the integration of the ^{1}H NMR spectrum is not accurate and some ^{13}C{^{1}H} NMR signals are missing.

Endo-**62.** **^{1}H NMR** (400 MHz, DMSO-d_6, 343 K) δ 9.80 (br s, 1H, NH), 7.86 (d, *J* = 8.2 Hz, 1H, ArH), 7.52 (dd, *J* = 7.7, 0.8 Hz, 1H, ArH), 7.45 (ddd, *J* = 8.1, 7.3, 1.7 Hz, 1H, ArH), 7.38 (ddd, *J*

= 8.3, 7.5, 1.3 Hz, 1H, ArH), 7.31 (d, J = 8.0 Hz, 1H, ArH), 7.24 (dd, J = 7.7, 1.4 Hz, 1H, ArH), 7.20 – 7.13 (m, 2H, ArH), 6.55 (s, 1H, H_{8a}), 6.60-6.40 (br s, 1H, NH), 4.65 (d, J = 8.3 Hz, 1H, H_2), 3.96 (app. t, J = 7.3 Hz, 1H, $H_{2''}$), 3.48 – 3.31 (m, 2H, $2H_3$), 3.11 (s, 3H, $CO_2C\underline{H_3}$), 2.42 (s, 3H, $COC\underline{H_3}$), 2.15 – 2.02 (m, 1H, $C\underline{H}(CH_3)_2$), 1.44 (s, 9H, $C(\underline{CH_3})_3$), 0.99 (d, J = 6.8 Hz, 3H, $CH(C\underline{H_3})_2$), 0.96 (d, J = 6.8 Hz, 3H, $CH(C\underline{H_3})_2$) ppm. **$^{13}C\{^1H\}$ NMR** (101 MHz, DMSO-d_6, **343 K**) δ 170.9 (s), 169.6 (s), 169.4 (s), 155.2 (s), 142.0 (s), 134.1 (s), 132.1 (s), 130.4 (d, 2x), 129.5 (s), 126.5 (d), 124.8 (d), 124.6 (d, 2x), 124.1 (d), 118.2 (d), 85.0 (d), 78.2 (s), 61.8 (s), 61.6 (d), 60.4 (d), 51.7 (q), 43.1 (t), 30.3 (d), 28.0 (q), 22.6 (q), 18.8 (q), 18.0 (q) ppm. **HRMS** (ESI-TOF) *m/z*: $[M + H]^+$ calcd. for $C_{31}H_{38}{}^{79}BrN_4O_7$, 657.1918; found, 657.1932. **IR** (NaCl): ν 3400-3100 (br, N-H), 3007 (w, C-H), 2969 (w, C-H), 2932 (w, C-H), 1741 (m, C=O), 1715 (m, C=O), 1680 (s, C=O), 1602 (w), 1479 (m), 1392 (m), 1368 (m), 1167 (m), 1083 (w), 754 (m) cm^{-1}. $[\alpha]_D^{24}$ +60 (c 0.18, $CHCl_3$).

REMARK: the NMR spectra show unidentified aromatic signals, which could not be removed after several purifications by column chromatography.

(2*S*,3a*S*,8a*S*,2'*S*)-8-Acetyl-1-{2'-[2"-(*tert*-butoxycarbonyl)amino-3"-methylbutanamido]benzoyl}-3a-(3'''-methylbut-1-en-3'''-yl)-(1,2,3,3a,8,8a)-hexahydropyrrolo[2,3-*b*]indole-2-carboxylic Acid Methyl Ester (*endo*-49)

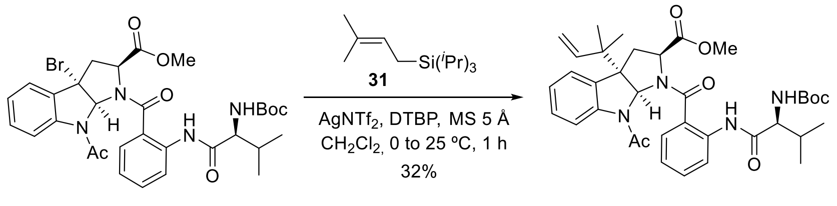

Following the general procedure described above for the reverse prenylation with triisopropyl(3-methyl-2-butenyl)silane (Method B), the reaction of *endo*-bromopyrroloindoline *endo*-**62** (0.13 g, 0.2 mmol), prenyl tri*iso*propyl silane **31** (0.07 g, 0.29 mmol), freshly activated 5Å MS (1.13 g), DTBP (66.0 µL, 0.06 g, 0.29 mmol) and $AgNTf_2$ (0.13 g, 0.29 mmol) in CH_2Cl_2 (3.9 mL) at room temperature for 1 h afforded, after purification by flash column chromatography (silica gel, 60:40 *v/v* hexane/EtOAc), 40.3 mg (32% yield) of the desired compound *endo*-**49** as a white solid. **^1H NMR** (400 MHz, DMSO-d_6, 343 K) δ 9.85 (s, 1H, NH), 7.84 (d, J = 7.8 Hz, 1H, ArH), 7.46 – 7.38 (m, 1H, ArH), 7.37 – 7.21 (m, 4H, ArH), 7.18 – 7.00 (m, 2H, ArH), 6.31 (s, 0.8H, H_2), 6.28 (s, 0.2H, H_2), 5.87 (dd, J = 17.1, 11.0 Hz, 1H, $C(CH_3)_2C\underline{H}=CH_2$), 5.12 – 4.82 (m, 2H, $C(CH_3)_2CH=C\underline{H_2}$), 4.62 – 4.48 (m, 1H, H_2), 4.04 – 3.90 (m, 1H, $H_{2''}$), 3.20-3.12 (m, 1H, H_{3A}), 3.05 (s, $CO_2C\underline{H_3}$), 2.94 (dd, J = 12.7, 8.9 Hz, 1H, H_{3B}), 2.55-2.35 (br s, 1H, $COCH_3$), 2.14 – 2.04 (m, 1H, $C\underline{H}(CH_3)_2$), 1.44 (s, 9H, $C(\underline{CH_3})_3$), 1.07 (s, 3H, $C(C\underline{H_3})_2CH=CH_2$), 1.00 (s, 3H, $C(C\underline{H_3})_2CH=CH_2$), 0.97 (d, J = 6.9 Hz, 3H, $CH(C\underline{H_3})_2$), 0.93 (d, J = 6.8 Hz, 3H, $CH(C\underline{H_3})_2$) ppm. **HRMS** (ESI-TOF) *m/z*: $[M + H]^+$ calcd. for $C_{36}H_{47}N_4O_7$, 647.3439; found, 647.3447. **IR** (NaCl):

ν 3500-3100 (br, N-H), 2968 (m, C-H), 2936 (m, C-H), 2871 (w, C-H), 1718 (s, C=O), 1677 (s, C=O), 1602 (m), 1483 (s), 1394 (s), 1370 (s), 1337 (m), 1232 (m), 1164 (s), 1085 (w), 1022 (m), 755 (s) cm^{-1}. $[\alpha]_D^{22}$ +32.0 (*c* 0.41, $CHCl_3$).

REMARK: due to the small amount of product available for the next steps of the synthetic sequence, it was not further characterized.

(2*S*,3a*S*,8a*S*,2'*S*)-8-Acetyl-1-{2'-[2"-(*tert*-butoxycarbonyl)amino-3''-methylbutanamido]benzoyl}-3a-(3'''-methylbut-1-en-3'''-yl)-(1,2,3,3a,8,8a)-hexahydropyrrolo[2,3-*b*]indole-2-carboxylic Acid (*endo*-68)

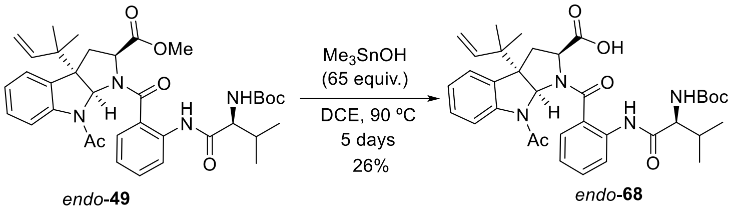

Following the general procedure described above for the hydrolysis of methyl esters (Method B), the reaction of *endo*-**49** (0.02 g, 0.03 mmol) with trimethyltin hydroxide (0.36 g, 2.02 mmol) in DCE (0.5 mL) at 90 ºC (metal heating block) for 5 days afforded, after purification by flash column chromatography (silica gel, gradient from 97.5:2.5 to 95:5 *v/v* CH_2Cl_2/MeOH), 5.2 mg (26% yield) of the titled compound as a white solid.

REMARK: The reaction was followed by HPLC-MS. The starting material is very unreactive under these reaction conditions. It seems the reagent Me_3SnOH poisons the reaction, so after addition of 20-30 equivalents a workup was performed, and the reaction was set up again with the crude mixture. This process was repeated several times.

(2*S*,3a*S*,8a*S*,2'*S*)-8-Acetyl-1-[2'-(2"-amino-3''-methylbutanamido)benzoyl]-3a-(3'''-methylbut-1-en-3'''-yl)-(1,2,3,3a,8,8a)-hexahydropyrrolo[2,3-*b*]indole-2-carboxylic Acid (*endo*-69)

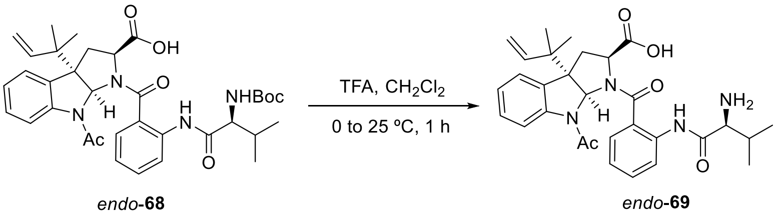

Following the general procedure described above for the deprotection of the *N*-Boc protected amines, the reaction of the *endo* carboxylic acid *endo*-**68** (6.5 mg, 10.3 μmol), TFA (0.16 mL, 0.24 g, 2.13 mmol) in CH_2Cl_2 (1.1 mL) at room temperature for 1 h afforded a residue that was used in the next step without further purification.

(2*S*,3a*R*,8a*R*,2'*S*)-8-Acetyl-1-{2'-[2"-(*tert*-butoxycarbonyl)amino-3''-methylbutanamido]benzoyl}-3a-(3'''-methylbut-1-en-3'''-yl)-(1,2,3,3a,8,8a)-hexahydropyrrolo[2,3-*b*]indole-2-carboxylic Acid Methyl Ester (*exo*-49)

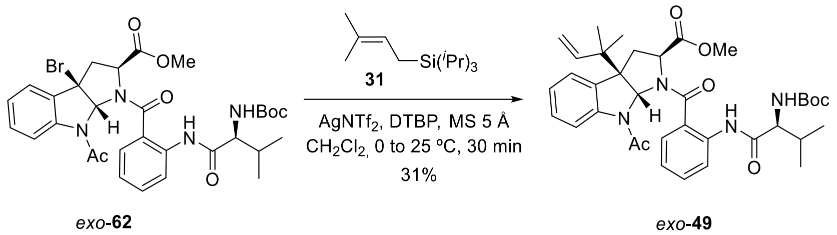

Following the general procedure described above for the reverse prenylation with triisopropyl(3-methyl-2-butenyl)silane (Method B), the reaction of *exo*-bromopyrroloindoline *exo*-**62** (0.15 g, 0.23 mmol), prenyl triisopropyl silane (0.08 g, 0.34 mmol), freshly activated 5Å MS (1.3 g), DTBP (77.0 μL, 65.0 mg, 0.34 mmol) and $AgNTf_2$ (0.15 g, 0.34 mmol) in CH_2Cl_2 (4.6 mL) at room temperature for 30 min afforded, after purification by flash column chromatography (silica gel, gradient from 70:30 to 60:40 *v/v* hexane/EtOAc), 45.0 mg of the desired compound *exo*-**49** (31% yield) as a white solid.

REMARK: due to the small amount of product available for the next steps of the synthetic sequence, it was not further characterized.

(2*S*,3a*R*,8a*R*,2'*S*)-8a-Acetyl-1-{2'-[2"-(*tert*-butoxycarbonyl)amino-3''-methylbutanamido]benzoyl}-3a-(3'''-methylbut-1-en-3'''-yl)-(1,2,3,3a,8,8a)-hexahydropyrrolo[2,3-*b*]indole-2-carboxylic Acid (*exo*-68)

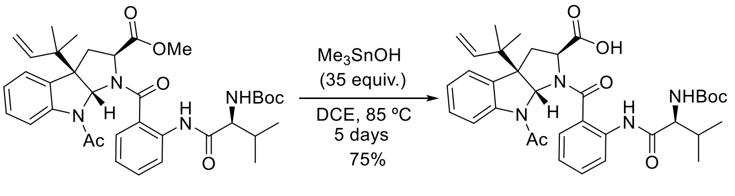

Following the general procedure described above for the hydrolysis of methyl esters (Method B), the reaction of *exo*-**49** (45.0 mg, 0.07 mmol) with trimethyltin hydroxide (0.44 g, 2.45 mmol) in DCE (0.8 mL) at 85 ºC (metal heating block) for 5 days afforded, after purification by flash column chromatography (silica gel, gradient from 97.5:2.5 to 95:5 *v/v* CH_2Cl_2/MeOH), 33.2 mg (75% yield) of the titled compound as a white solid. **^{1}H NMR** (400 MHz, DMSO-d_6, 383 K) δ 8.21 (d, J = 8.3 Hz, 1H, ArH), 7.72 – 7.56 (m, 1H, ArH), 7.45 (dd, J = 7.5, 0.7 Hz, 1H, ArH), 7.41 – 7.31 (m, 2H, ArH), 7.22 (td, J = 7.5, 1.0 Hz, 1H, ArH), 7.05 (t, J = 7.1 Hz, 1H, ArH), 6.94 (br s, 1H, ArH), 6.48 (br s, 1H, NH), 6.07 (br s, 1H, H_{8a}), 5.79 (dd, J = 17.3, 10.8 Hz, 1H, $C(CH_3)_2C\underline{H}{=}CH_2$), 5.09 – 4.87 (m, 2H, $C(CH_3)_2CH{=}C\underline{H_2}$), 4.07 – 3.98 (m, 1H, $H_{2''}$), 3.94 (dd, J = 9.9, 7.3 Hz, 1H, H_2), 2.72 (dd, J = 12.8, 7.2 Hz, 1H, H_{3A}), 2.30 (dd, J = 12.9, 10.0 Hz, 1H, H_{3B}), 2.23 – 2.10 (m, 1H, $C\underline{H}(CH_3)_2$), 1.43 (s, 9H, $C(\underline{CH_3})_3$), 1.04 (s, 3H, $C(\underline{CH_3})_2CH{=}CH_2$), 0.96 (d, J

= 6.8 Hz, 3H, CH($\underline{CH_3}$)$_2$), 0.93 (d, *J* = 6.8 Hz, 3H, CH($\underline{CH_3}$)$_2$), 0.88 (s, 3H, C($\underline{CH_3}$)$_2$CH=CH$_2$) ppm. **^{13}C{^{1}H} NMR** (101 MHz, DMSO-*d*$_6$, 383 K) δ 172.6 (s), 169.9 (s), 168.9 (s), 167.8 (s), 154.9 (s), 142.9 (d), 141.7 (s), 135.8 (s), 133.6 (s), 130.8 (d), 130.1 (d), 128.0 (d), 126.9 (d), 125.3 (s), 124.5 (d), 123.9 (d), 122.8 (d), 120.8 (d), 118.2 (d), 113.2 (t), 79.3 (d), 77.9 (s), 61.0 (s), 60.3 (d, 2x), 39.3 (s, HMBC), 33.5 (t), 29.9 (d), 27.7 (q, 3x), 22.4 (q), 21.9 (q), 18.7 (q), 17.3 (q) ppm. **HRMS** (ESI-TOF) *m/z*: [M + H]$^+$ calcd. for $C_{35}H_{45}N_4O_7$, 633.3282; found, 633.3282. **IR** (NaCl): ν 3600-3300 (br, N-H), 2970 (m, C-H), 2930 (m, C-H), 1718 (s, C=O), 1688 (s, C=O), 1647 (s, C=O), 1588 (m), 1507 (m), 1478 (m), 1456 (m), 1395 (s), 1370 (s), 1322 (m), 1286 (m), 1167 (s), 756 (s) cm^{-1}. $[\alpha]_D^{22}$ –68.8 (*c* 0.28, $CHCl_3$).

REMARK: the ^{1}H NMR and ^{13}C{^{1}H} NMR signals corresponding to the CO$\underline{CH_3}$ group are missing.

(2*S*,3a*R*,8a*R*,2'*S*)-8a-Acetyl-1-[2'-(2"-amino-3''-methylbutanamido)benzoyl]-3a-(3'''-methylbut-1-en-3'''-yl)-(1,2,3,3a,8,8a)-hexahydropyrrolo[2,3-*b*]indole-2-carboxylic Acid (*exo*-69)

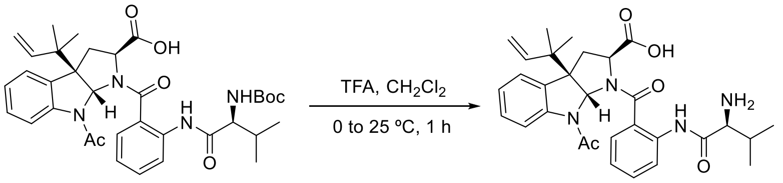

exo-**68** *exo*-**69**

Following the general procedure described above for the deprotection of the *N*-Boc protected amines, the reaction of the *exo*-**68** (17.3 mg, 27.3 μmol) and TFA (0.44 mL, 0.65 g, 5.68 mmol) in CH_2Cl_2 (3 mL) at room temperature for 1 h afforded a residue that was used in the next step without further purification. A HPLC trace is shown in section "**4. Spectra collection and HPLC-MS traces**".

(*S*)-3-(1'-Acetylindole-3'-yl)-2-(*tert*-butoxycarbonyl)aminopropanoic Acid (*S*)-70

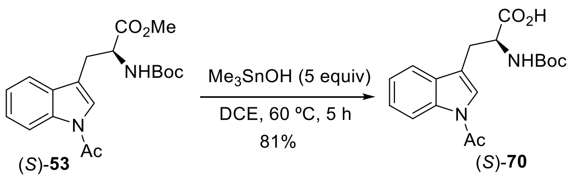

(*S*)-**53** (*S*)-**70**

Following the general procedure described above for the hydrolysis of methyl esters (Method B), the reaction of (*S*)-**53** (0.89 g, 2.46 mmol) with trimethyltin hydroxide (2.23 g, 12.32 mmol) in DCE (24.6 mL) at 60 ºC (metal heating block) for 5 h afforded, after purification by flash column chromatography (CombiFlash® Rf+ system, 40 g silica gel, gradient from 100:0 to 90:10 *v/v* CH_2Cl_2/MeOH, flow rate = 40 mL/min), 0.69 g (81% yield) of the titled compound as a white solid. **^{1}H NMR** (400 MHz, DMSO-*d*$_6$, 343 K) δ 8.32 – 8.27 (m, 1H), 7.65 – 7.58 (m, 2H), 7.32 (td, *J* = 7.9, 1.6 Hz, 1H), 7.28 (td, *J* = 7.4, 1.4 Hz, 1H), 7.02 – 6.86 (br, 1H, NH), 4.35-4.20 (br,

1H, H_2), 3.23 – 3.07 (m, 1H, H_{3A}), 3.01 (dd, *J* = 14.9, 9.3 Hz, 1H, H_{3B}), 2.60 (s, 3H, $COC\underline{H_3}$), 1.32 (s, 9H, $C(\underline{CH_3})_3$) ppm.

Allyl (2'*S*,2''*S*)-2-{2'-[3''-(1'''-Acetylindole-3'''-yl)-2''-(*tert*-butoxycarbonyl amino)propanamido]-3'-methylbutanamido}benzoate 71

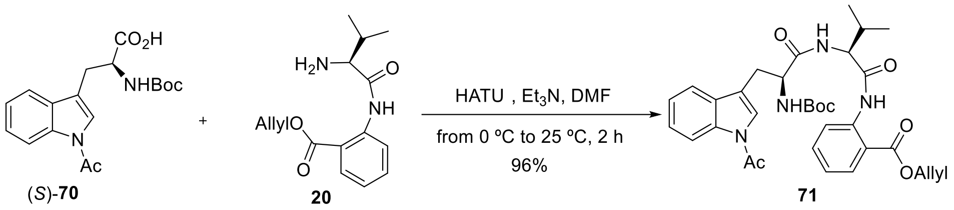

Following the general procedure described above for amide (peptide) bond formation (Method B), the reaction of tryptophan derivative (*S*)-**70** (0.65 g, 1.88 mmol), dipeptide **20** (0.52 g, 1.88 mmol), HATU (1.07 g, 2.82 mmol) and Et_3N (0.39 mL, 0.28 g, 2.82 mmol) in DMF (25.4 mL) at 25 ºC afforded, after 2 h reaction time and work-up, a residue that was purified by flash column chromatography (CombiFlash® Rf+ system, 80 g silica gel, gradient from 90:10 to 50:50 *v/v* hexane/EtOAc, flow rate = 60 mL/min) to afford 1.09 g (96% yield) of the titled compound as a white solid. **^{1}H NMR** (400 MHz, DMSO-d_6, 343 K) δ 8.38 (dd, *J* = 8.4, 0.9 Hz, 1H, ArH), 8.28 – 8.24 (m, 1H, ArH), 8.12 (d, *J* = 7.6 Hz, 1H, NH), 7.96 (dd, *J* = 8.0, 1.5 Hz, 1H, ArH), 7.71 (d, *J* = 7.4 Hz, 1H, ArH), 7.62 (ddd, *J* = 8.7, 7.4, 1.6 Hz, 1H, ArH), 7.58 (s, 1H, ArH), 7.29 (td, *J* = 7.6, 1.4 Hz, 1H, ArH), 7.25 (td, *J* = 7.5, 1.4 Hz, 1H, ArH), 7.21 (ddd, *J* = 8.4, 7.7, 1.2 Hz, 1H, ArH), 7.0-6.8 (br, 1H, NH), 5.97 (ddt, *J* = 17.2, 10.9, 5.6 Hz, 1H, $OCH_2C\underline{H}{=}CH_2$), 5.32 (app. dq, *J* = 17.3, 1.6 Hz, 1H, $OCH_2CH{=}C\underline{H}_{trans}H$), 5.22 (app. dq, *J* = 10.5, 1.3 Hz, 1H, $OCH_2CH{=}C\underline{H}_{cis}H$), 4.78 – 4.63 (m, 2H, $OC\underline{H_2}CH{=}CH_2$), 4.53 (app. td, *J* = 8.5, 4.5 Hz, 1H, $H_{2''}$), 4.28 (dd, *J* = 7.7, 6.2 Hz, 1H, $H_{2'}$), 3.23 (dd, *J* = 15.3, 4.9 Hz, 1H, $H_{3A''}$), 2.98 (dd, *J* = 14.6, 9.2 Hz, 1H, $H_{3B''}$), 2.52 (s, 3H, $COCH_3$), 2.31 – 2.15 (m, 1H, $C\underline{H}(CH_3)_2$), 1.32 (s, 9H, $C(CH_3)_3$), 0.97 (d, *J* = 6.8 Hz, 6H, $CH(C\underline{H_3})_2$) ppm. **^{13}C{^{1}H} NMR** (101 MHz, DMSO-d_6, 343 K) δ 171.9 (s), 169.8 (s), 168.4 (s), 166.4 (s), 155.0 (s), 139.4 (s), 134.9 (s), 133.9 (d), 131.9 (d), 130.4 (s), 130.3 (d), 124.4 (d), 124.3 (d), 123.1 (d), 122.7 (d), 120.6 (d), 118.9 (d), 118.0 (t), 117.4 (s), 116.8 (s), 115.5 (d), 78.1 (s), 65.2 (t), 59.6 (d), 53.8 (d), 29.8 (d), 27.8 (q), 26.7 (t), 23.3 (q), 18.9 (q), 17.7 (q) ppm. **HRMS** (ESI-TOF) *m/z*: $[M + H]^+$ calcd. for $C_{33}H_{41}N_4O_7$, 605.2970; found, 605.2976. **IR** (neat): ν 3400-3200 (br, N-H), 2970 (w, C-H), 2933 (w, C-H), 1691 (s, C=O), 1589 (w), 1516 (s), 1450 (m), 1388 (m), 1369 (m), 1255 (s), 1167 (m), 754 (m) cm^{-1}. $[\alpha]_D^{22}$ –17.5 (*c* 0.05, MeOH).

Bromine-containing precursor of synthetic novofumigatamide from L-Trp (*exo*-72 and *endo*-72)

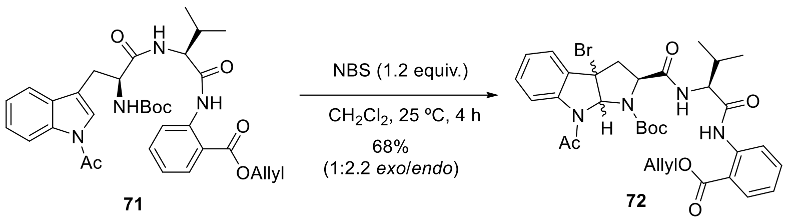

Following the general procedure described above for the bromocyclization with *N*-bromosuccinimide (Method A), the reaction of acyclic intermediate **71** (0.05 g, 83.0 μmol) and NBS (16.2 mg, 0.09 mmol) in CH_2Cl_2 (4 mL) at room temperature for 4 h afforded, after purification by flash column chromatography (CombiFlash® Rf+ system, 24 g silica gel, gradient from 90:10 to 80:20 *v/v* hexane/EtOAc, flow rate = 35 mL/min), 26.5 mg of *endo*-**72** and 12.0 mg of *exo*-**72** (68% overall yield), both as white solids.

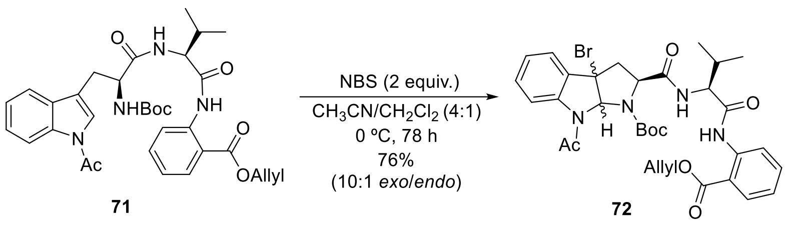

Following the general procedure described above for the bromocyclization with *N*-bromosuccinimide (Method A), the reaction of acyclic intermediate **71** (0.5 g, 0.84 mmol) and NBS (0.3 g, 1.67 mmol) in CH_2Cl_2/CH_3CN (99 mL, 4:1 *v/v*) at 0 ºC for 78 h afforded, after purification by flash column chromatography (CombiFlash® Rf+ system, 80 g silica gel, gradient from 90:10 to 70:30 hexane/EtOAc, flow rate = 60 mL/min), 0.40 g of *exo*-**72** and 40.6 mg of *endo*-**72** (76% overall yield), both as white solids.

REMARK: the time required to reach full conversion is strongly dependent on the reaction scale.

Endo-**72.** ^{1}**H NMR** (400 MHz, DMSO-*d*$_6$, 343 K) δ 10.73 (s, 1H, NH), 8.38 (d, *J* = 8.7 Hz, 1H, ArH), 8.04 (dd, *J* = 8.0, 1.5 Hz, 1H, ArH), 7.78 – 7.63 (m, 3H, ArH + NH), 7.29 – 7.21 (m, 1H, ArH), 7.16 – 7.08 (m, 1H, ArH), 7.07 (d, *J* = 7.5 Hz, 1H, ArH), 6.46 (t, *J* = 7.3 Hz, 1H, ArH), 6.28 (s, 1H, H_{8a}), 6.09 (ddt, *J* = 17.3, 10.7, 5.5 Hz, 1H, $OCH_2C\underline{H}=CH_2$), 5.44 (app. dq, *J* = 17.2, 1.6 Hz, 1H, $OCH_2CH=C\underline{H}_{trans}H$), 5.32 (app. dq, *J* = 10.5, 1.4 Hz, 1H, $OCH_2CH=C\underline{H}_{cis}H$), 4.93 – 4.79 (m, 2H, $OC\underline{H_2}CH=CH_2$), 4.61 – 4.48 (m, 1H, H_2), 3.80 (dd, *J* = 7.4, 6.1 Hz, 1H, $H_{2'}$), 3.36 – 3.28 (m, 1H, H_{3A}), 3.25 (dd, *J* = 13.2, 9.8 Hz, 1H, H_{3B}), 2.53 (s, 3H, $COC\underline{H_3}$), 1.94 – 1.84 (m, 1H, $C\underline{H}(CH_3)_2$), 1.32 (s, 9H, $C(CH_3)_3$), 0.75 (d, *J* = 6.7 Hz, 6H, $CH(C\underline{H_3})_2$) ppm. ^{13}**C{^{1}H} NMR** (101 MHz, DMSO-*d*$_6$, **343 K**) δ 170.1 (s), 169.9 (s), 169.6 (s), 167.0 (s), 153.2 (s), 141.3 (s), 139.7 (s), 134.5 (d), 133.3 (s), 132.2 (d), 130.7 (d), 130.0 (d), 124.1 (d), 123.5 (d), 123.4 (d), 120.8 (d),

118.6 (t), 118.5 (d), 116.8 (s), 85.5 (d), 80.7 (s), 65.7 (t), 63.0 (s), 60.7 (d), 59.9 (d), 41.8 (t), 30.0 (d), 27.8 (q), 23.6 (q), 19.1 (q), 18.0 (q) ppm. **HRMS** (ESI-TOF) *m/z*: $[M + H]^+$ calcd. for $C_{33}H_{40}{}^{79}BrN_4O_7$, 683.2074; found, 683.2074. **IR** (NaCl): ν 3400 (w, N-H), 3303 (w, N-H), 2965 (w, C-H), 2931 (w, C-H), 1682 (s, C=O), 1604 (m), 1590 (m), 1509 (m), 1476 (m), 1449 (m), 1395 (m), 1367 (m), 1339 (m), 1313 (m), 1256 (s), 1162 (m), 755 (m) cm^{-1}. $[\alpha]_D^{22}$ +41.5 (*c* 0.23, $CHCl_3$).

Exo-**72.** ^{1}**H NMR** (400 MHz, DMSO-d_6, 343 K) δ 8.47 (dd, *J* = 8.5, 0.8 Hz, 1H, ArH), 8.27 (d, *J* = 6.7 Hz, 1H, NH), 8.03 (dd, *J* = 8.0, 1.3 Hz, 1H, ArH), 7.83 (d, *J* = 8.0 Hz, 1H, ArH), 7.70 – 7.57 (m, 2H, ArH), 7.45 – 7.36 (m, 1H, ArH), 7.26 (td, *J* = 7.6, 1.0 Hz, 1H, ArH), 7.22 (ddd, *J* = 8.0, 7.3, 1.2 Hz, 1H, ArH), 6.20 (s, 1H, H_{8a}), 6.20 – 6.06 (m, 1H, $OCH_2C\underline{H}=CH_2$), 5.46 (app. dq, *J* = 17.3, 1.5 Hz, 1H, $OCH_2CH=C\underline{H}_{trans}H$), 5.34 (app. dq, *J* = 10.5, 1.3 Hz, 1H, $OCH_2CH=C\underline{H}_{cis}H$), 4.93 (app. t, *J* = 1.3 Hz, 1H, $OC\underline{H_2}CH=CH_2$), 4.92 (app. t, *J* = 1.4 Hz, 1H, $OC\underline{H_2}CH=CH_2$), 4.14 – 4.04 (m, 2H, H_2 + $H_{2'}$), 3.50 (dd, *J* = 12.9, 6.8 Hz, 1H, H_{3A}), 2.66 (dd, *J* = 12.8, 9.8 Hz, 1H, H_{3B}), 2.50 (s, 3H, $COC\underline{H_3}$), 2.23 – 2.10 (m, 1H, $C\underline{H}(CH_3)_2$), 1.29 (s, 9H, $C(CH_3)_3$), 0.98 (d, *J* = 6.8 Hz, 3H, $CH(C\underline{H_3})_2$), 0.97 (d, *J* = 6.8 Hz, 3H, $CH(C\underline{H_3})_2$) ppm. ^{13}**C{^{1}H} NMR** (101 MHz, DMSO-d_6, **343 K**) δ 170.7 (s), 169.6 (s), 169.4 (s), 166.5 (s), 151.8 (s), 140.5 (s), 139.6 (s), 133.9 (d), 133.0 (s), 132.0 (d), 130.3 (d), 130.2 (d), 124.7 (d), 123.4 (d), 122.8 (d), 120.3 (d), 118.3 (d), 118.0 (t), 116.3 (s), 84.3 (d), 80.5 (s), 65.3 (t), 61.0 (s), 60.6 (d, 2x), 40.8 (t), 29.5 (d), 27.5 (q), 23.2 (q), 18.7 (q), 18.0 (q) ppm. **HRMS** (ESI-TOF) *m/z*: $[M + H]^+$ calcd. for $C_{33}H_{40}{}^{79}BrN_4O_7$, 683.2075; found, 683.2075. **IR** (NaCl): ν 3400-3100 (br, N-H), 2964 (m, C-H), 2927 (m, C-H), 1687 (s, C=O), 1665 (s, C=O), 1604 (m), 1588 (m), 1523 (m), 1477 (m), 1465 (m), 1449 (m), 1410 (m), 1344 (m), 1320 (m), 1339 (m), 1313 (m), 1287 (m), 1257 (m), 1166 (m), 1143 (m), 755 (m) cm^{-1}. $[\alpha]_D^{21}$ –87.3 (*c* 0.22, $CHCl_3$).

***Exo* reverse-prenylated precursor of synthetic novofumigatamide from L-Trp (*exo*-73)**

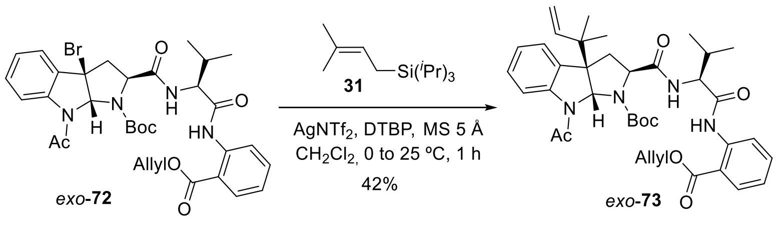

Following the general procedure described above for the reverse prenylation with triisopropyl(3-methyl-2-butenyl)silane (Method B), the reaction of *exo*-bromopyrroloindoline *exo*-**72** (0.2 g, 0.29 mmol), prenyl tri*iso*propyl silane (0.1 g, 0.44 mmol), freshly activated 5Å MS (1.64 g), DTBP (0.1 mL, 84.0 mg, 0.44 mmol) and $AgNTf_2$ (0.19 g, 0.44 mmol) in CH_2Cl_2 (6 mL) at room temperature for 1 h afforded, after purification by flash column chromatography (CombiFlash® Rf+ system, 24 g silica gel, gradient from 90:10 to 60:40 *v/v* hexane/EtOAc, flow rate = 35

mL/min), the desired compound *exo*-**73** (82.3 mg, 42% yield) as a white solid. **^{1}H NMR** (400 MHz, DMSO-d_6, 343 K) δ 10.84 (s, 1H, NH), 8.50 (d, *J* = 8.4 Hz, 1H, ArH), 8.29 – 8.23 (br, 1H, NH), 8.04 (dd, *J* = 8.0, 1.4 Hz, 1H, ArH), 7.78 (br, 1H, ArH), 7.68 – 7.60 (m, 1H, ArH), 7.36 (d, *J* = 7.5 Hz, 1H, ArH), 7.31 (t, *J* = 7.7 Hz, 1H, ArH), 7.25 – 7.14 (m, 2H, ArH), 6.12 (ddt, *J* = 17.5, 10.7, 5.4 Hz, 1H, $OCH_2C\underline{H}=CH_2$), 5.84 (br s, 1H, H_{8a}), 5.73 (dd, *J* = 17.1, 11.1 Hz, 1H, $C(CH_3)_2C\underline{H}=CH_2$), 5.46 (app. dq, *J* = 17.2, 1.7 Hz, 1H, $OCH_2CH=C\underline{H}_{trans}H$), 5.38 – 5.30 (m, 1H, $OCH_2CH=C\underline{H}_{cis}H$), 4.99 – 4.80 (m, 4H, $OC\underline{H_2}CH=CH_2 + C(CH_3)_2CH=C\underline{H_2}$), 4.08 – 4.01 (m, 1H, $H_{2'}$), 3.96 (dd, *J* = 9.1, 7.4 Hz, 1H, H_2), 2.58 (dd, *J* = 12.9, 7.2 Hz, 1H, H_{3A}), 2.43 (s, 3H, $COC\underline{H_3}$), 2.20 – 2.06 (m, 2H, H_{3B} + $C\underline{H}(CH_3)_2$), 1.28 (s, 9H, $C(CH_3)_3$), 1.04 – 0.83 (m, 9H, $C(C\underline{H_3})_2CH=CH_2 + CH(\underline{CH_3})_2)$), 0.81 (s, 3H, $C(C\underline{H_3})_2CH=CH_2$) ppm. **^{13}C{^{1}H} NMR** (101 MHz, DMSO-d_6, **343 K**) δ 172.3 (s), 169.9 (s), 168.9 (s), 166.6 (s), 152.4 (s), 143.2 (d), 142.0 (s), 139.8 (s), 134.1 (d), 133.9 (s), 132.0 (d), 130.4 (d), 128.0 (d), 124.6 (d), 123.5 (d), 122.8 (d), 120.1 (d), 118.0 (t), 118.0 (d), 115.9 (s), 113.2 (t), 79.7 (d), 78.9 (s), 65.2 (t), 60.9 (s), 60.6 (d), 59.3 (d), 39.6 (s), 34.2 (t), 29.4 (d), 27.6 (q), 23.4 (q), 22.6 (q), 21.7 (q), 18.8 (q), 18.3 (q) ppm. **HRMS** (ESI-TOF) *m/z*: $[M + H]^+$ calcd. for $C_{38}H_{49}N_4O_7$, 673.3595; found, 673.3596. **IR** (NaCl): ν 3400-3100 (br, N-H), 3009 (w, C-H), 2973 (m, C-H), 2933 (w, C-H), 2876 (w, C-H), 1693 (s, C=O), 1589 (m), 1524 (m), 1478 (m), 1449 (m), 1410 (m), 1366 (m), 1312 (m), 1256 (s), 1164 (m), 1152 (m), 1085 (m), 927 (m), 755 (s) cm^{-1}. $[\alpha]_D^{24}$ –47.1 (*c* 0.27, $CHCl_3$).

Exo **reverse-prenylated precursor of the synthetic novofumigatamide from L-Trp (*Exo*-74)**

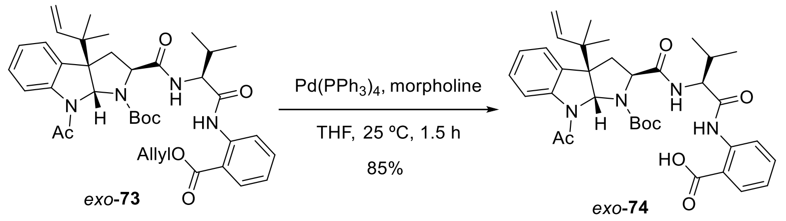

Following the general procedure described above for deprotection of allyl esters, the reaction of *exo*-**73** (0.06 g, 0.08 mmol), $Pd(PPh_3)_4$ (1.9 mg, 2.0 μmol) and morpholine (14.0 μL, 14.2 mg, 0.16 mmol) in THF (4.3 mL) at 25 ºC for 1.5 h afforded, after purification by flash column chromatography (silica gel, gradient from 95:5 to 90:10 *v/v* CH_2Cl_2/MeOH), 44.0 mg (85% yield) of the titled compound as a white solid. **^{1}H NMR** (400 MHz, DMSO-d_6, 343 K) δ 8.55 (dd, *J* = 8.3, 1.2 Hz, 1H, ArH), 8.13 (d, *J* = 6.9 Hz, 1H, NH), 8.07 (dd, *J* = 7.8, 1.8 Hz, 1H, ArH), 7.79 – 7.74 (m, 1H, ArH), 7.48 – 7.34 (m, 2H, ArH), 7.28 (td, *J* = 7.7, 1.3 Hz, 1H, ArH), 7.13 (td, *J* = 7.5, 1.2 Hz, 1H, ArH), 7.06 (td, *J* = 7.5, 1.3 Hz, 1H, ArH), 5.81 (br s, 1H, H_{8a}), 5.72 (dd, *J* = 17.6, 10.6 Hz, 1H, $C(CH_3)_2C\underline{H}=CH_2$), 4.95 – 4.92 (m, 1H, $C(CH_3)_2CH=C\underline{H_2}$), 4.91-4.88 (m, 1H, $C(CH_3)_2CH=C\underline{H_2}$), 4.06-3.96 (m, 1H, $H_{2'}$), 3.93 (dd, *J* = 9.2, 7.3 Hz, 1H, H_2), 2.65 (dd, *J* = 13.0, 7.3 Hz, 1H, H_{3A}), 2.43 (s, 3H, $COC\underline{H_3}$), 2.19 – 2.02 (m, 2H, H_{3B} + $C\underline{H}(CH_3)_2$), 1.26 (s, 9H, $C(CH_3)_3$), 1.00 – 0.91 (m, 9H, $C(C\underline{H_3})_2CH=CH_2 + CH(\underline{CH_3})_2)$), 0.80 (s, 3H, $C(C\underline{H_3})_2CH=CH_2$)

ppm. **^{13}C{^{1}H} NMR** (101 MHz, DMSO-*d*$_6$, **343 K**) δ 172.0 (s), 170.1 (s), 169.5 (s), 168.9 (s), 152.5 (s), 143.4 (d), 142.0 (s), 140.3 (s), 134.1 (s), 131.6 (d), 131.0 (d), 127.9 (d), 124.8 (d), 123.4 (d), 121.6 (d), 120.5 (s), 118.6 (d), 117.9 (d), 113.2 (t), 79.6 (d), 78.9 (s), 61.0 (d), 60.6 (s), 59.5 (d), 39.6 (s), 34.3 (t), 29.8 (d), 27.6 (q), 23.4 (q), 22.7 (q), 21.7 (q), 18.9 (q), 18.5 (q) ppm. **HRMS** (ESI-TOF) *m/z*: $[M + H]^+$ calcd. for $C_{35}H_{45}N_4O_7$, 633.3282; found, 633.3282. **IR** (NaCl): ν 3500-3100 (br, O-H, N-H), 3008 (m, C-H), 2971 (m, C-H), 2932 (m, C-H), 1679 (s, C=O), 1590 (m), 1512 (s), 1479 (m), 1451 (m), 1395 (s), 1368 (s), 1315 (m), 1287 (m), 1251 (m), 1215 (m), 1152 (m), 756 (s) cm^{-1}. **$[\alpha]_D^{24}$** –75.6 (*c* 0.21, $CHCl_3$).

Synthetic novofumigatamide from L-Trp (L-Trp-*exo*-11)

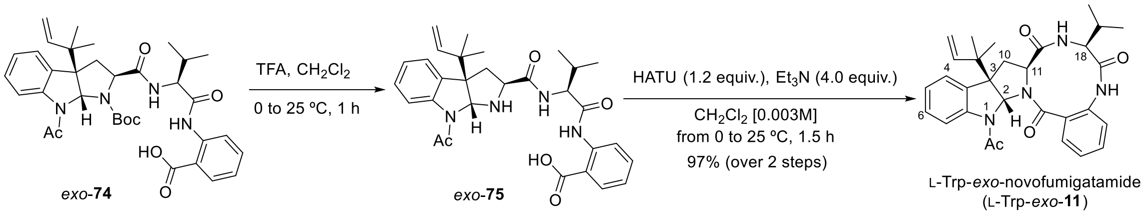

Following the general procedure described above for the deprotection of the *N*-Boc protected amines, the reaction of *exo*-**74** (0.02 g, 0.03 mmol), TFA (0.06 mL, 0.09 g, 0.78 mmol) in CH_2Cl_2 (0.3 mL) at room temperature for 1 h afforded a residue that was used in the next step without further purification.

Following the general procedure described above for the macrolactamization, the reaction of *exo*-**75** (17 mg, 32 μmol), Et_3N (5.0 μL, 4.0 mg, 38.0 μmol) and HATU (15 mg, 38.0 μmol) in CH_2Cl_2 (10.7 mL) at 0 ºC for 1.5 h afforded, after purification of the residue by flash column chromatography (silica gel, 95:5 *v/v* CH_2Cl_2/MeOH), 16 mg (97% yield, over two steps) of synthetic novofumigatamide from L-Trp (**L-Trp-*exo*-11**), as a white solid. **^{1}H NMR** (400 MHz, $CDCl_3$, 298 K) δ 8.24 – 8.05 (m, 2H, ArH), 7.86 – 7.75 (m, 1H, ArH), 7.67 (d, *J* = 9.6 Hz, 0.5H, NH from a rotamer), 7.65 – 7.47 (m, 2H, ArH), 7.35 (d, *J* = 7.5 Hz, 0.5H, ArH), 7.31 – 7.18 (m, 1H, ArH), 7.13 – 7.03 (m, 1.5H, ArH), 5.97 (dd, *J* = 16.8, 10.4 Hz, 0.37H, $C(CH_3)_2C\underline{H}=CH_2$, rotamer), 5.89 (dd, *J* = 17.3, 10.7 Hz, 0.63H, $C(CH_3)_2C\underline{H}=CH_2$, rotamer), 5.78 (s, 0.42H, H_2, rotamer), 5.41 (s, 0.53H, H_2, rotamer) 5.16 – 5.02 (m, 2H, $C(CH_3)_2CH=C\underline{H_2}$), 4.88 – 4.81 (m, 1H, H_{18}), 3.55 (td, *J* = 10.6, 5.6 Hz, 1H, H_{11}), 2.52 – 2.31 (m, 5H, $COC\underline{H_3}$ + H_{10A} + $C\underline{H}(CH_3)_2$, 2.29 – 2.18 (m, 1H, H_{10B}), 1.13 – 1.08 (m, 3H, $C(C\underline{H_3})_2CH=CH_2$), 1.05 – 1.00 (m, 3H, $CH(C\underline{H_3})_2$), 0.99 – 0.91 (m, 6H, $C(C\underline{H_3})_2CH=CH_2$ + $CH(C\underline{H_3})_2$) ppm. **^{13}C{^{1}H} NMR** (101 MHz, DMSO-*d*$_6$, **343 K**) δ 173.5 (s, rotamer: 172.9), 169.5 (s, rotamer: 168.6), 161.1 (s, rotamer: 160.9), 159.2 (s, rotamer: 159.1), 145.8 (s, rotamer: 145.7), 143.8 (d), 143.1 (s, rotamer: 141.9), 136.9 (d, rotamer: 136.7), 135.7 (s, rotamer: 133.2), 129.0, 128.9, 128.8, 128.7, 128.6, 128.6 (d, 3 x C, 2 rotamers each carbon), 127.3 (d, rotamer: 127.0), 126.8 (d, rotamer: 125.2), 124.1 (d, rotamer: 123.8), 117.2 (d), 117.0 (s), 114.5 (t, rotamer: 114.4), 113.7 (d), 81.8 (d, rotamer: 80.9), 63.6 (s, rotamer: 60.7), 61.1

(d, rotamer: 60.3), 56.4 (d, rotamer: 55.8), 41.3 (t, rotamer: 40.3), 41.2 (s, rotamer: 41.1), 31.9 (d, rotamer: 31.7), 25.0 (q, rotamer: 24.2), 23.8 (q, rotamer: 23.5), 22.7 (q, rotamer: 22.4), 19.7 (q, rotamer: 19.6), 17.8 (q, rotamer: 17.5) ppm. **HRMS** (ESI-TOF) *m/z*: $[M + H]^+$ calcd. for $C_{30}H_{35}N_4O_4$, 515.2653; found, 515.2655. **IR** (NaCl): ν 3400-3100 (br, N-H), 2965 (m, C-H), 2928 (m, C-H), 1762 (s, C=O), 1664 (s, C=O), 1645 (s, C=O), 1514 (m), 1479 (s), 1465 (m), 1399 (s), 1368 (s), 1315 (m), 1284 (m), 1258 (m), 1135 (m), 1007 (m), 756 (s) cm^{-1}. $[\alpha]_D^{22}$ –62.9 (*c* 0.14, $CHCl_3$).

REMARK: difficult to distinguish rotamers in the aromatic signals.

***Endo* reverse-prenylated precursor of synthetic novofumigatamide from L-Trp (*endo*-73)**

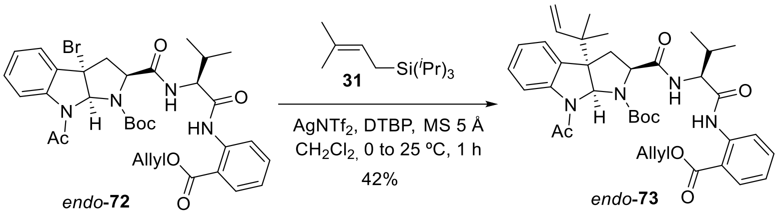

Following the general procedure described above for the reverse prenylation with triisopropyl(3-methyl-2-butenyl)silane, the reaction of *endo*-bromopyrroloindoline *endo*-**72** (0.13 g, 0.19 mmol), prenyl triisopropyl silane **31** (64.0 mg, 0.28 mmol), freshly activated 5Å MS (1.07 g), DTBP (63.0 μL, 54.0 mg, 0.28 mmol) and $AgNTf_2$ (0.12 g, 0.28 mmol) in CH_2Cl_2 (3.7 mL) at room temperature for 1 h afforded, after purification by flash column chromatography (CombiFlash® Rf+ system, 24 g silica gel, gradient from 90:10 to 70:30 *v/v* hexane/EtOAc, flow rate = 35 mL/min), the desired compound *endo*-**73** (53.2 mg, 42% yield) as a white solid. **^{1}H NMR** (400 MHz, DMSO-d_6, 343 K) δ 10.65 (s, 1H, NH), 8.36 (dd, *J* = 8.4, 1.2 Hz, 1H, ArH), 8.04 (dd, *J* = 8.0, 1.7 Hz, 1H, ArH), 7.74 – 7.61 (m, 2H, ArH), 7.48 (d, *J* = 7.5 Hz, 1H, NH), 7.26 (ddd, *J* = 7.9, 7.3, 1.2 Hz, 1H, ArH), 7.02 – 6.94 (m, 1H, ArH), 6.87 (d, *J* = 7.5 Hz, 1H, ArH), 6.33 (t, *J* = 7.5 Hz, 1H, ArH), 6.08 (ddt, *J* = 17.3, 10.7, 5.4 Hz, 1H, $OCH_2C\underline{H}=CH_2$), 5.89 – 5.79 (m, 2H, H_{8a} + $C(CH_3)_2C\underline{H}=CH_2$), 5.43 (app. dq, *J* = 17.3, 1.6 Hz, 1H, $OCH_2CH=C\underline{H}_{trans}H$), 5.31 (app. dq, *J* = 10.5, 1.4 Hz, 1H, $OCH_2CH=C\underline{H}_{cis}H$), 5.09 – 5.02 (m, 2H, $C(CH_3)_2CH=C\underline{H_2}$), 4.85 (app. dt, *J* = 5.3, 1.6 Hz, 2H, $OC\underline{H_2}CH=CH_2$), 4.49 (d, *J* = 9.8 Hz, 1H, H_2), 3.75 – 3.67 (m, 1H, $H_{2'}$), 2.72 – 2.62 (m, 1H, H_{3A}), 1.90 – 1.77 (m, 4H, H_{3B} + $COC\underline{H_3}$), 1.31 (s, 9H, $C(CH_3)_3$), 0.99 (s, 3H, $C(C\underline{H_3})_2CH=CH_2$), 0.79 (s, 3H, $C(C\underline{H_3})_2CH=CH_2$), 0.74 (d, *J* = 6.8 Hz, 6H, $CH(\underline{CH_3})_2$) ppm. **^{13}C{^{1}H} NMR** (101 MHz, DMSO-d_6, **343 K**) δ 170.3 (s), 170.0 (s), 169.6 (s), 166.9 (s), 153.6 (s), 143.8 (d), 142.9 (s), 139.7 (s), 134.4 (d), 133.3 (s), 132.3 (d), 130.7 (d), 127.8 (d), 124.6 (d), 123.4 (d), 122.7 (d), 120.9 (d), 118.3 (t), 118.2 (d), 116.9 (s), 114.0 (t), 80.1 (d), 80.1 (s), 65.6 (t), 61.2 (s), 60.6 (d), 59.8 (d), 34.5 (t), 30.2 (d), 27.8 (q), 23.8 (q), 22.7 (q), 22.0 (q), 19.1 (q), 18.2 (q) ppm. **HRMS** (ESI-TOF) *m/z*: $[M + H]^+$ calcd. for $C_{38}H_{49}N_4O_7$, 673.3596; found, 673.3599. **IR** (NaCl): ν 3404 (w, N-H), 3304 (w, N-H), 2968 (w, C-H), 2933 (w, C-H), 1706 (s, C=O), 1676

(s, C=O), 1590 (m), 1506 (m), 1449 (m), 1395 (m), 1255 (s), 1157 (m), 755 (m) cm^{-1}. $[\boldsymbol{\alpha}]_D^{24}$ +6.8 (*c* 0.2, $CHCl_3$).

Endo reverse-prenylated precursor of synthetic novofumigatamide from L-Trp (_endo_-74)

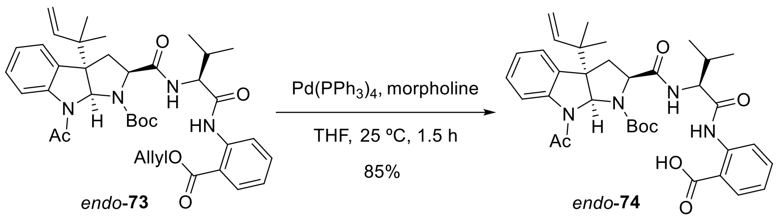

Following the general procedure described above for deprotection of allyl esters, the reaction of *endo*-**73** (0.05 g, 73.0 μmol), $Pd(PPh_3)_4$ (1.7 mg, 1.5 μmol) and morpholine (13.0 μL, 12.7 mg, 0.15 mmol) in THF (3.8 mL) at 25 ºC for 2 h afforded, after purification by flash column chromatography (silica gel, 95:5 *v/v* CH_2Cl_2/MeOH), 44.8 mg (92% yield) of the titled compound as a white solid. *Endo*-**74. ^{1}H NMR** (400 MHz, DMSO-d_6, 343 K) δ 12.15 (br s, 1H, OH), 8.47 (d, *J* = 8.4 Hz, 1H, ArH), 8.04 (d, *J* = 7.8 Hz, 1H, ArH), 7.70 (d, *J* = 8.3 Hz, 1H, ArH), 7.51 – 7.43 (m, 1H, ArH), 7.16 – 6.94 (m, 4H, 3ArH + NH), 6.60 (t, *J* = 7.5 Hz, 1H, ArH), 5.92 (s, 1H, H_{8a}), 5.84 (dd, *J* = 17.0, 11.2 Hz, 1H, $C(CH_3)_2C\underline{H}=CH_2$), 5.09 – 5.00 (m, 2H, $C(CH_3)_2CH=C\underline{H_2}$), 4.57 – 4.45 (m, 1H, H_2), 3.71 (dd, *J* = 7.7, 6.2 Hz, 1H, $H_{2'}$), 2.68 – 2.57 (m, 2H, $2H_3$), 2.49 (s, 3H, $COC\underline{H_3}$), 1.79 – 1.67 (m, 1H, $C\underline{H}(CH_3)_2$), 1.36 (s, 9H, $C(CH_3)_3$), 1.02 (s, 3H, $C(C\underline{H_3})_2CH=CH_2$), 0.83 (s, 3H, $C(C\underline{H_3})_2CH=CH_2$), 0.72 (d, *J* = 6.7 Hz, 3H, $CH(C\underline{H_3})_2$), 0.70 (d, *J* = 6.7 Hz, 3H, $CH(C\underline{H_3})_2$) ppm. **^{13}C{^{1}H} NMR** (101 MHz, DMSO-d_6, **343 K**) δ 169.7 (s), 169.1 (s), 169.0 (s), 153.6 (s), 143.6 (d), 142.5 (s), 140.0 (s), 133.0 (s), 132.2 (d), 130.9 (d), 127.6 (d), 124.8 (d), 122.8 (d), 122.1 (d), 119.4 (s), 119.1 (d), 117.7 (d), 113.5 (t), 80.3 (s), 80.1 (d), 78.9 (s), 61.2 (s), 60.9 (d), 59.5 (d), 34.0 (t), 30.3 (d), 27.5 (q), 23.4 (q), 22.5 (q), 21.8 (q), 18.6 (q), 18.0 (q) ppm. **HRMS** (ESI-TOF) *m/z*: $[M + H]^+$ calcd. for $C_{35}H_{45}N_4O_7$, 633.3283; found, 633.3287. **IR** (NaCl): ν 3500-3100 (br, O-H, N-H), 3008 (m, C-H), 2970 (m, C-H), 2932 (m, C-H), 1710 (s, C=O), 1680 (s, C=O), 1590 (m), 1506 (s), 1479 (m), 1449 (m), 1395 (s), 1368 (m), 1339 (m), 1288 (m), 1252 (m), 1230 (m), 1156 (m), 756 (s) cm^{-1}. $[\boldsymbol{\alpha}]_D^{24}$ –23.4 (*c* 0.22, $CHCl_3$).

REMARK: the ^{13}C{^{1}H} NMR signal of the CO_2H carbonyl is missing.

Synthetic novofumigatamide from L-Trp (L-Trp-_endo_-11)

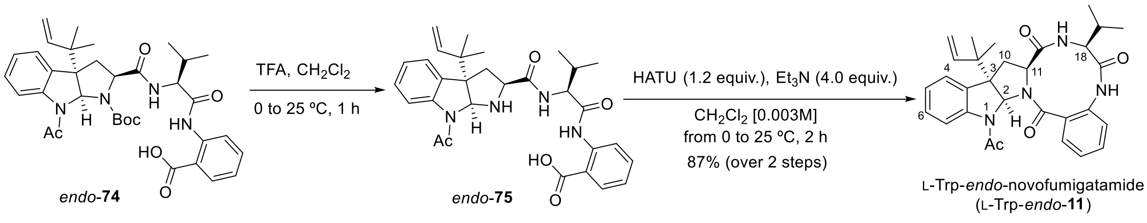

Following the general procedure described above for the deprotection of the *N*-Boc-protected amines, the reaction of *endo*-**74** (0.02 g, 31.6 μmol) with TFA (0.06 mL, 0.09 g, 0.78 mmol) in

CH_2Cl_2 (0.3 mL) at room temperature for 1 h afforded a residue that was used in the next step without further purification.

Following the general procedure described above for the macrolactamization, the reaction of *endo*-**75** (17 mg, 32 μmol), Et_3N (5.0 μL, 4.0 mg, 38.0 μmol) and HATU (15 mg, 38.0 μmol) in CH_2Cl_2 (10.7 mL) at 0 ºC for 2 h afforded, after purification of the residue by flash column chromatography (silica gel, 97.5:2.5 *v/v* CH_2Cl_2/MeOH), 14.3 mg (87% yield, over two steps) of synthetic novofumigatamide from L-Trp **(**L-Trp-*endo*-**11)**, as a white solid. **^{1}H NMR** (400 MHz, $CDCl_3$, 298 K) δ 8.18 – 8.05 (m, 2H, ArH), 7.82 – 7.73 (m, 1H, ArH), 7.71 – 7.63 (m, 1.5H, 0.5ArH + 1NH), 7.58 – 7.42 (m, 3H, 1.5ArH + 1.5NH), 7.32 – 7.17 (m, 1.5H, ArH + 0.5 NH), 7.11 – 7.00 (m, 1.5H, ArH), 5.97 – 5.77 (m, 1.3H, $C(CH_3)_2C\underline{H}=CH_2$ + $0.3H_2$), 5.45 (s, 0.7H, $0.7H_2$), 5.21 – 4.94 (m, 2H, $C(CH_3)_2CH=C\underline{H_2}$), 4.38 (dd, *J* = 8.7, 5.6 Hz, 0.3H, $0.3H_{18}$), 4.21 (dd, *J* = 8.0, 5.3 Hz, 0.7H, $0.7H_{18}$), 3.97 – 3.85 (m, 1H, H_{11}), 2.78 – 2.61 (m, 2H, $2H_{10}$), 2.46 (s, 1H, $COC\underline{H_3}$), 2.42 (s, 2H, $COC\underline{H_3}$, rotamer), 1.98 – 1.65 (m, 1H, $C\underline{H}(CH_3)_2$), 1.10 (s, 2H, $C(C\underline{H_3})_2CH=CH_2$), 1.04 (s, 1H, $C(C\underline{H_3})_2CH=CH_2$, rotamer), 0.89 (s, 1H, $C(C\underline{H_3})_2CH=CH_2$), 0.85 (s, 2H, $C(C\underline{H_3})_2CH=CH_2$, rotamer), 0.67 – 0.52 (m, 6H, $CH(C\underline{H_3})_2$) ppm. **^{13}C{^{1}H} NMR** (101 MHz, $CDCl_3$) δ 174.2 (s, rotamer: 173.2), 169.0 (s, rotamer: 168.6), 161.1 (s, rotamer: 160.9), 159.2 (s), 145.8 (s), 143.8 (d, rotamer: 143.5), 142.3 (s), 140.7 (s, rotamer), 136.8 (s, rotamer), 136.7 (d), 133.2 (s), 132.3 (d, rotamers: 132.2, 132.1), 128.9 (d), 128.7 (d), 128.6 (d, 2x, rotamers), 127.2 (d, rotamers: 127.1, 127.0), 125.6 (d, rotamer), 124.1 (d), 123.7 (d, rotamer), 117.0 (d), 116.9 (s), 114.7 (t, rotamer: 114.4), 83.5 (d, rotamer: 81.7), 62.9 (s, rotamer: 60.0), 61.8 (d, rotamer: 60.6), 57.0 (d, rotamer: 56.4), 41.6 (s, rotamer: 41.5), 37.9 (t, rotamer: 37.7), 31.7 (d, rotamer: 31.5), 24.8 (q, rotamer: 24.5), 23.0 (q, rotamer: 22.9), 22.7 (q, rotamer: 22.4), 19.2 (q, rotamer: 18.9), 18.0 (q, rotamer: 17.7) ppm. **HRMS** (ESI-TOF) *m/z*: $[M + H]^+$ calcd. for $C_{30}H_{35}N_4O_4$, 515.2653; found, 515.2650. **IR** (NaCl): ν 3400-3100 (br, N-H), 2965 (m, C-H), 2931 (w, C-H), 1762 (s, C=O), 1664 (s, C=O), 1643 (s, C=O), 1608 (m, C=O), 1508 (m), 1478 (s), 1398 (m), 1311 (w), 1285 (w), 1135 (m), 1004 (w), 756 (m) cm^{-1}. $[\alpha]_D^{24}$ –30.4 (*c* 0.14, $CHCl_3$). REMARK: difficult to distinguish rotamers in the aromatic signals.

3. Comparative tables of the spectroscopic data of natural and synthetic products

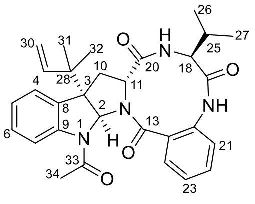

Proposed structure for (-)-novofumigatamide (**11**)

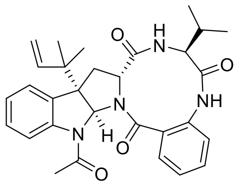

D-Trp-*exo*-**11**

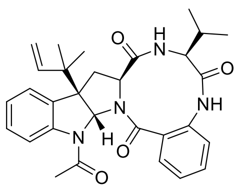

L-Trp-*exo*-**11**

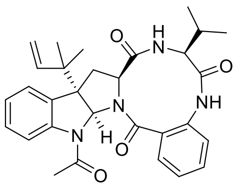

L-Trp-*endo*-**11**

The atom numbering from the original publication is used.[5]

Table 1. ^{1}H and $^{13}C\{^{1}H\}$ NMR data for the most representative signals of (-)-novofumigatamide (**11**) in $CDCl_3$ at 298K

	**Natural source**		**Synthetic compounds**			
	**(-)-novofumigatamide (11)**		**D-Trp-*exo*-11**		**L-Trp-*exo*-11**	
**Atom no.**	δ_H (mult., *J*/Hz)	δ_C	δ_H (mult., *J*/Hz)	δ_C	δ_H (mult., *J*/Hz)	δ_C
2	6.04 (s)	79.2 CH	5.78 (s, 0.5H)<br>5.42 (s, 0.5H)	82.0 CH	5.78 (s, 0.42H)<br>5.41 (s, 0.53H)	81.8 CH
10	2.99 (dd, 12.4, 5.5, H_{10A})<br>2.62 (dd, 12.4, 10.7, H_{10B})	37.5 CH_2	2.52 – 2.21 (m)	41.0 CH_2	2.52 – 2.31 (m, H_{10A})<br>2.29 – 2.18 (m, H_{10B})	41.3 CH_2
11	4.43 (dd, 10.7, 5.5)	59.0 CH	3.68 – 3.50 (m)	61.2 CH	3.55 (td, 10.6, 5.6)	61.1 CH
18	5.22 (d, 7.5)	62.3 CH	4.81 – 4.76 (m)	56.2 CH	4.88 – 4.81 (m)	56.4 CH
25	1.98 (m)	31.9 CH	2.52 – 2.21 (m)	31.8 CH	2.52 – 2.31 (m)	31.9 CH
29	5.84 (dd, 17.4, 10.8)	143.1 CH	6.06 – 5.85 (m)	143.9 CH	5.97 (dd, 16.8, 10.4, 0.37H)<br>5.89 (dd, 17.3, 10.7, 0.63H)	143.8 CH
30	5.13 (br s, 17.4)	114.6 CH_2	5.09 (d, 10.8)<br>5.04 (dd, 17.4, 1.2)	114.3 CH_2	5.16 – 5.02 (m)	114.5 CH_2

Table 2. ^{1}H and $^{13}C\{^{1}H\}$ NMR data for the most representative signals of (-)-novofumigatamide (**11**) in $CDCl_3$ at 298K

	**Natural source**		**Synthetic compounds**	
	**(-)-novofumigatamide (11)**		**L-Trp-*endo*-11**	
**Atom no.**	δ_H (mult., *J*/Hz)	δ_C	δ_H (mult., *J*/Hz)	δ_C
2	6.04 (s)	79.2 CH	5.97 – 5.77 (m, 0.3H)<br>5.45 (s, 0.7H)	83.5 CH
10	2.99 (dd, 12.4, 5.5, H_{10A})<br>2.62 (dd, 12.4, 10.7, H_{10B})	37.5 CH_2	2.78 – 2.61 (m)	37.9 CH_2
11	4.43 (dd, 10.7, 5.5)	59.0 CH	3.97 – 3.85 (m)	61.8 CH
18	5.22 (d, 7.5)	62.3 CH	4.38 (dd, 8.7, 5.6, 0.3H_{18})<br>4.21 (dd, 8.0, 5.3,0.7H_{18})	57.0 CH
25	1.98 (m)	31.9 CH	1.98 – 1.65 (m)	31.7 CH
29	5.84 (dd, 17.4, 10.8)	143.1 CH	5.97 – 5.77 (m)	143.8 CH
30	5.13 (br s, 17.4)	114.6 CH_2	5.21 – 4.94 (m)	114.7 CH_2

4. Spectra collection and HPLC-MS traces

^{1}H NMR (400 MHz, $CDCl_3$)

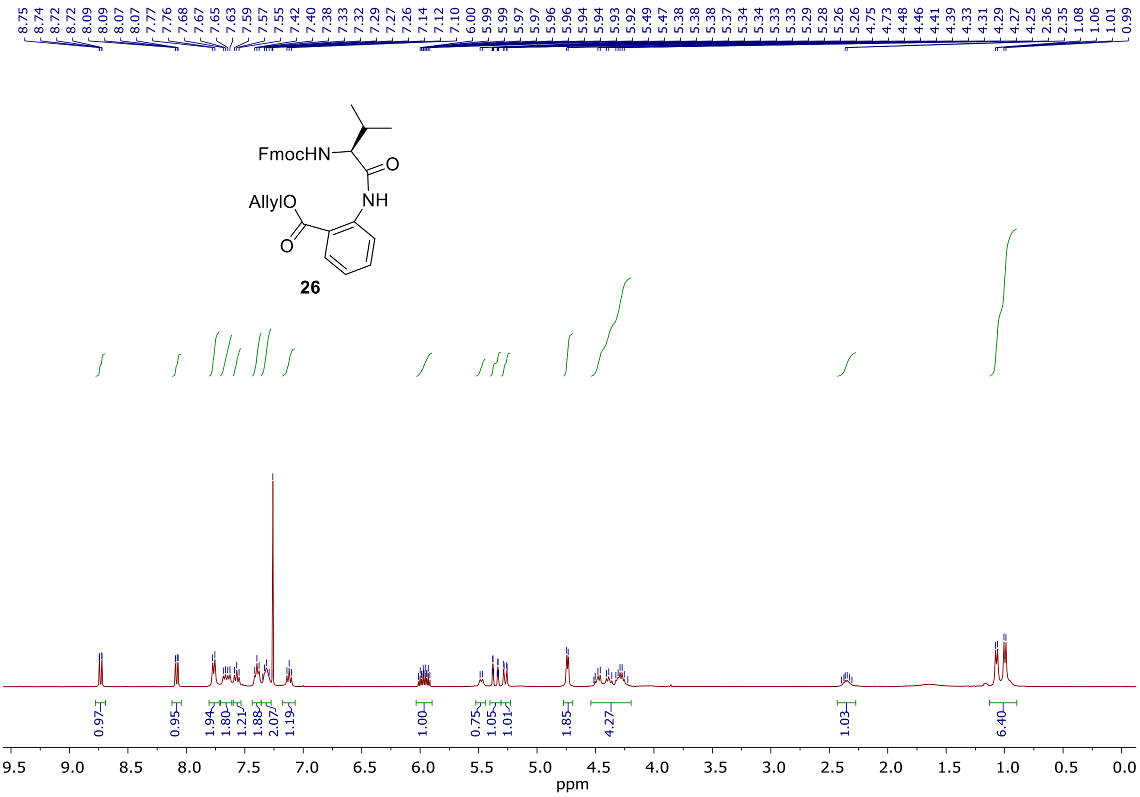

^{13}C{^{1}H} NMR (101 MHz, $CDCl_3$)

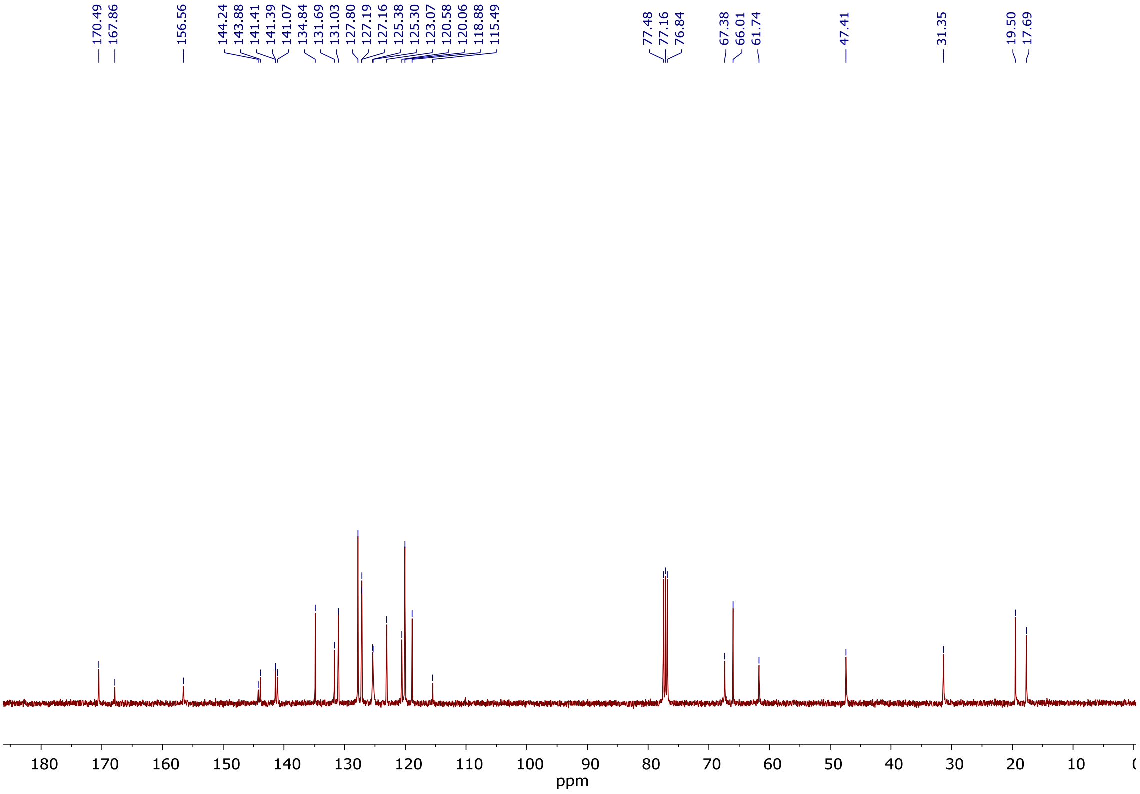

^{1}H NMR (400 MHz, $CDCl_3$)

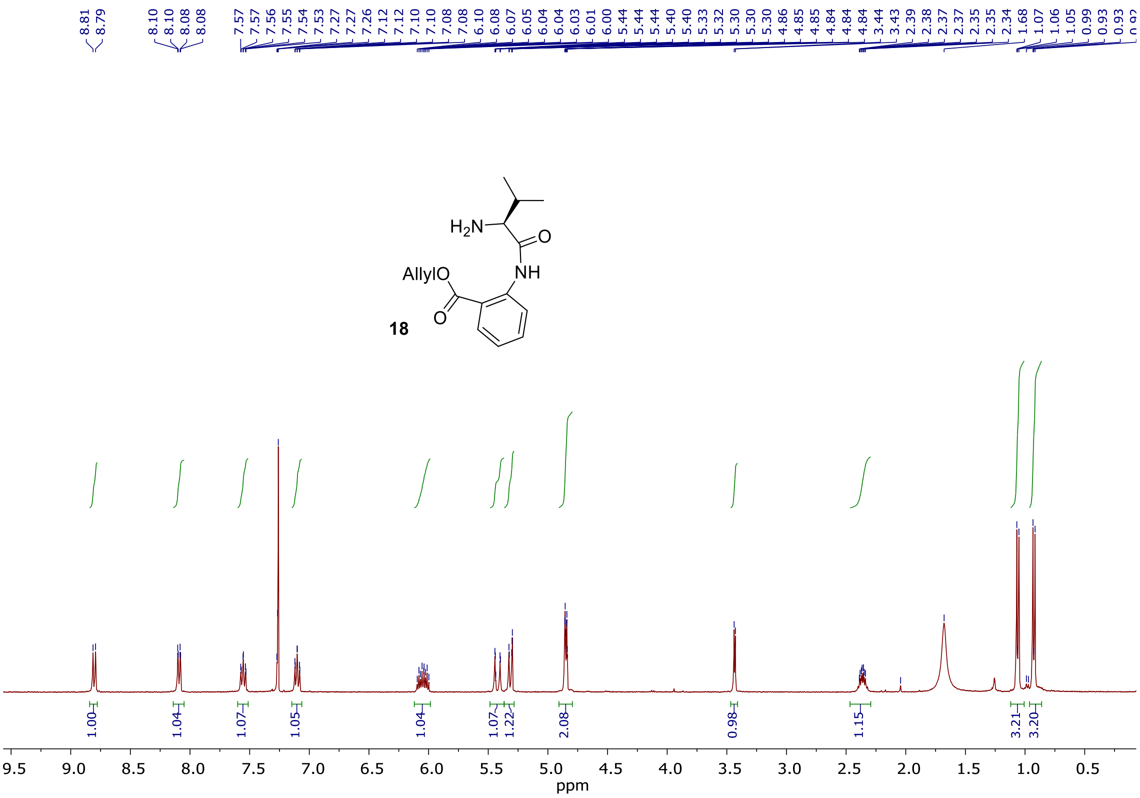

$^{13}C\{^{1}H\}$ NMR (101 MHz, $CDCl_3$)

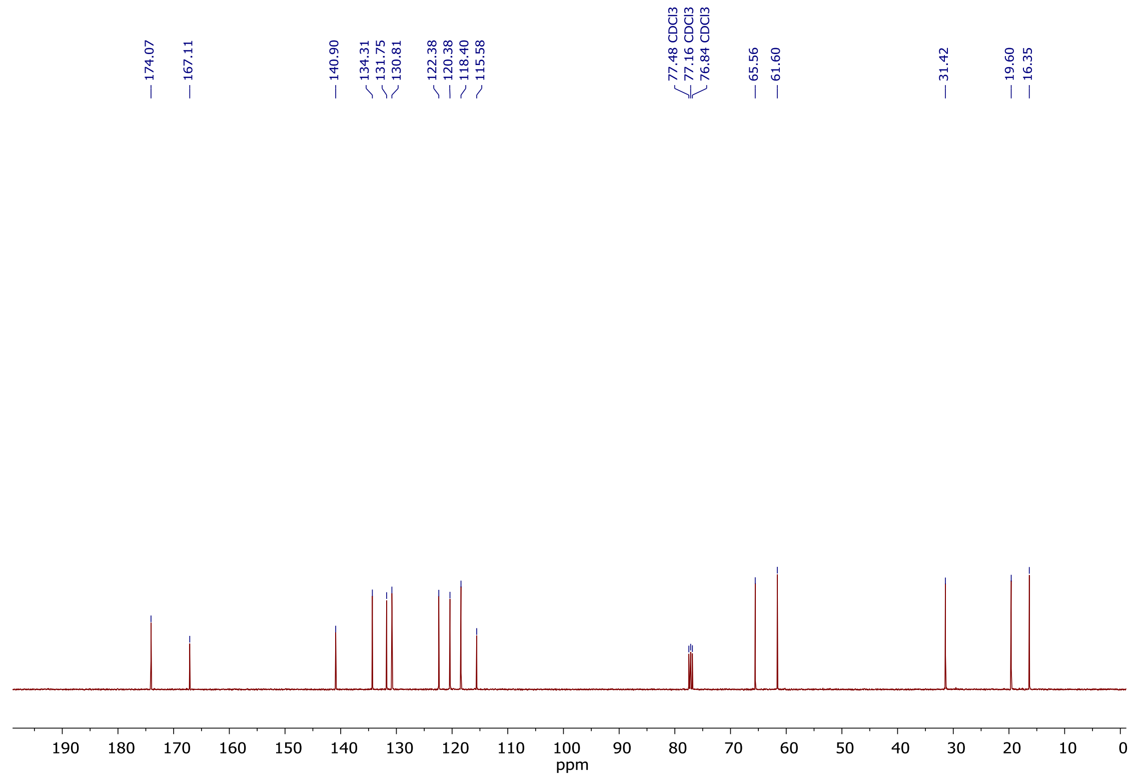

^{1}H NMR (400 MHz, CD_3OD)

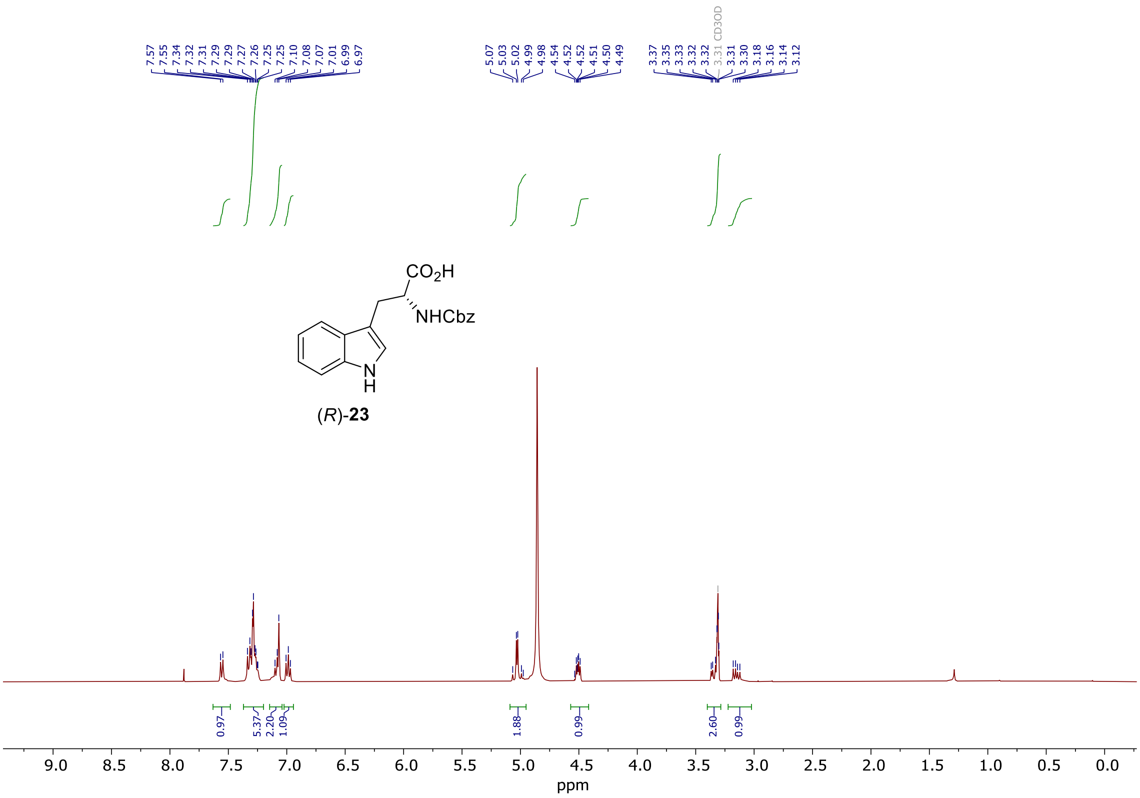

^{1}H NMR (400 MHz, $CDCl_3$)

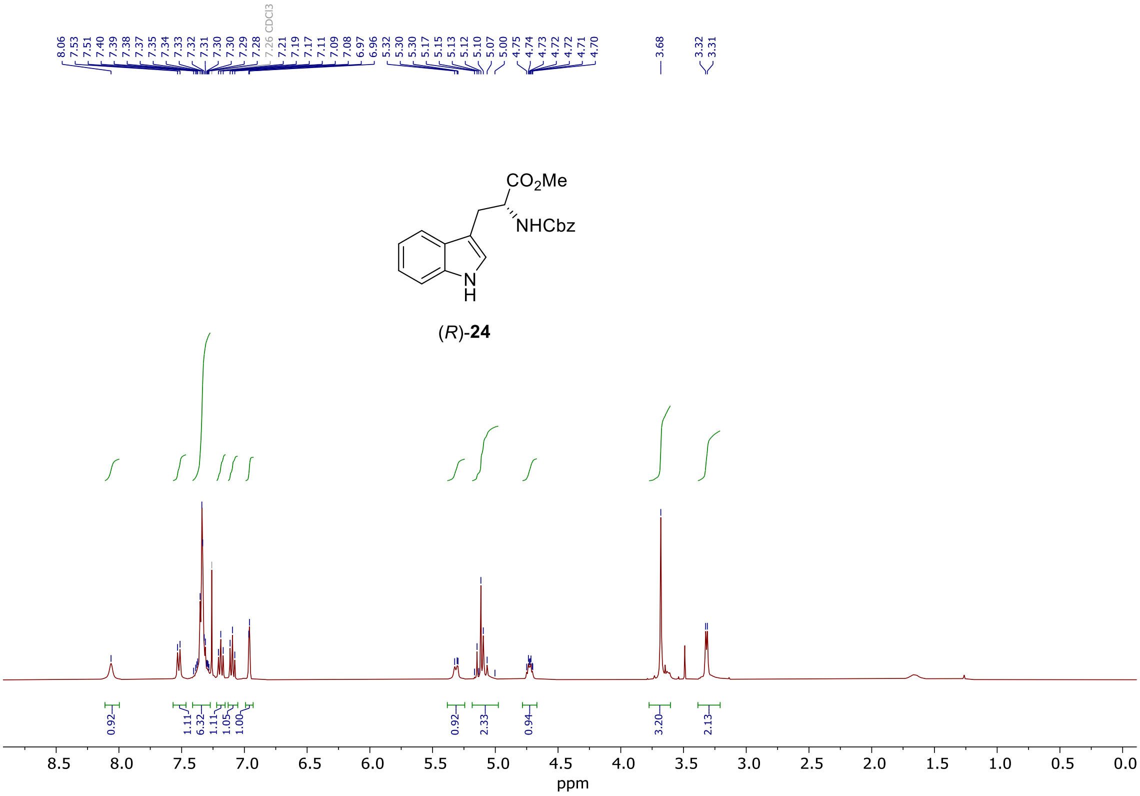

^{1}H NMR (400 MHz, $CDCl_3$)

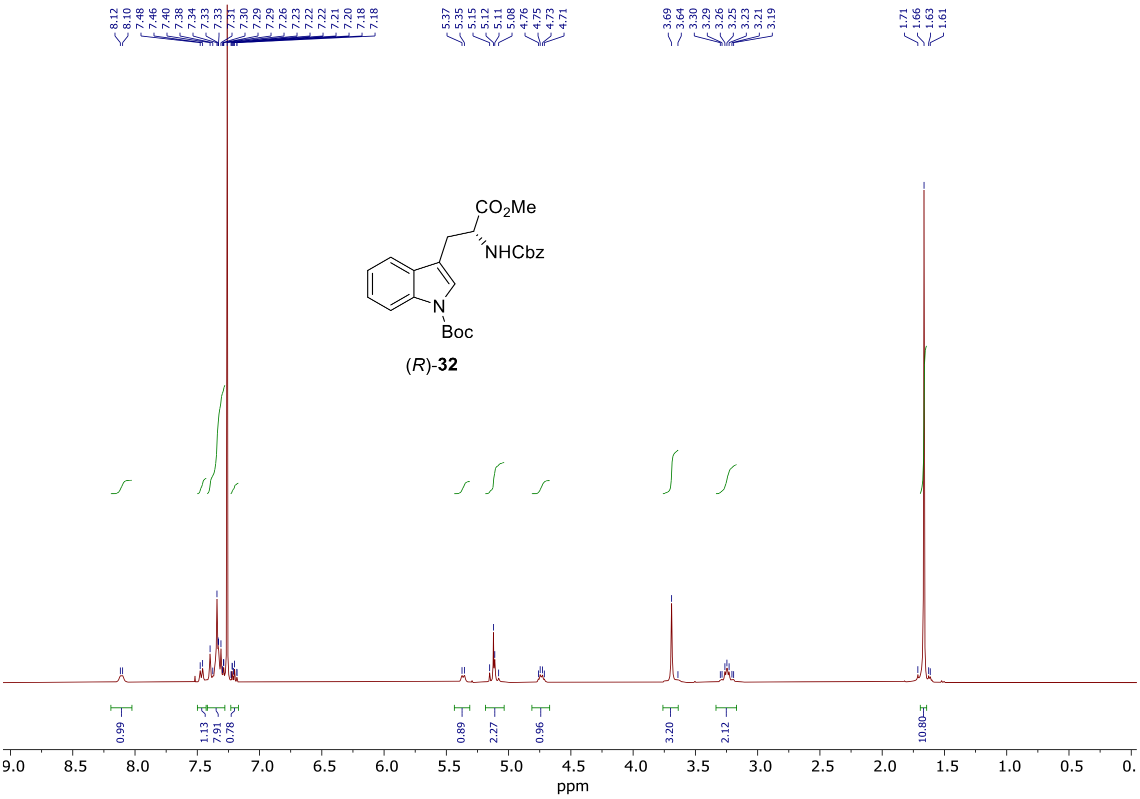

^{1}H NMR (400 MHz, DMSO-d_6, 343 K)

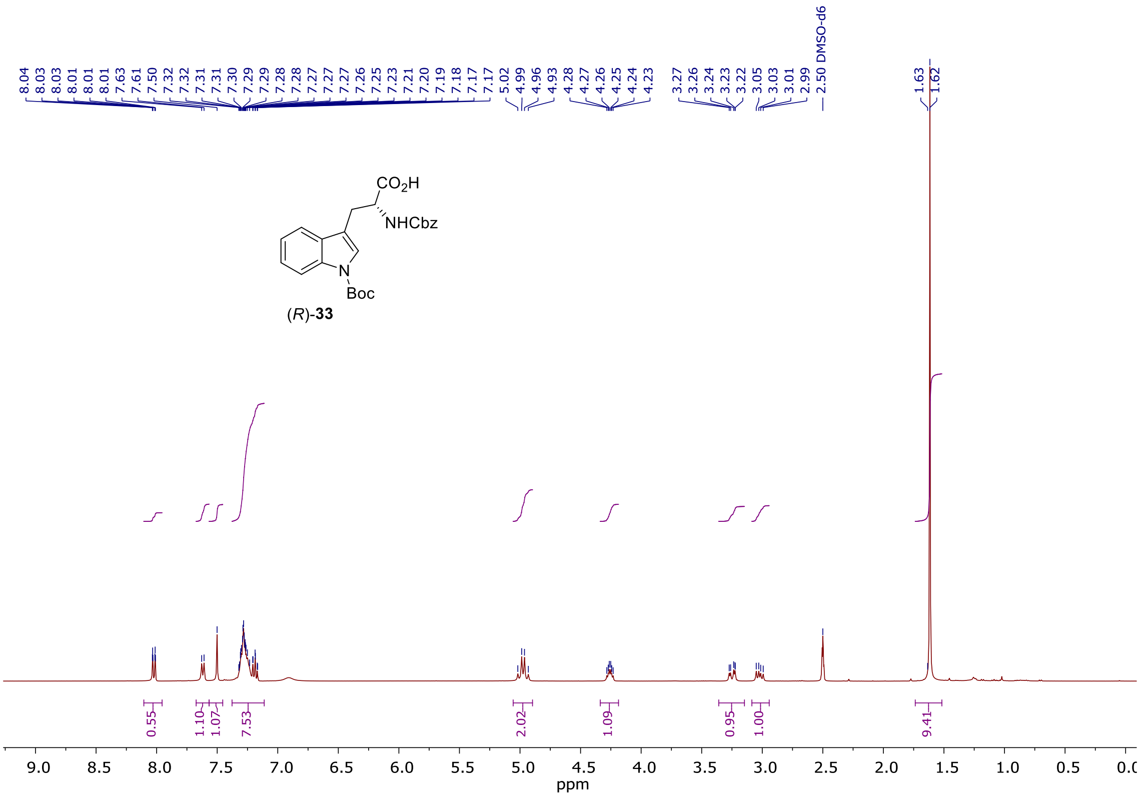

^{13}C{^{1}H} NMR (101 MHz, DMSO-d_6, 343 K)

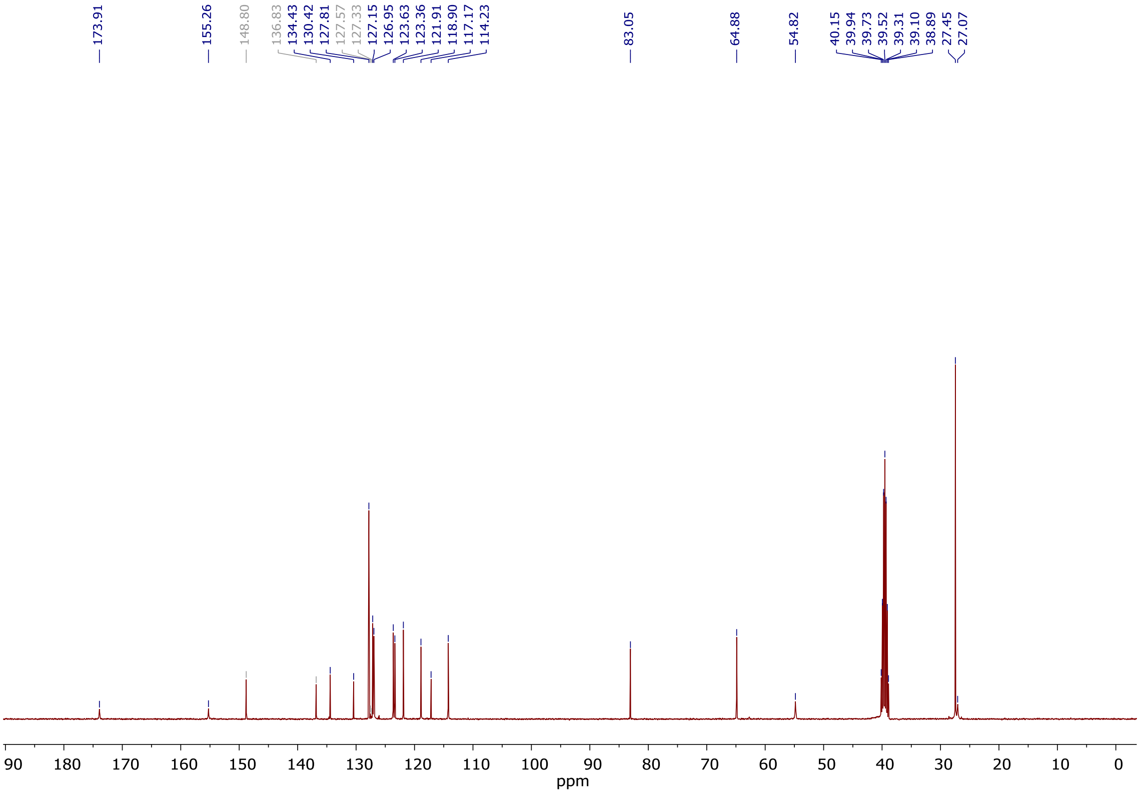

^{1}H NMR (400 MHz, DMSO-d_6, 343 K)

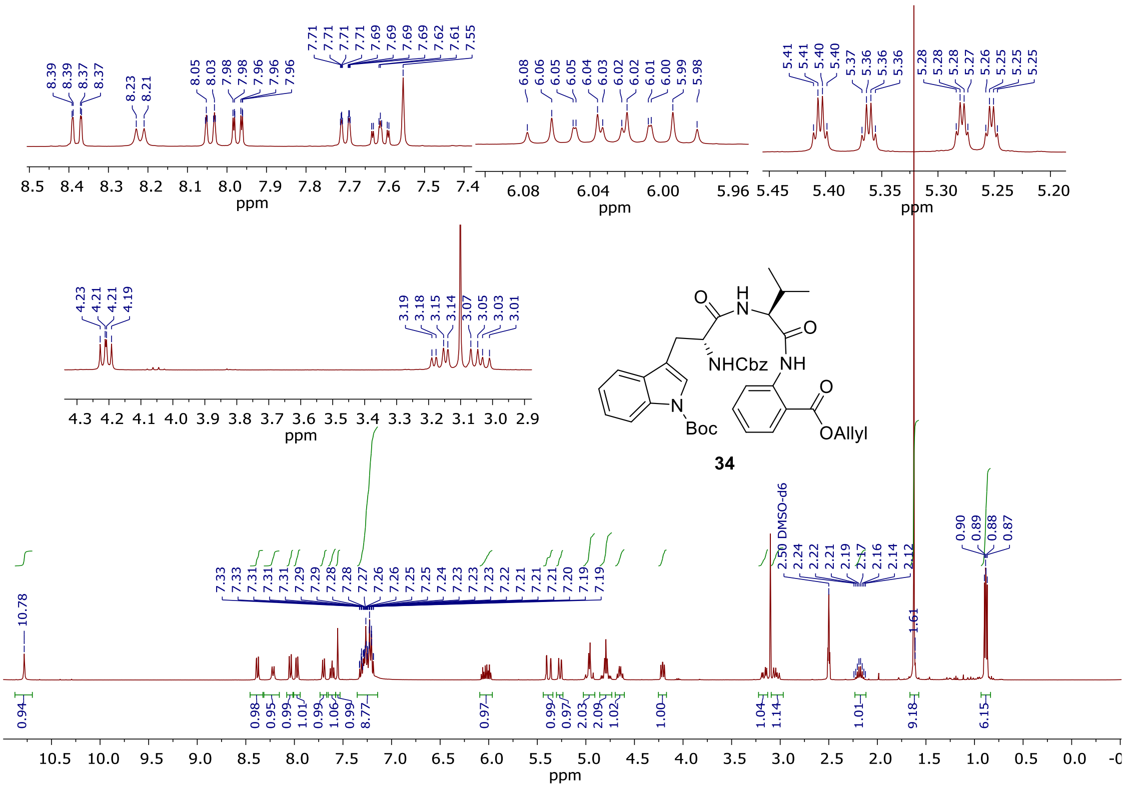

^{13}C{^{1}H} NMR (101 MHz, DMSO-d_6, 343 K)

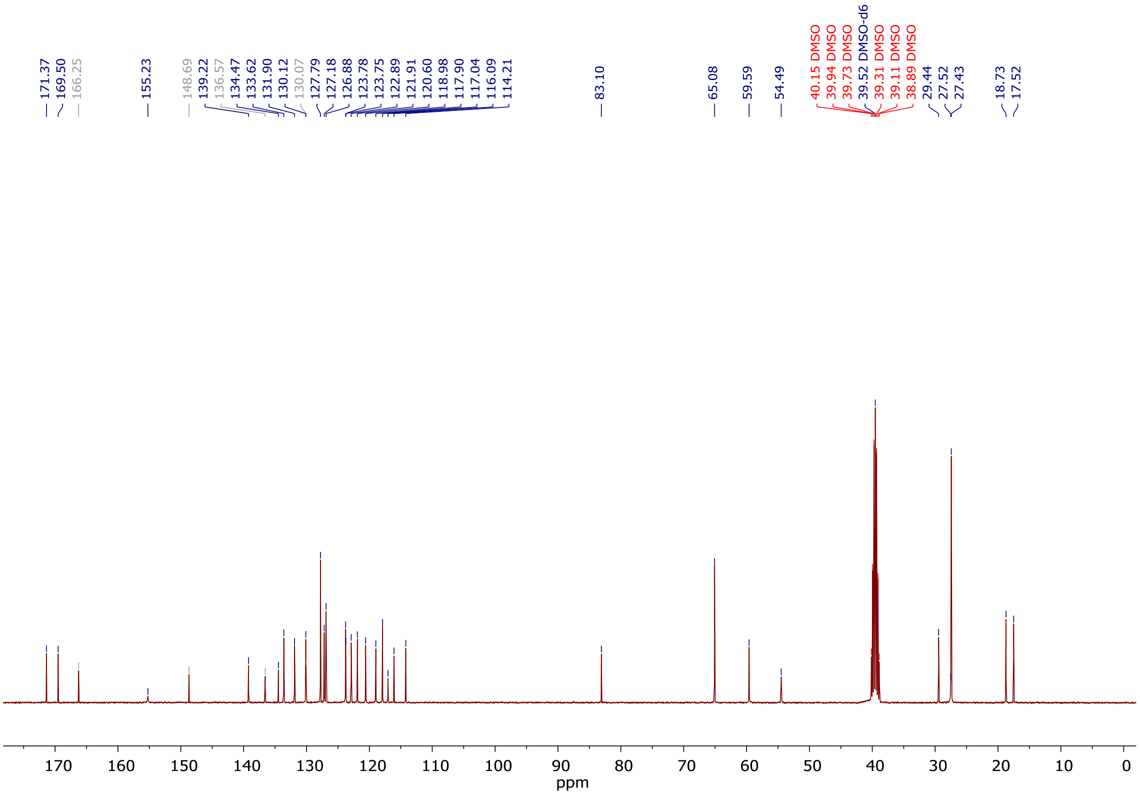

^{1}H NMR (400 MHz, DMSO-d_6, 343 K)

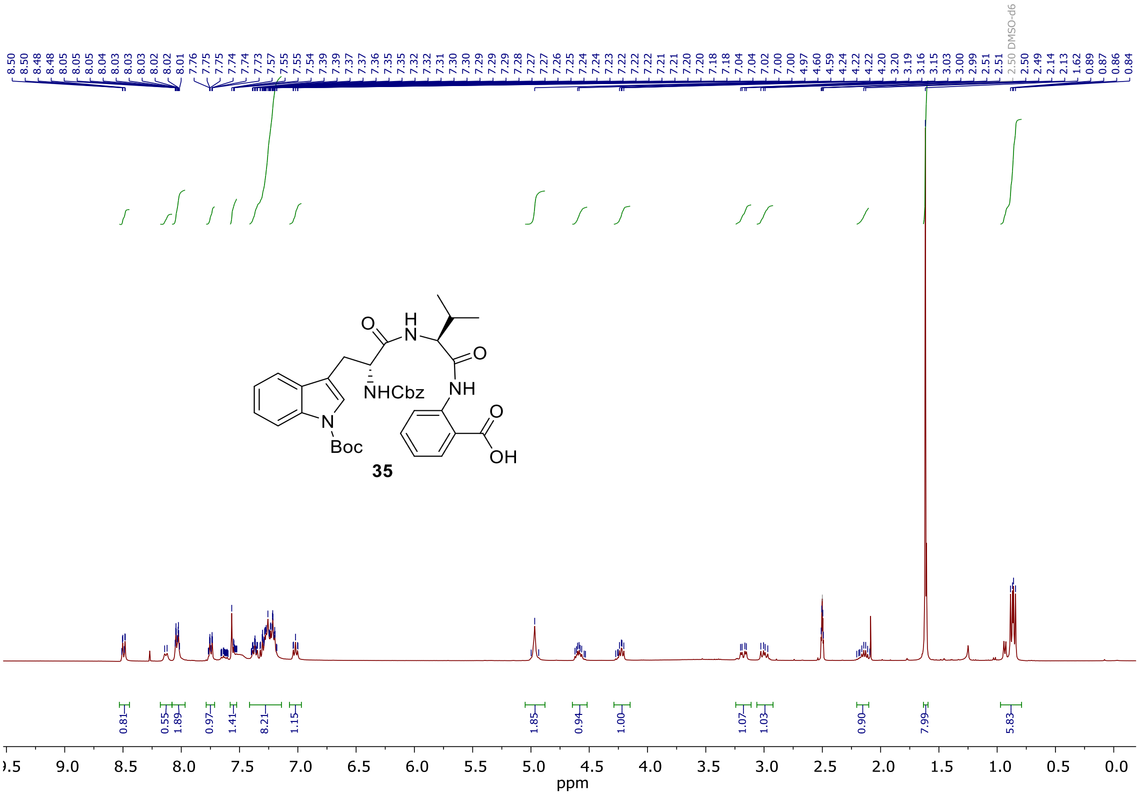

^{13}C{^{1}H} NMR (101 MHz, DMSO-d_6, 343 K)

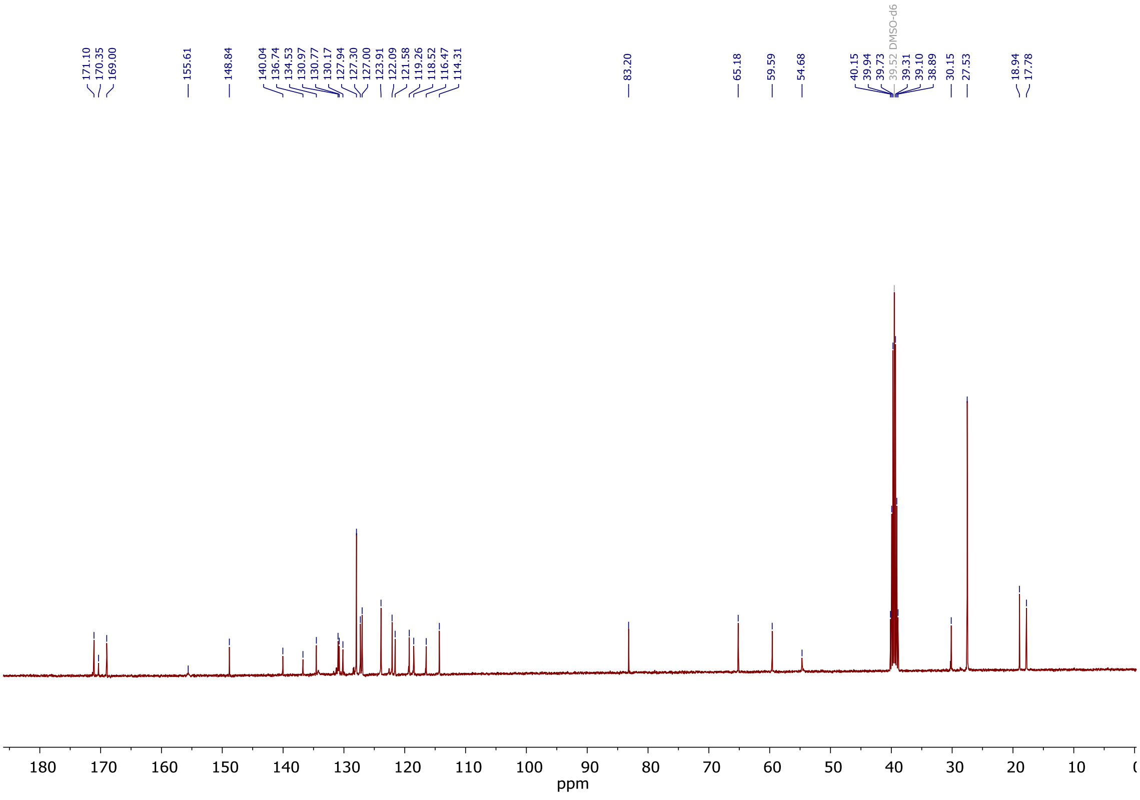

^{1}H NMR (400 MHz, DMSO-*d*6, 343 K)

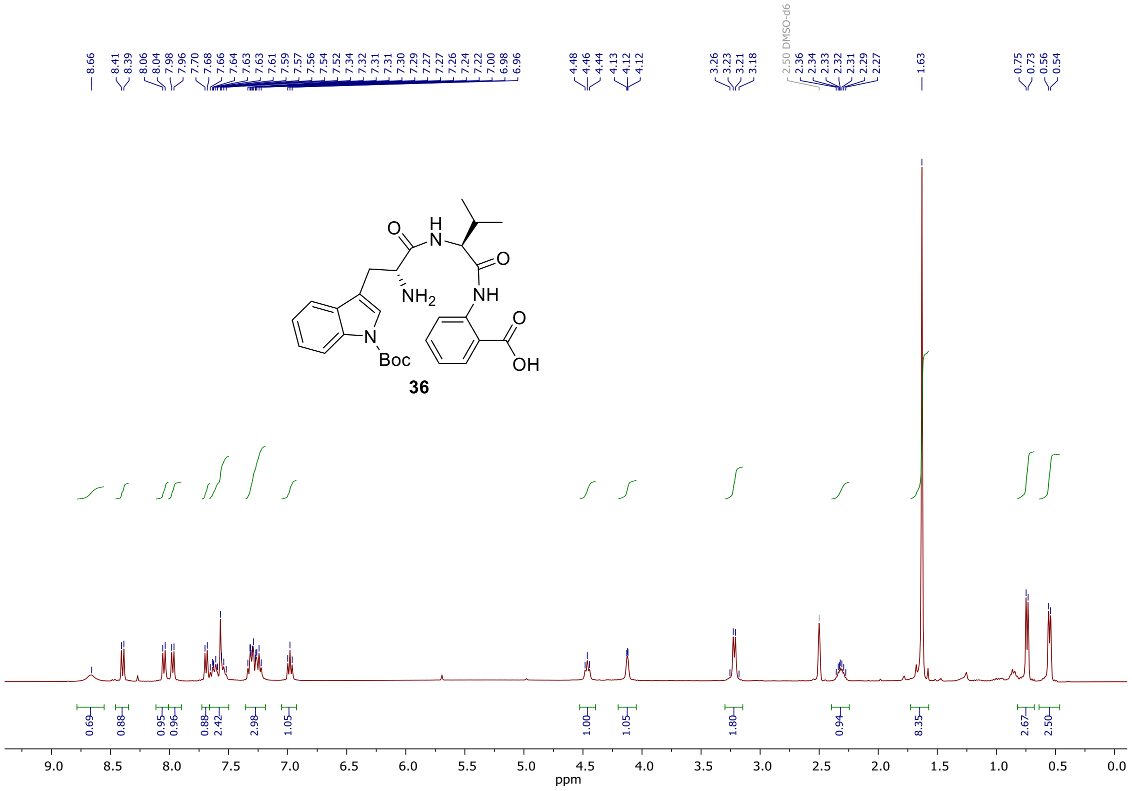

^{13}C{^{1}H} NMR (101 MHz, DMSO-*d*6, 343 K)

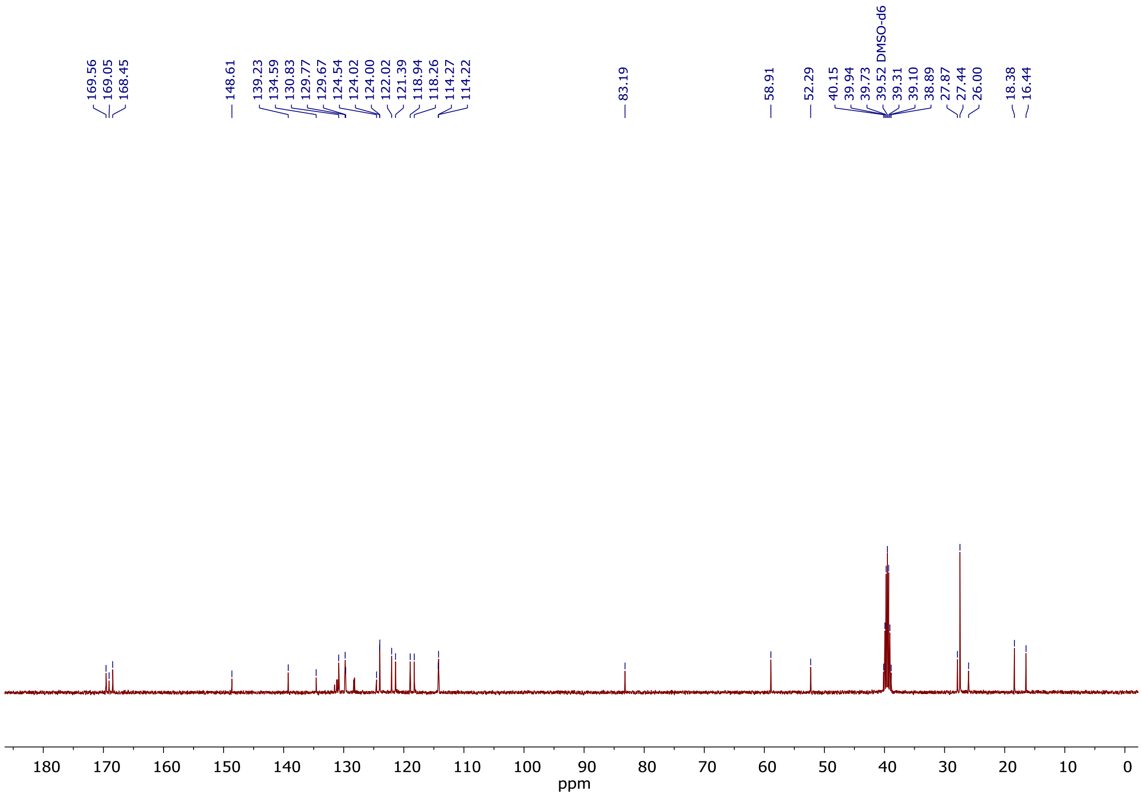

^{1}H NMR (400 MHz, $CDCl_3$, 328 K)

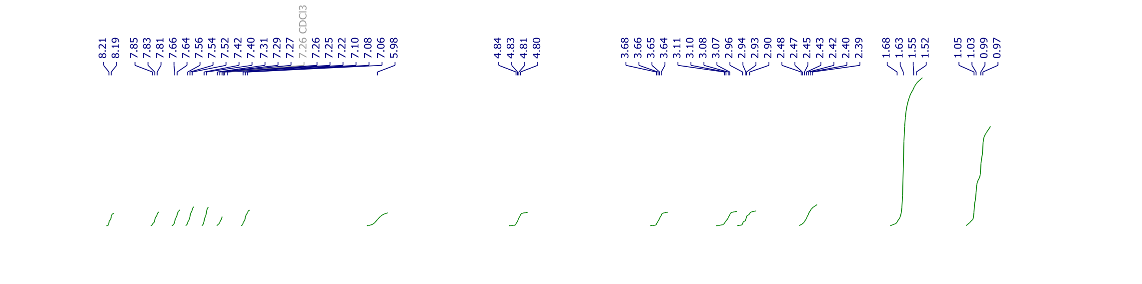

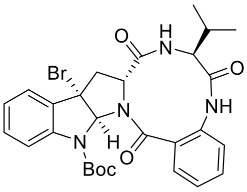

exo-**38**

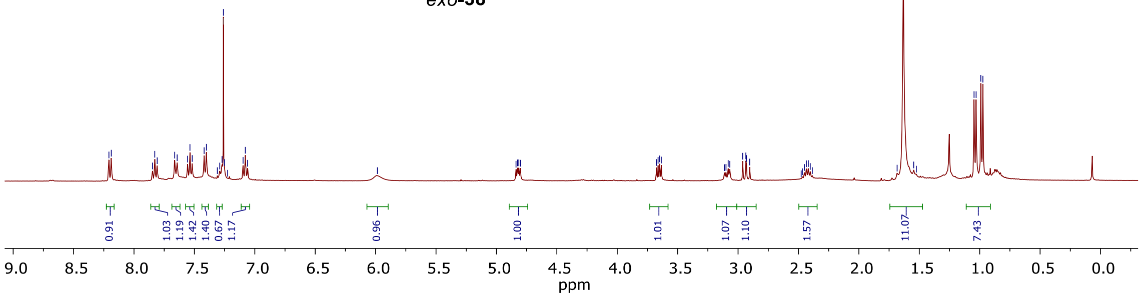

^{13}C{^{1}H} NMR (101 MHz, $CDCl_3$, 328 K)

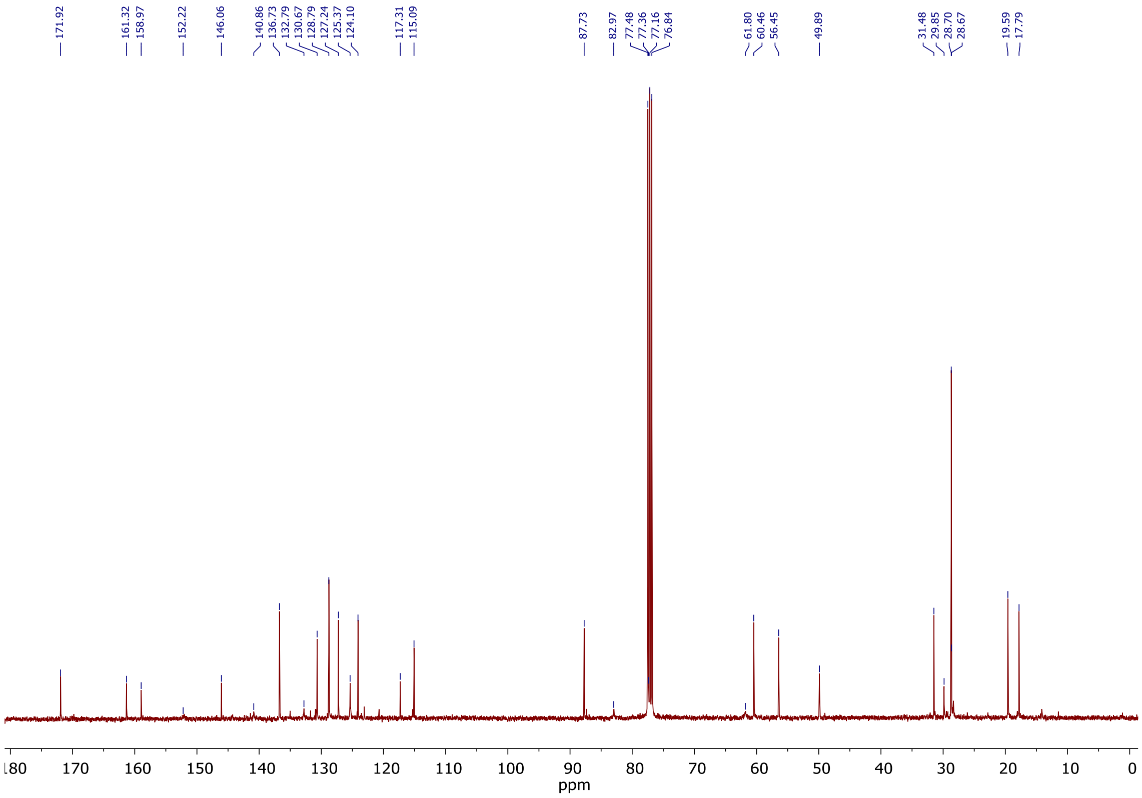

DEPT-135 (101 MHz, $CDCl_3$, 328 K)

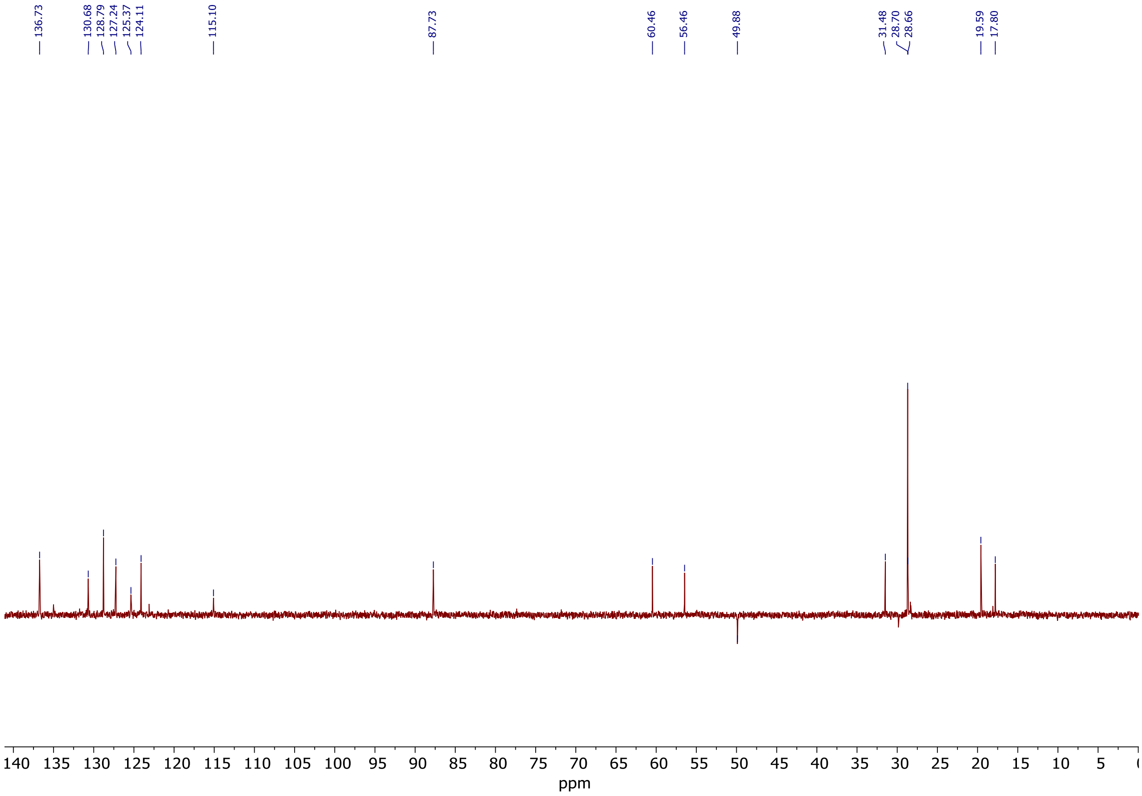

HSQC (101 MHz, $CDCl_3$, 328 K)

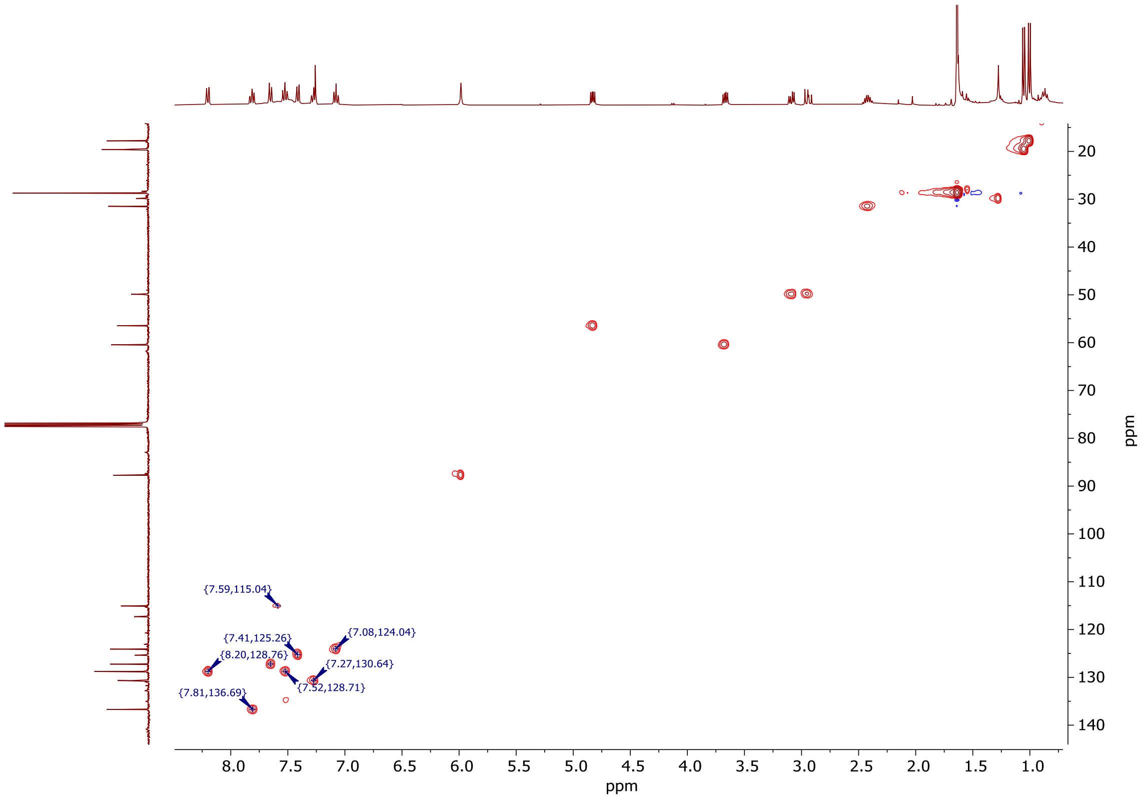

HMBC (101 MHz, $CDCl_3$, 328 K)

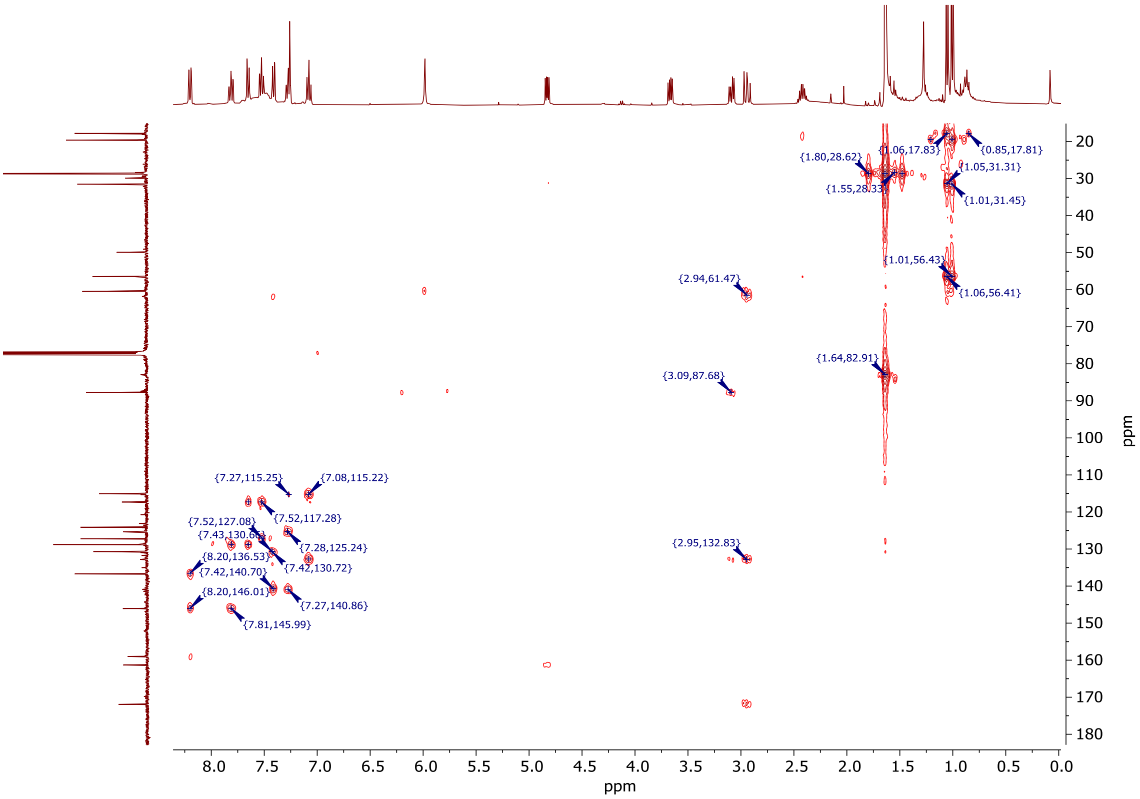

COSY (101 MHz, $CDCl_3$, 328 K)

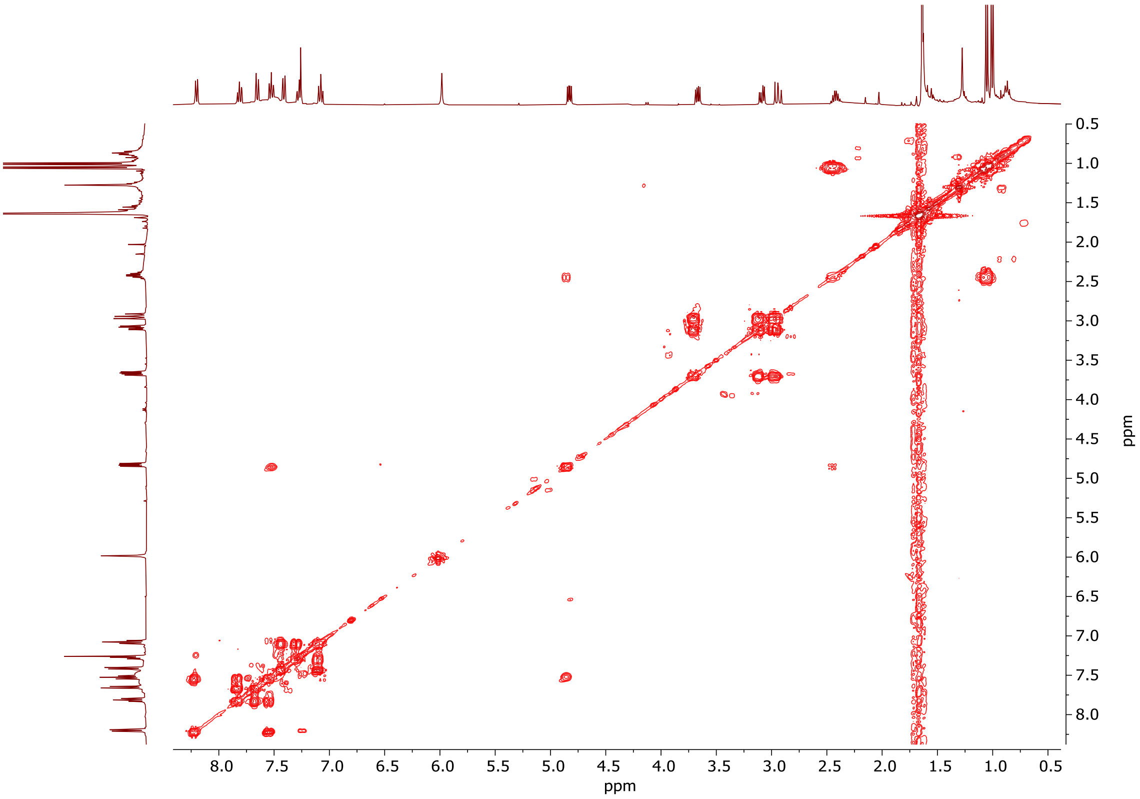

^{1}H NMR (400 MHz, $CDCl_3$, 328 K)

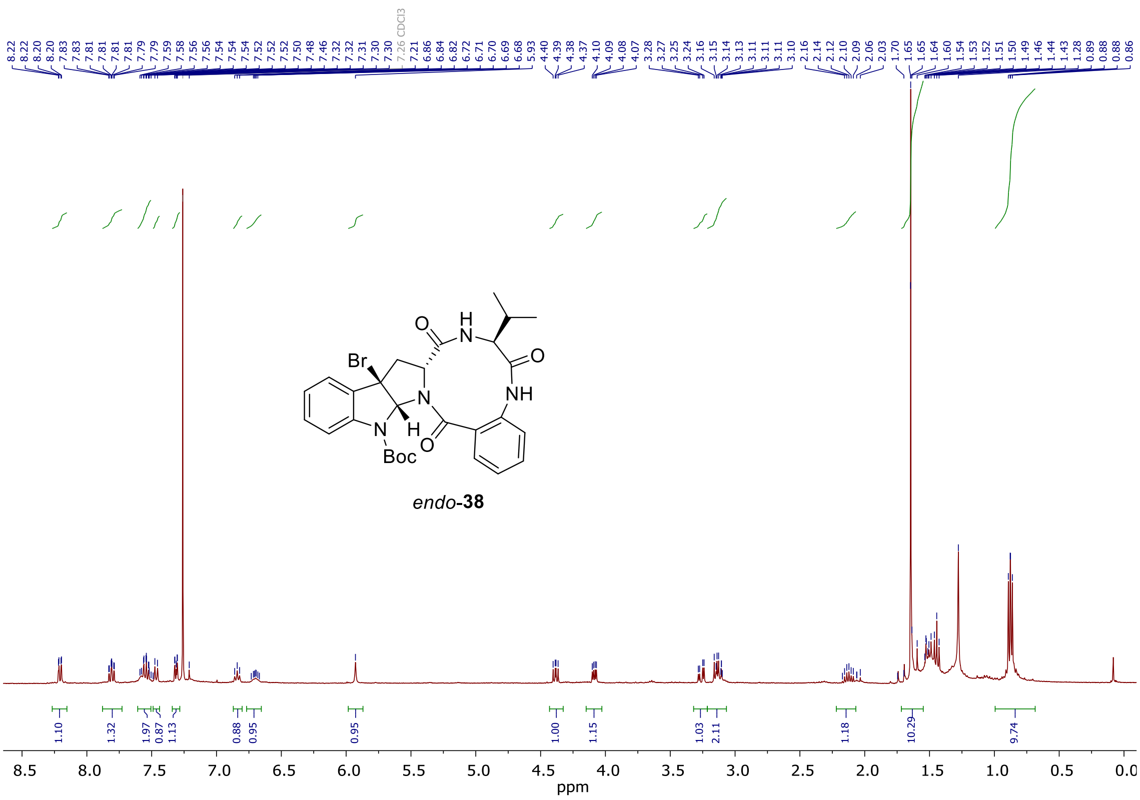

$^{13}C\{^{1}H\}$ NMR (101 MHz, $CDCl_3$, 328 K)

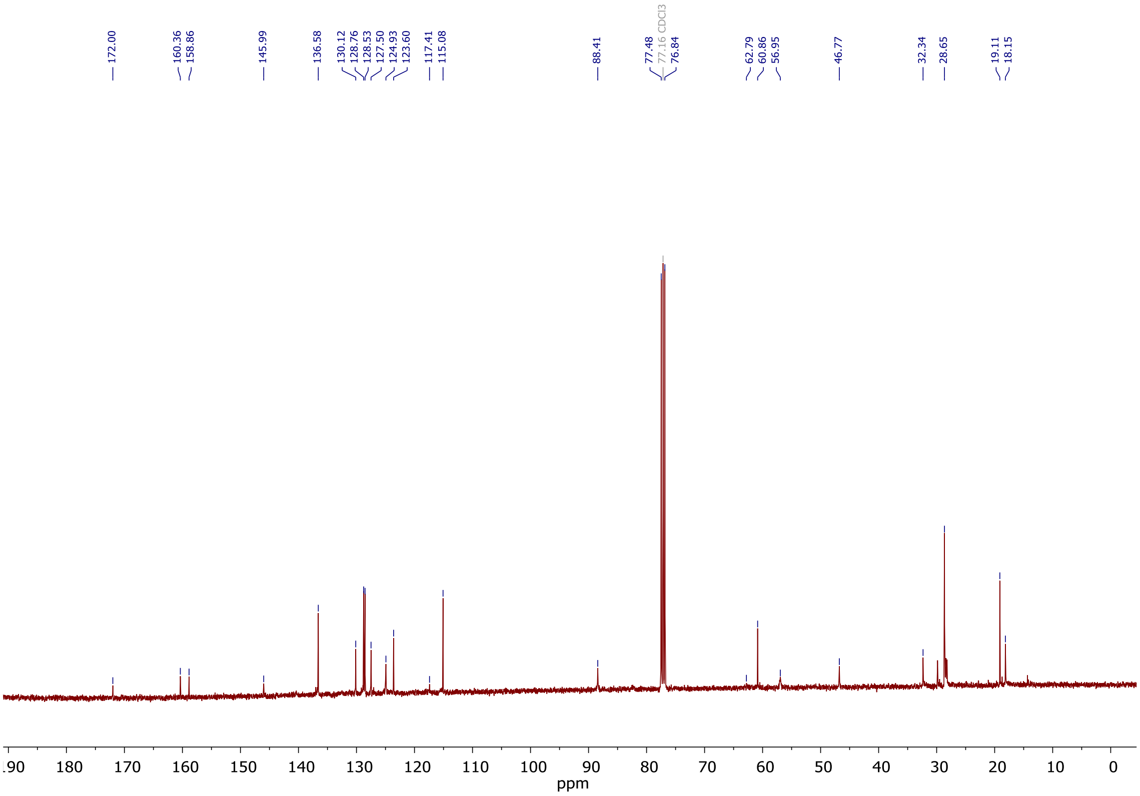

DEPT-135 (101 MHz, $CDCl_3$, 328 K)

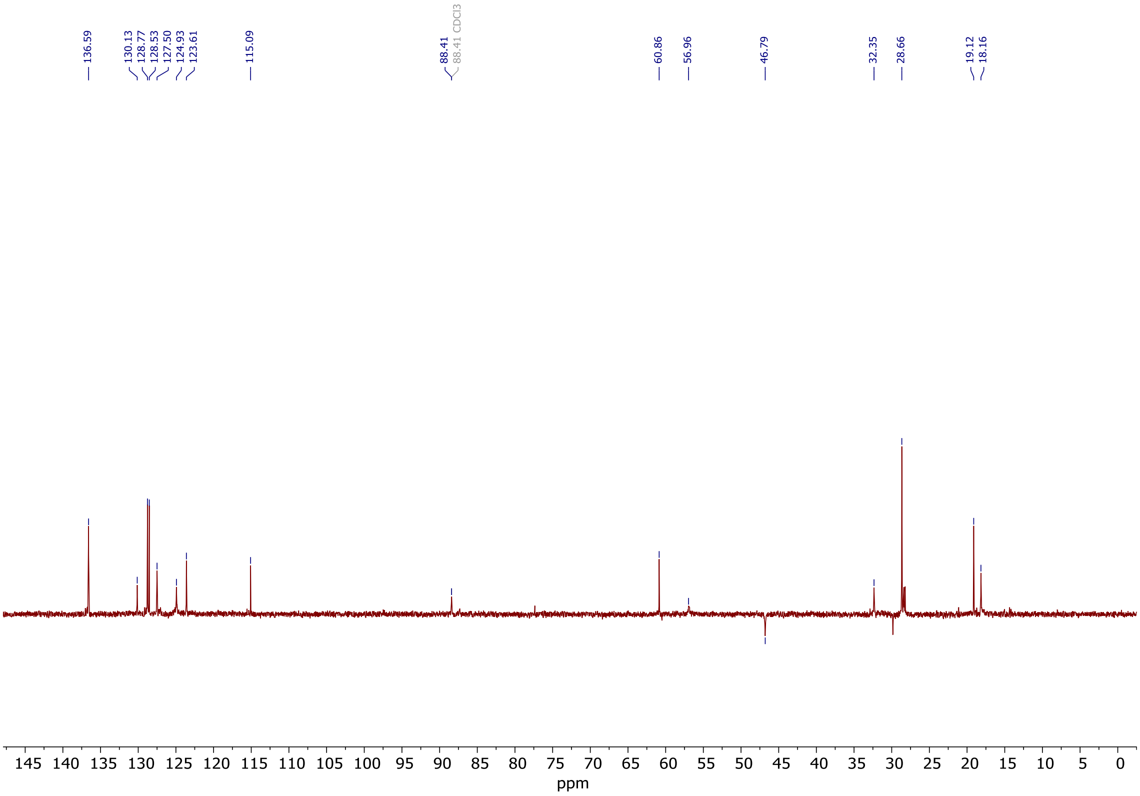

HSQC (101 MHz, $CDCl_3$, 328 K)

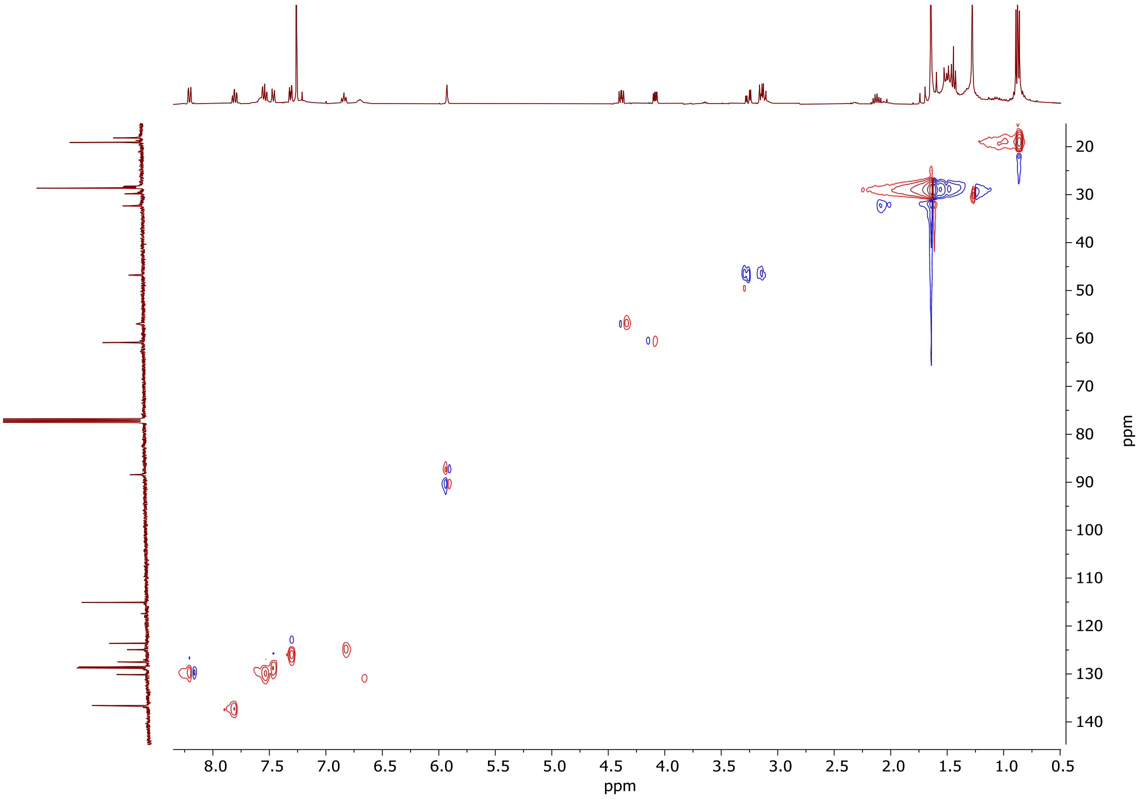

HMBC (101 MHz, $CDCl_3$, 328 K)

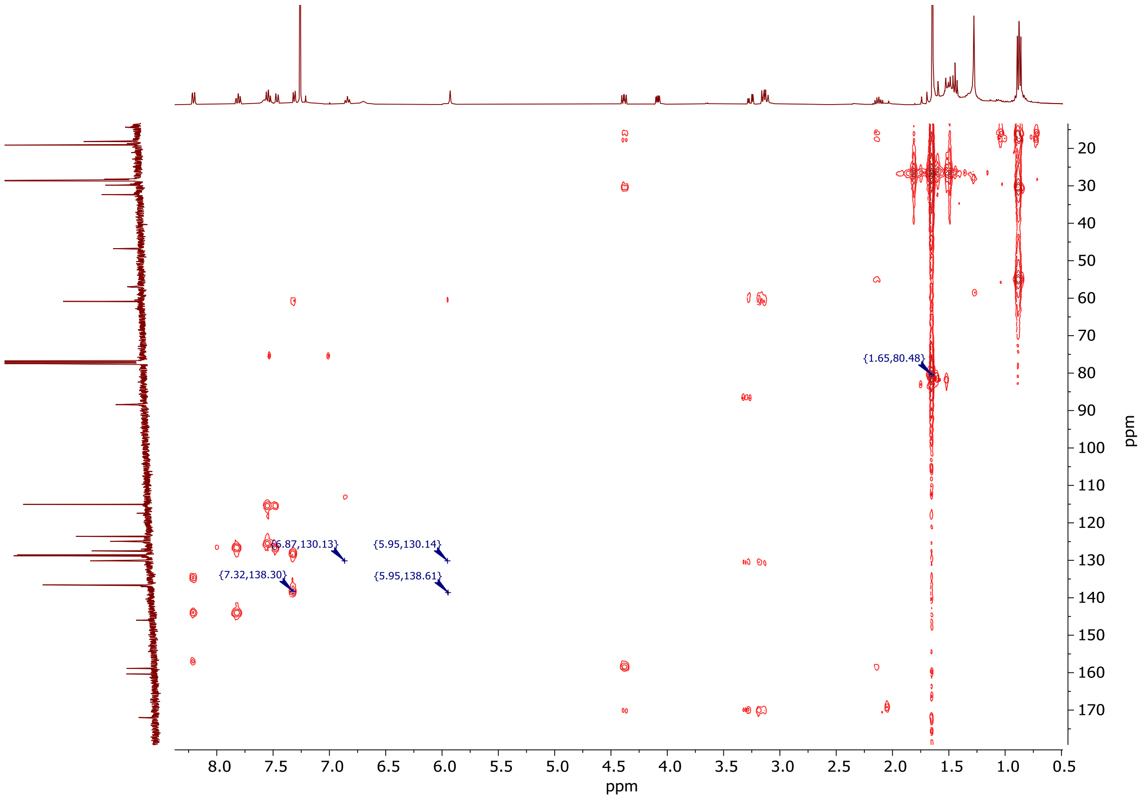

^{1}H NMR (400 MHz, $CDCl_3$, 328 K)

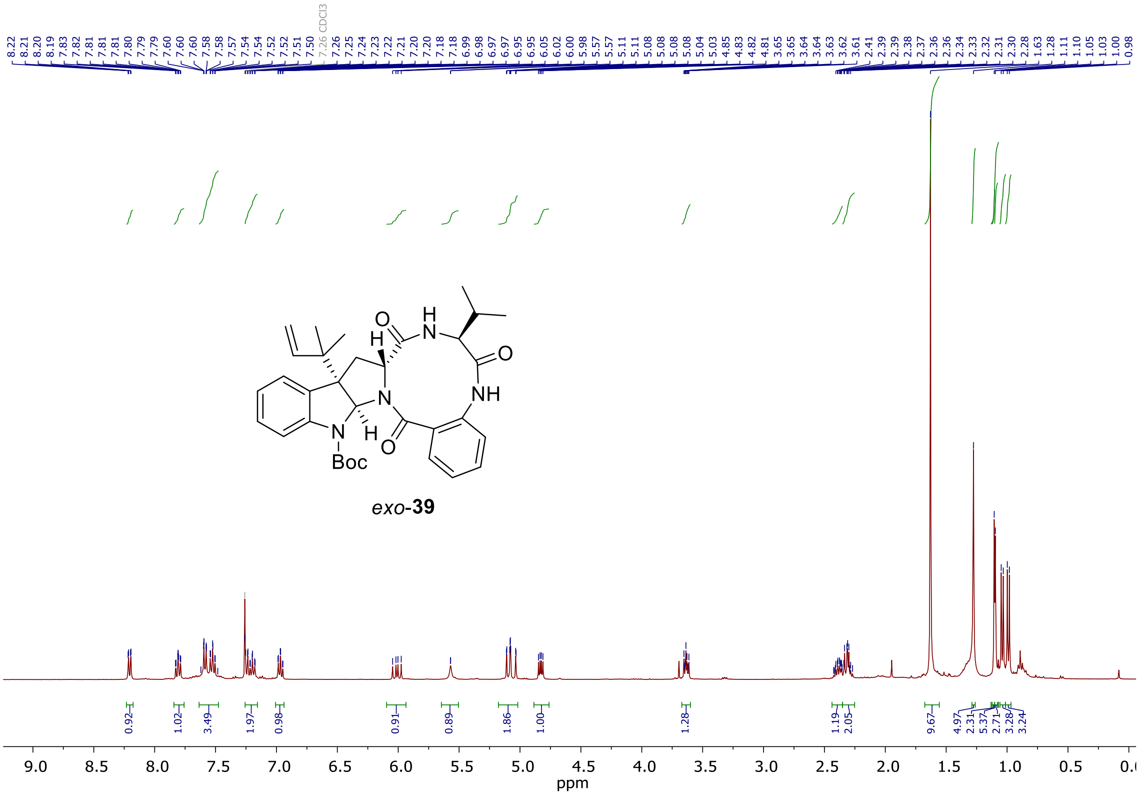

$^{13}C\{^{1}H\}$ NMR (101 MHz, $CDCl_3$, 328 K)

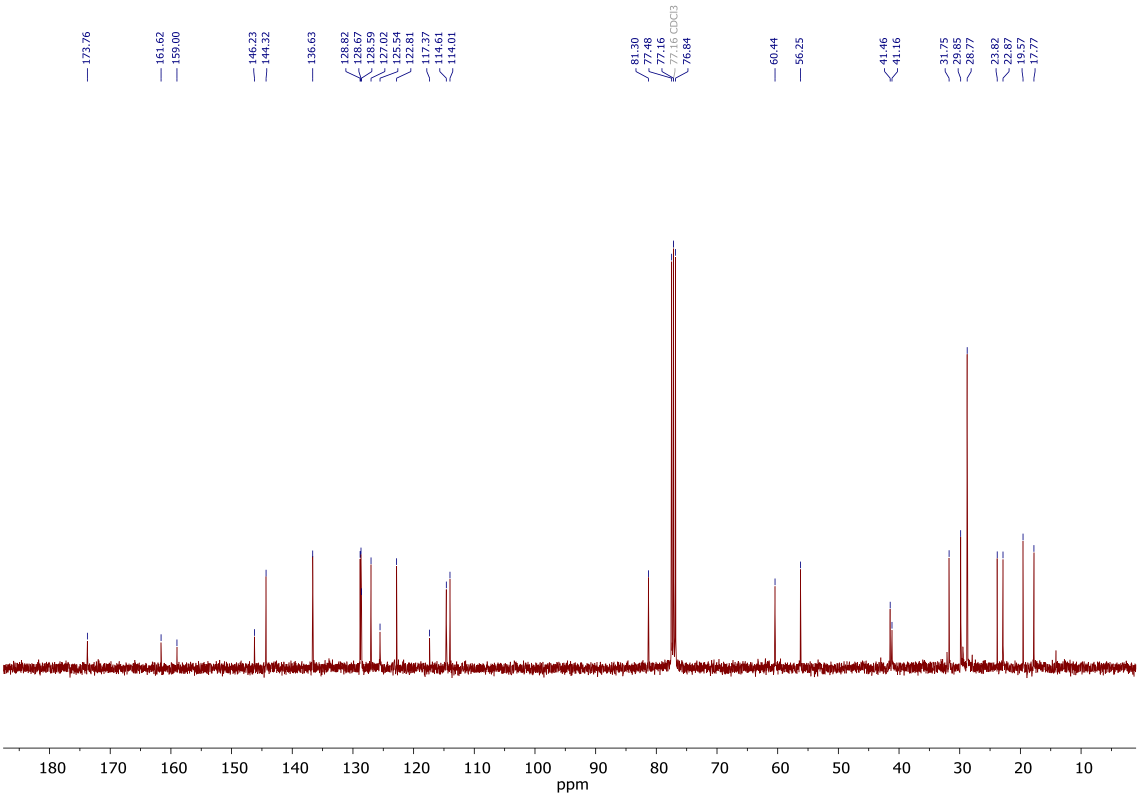

DEPT-135 (101 MHz, $CDCl_3$, 328 K)

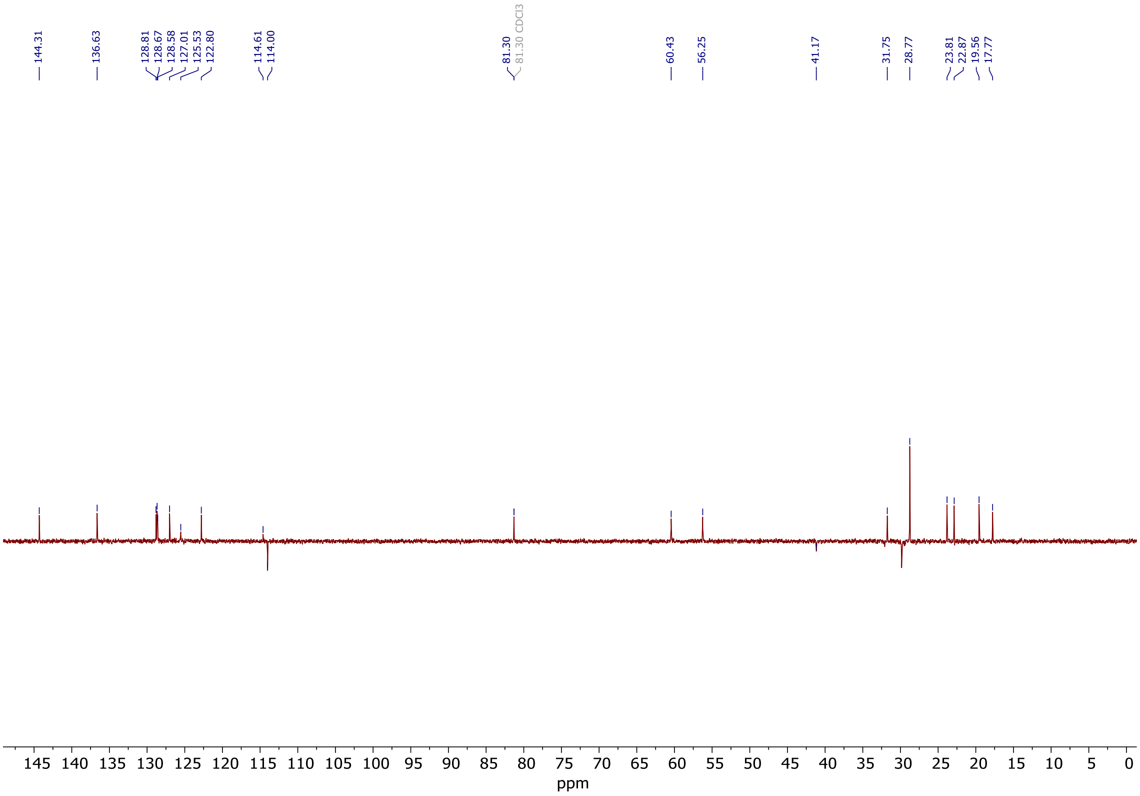

HSQC (101 MHz, $CDCl_3$, 328 K)

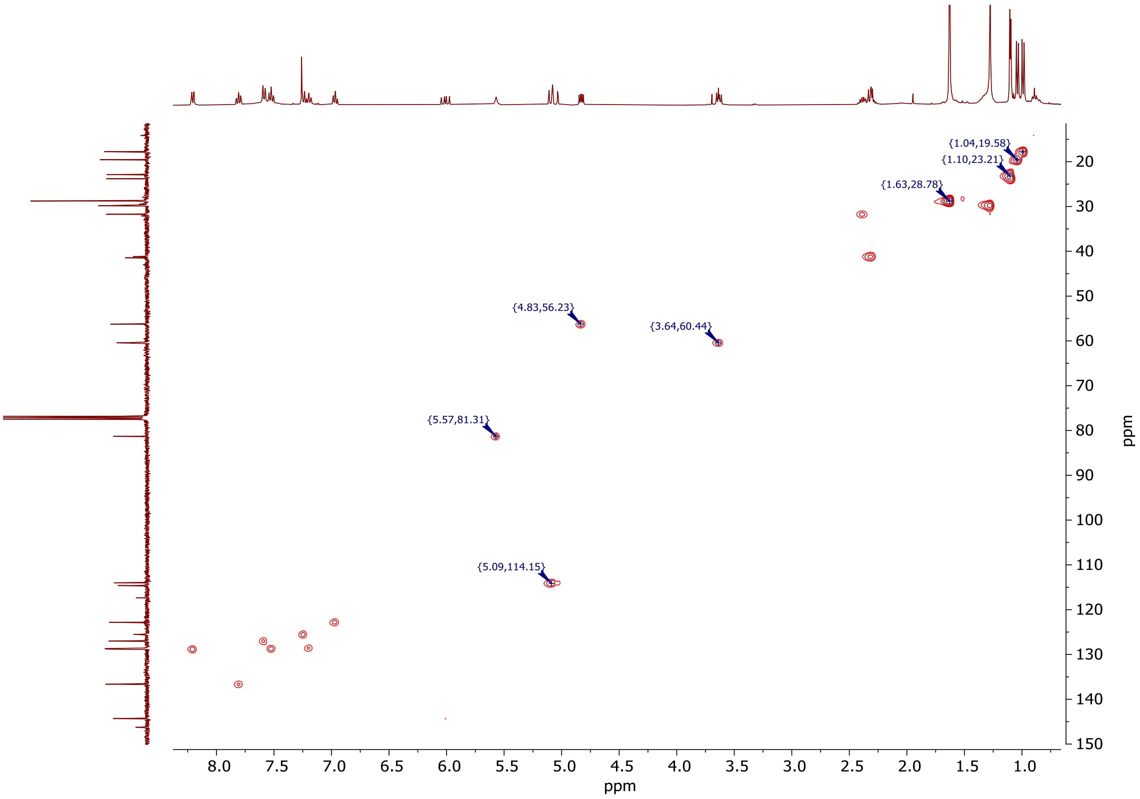

HMBC (101 MHz, $CDCl_3$, 328 K)

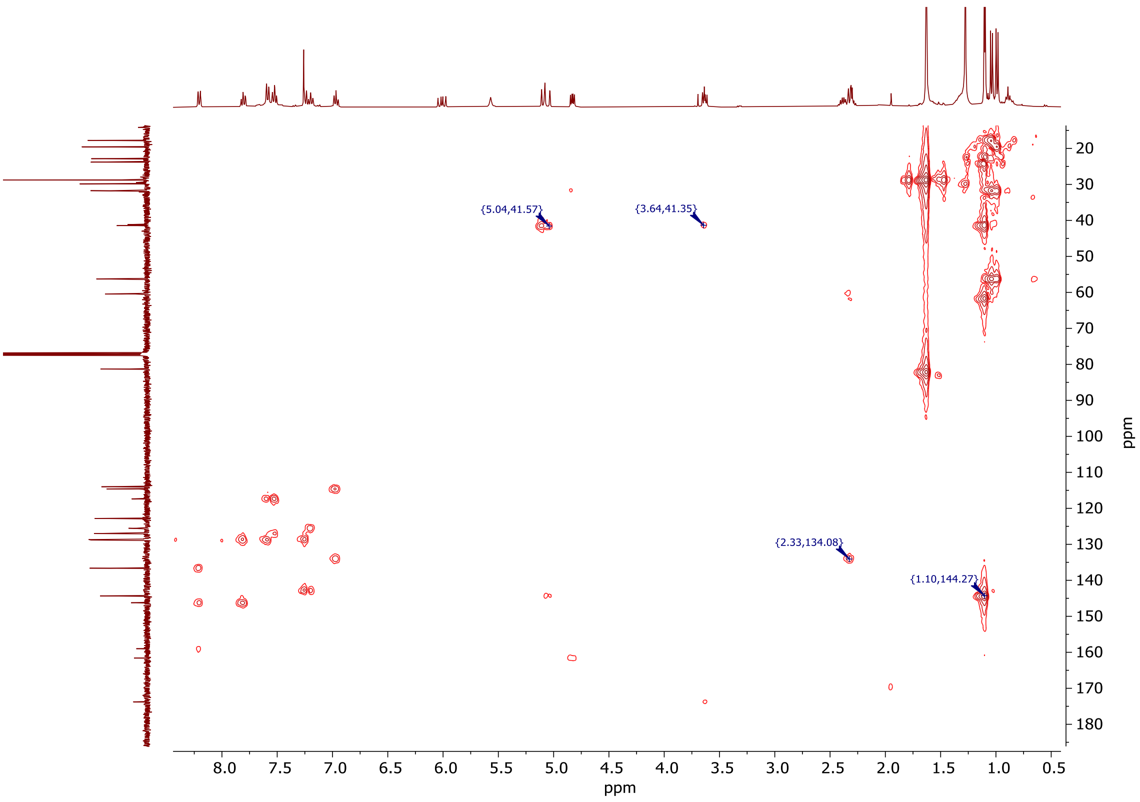

COSY (101 MHz, $CDCl_3$, 328 K)

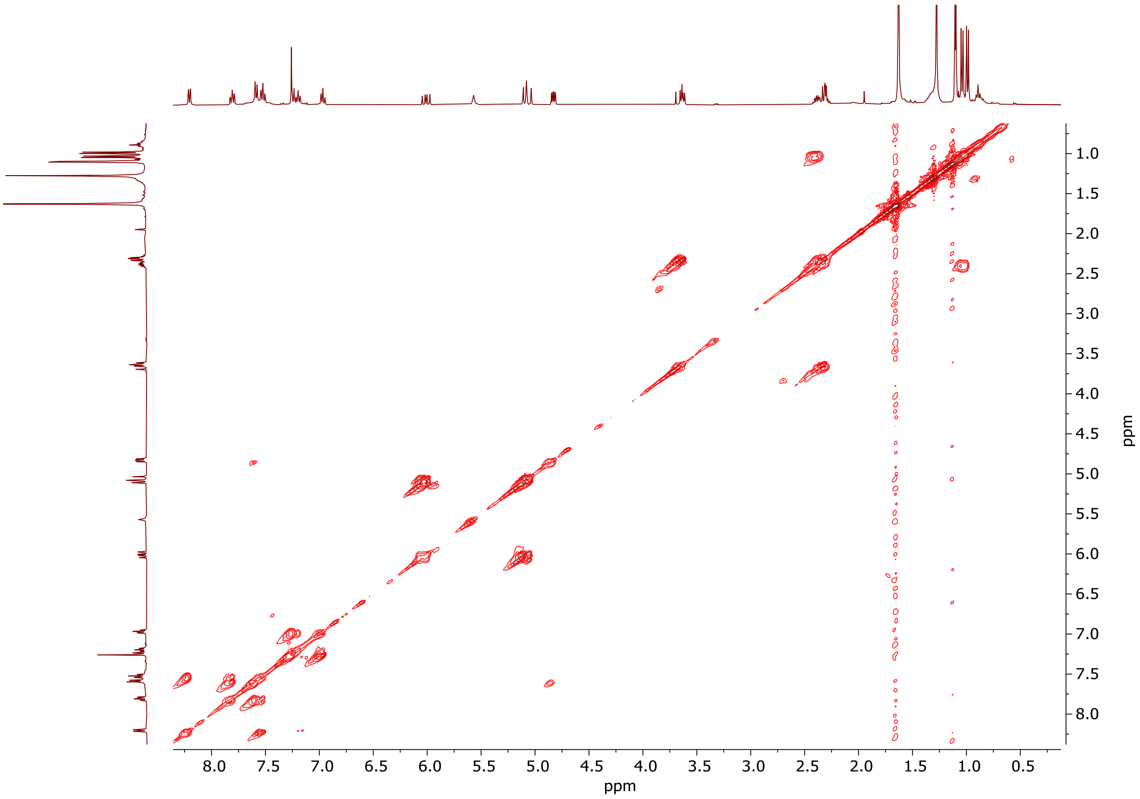

^{1}H NMR (400 MHz, $CDCl_3$)

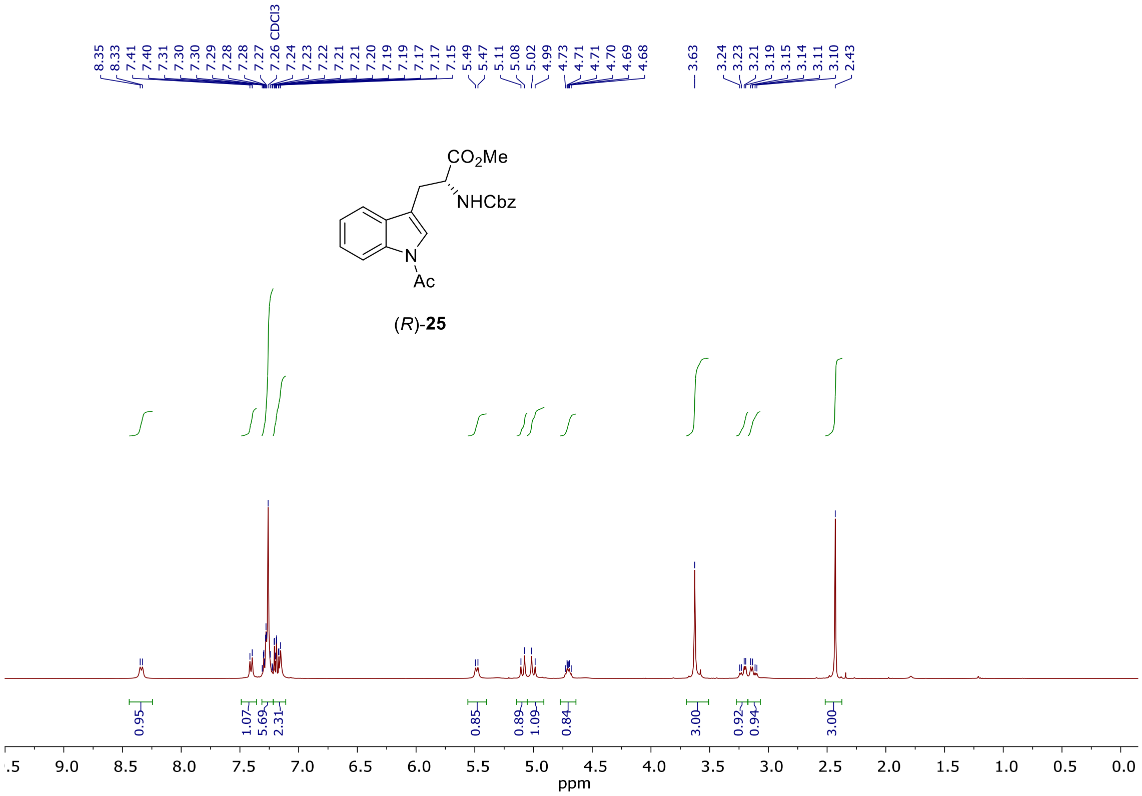

^{13}C{^{1}H} NMR (101 MHz, $CDCl_3$)

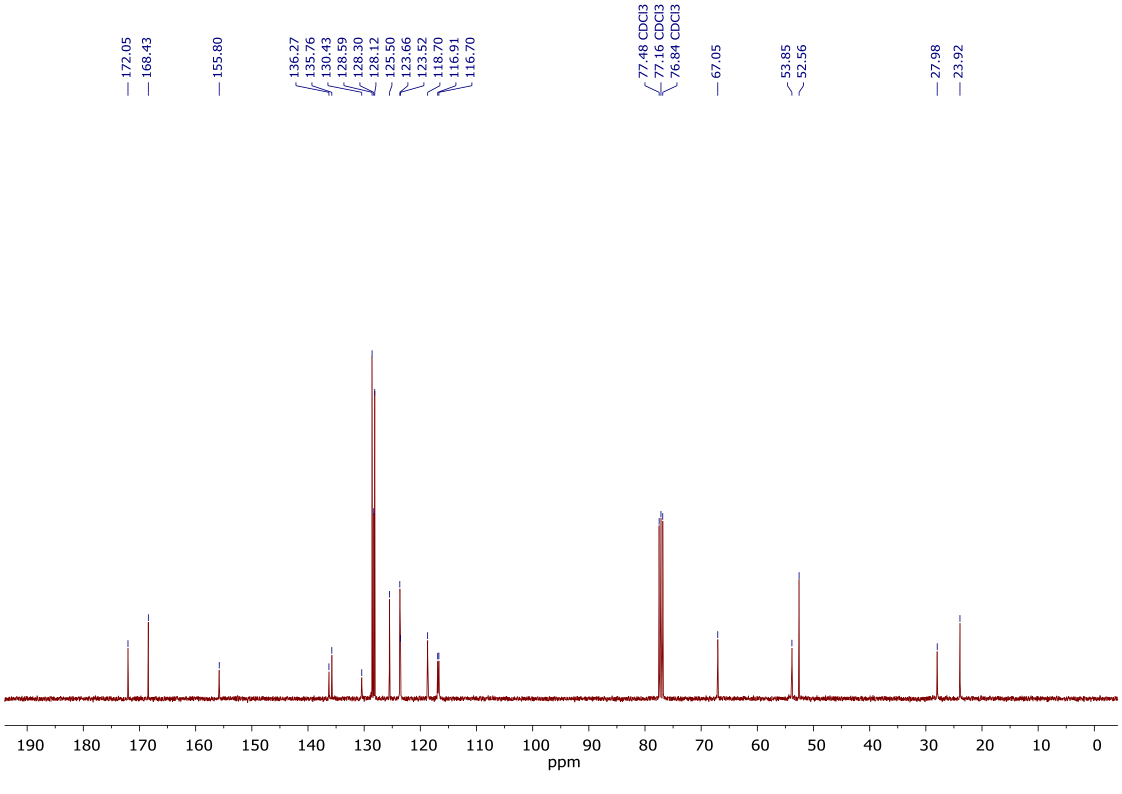

^{1}H NMR (400 MHz, DMSO-d_6, 343 K)

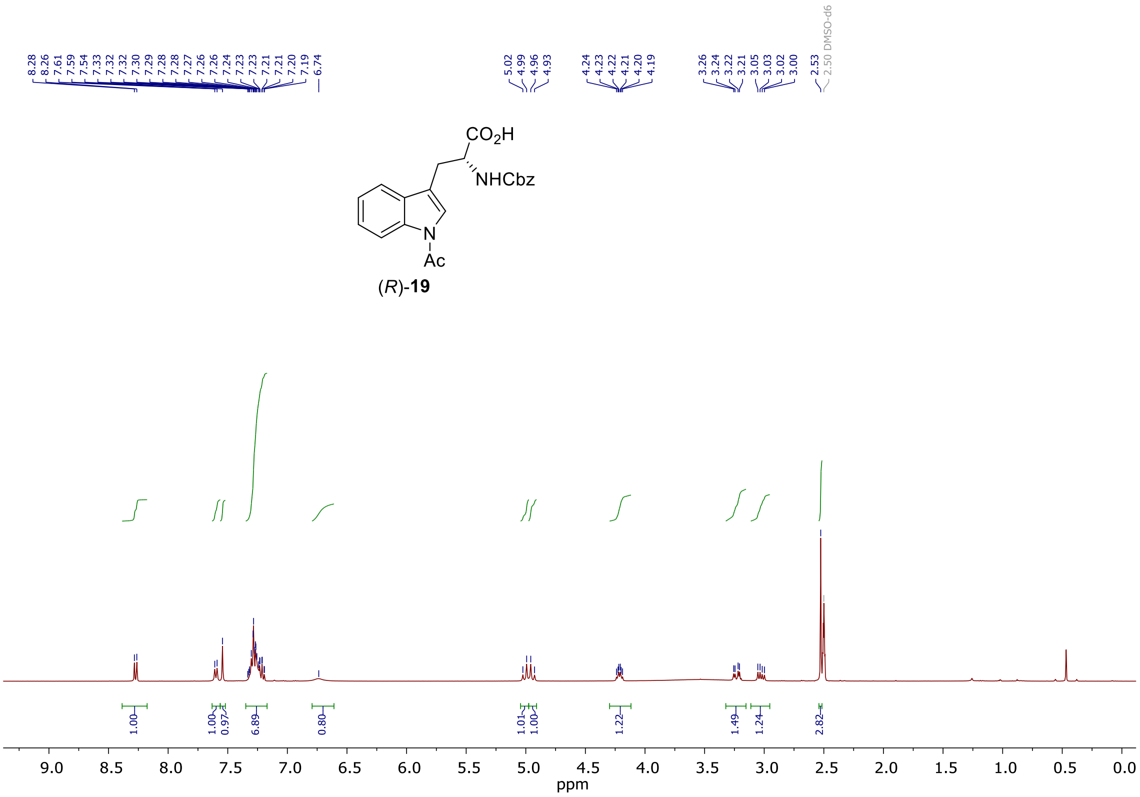

(*R*)-**19**

^{13}C{^{1}H} NMR (101 MHz, DMSO-d_6, 343 K)

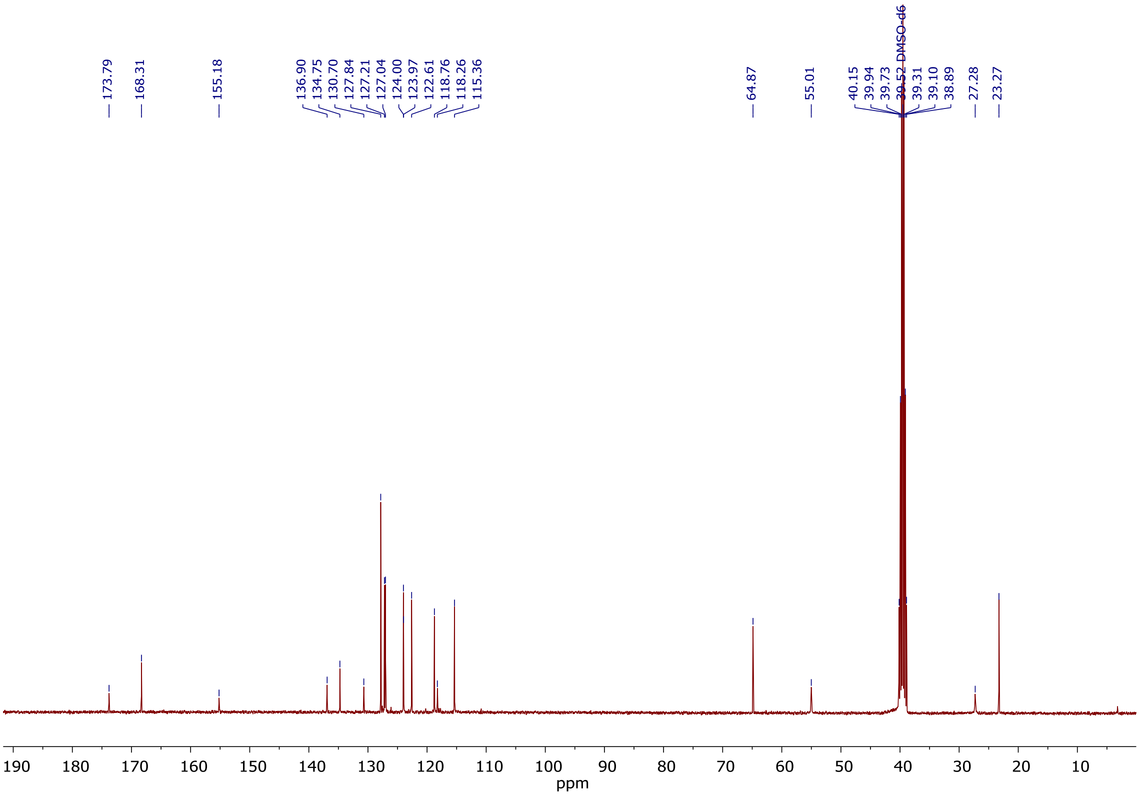

^{1}H NMR (400 MHz, DMSO-d_6, 343 K)

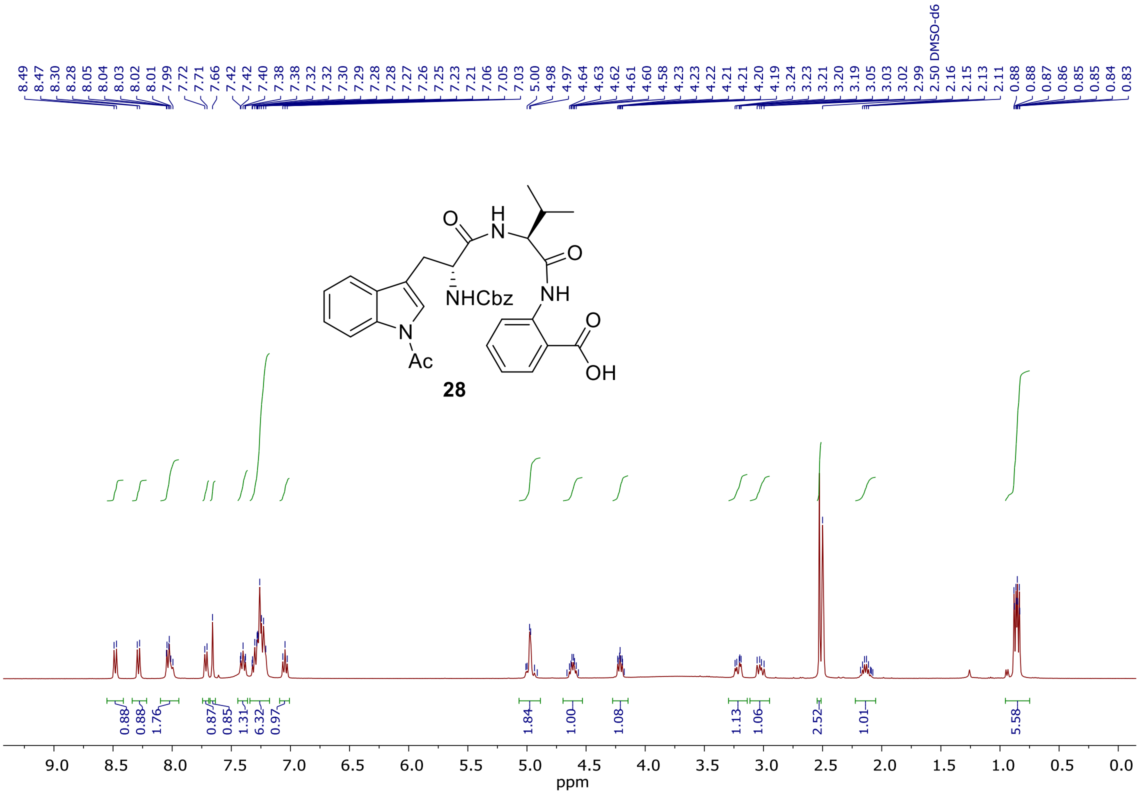

^{13}C{^{1}H} NMR (101 MHz, DMSO-d_6, 343 K)

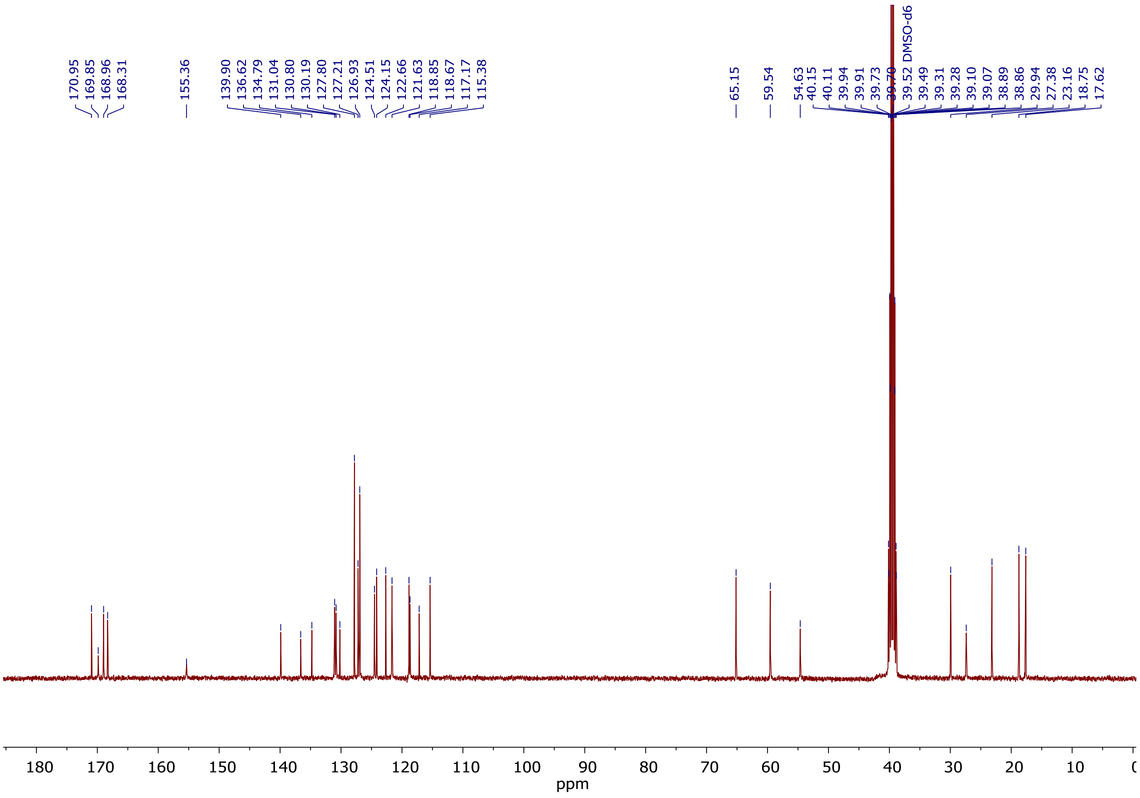

^{1}H NMR (400 MHz, DMSO-d_6, 343 K)

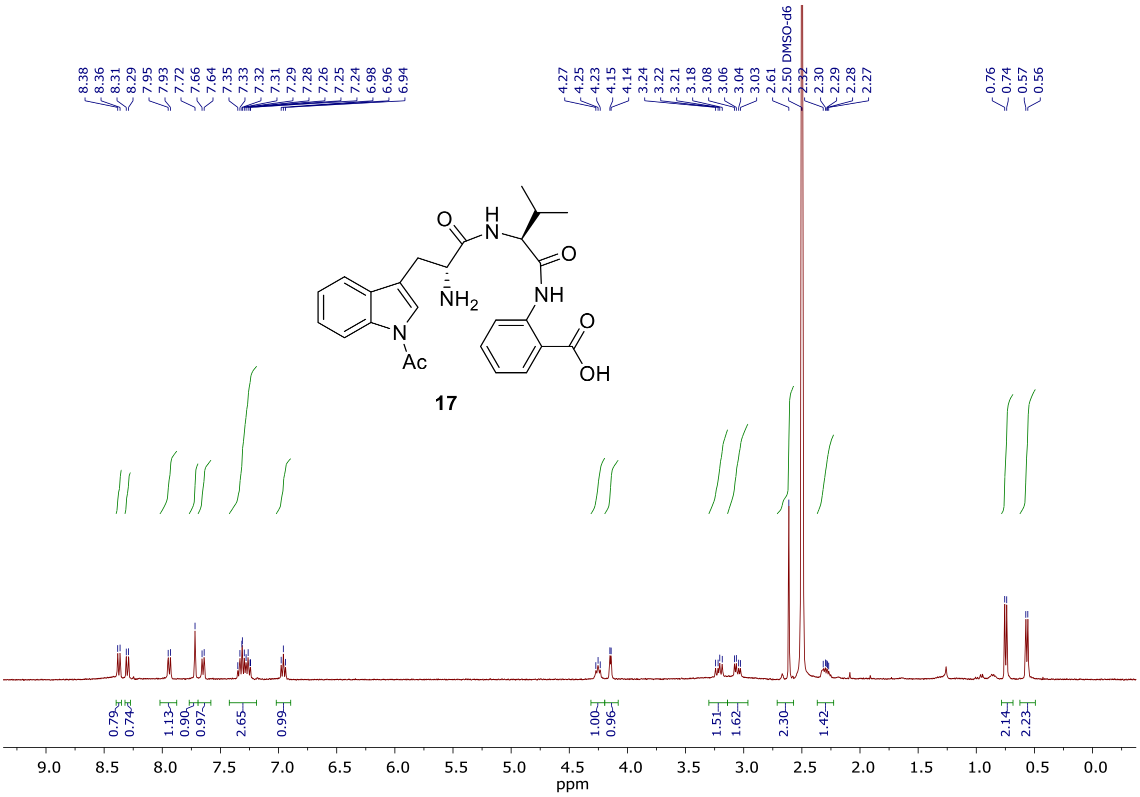

^{13}C{^{1}H} NMR (101 MHz, DMSO-d_6, 343 K)

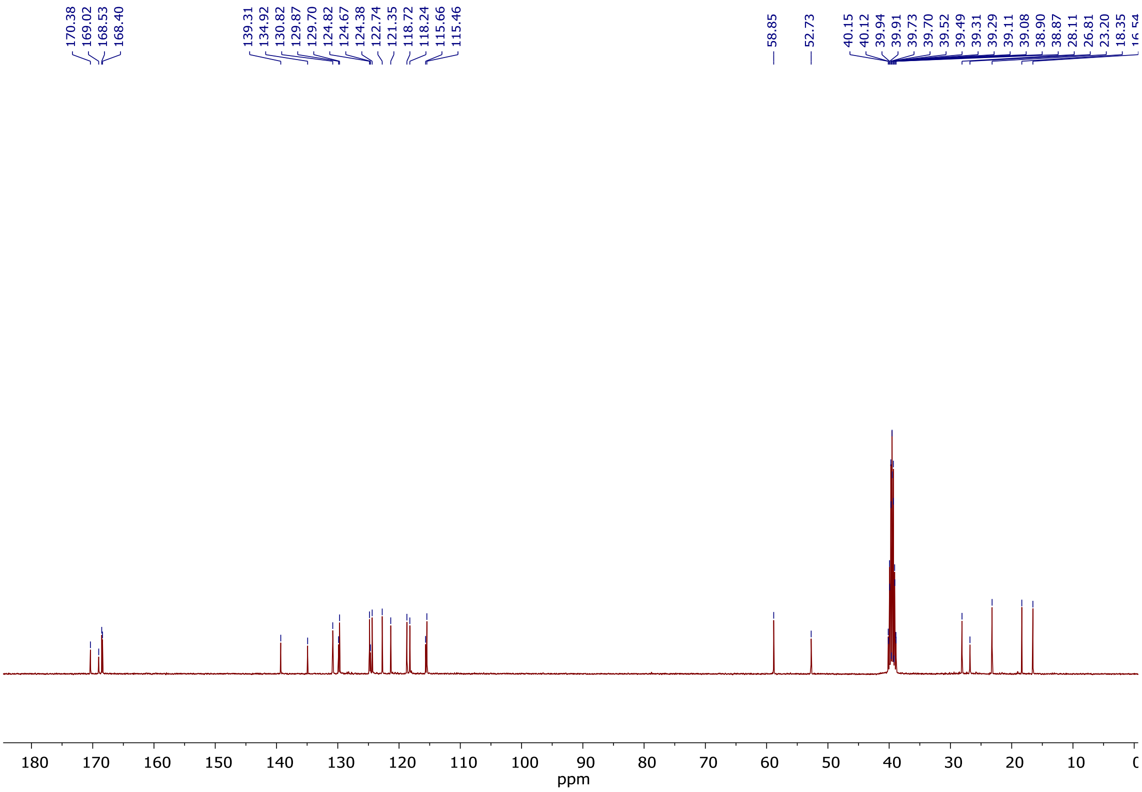

^{1}H NMR (400 MHz, $CDCl_3$, 323 K)

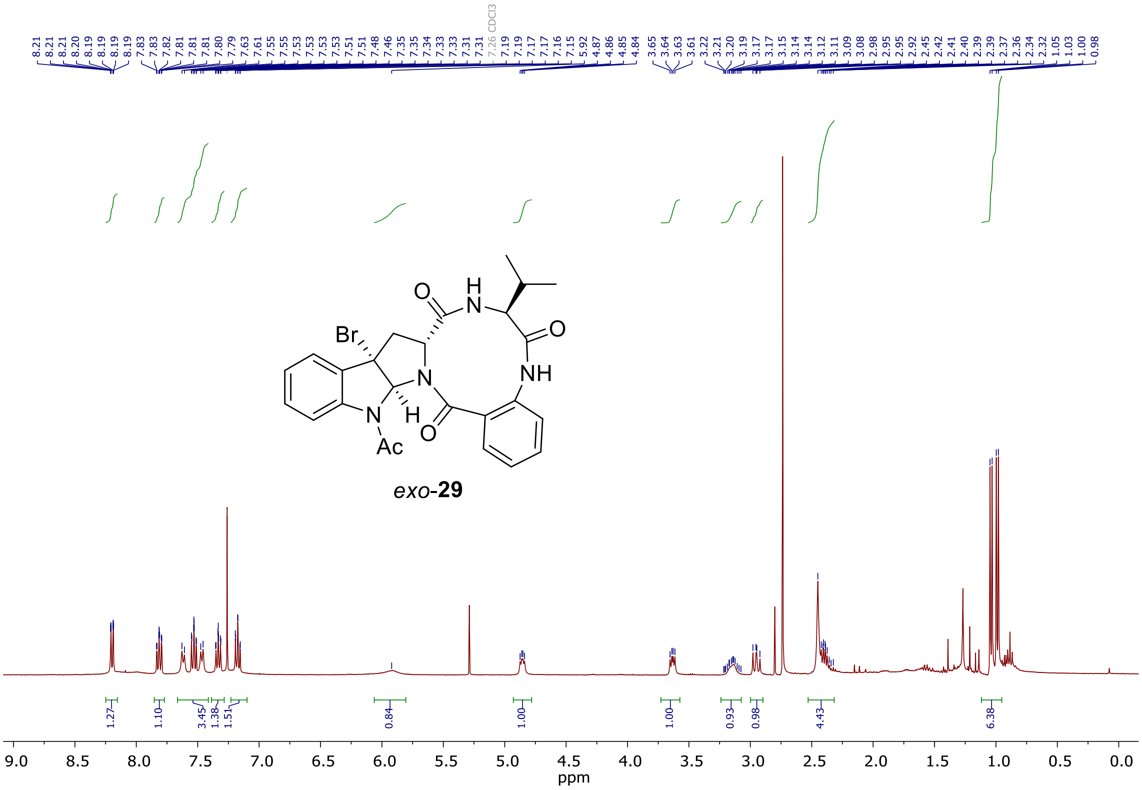

$^{13}C\{^{1}H\}$ NMR (101 MHz, $CDCl_3$, 323 K) (lb = 5)

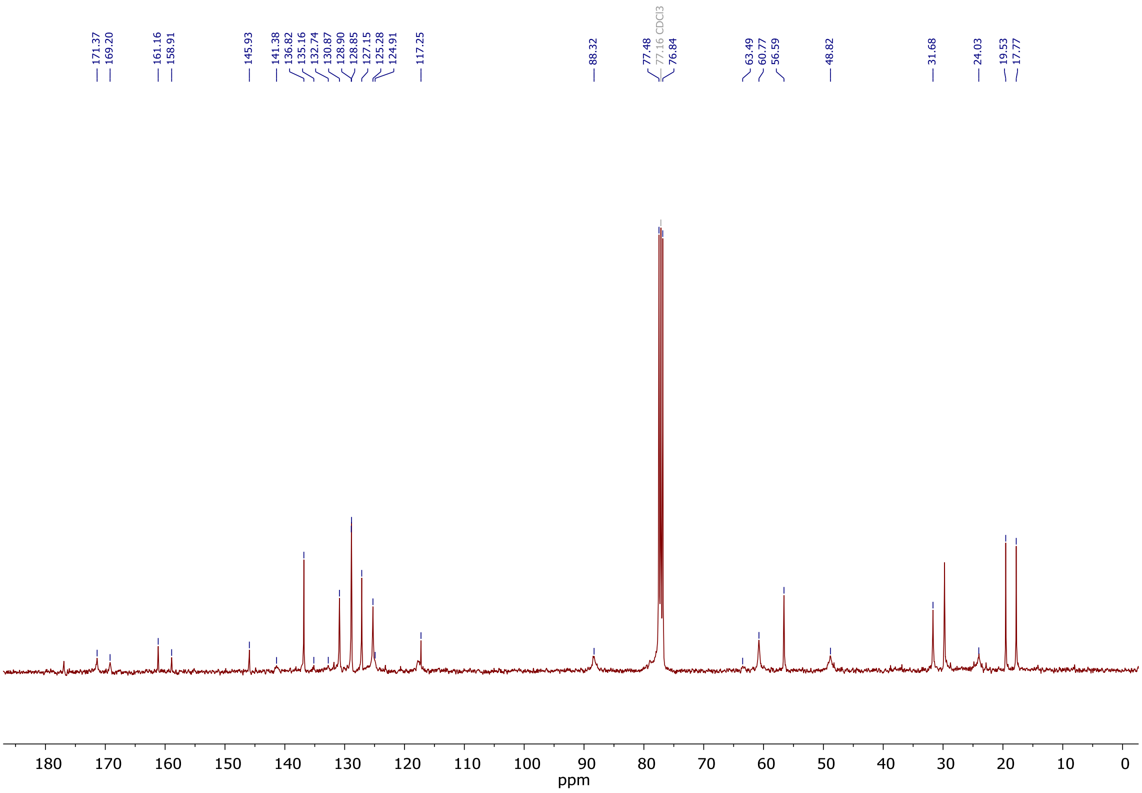

Synthetic novofumigatamide

^{1}H NMR (400 MHz, $CDCl_3$, 298 K)

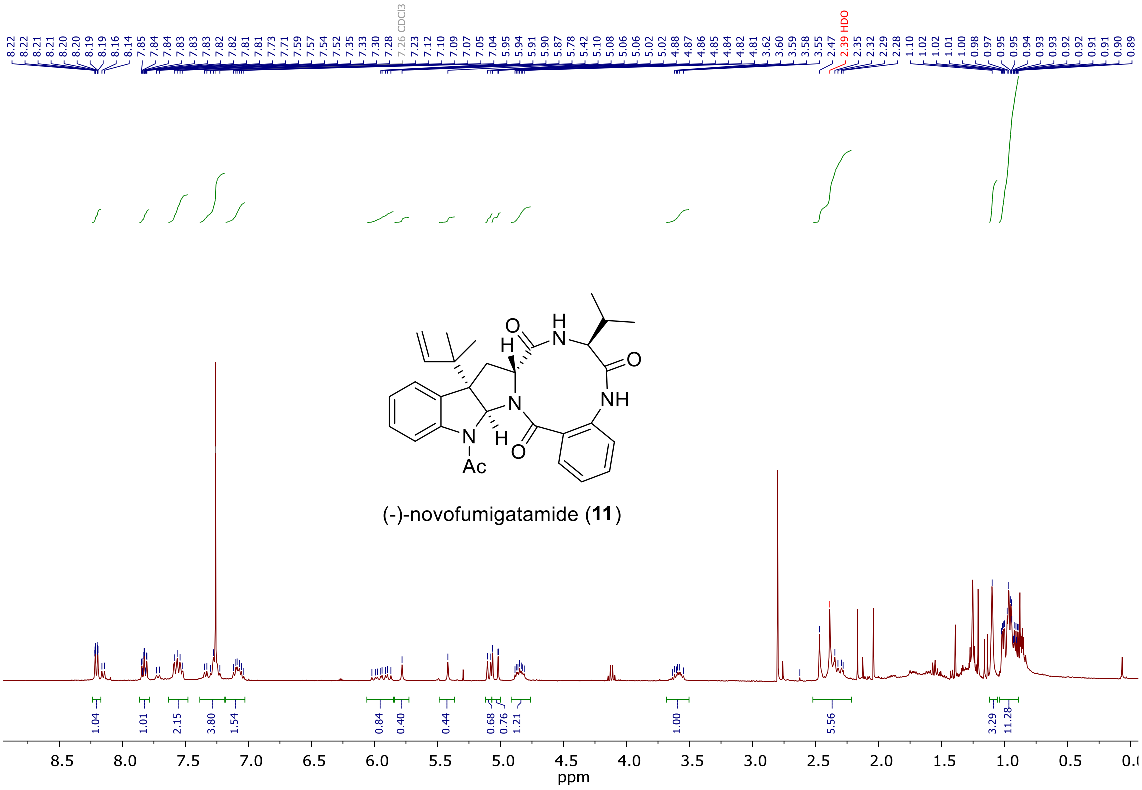

$^{13}C\{^{1}H\}$ NMR (101 MHz, $CDCl_3$, 298 K)

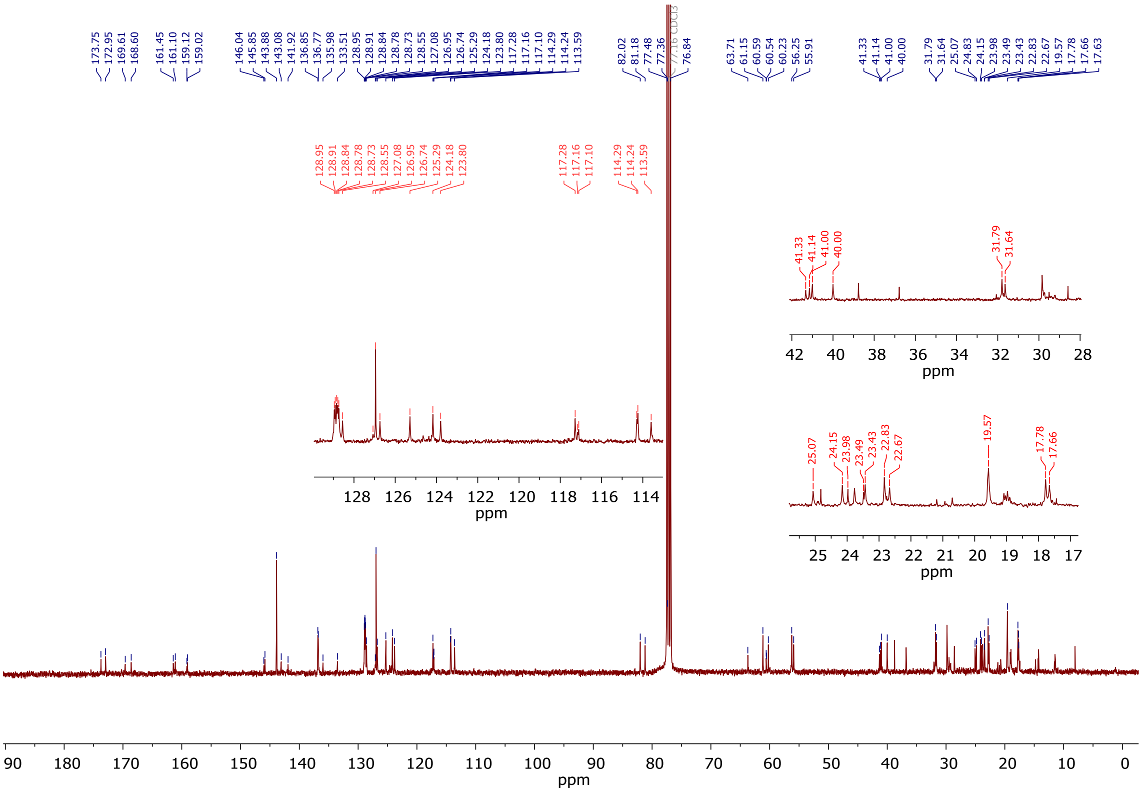

^{1}H NMR (400 MHz, $CDCl_3$, 328 K)

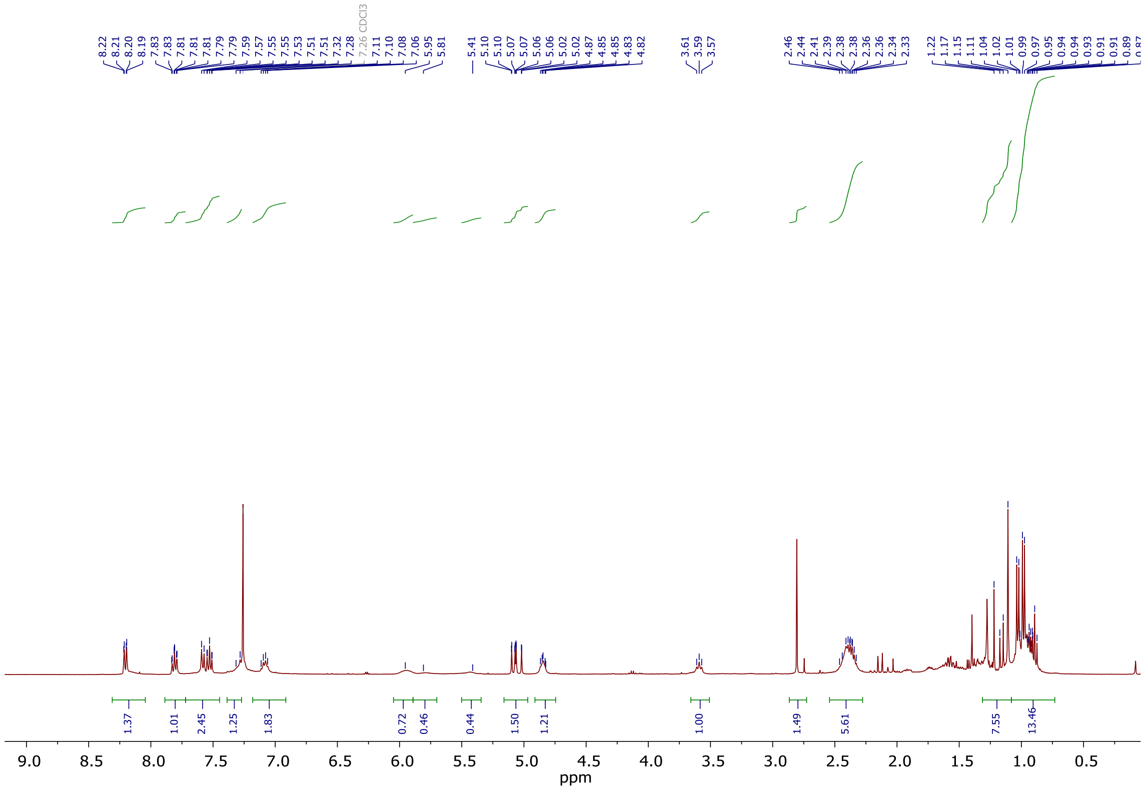

^{1}H NMR (400 MHz, DMSO-d_6, 298 K)

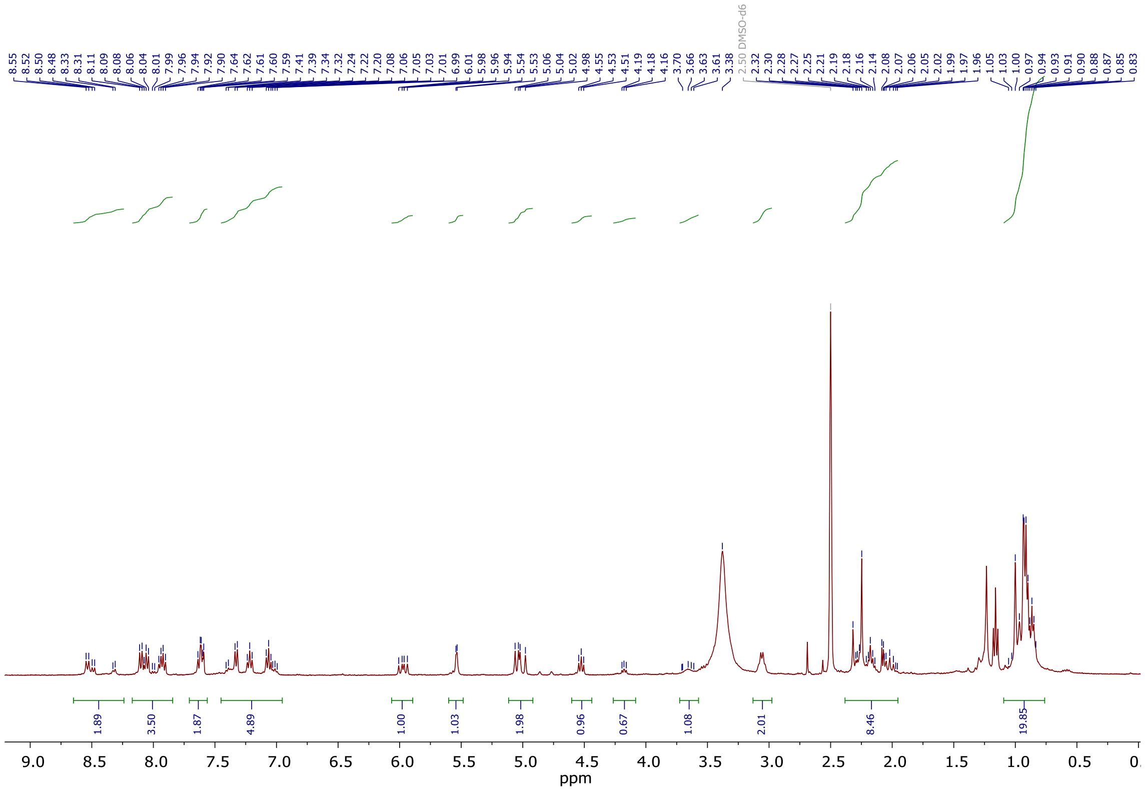

^{1}H NMR (400 MHz, DMSO-d_6, 343 K)

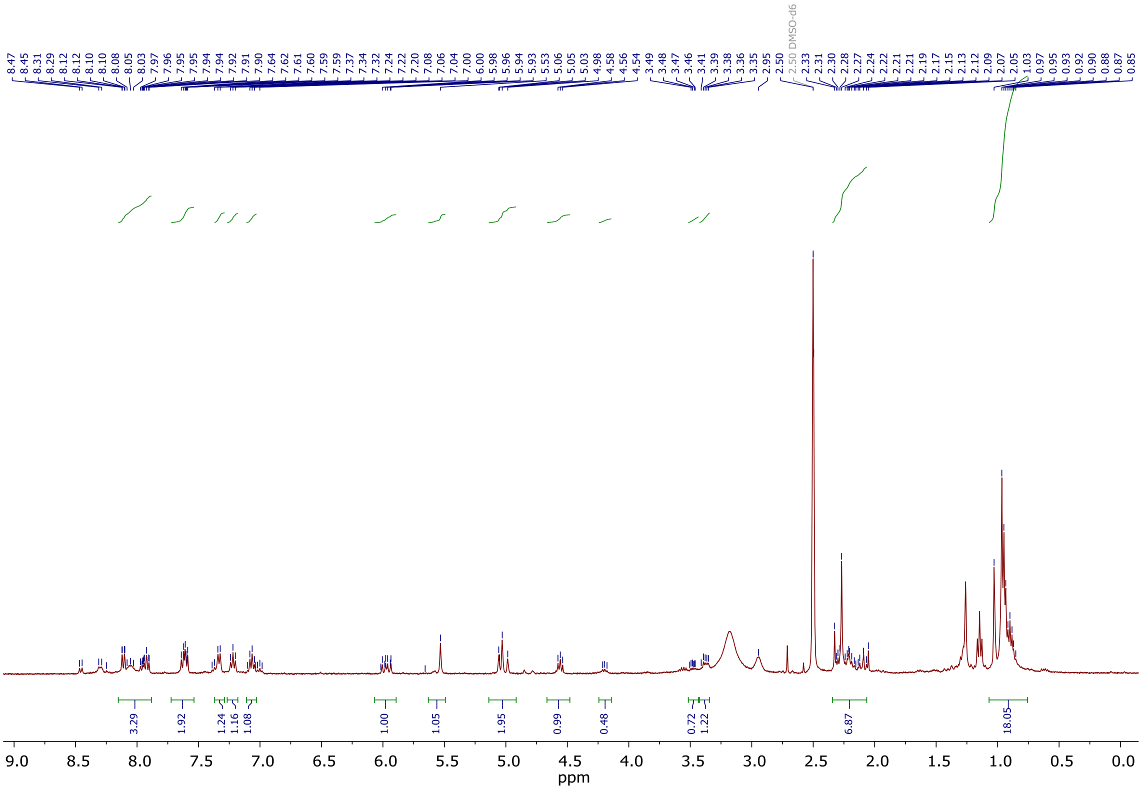

^{13}C{^{1}H} NMR (101 MHz, DMSO-d_6, 343 K)

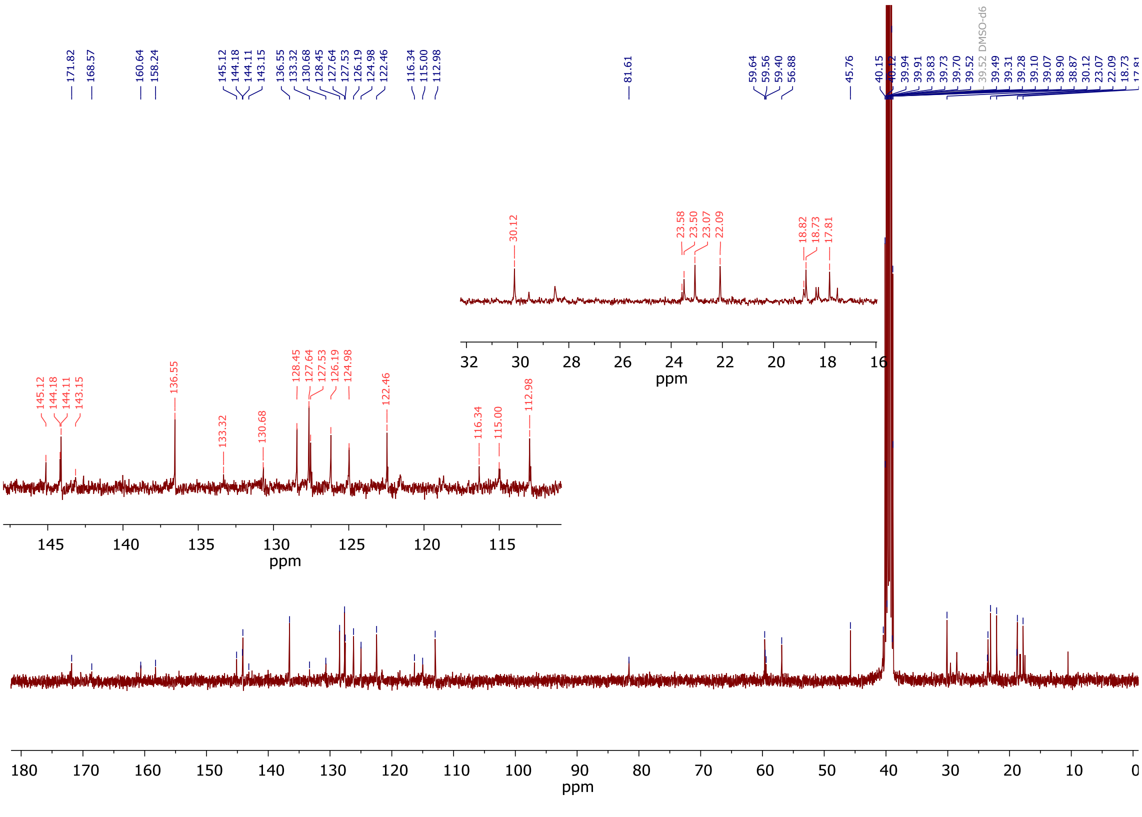

HSQC (101 MHz, DMSO-d_6, 343 K)

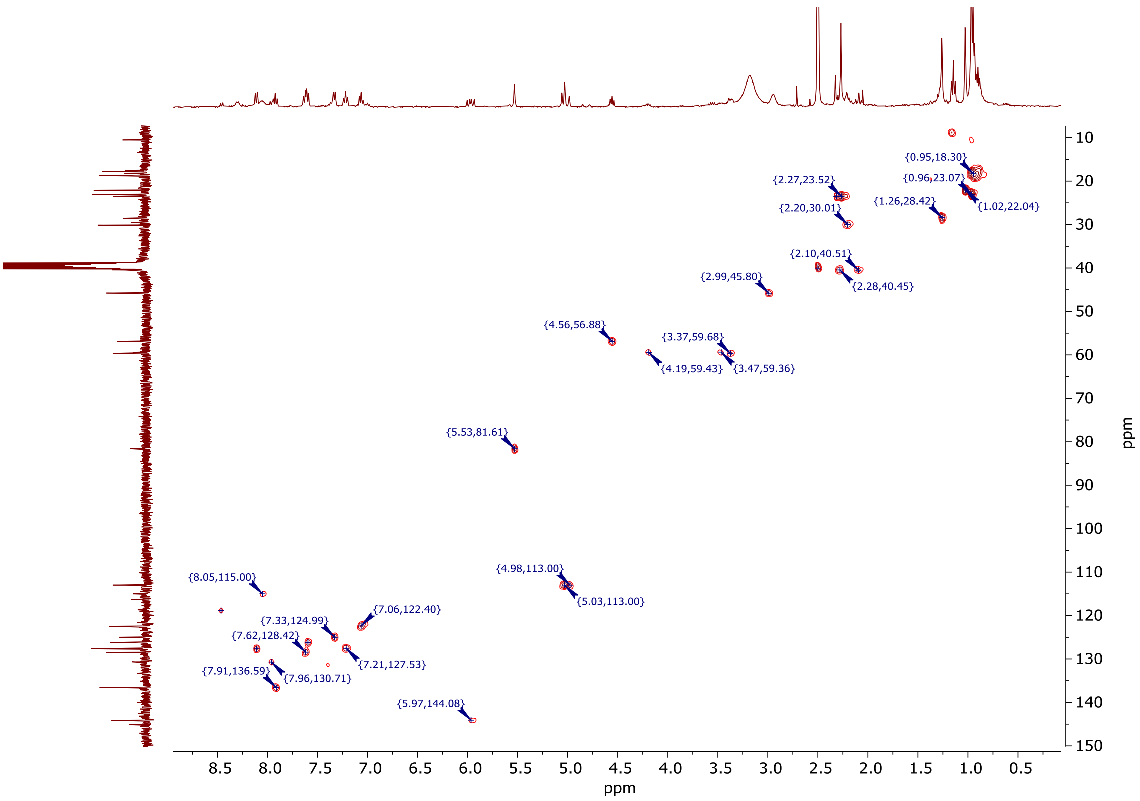

HMBC (101 MHz, DMSO-d_6, 343 K)

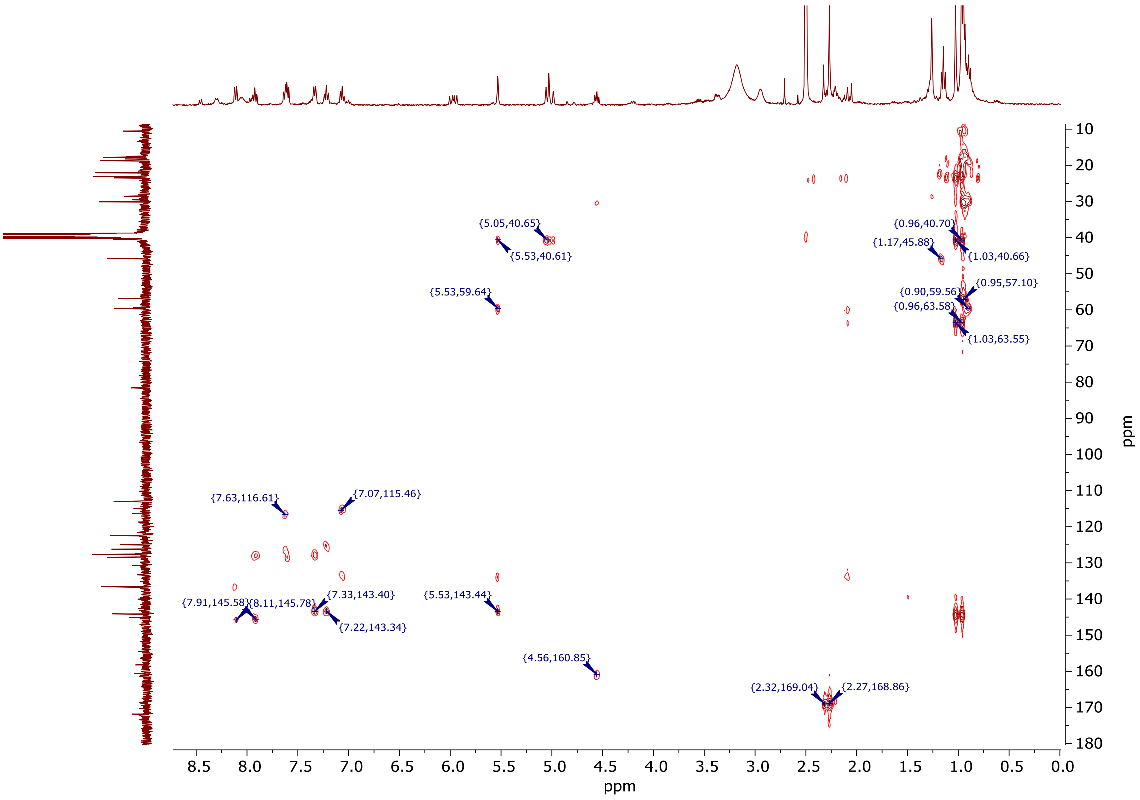

^{1}H NMR (400 MHz, $CDCl_3$, 333 K)

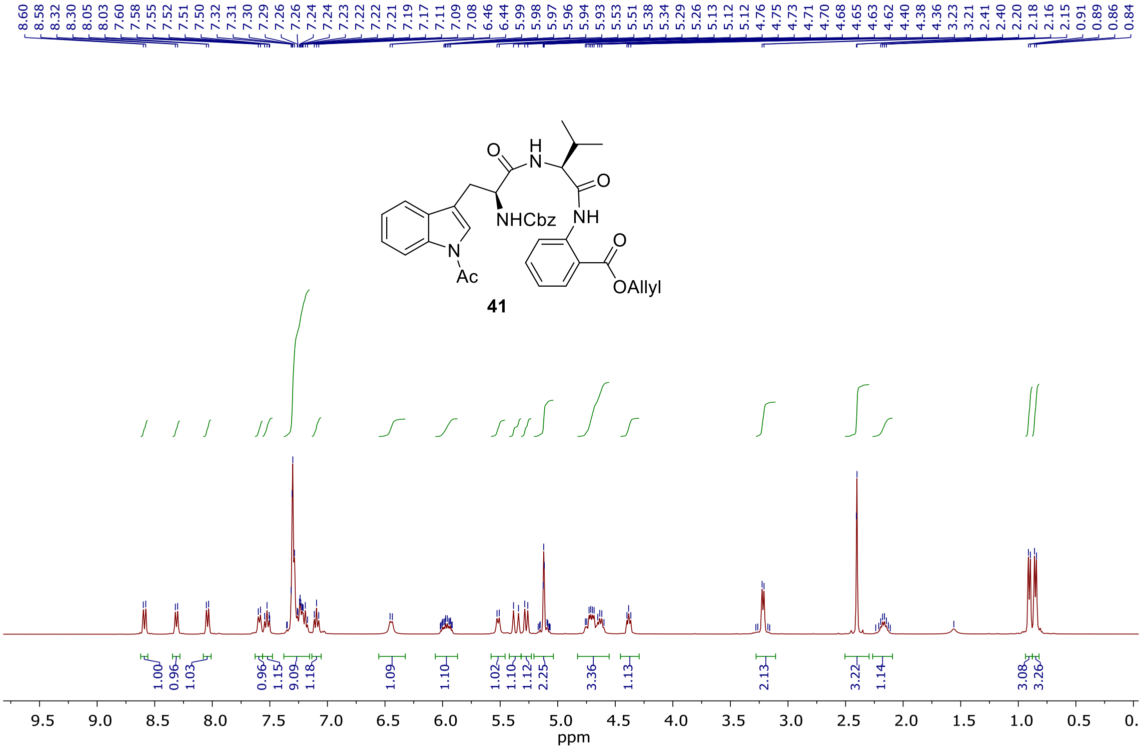

^{13}C{^{1}H} NMR (101 MHz, $CDCl_3$, 333 K)

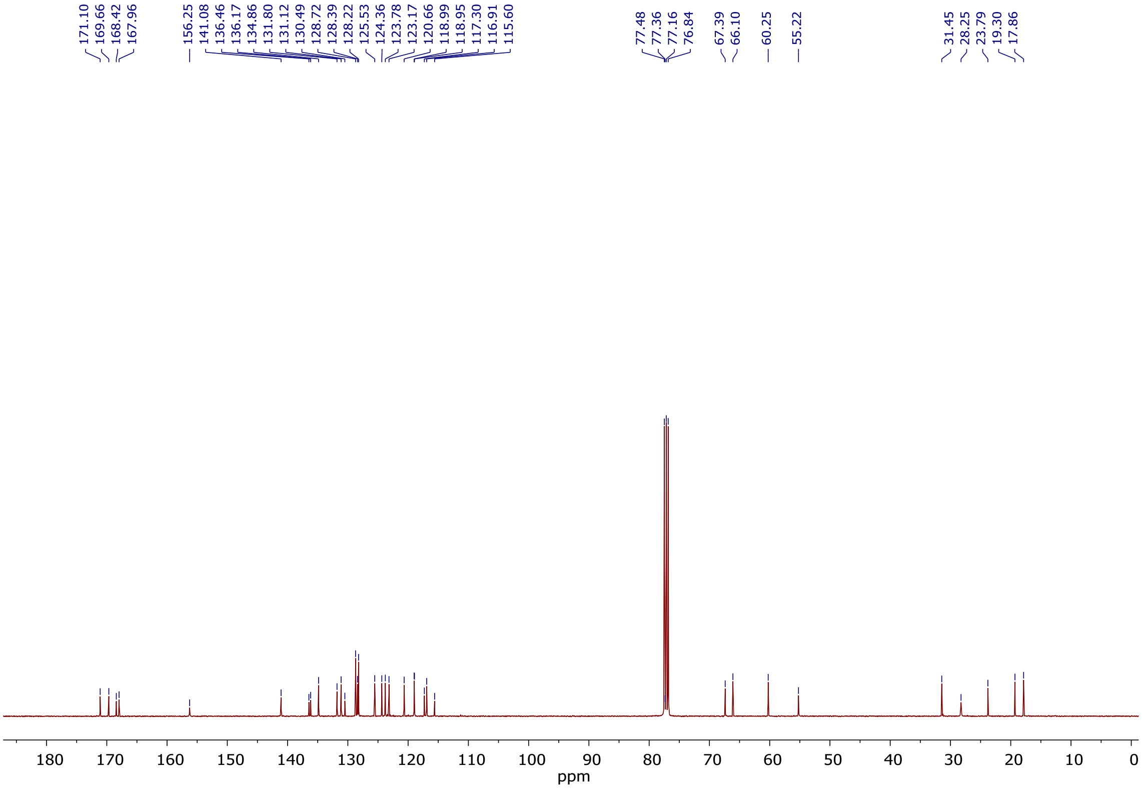

^{1}H NMR (400 MHz, DMSO-d_6, 343 K)

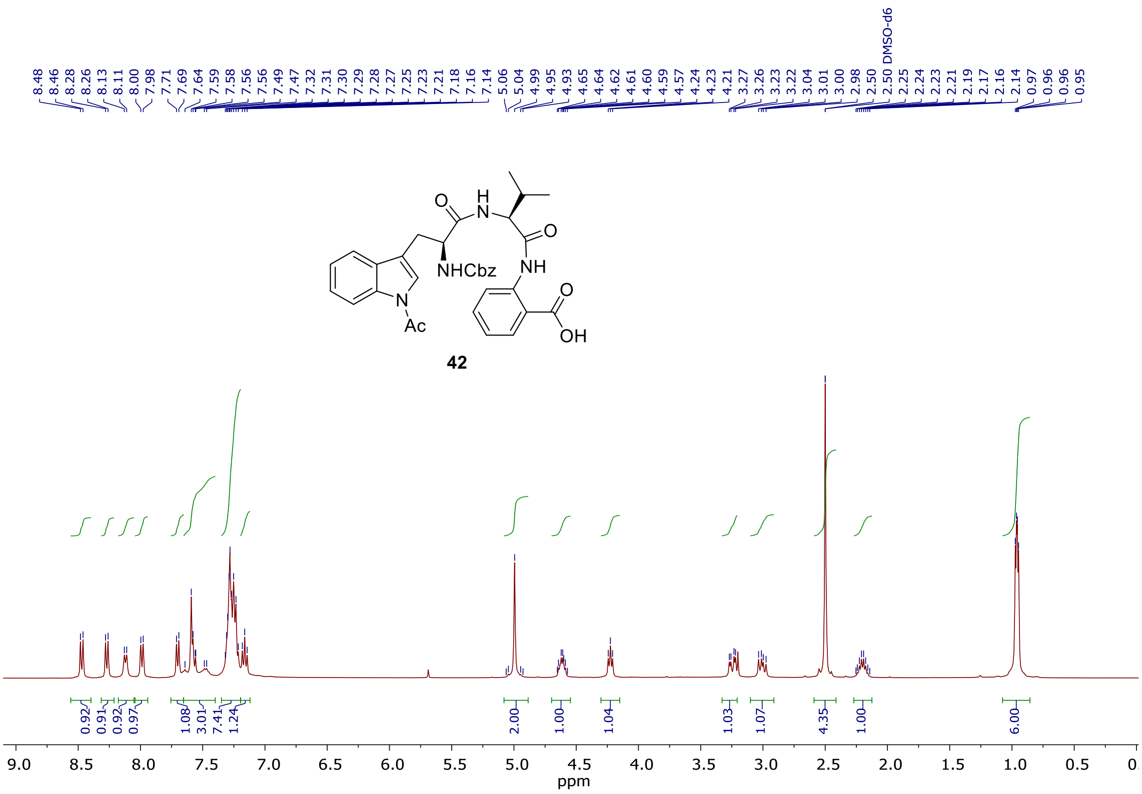

^{13}C{^{1}H} NMR (101 MHz, DMSO-d_6, 343 K)

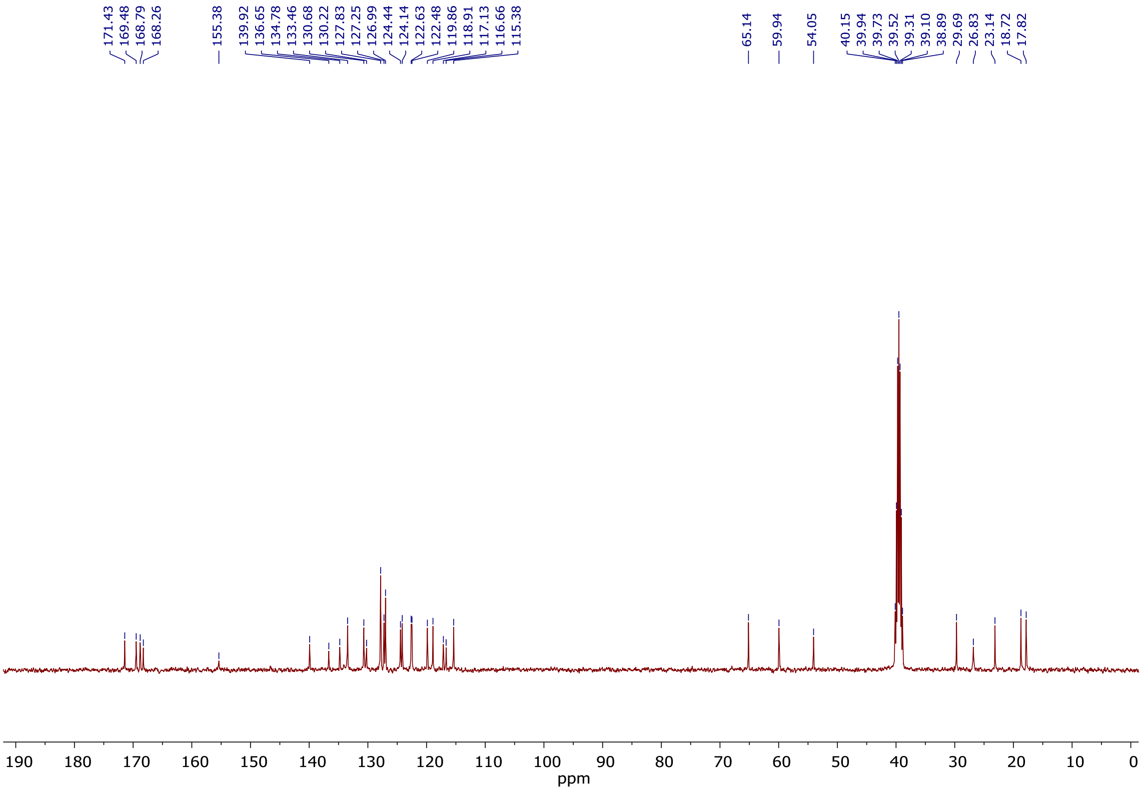

HPLC trace of the mixture of *exo*/*endo* diastereomers of **46** (Scharlau, C18 Kromaphase 100, 5 μm, 250 x 4.6 mm, gradient from CH_3CN/H_2O 50% to CH_3CN 100% in 20 min, 1.0 mL/min). No separation of the peaks.

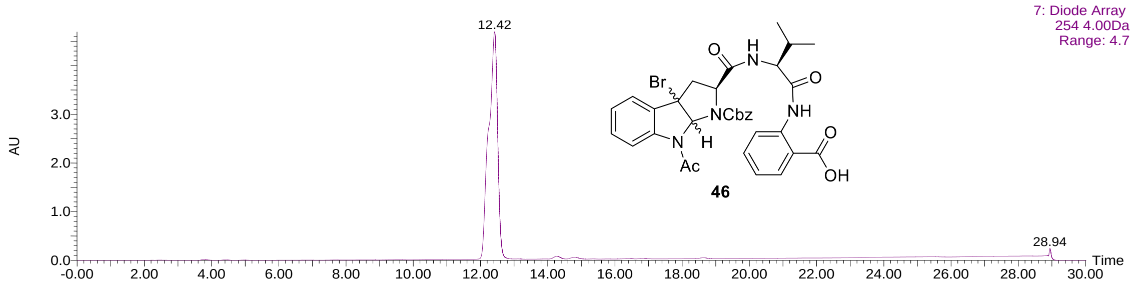

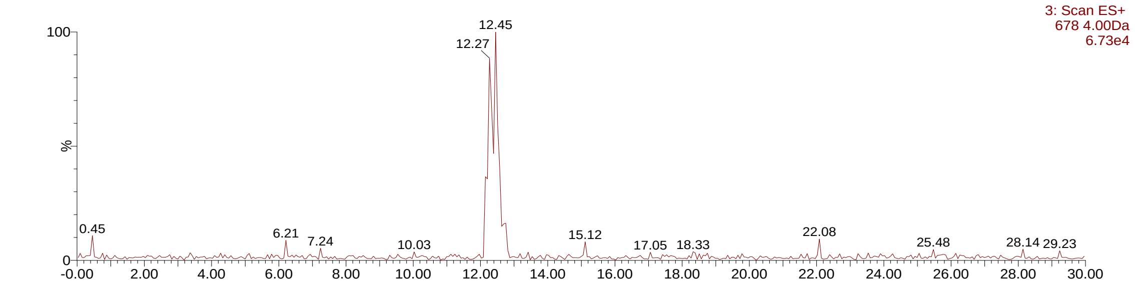

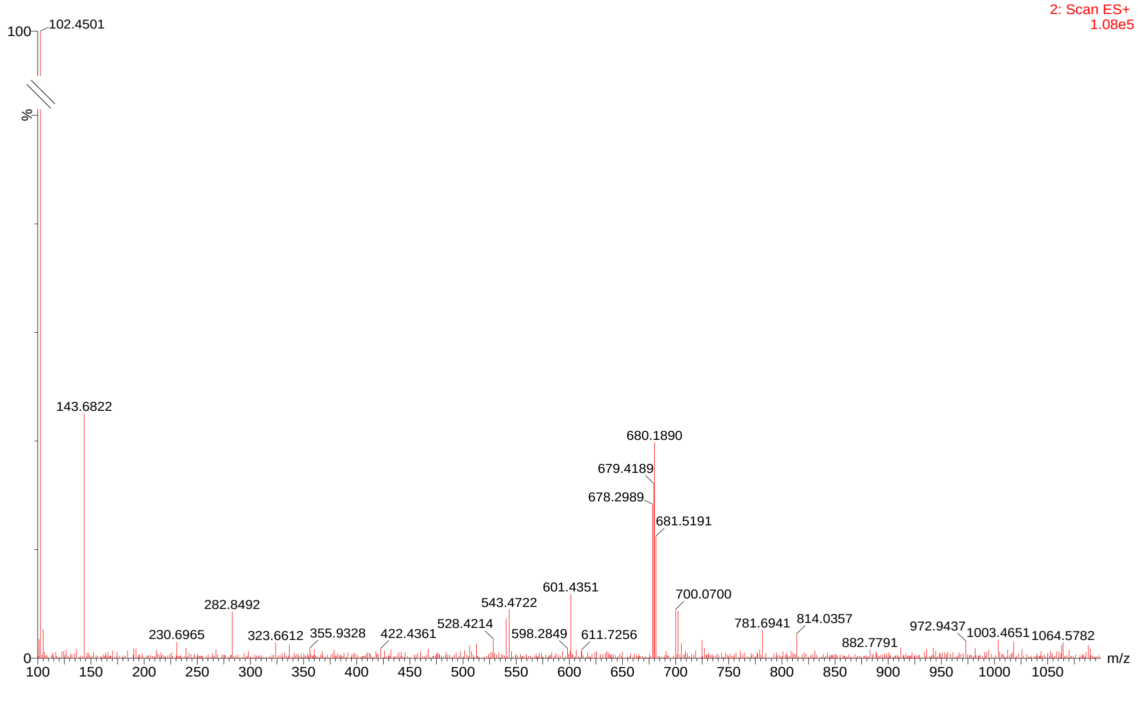

HPLC trace of the mixture of *exo*/*endo* diastereomers of **47** (Scharlau Kromaphase 100, C18, 5 µm, 250 x 4.6 mm, gradient from CH_3CN/H_2O 50% to CH_3CN 100% in 20 min, 1.0 mL/min). No separation of the peaks.

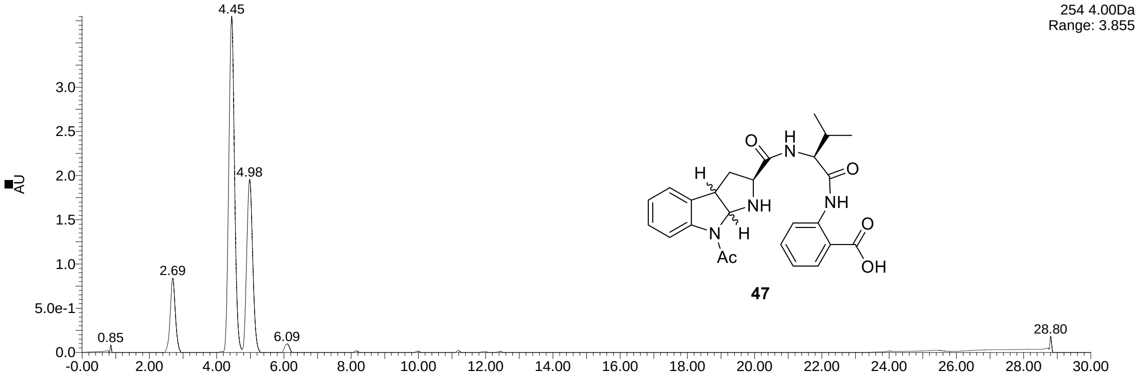

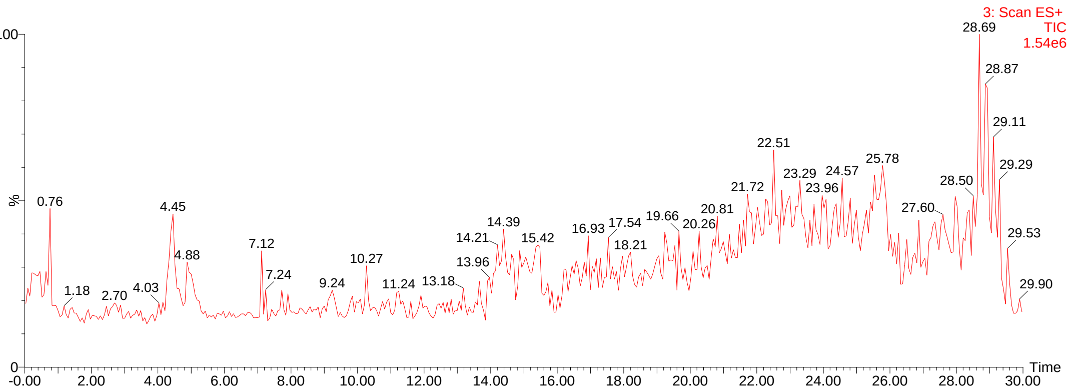

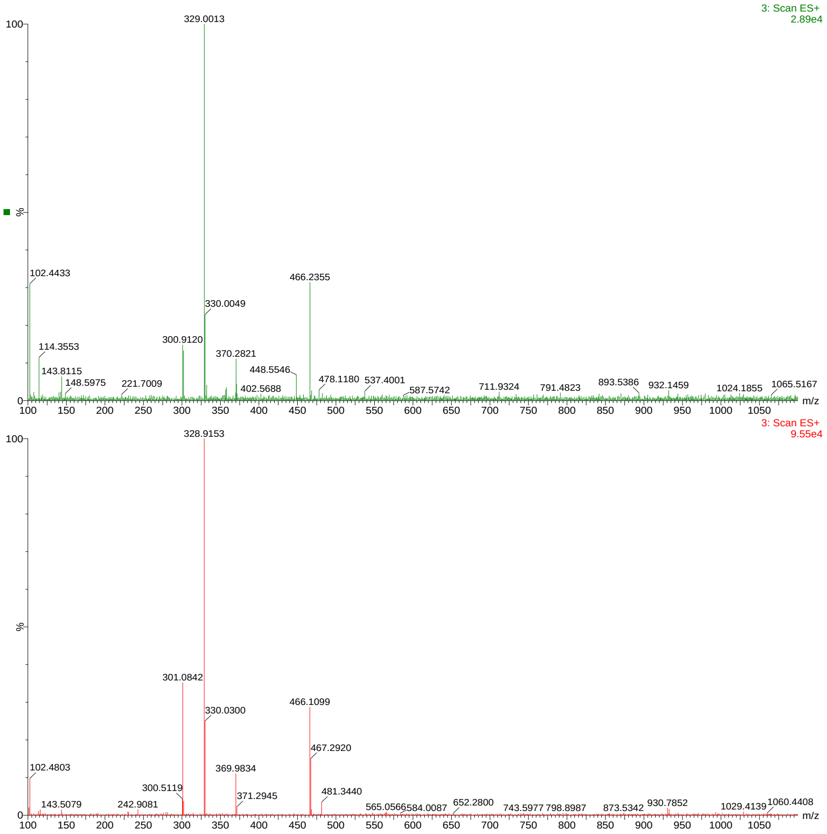

^{1}H NMR (400 MHz, DMSO-d_6, 298 K)

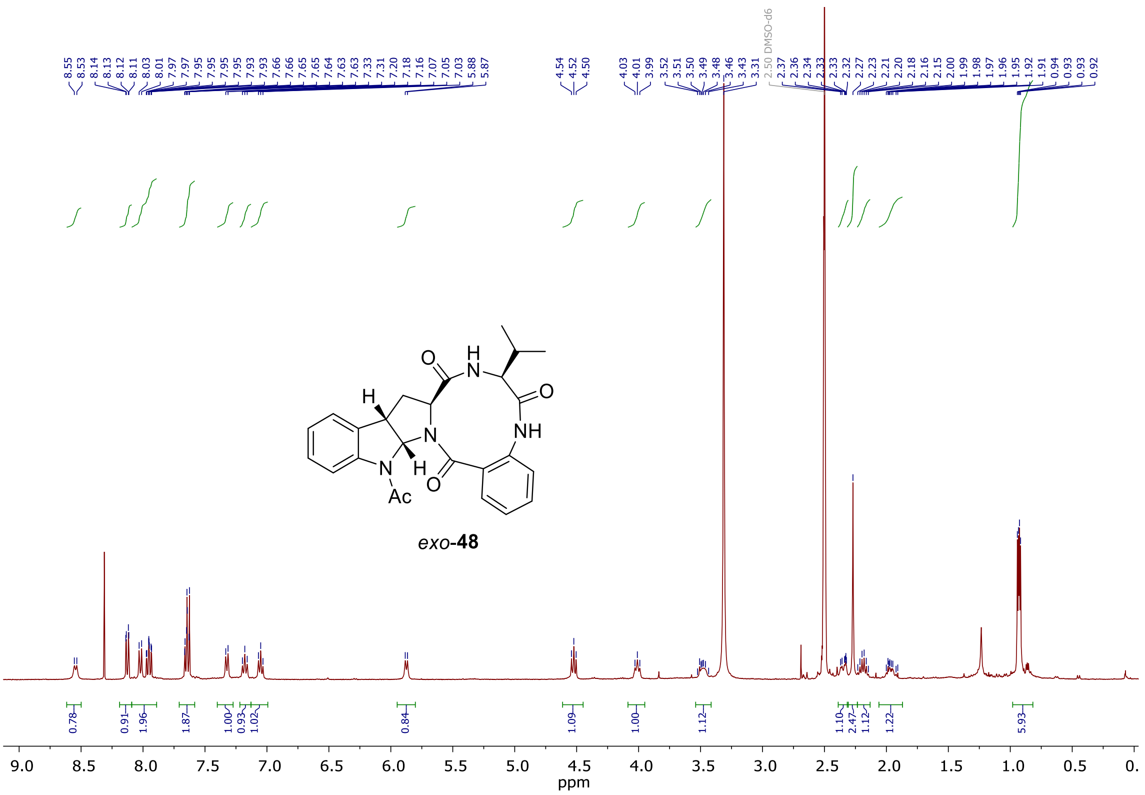

^{13}C{^{1}H} NMR (101 MHz, DMSO-d_6, 298 K)

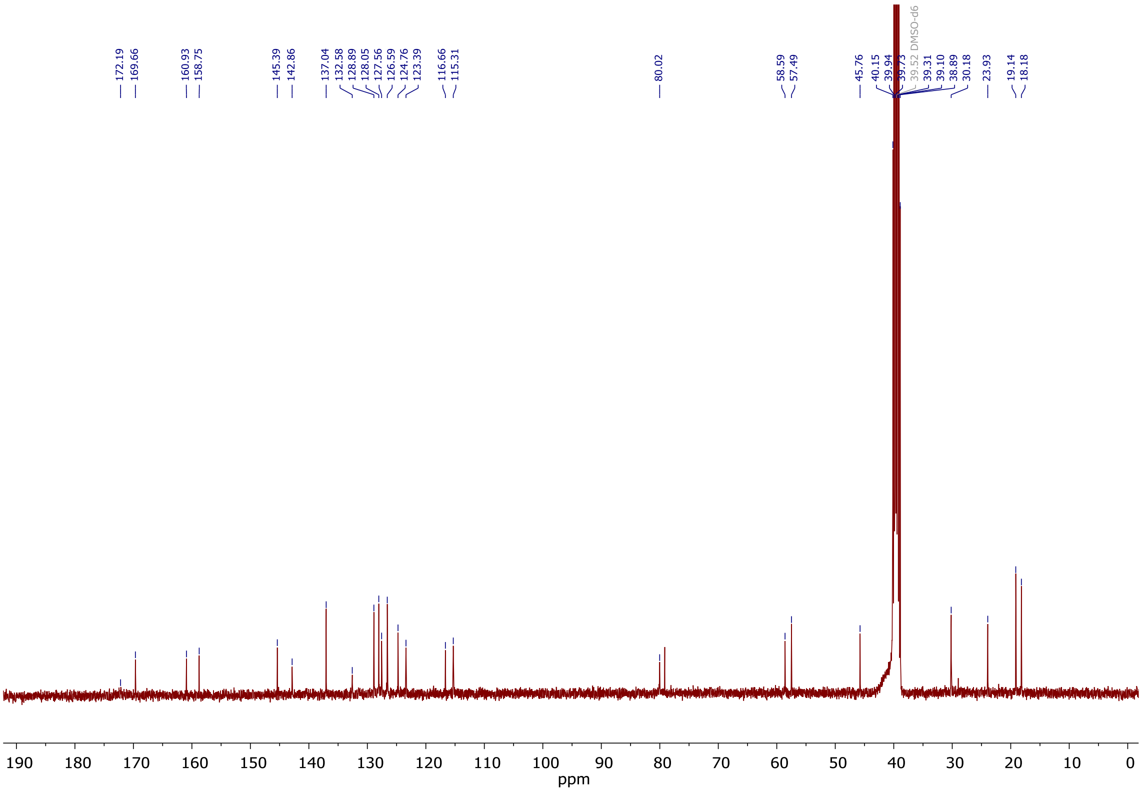

HSQC (101 MHz, DMSO-d_6, 298 K)

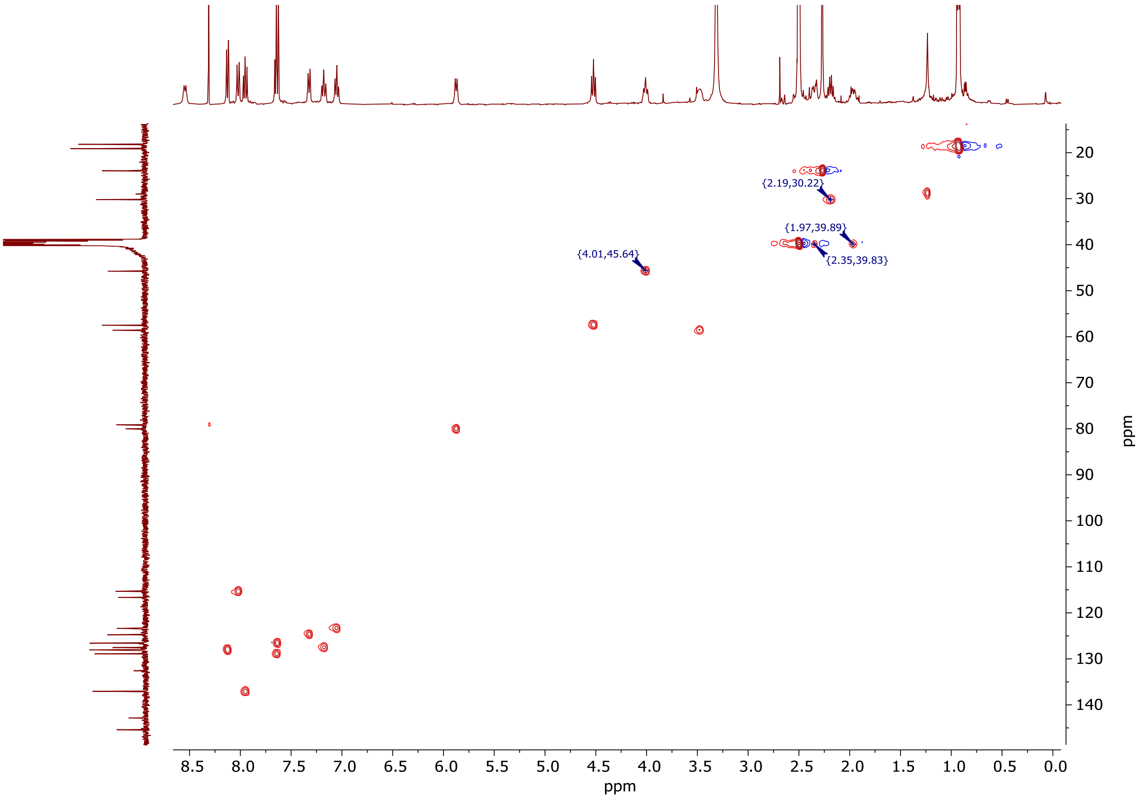

HMBC (101 MHz, DMSO-d_6, 298 K)

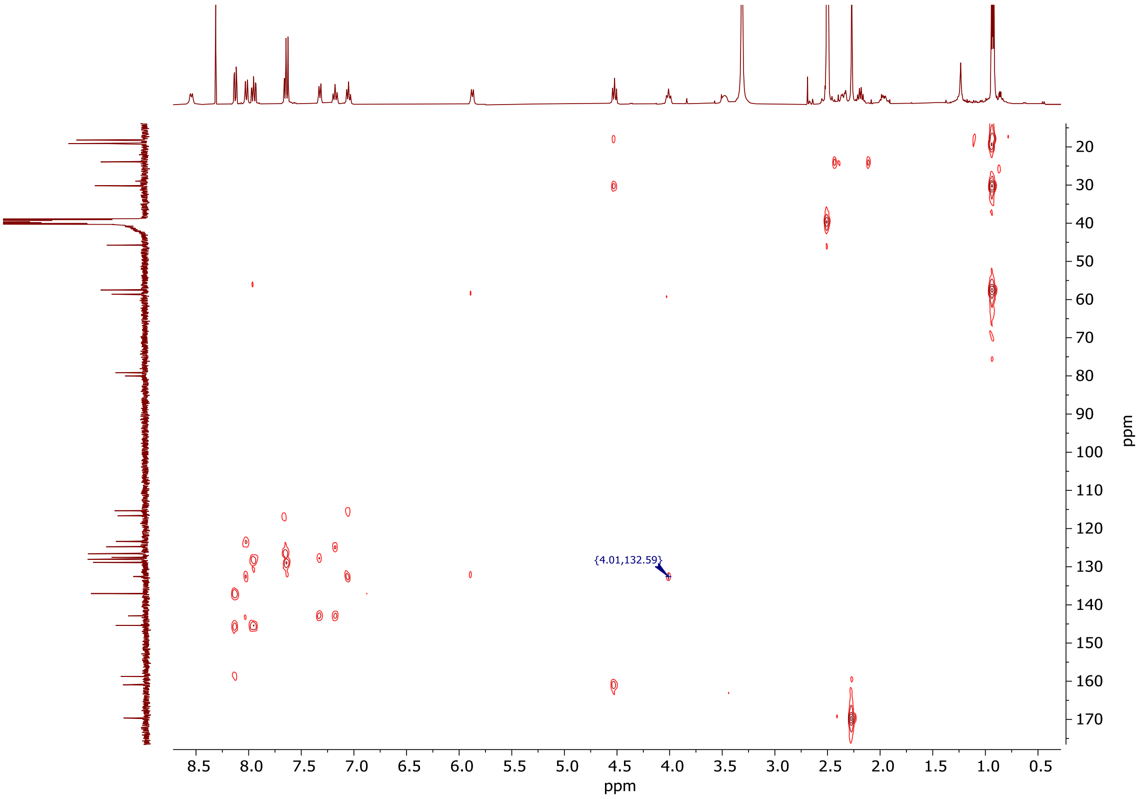

^{1}H NMR (400 MHz, DMSO-d_6, 343 K)

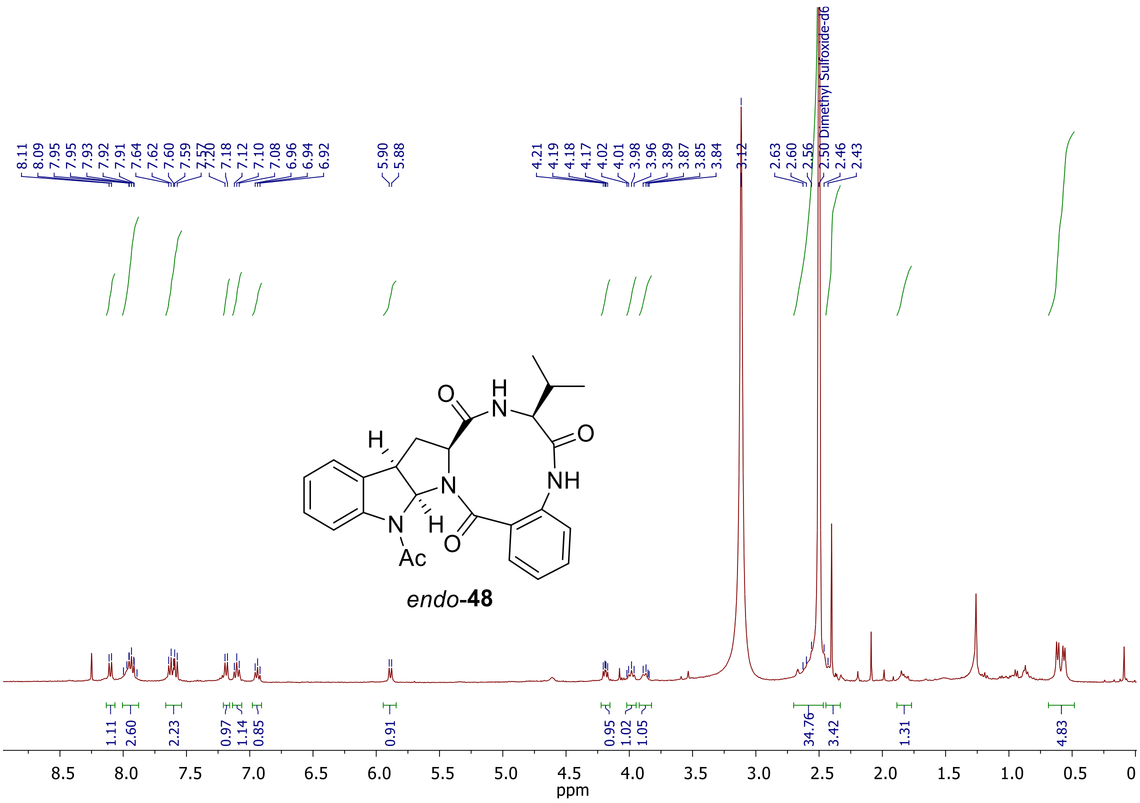

^{1}H NMR (400 MHz, CDCl$_3$)

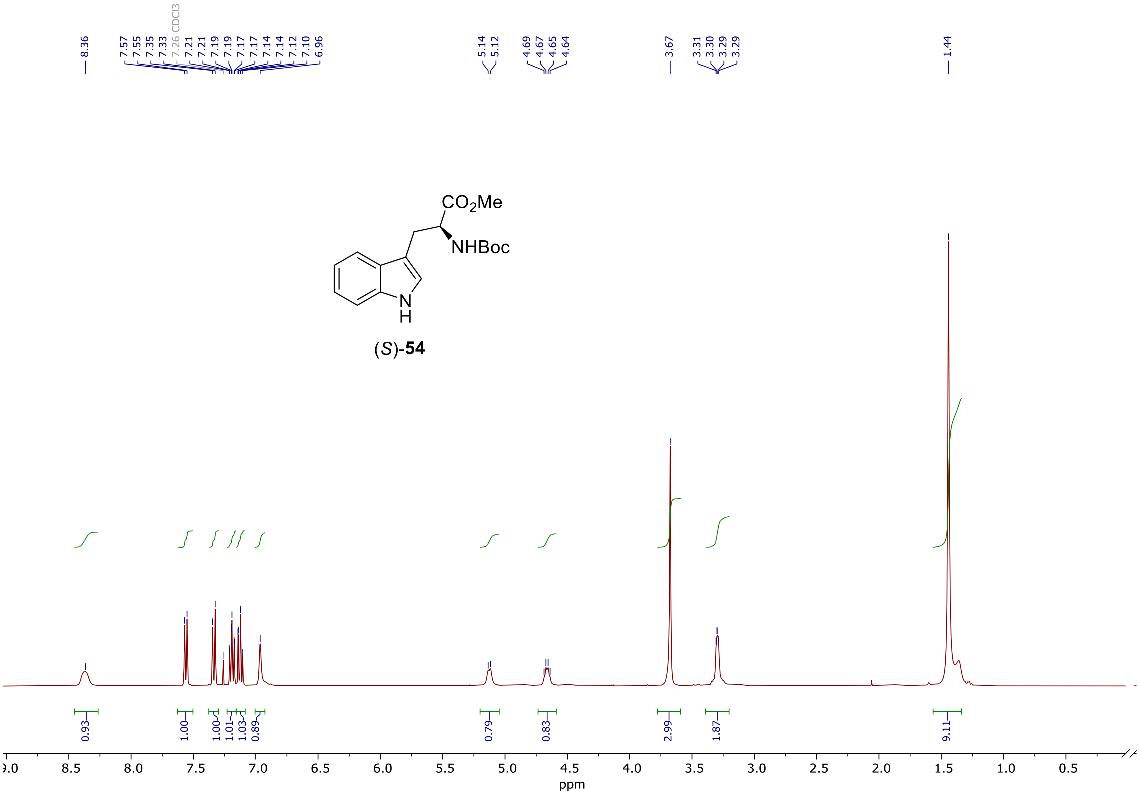

^{1}H NMR (400 MHz, CDCl$_3$, 323 K)

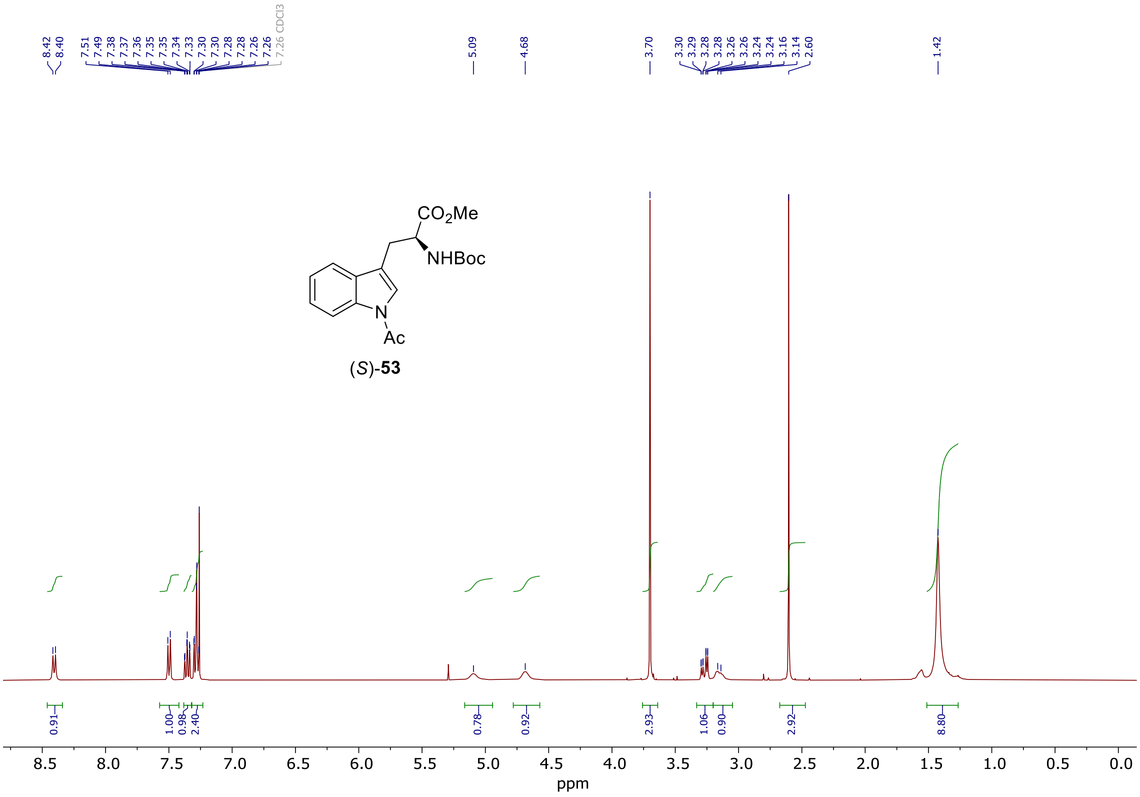

^{1}H NMR (400 MHz, DMSO-d_6, 343 K)

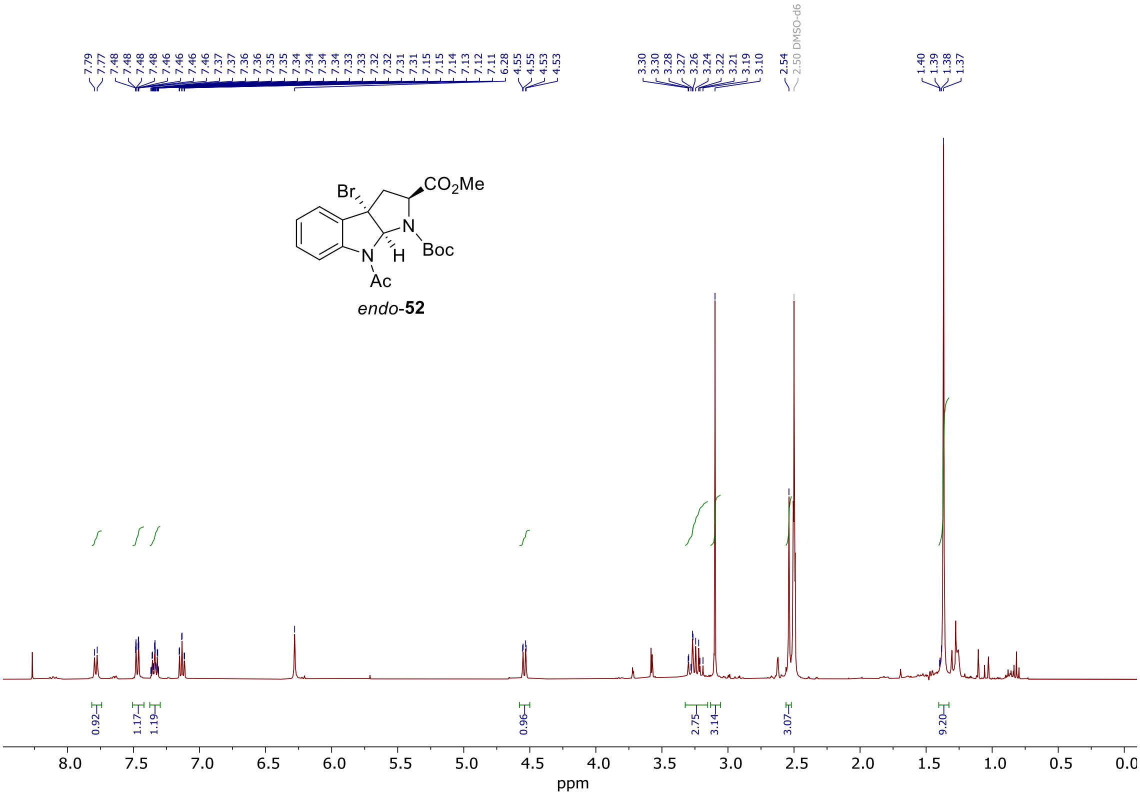

^{13}C{^{1}H} NMR (101 MHz, DMSO-d_6, 343 K)

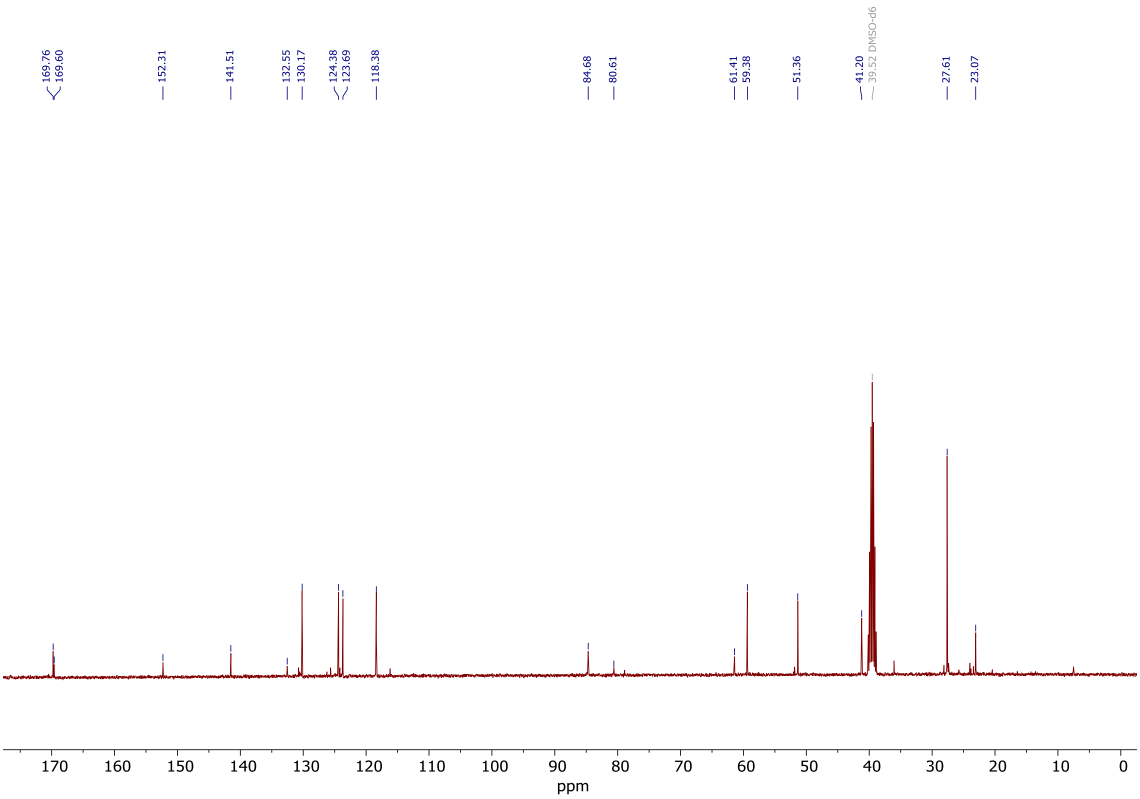

^{1}H NMR (400 MHz, DMSO-d_6, 343 K)

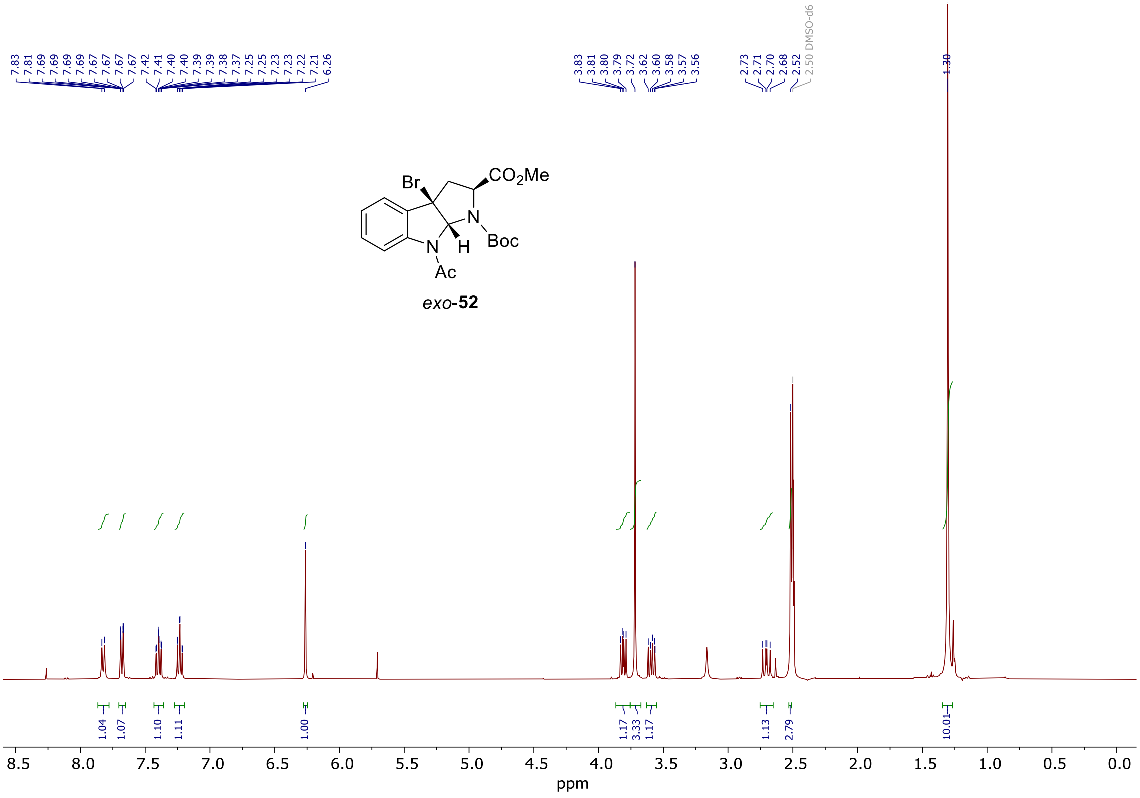

^{13}C{^{1}H} NMR (101 MHz, DMSO-d_6, 343 K)

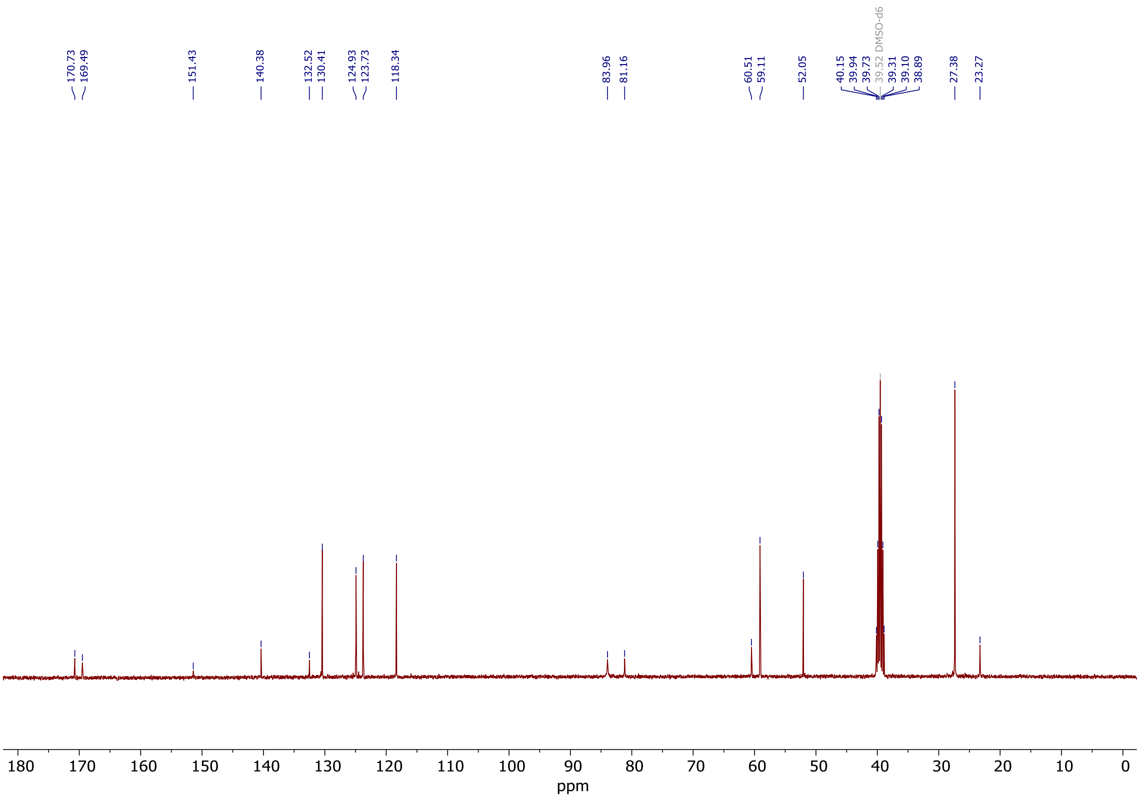

^{1}H NMR (400 MHz, DMSO-d_6, 308 K)

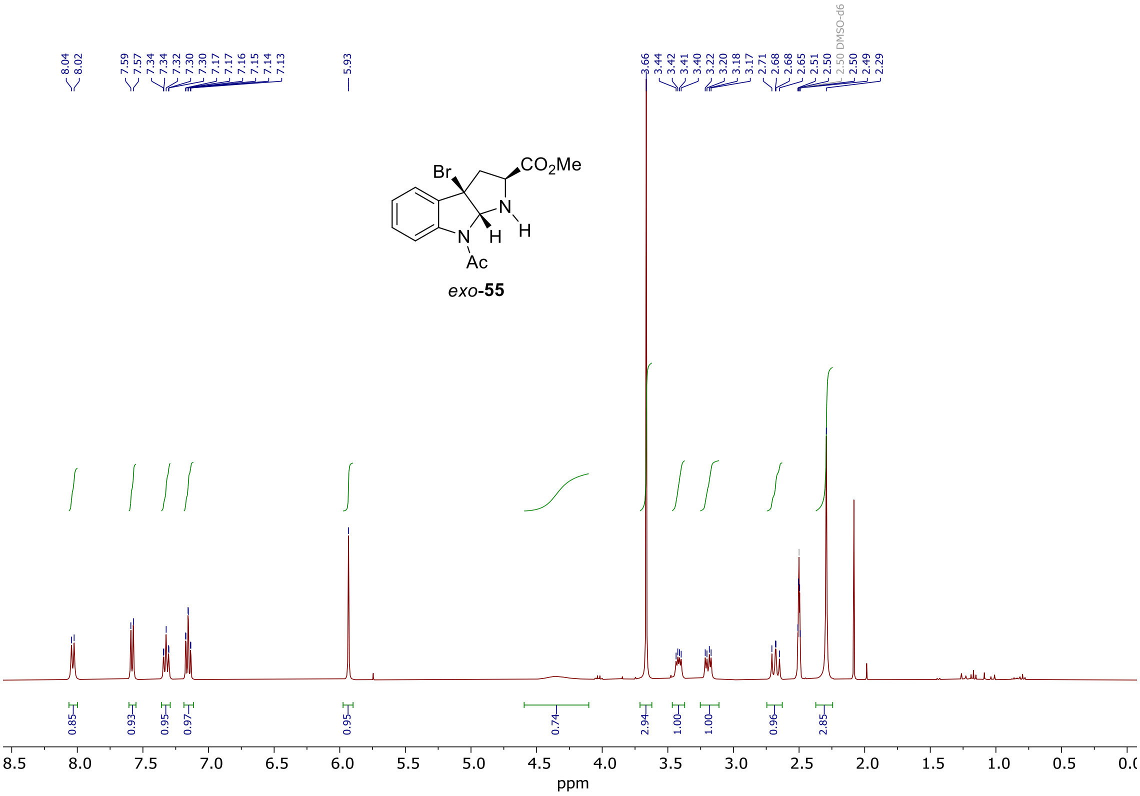

^{13}C{^{1}H} NMR (101 MHz, DMSO-d_6, 308 K)

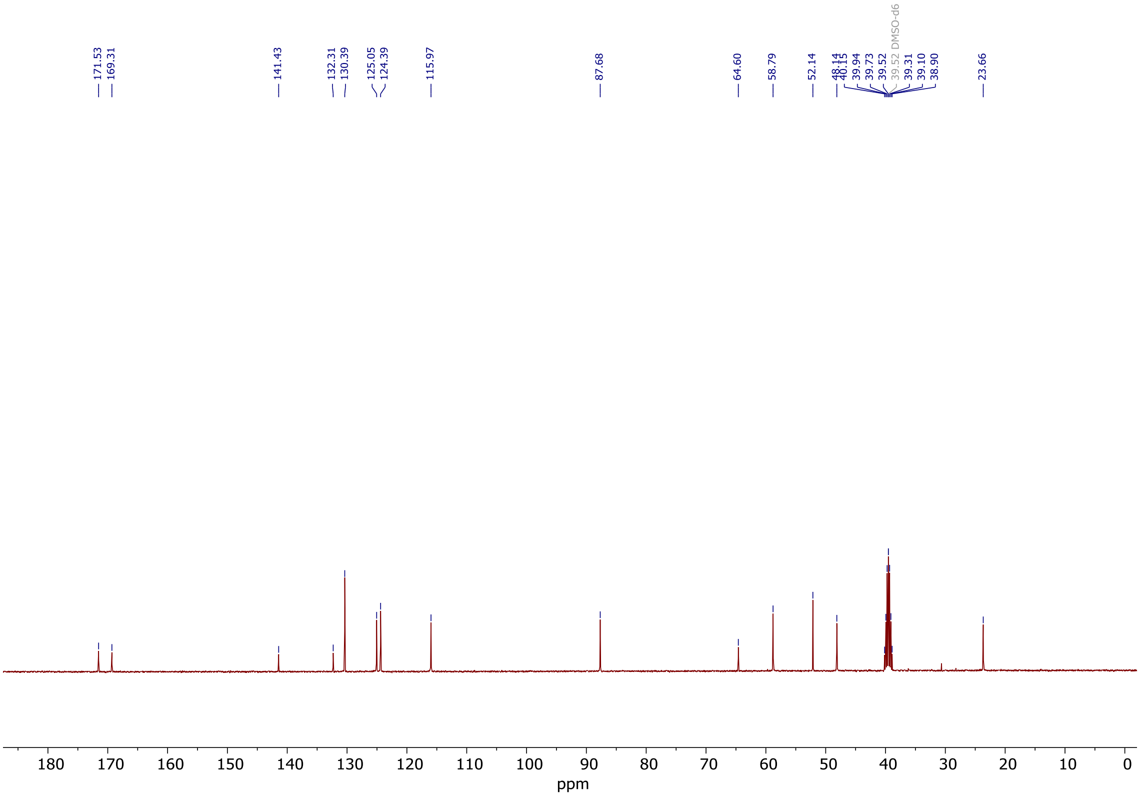

^{1}H NMR (400 MHz, DMSO-d_6, 298 K)

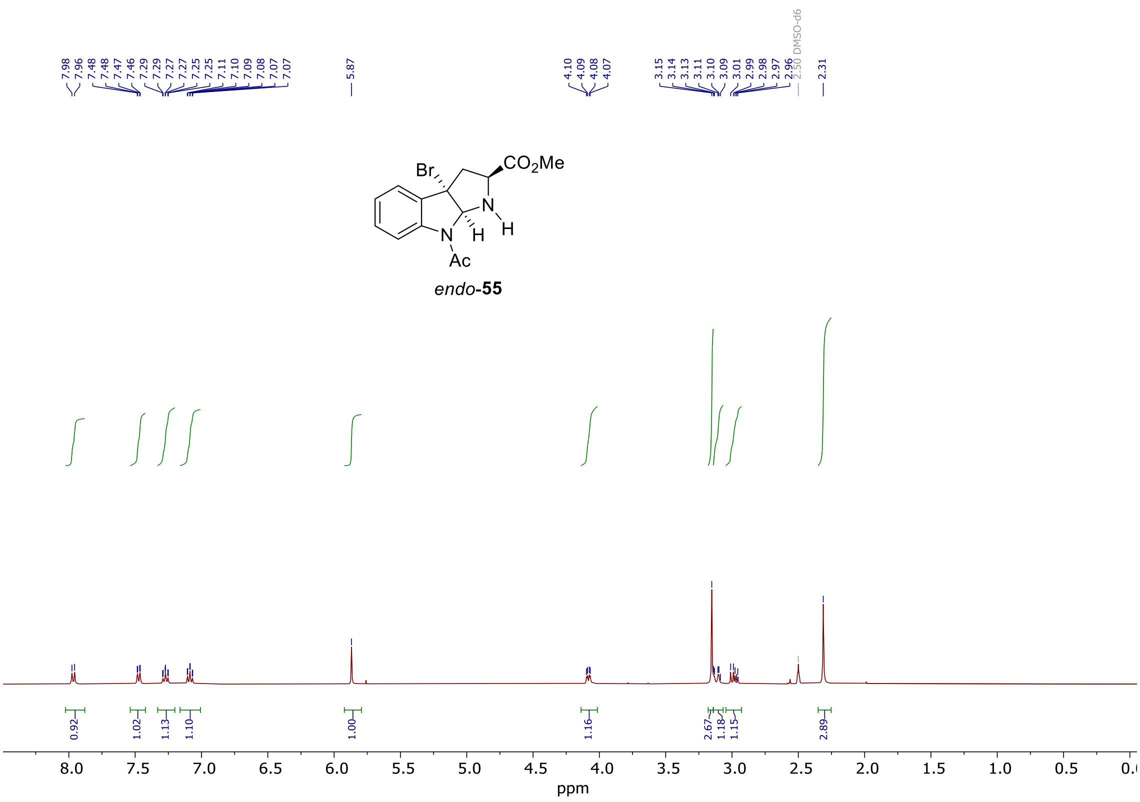

$^{13}C\{^{1}H\}$ NMR (101 MHz, DMSO-d_6, 298 K)

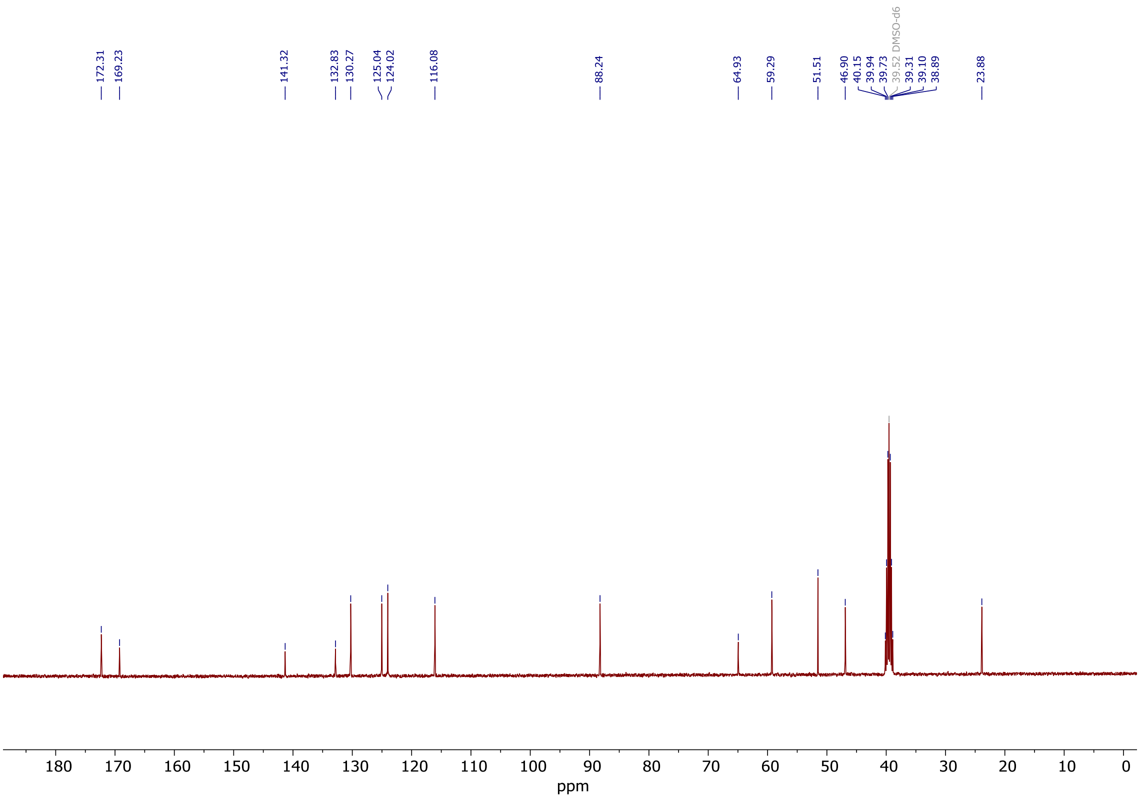

^{1}H NMR (400 MHz, DMSO-d_6, 343 K)

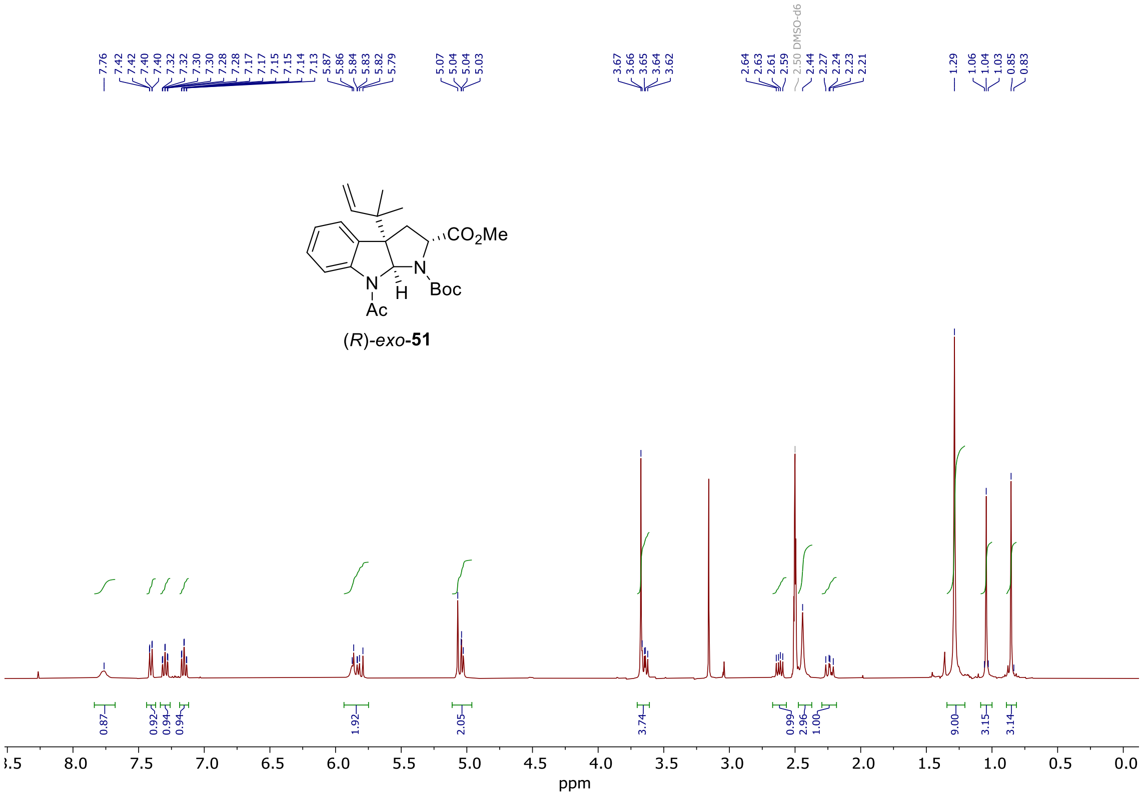

^{13}C{^{1}H} NMR (101 MHz, DMSO-d_6, 343 K)

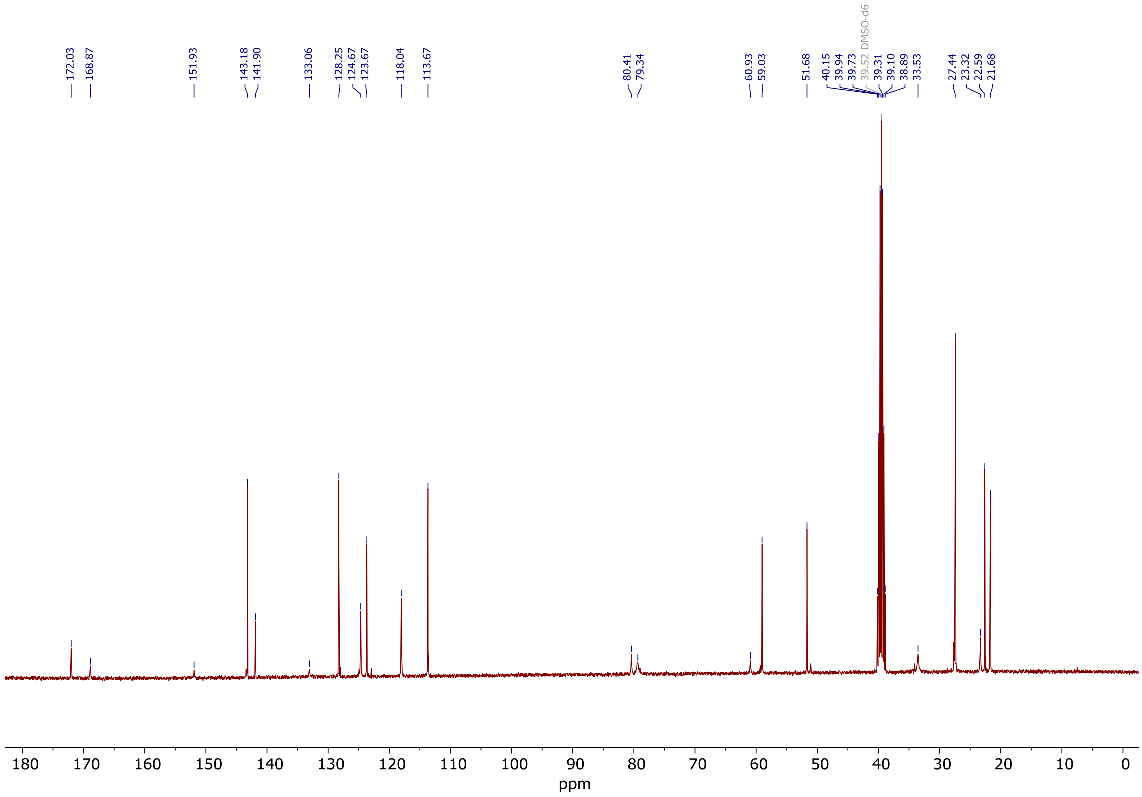

^{1}H NMR (400 MHz, $CDCl_3$)

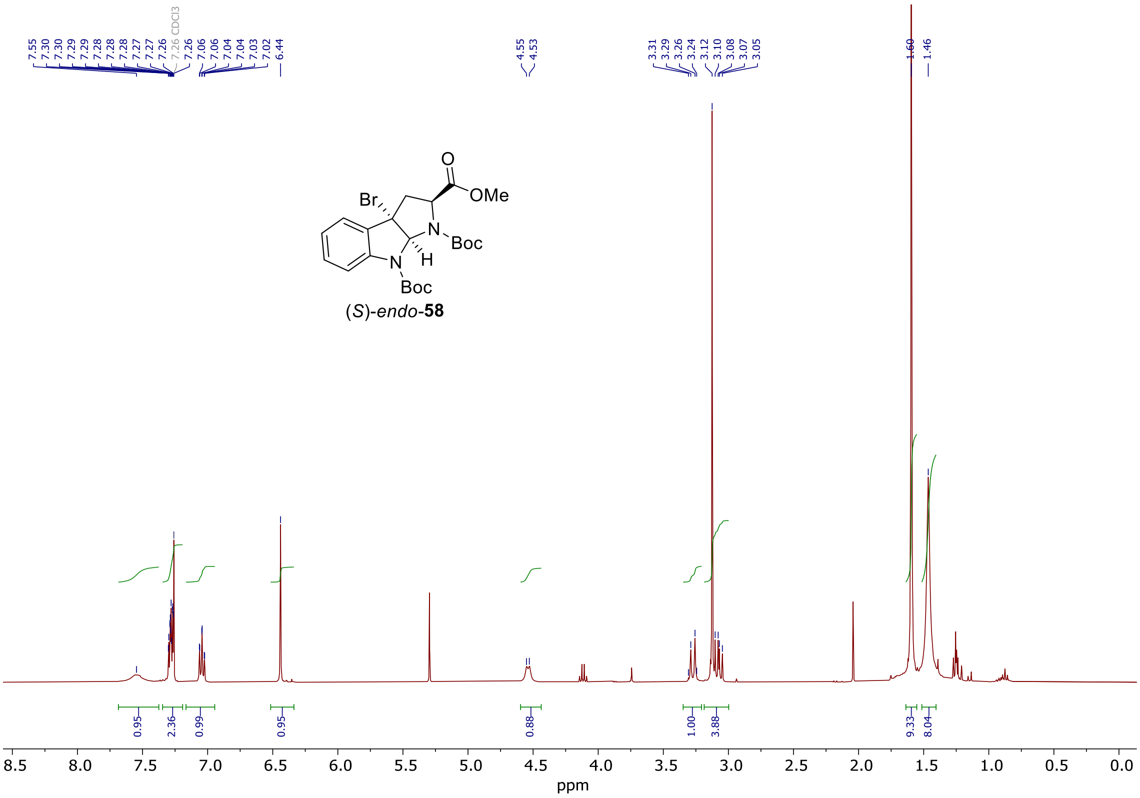

^{1}H NMR (400 MHz, $CDCl_3$)

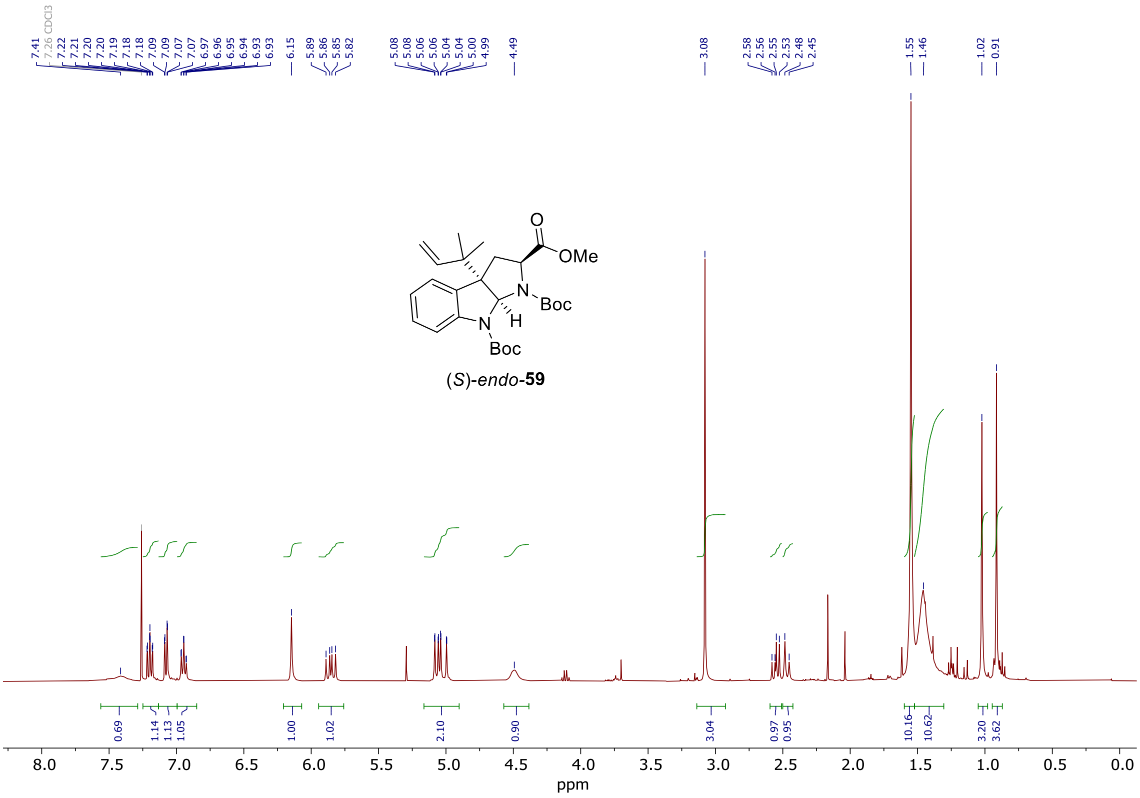

^{1}H NMR (400 MHz, CDCl$_3$)

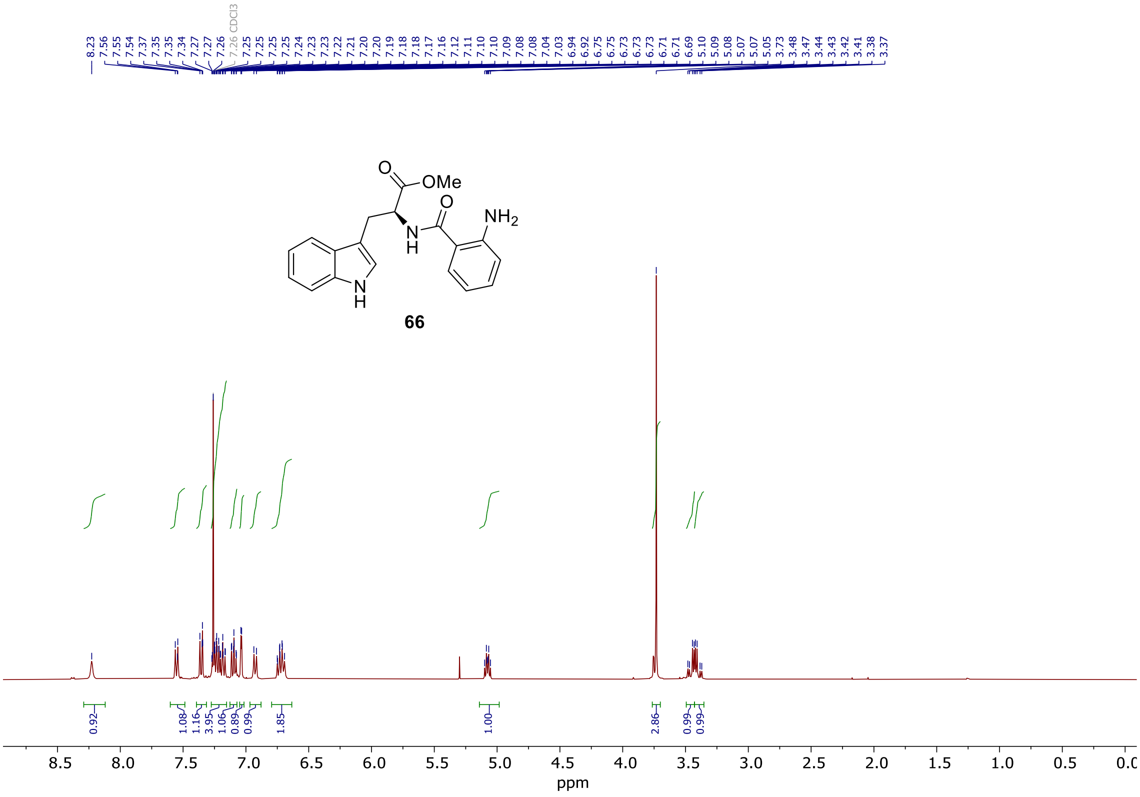

^{1}H NMR (400 MHz, DMSO-d_6, 343 K)

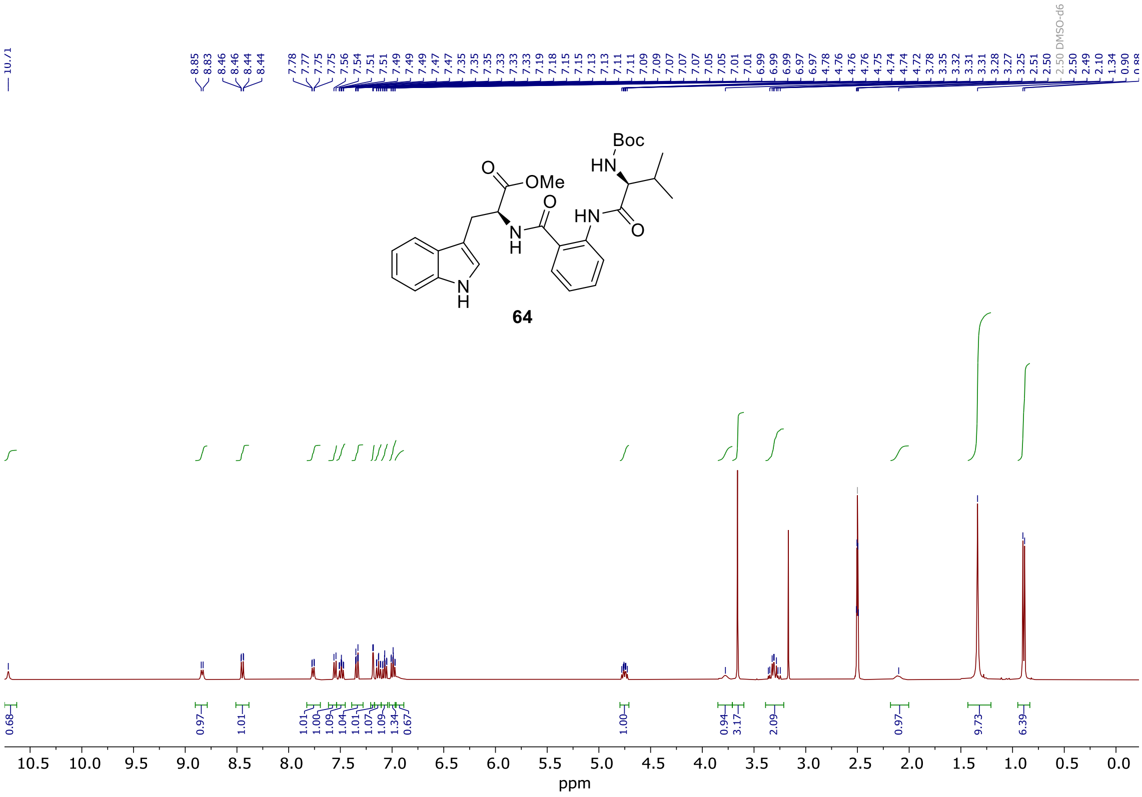

$^{13}C\{^{1}H\}$ NMR (101 MHz, DMSO-d_6, 343 K)

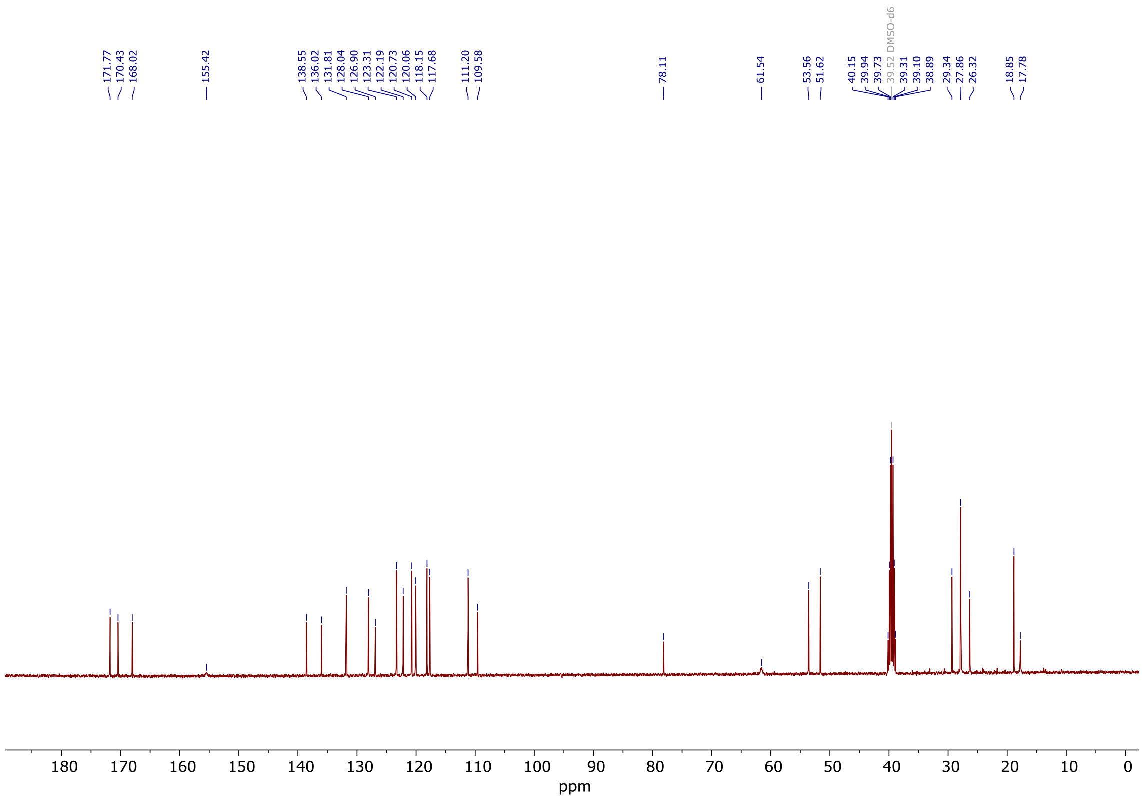

^{1}H NMR (400 MHz, DMSO-d_6, 343 K)

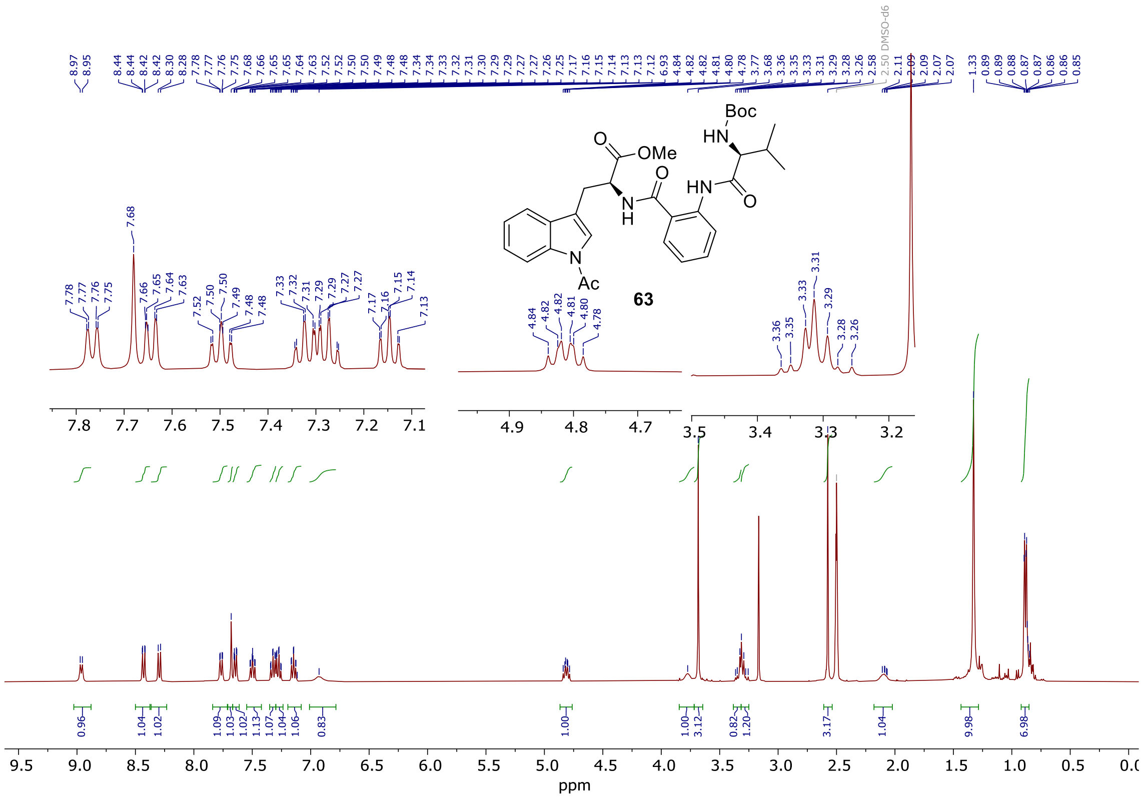

^{13}C{^{1}H} NMR (101 MHz, DMSO-d_6, 343 K)

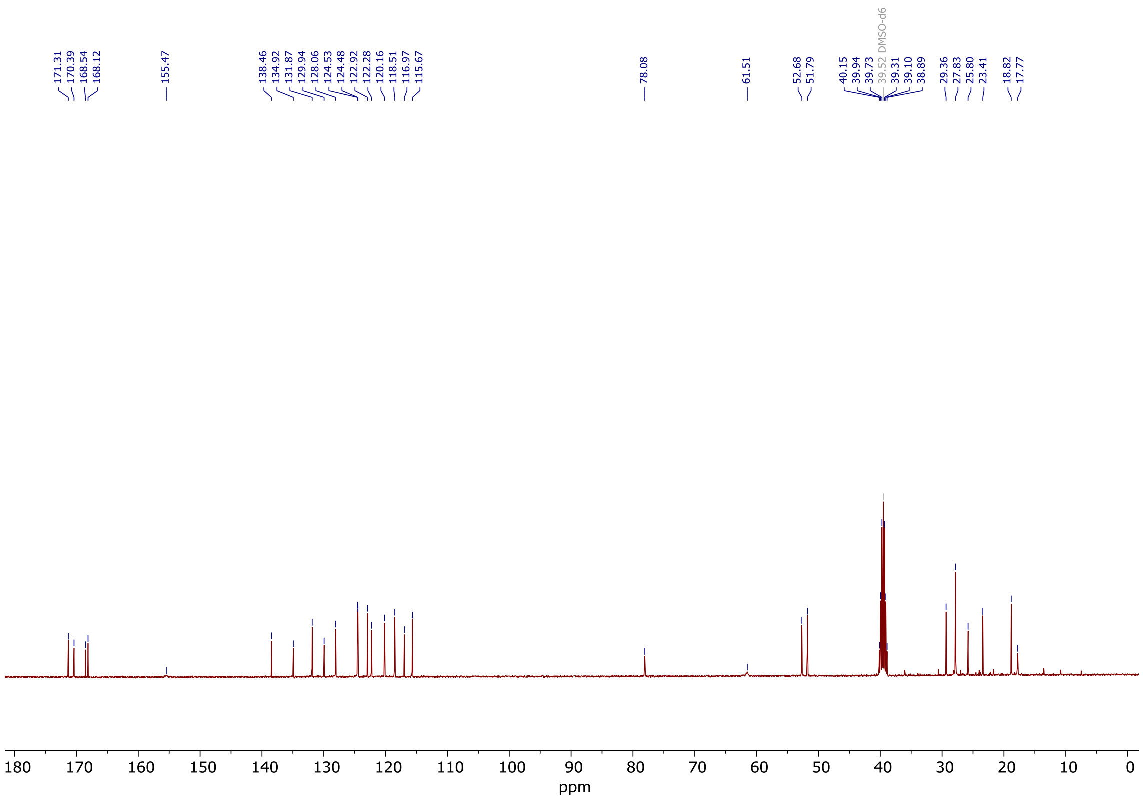

^{1}H NMR (400 MHz, DMSO-d_6, 343 K)

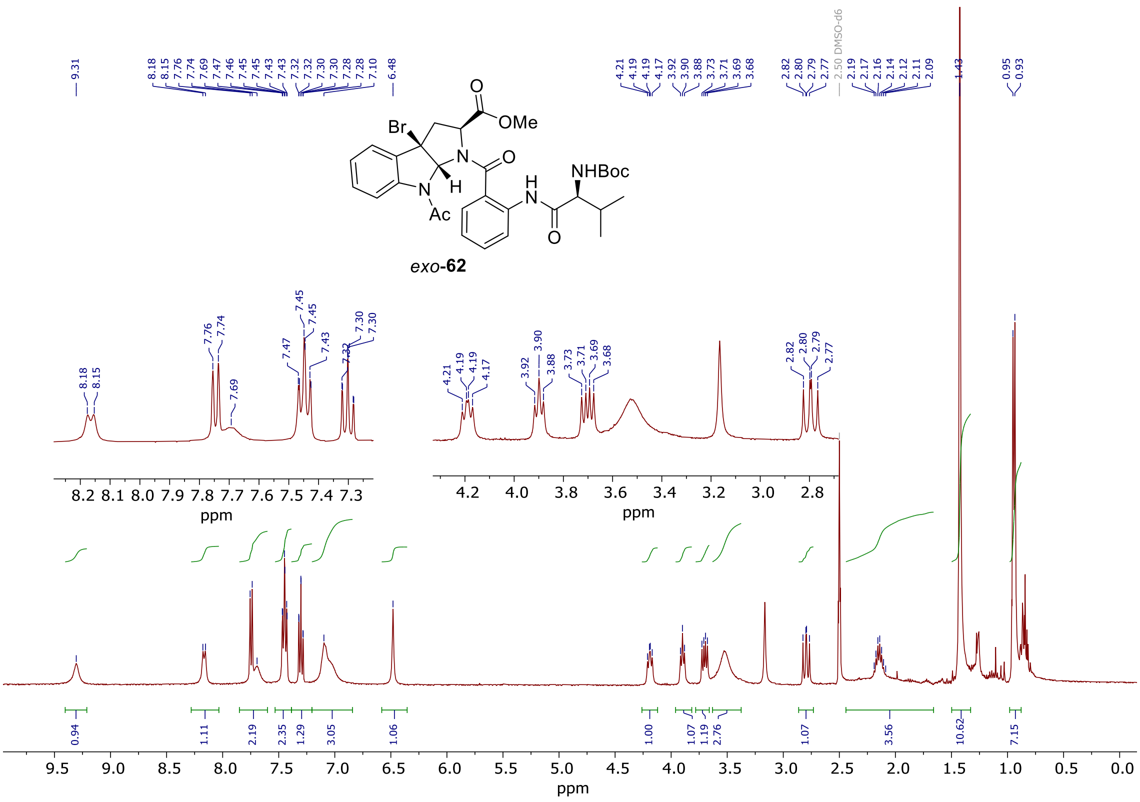

^{13}C{^{1}H} NMR (101 MHz, DMSO-d_6, 343 K)

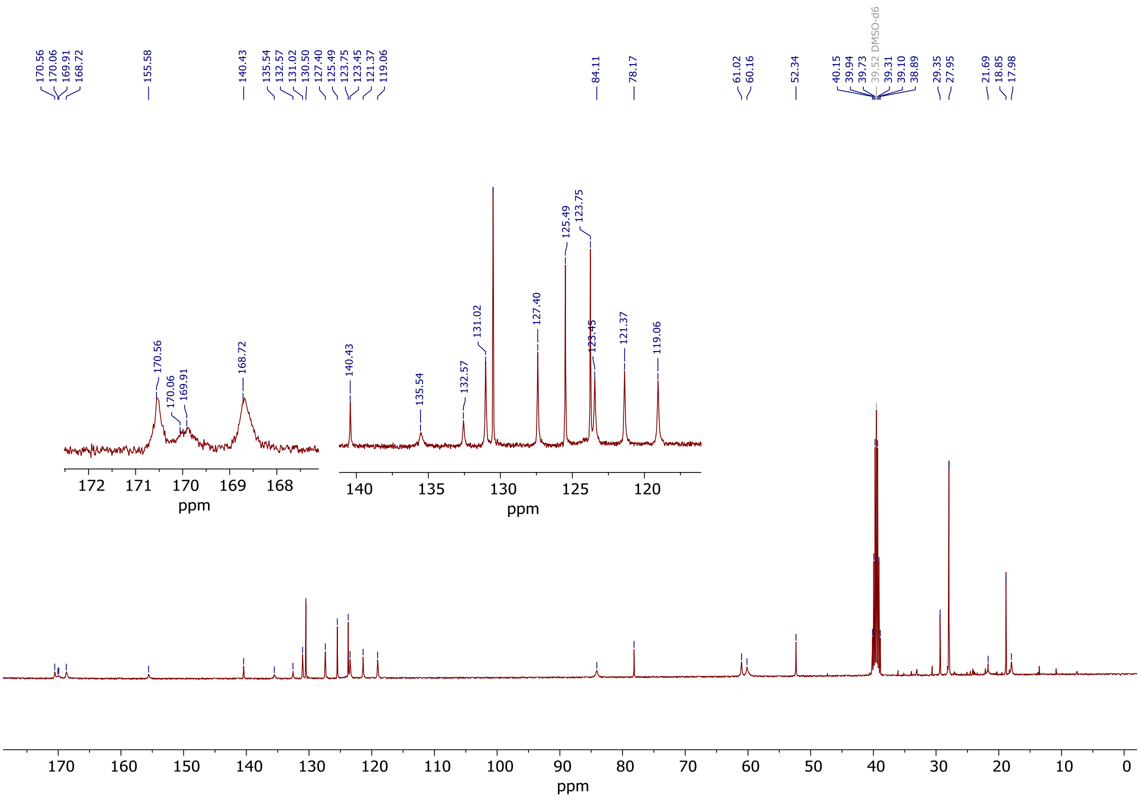

^{1}H NMR (400 MHz, DMSO-d_6, 343 K)

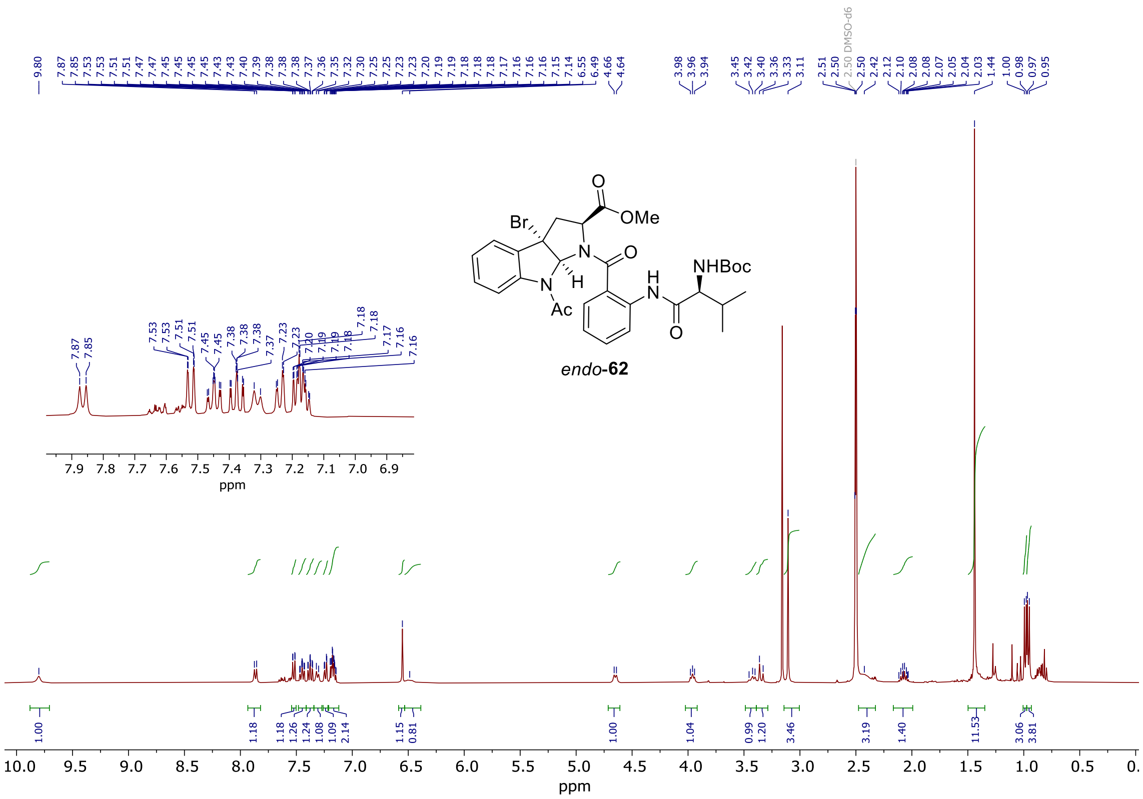

^{13}C{^{1}H} NMR (101 MHz, DMSO-d_6, 343 K)

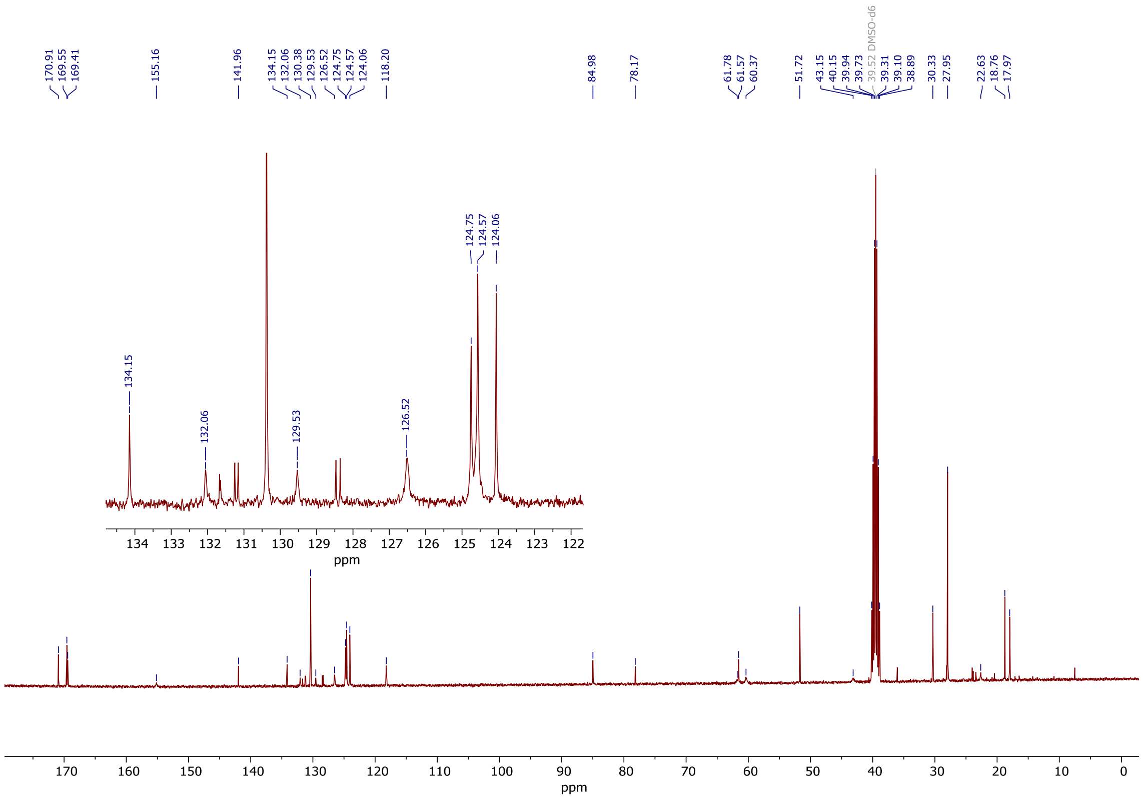

^{1}H NMR (400 MHz, DMSO-*d_6*, 343 K)

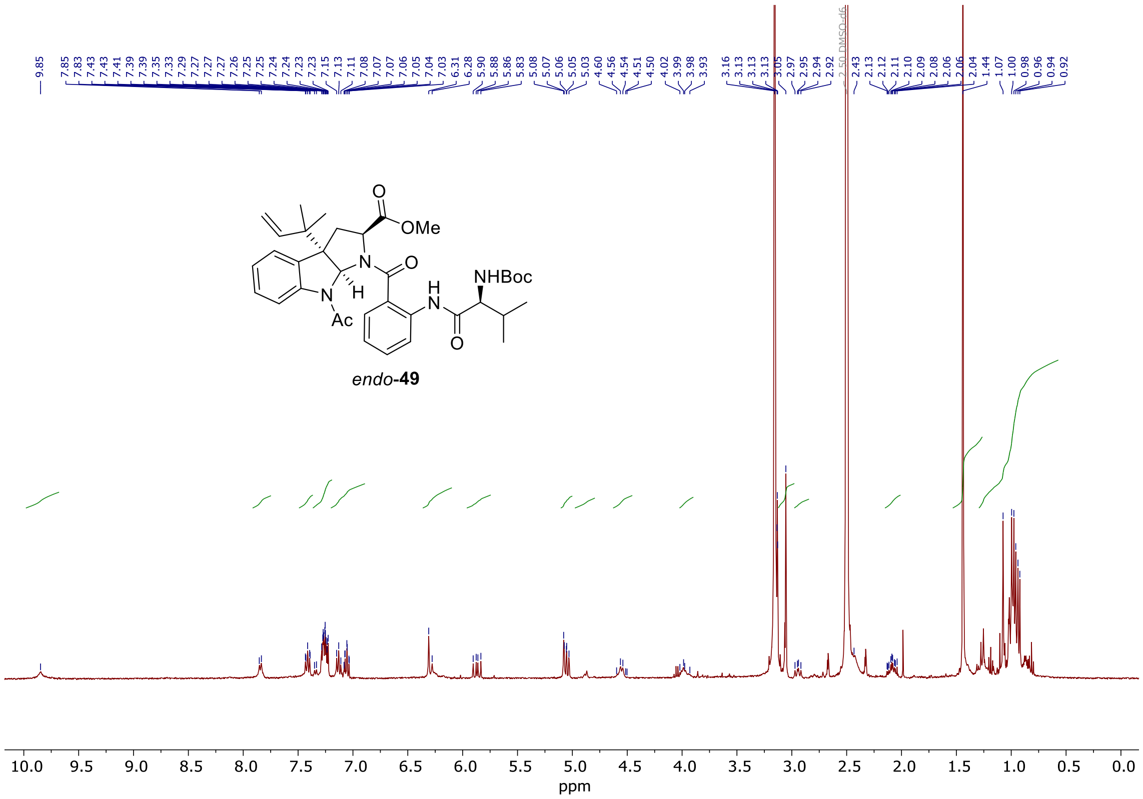

HPLC trace of ***endo*-68**. Conditions used in the chromatogram shown: Scharlau, C18 Kromaphase 100, 5 µm, 250 x 4.6 mm, gradient from CH_3CN/H_2O 30% to CH_3CN 100% in 20 min, 1.0 mL/min.

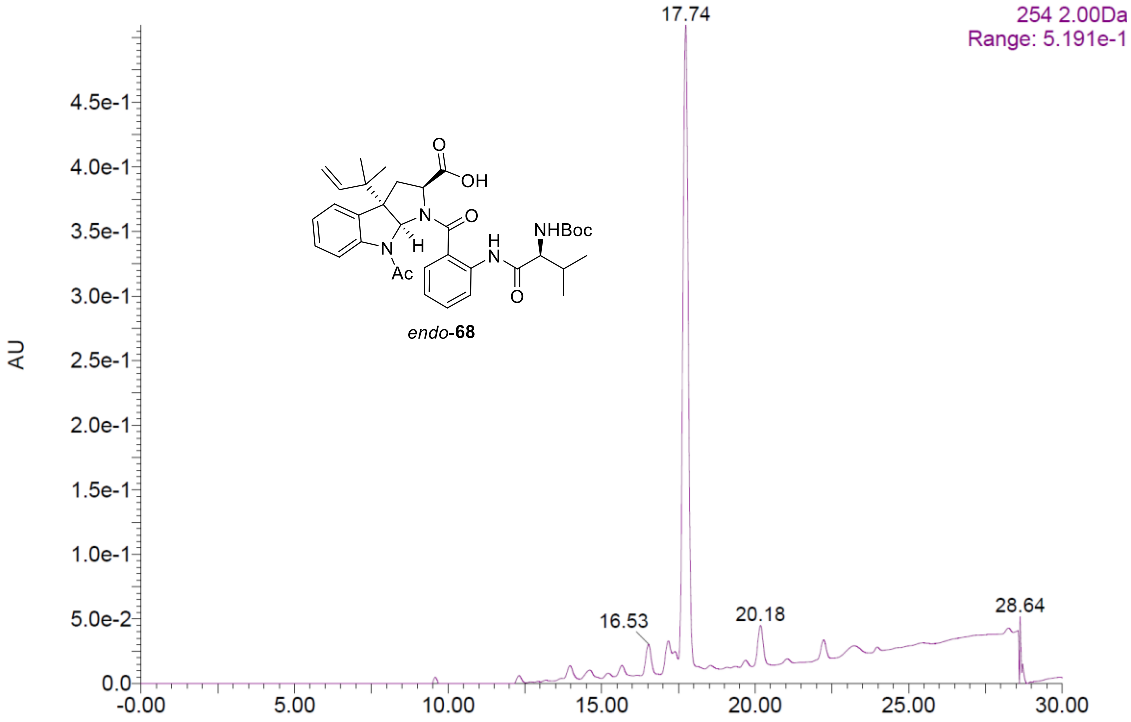

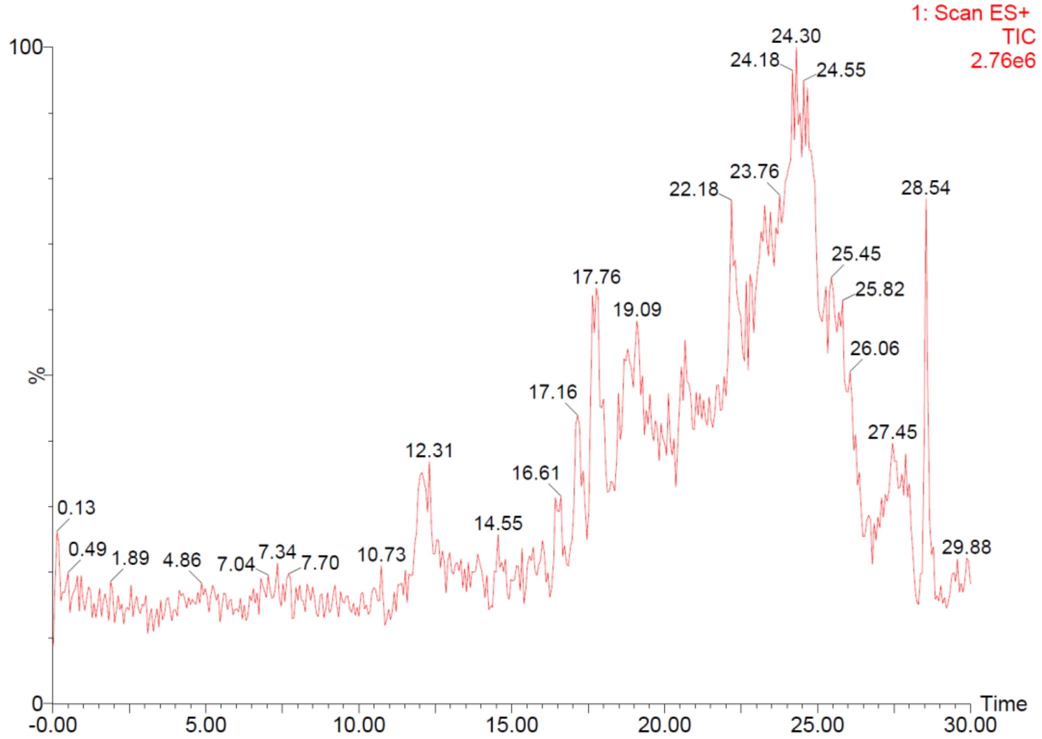
1: Scan ES+
TIC
2.76e6
100
%
0
24.30
24.18
24.55
23.76
22.18
28.54
25.45
17.76
25.82
19.09
26.06
17.16
27.45
12.31
16.61
0.13
14.55
0.49
1.89
4.86
7.04
7.34
7.70
10.73
29.88
Time
-0.00
5.00
10.00
15.00
20.00
25.00
30.00

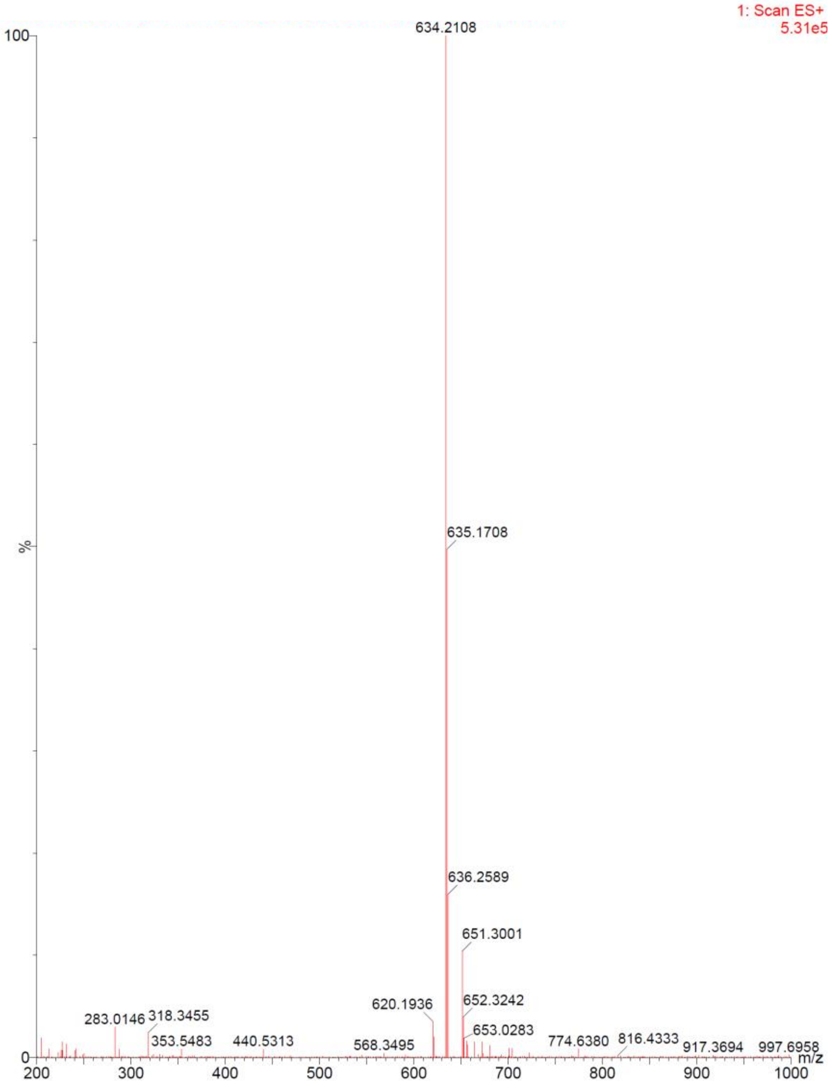
1: Scan ES+
5.31e5
100
%
0
634.2108
635.1708
636.2589
651.3001
620.1936
652.3242
283.0146
318.3455
653.0283
353.5483
440.5313
568.3495
774.6380
816.4333
917.3694
997.6958
m/z
200
300
400
500
600
700
800
900
1000

^{1}H NMR (400 MHz, DMSO-d_6, 383 K)

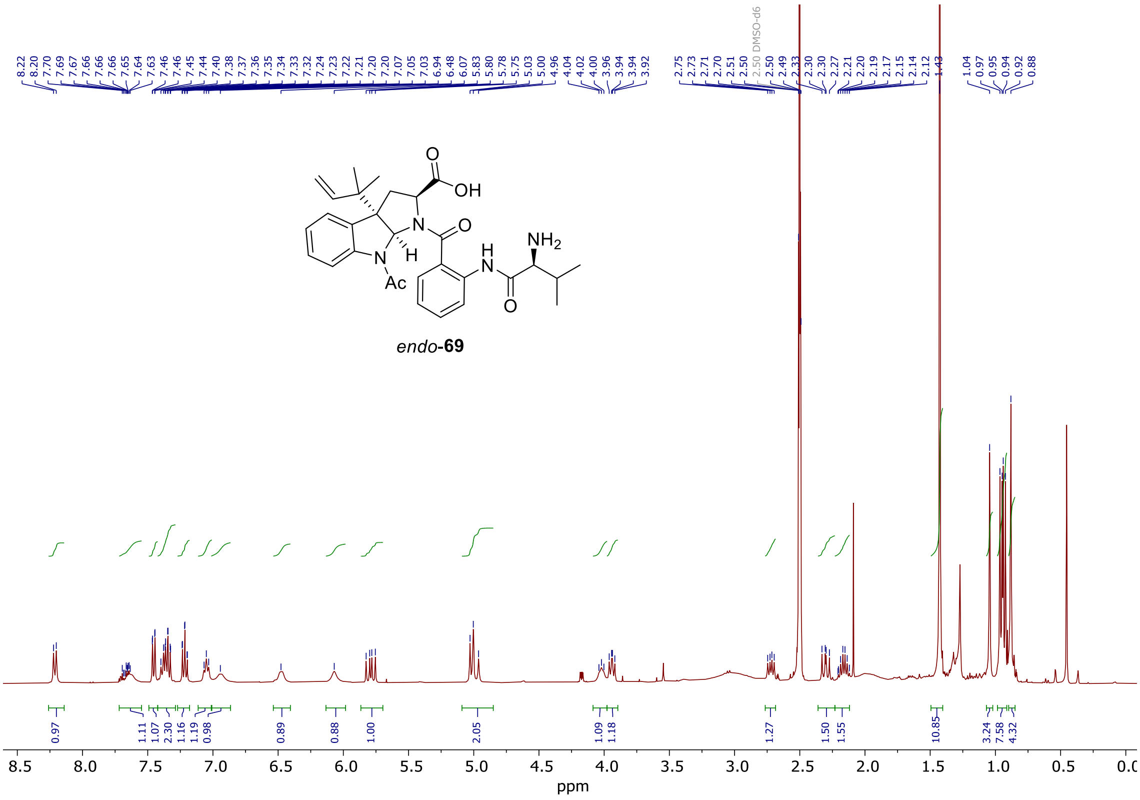

^{13}C{^{1}H} NMR (101 MHz, DMSO-d_6, 383 K)

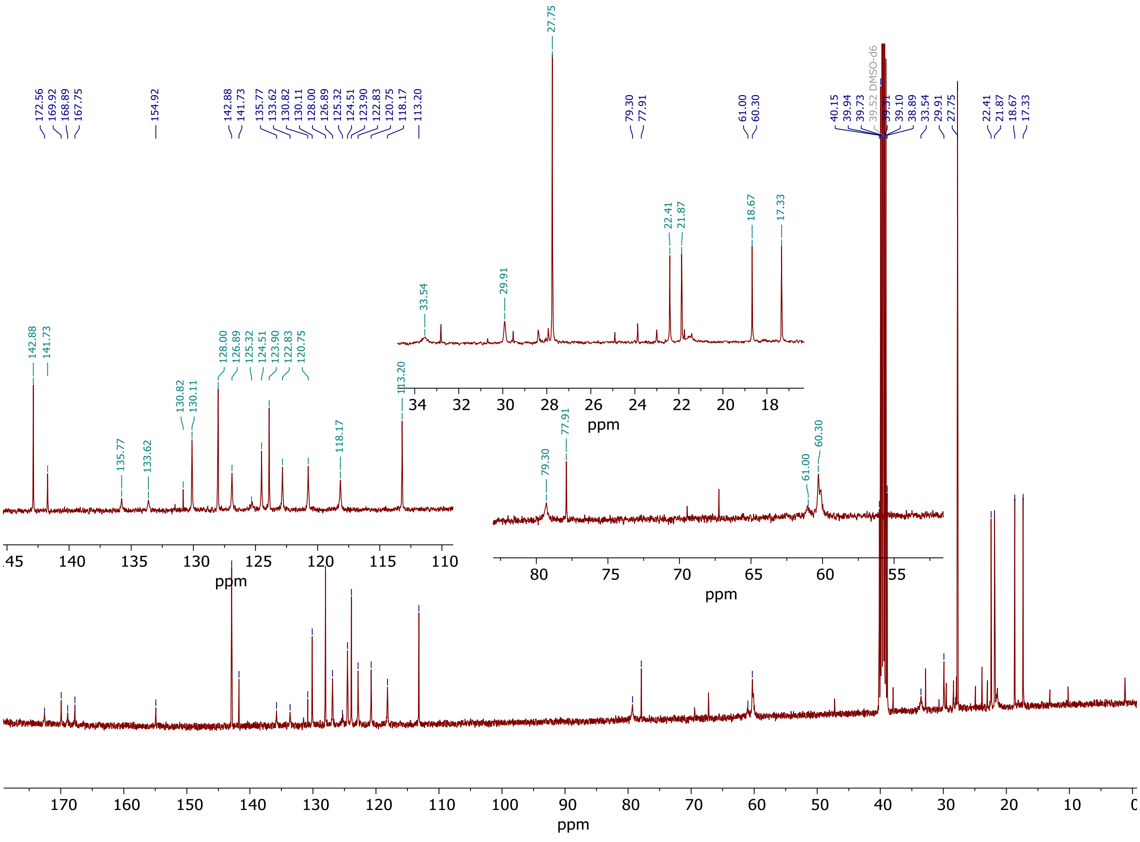

HPLC trace of *endo*-69. Conditions used in the chromatogram shown: Scharlau, C18 Kromaphase 100, 5 µm, 250 x 4.6 mm, gradient from CH_3CN/H_2O 50% to CH_3CN 100% in 20 min, 1.0 mL/min.

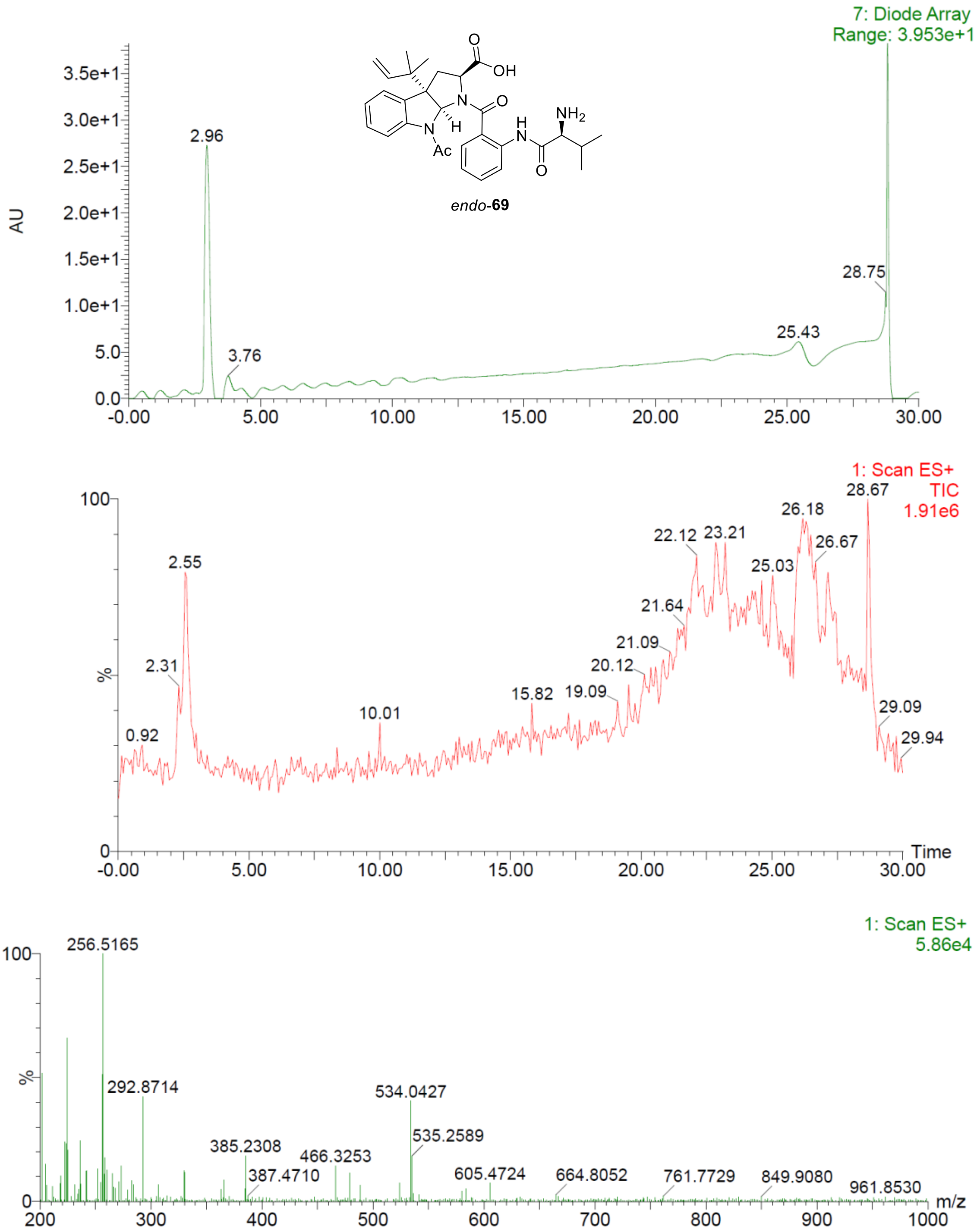

HPLC trace of *exo*-68. Conditions used in the chromatogram shown: Scharlau, C18 Kromaphase 100, 5 µm, 250 x 4.6 mm, gradient from CH_3CN/H_2O 30% to CH_3CN 100% in 20 min, 1.0 mL/min.

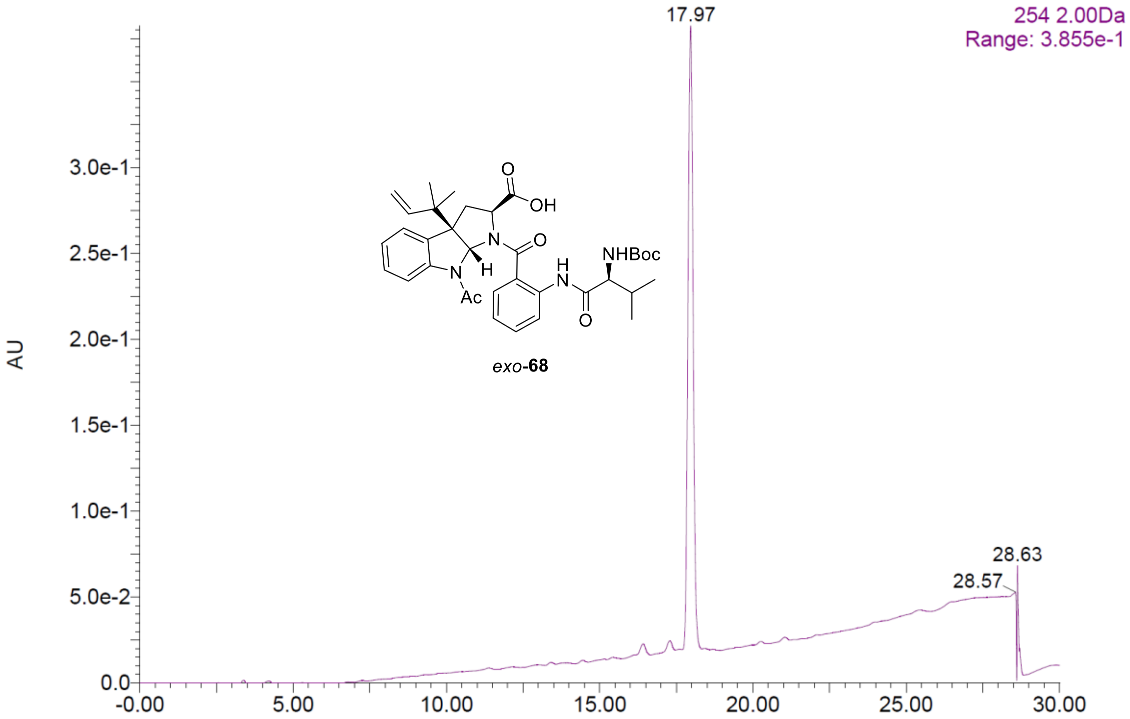

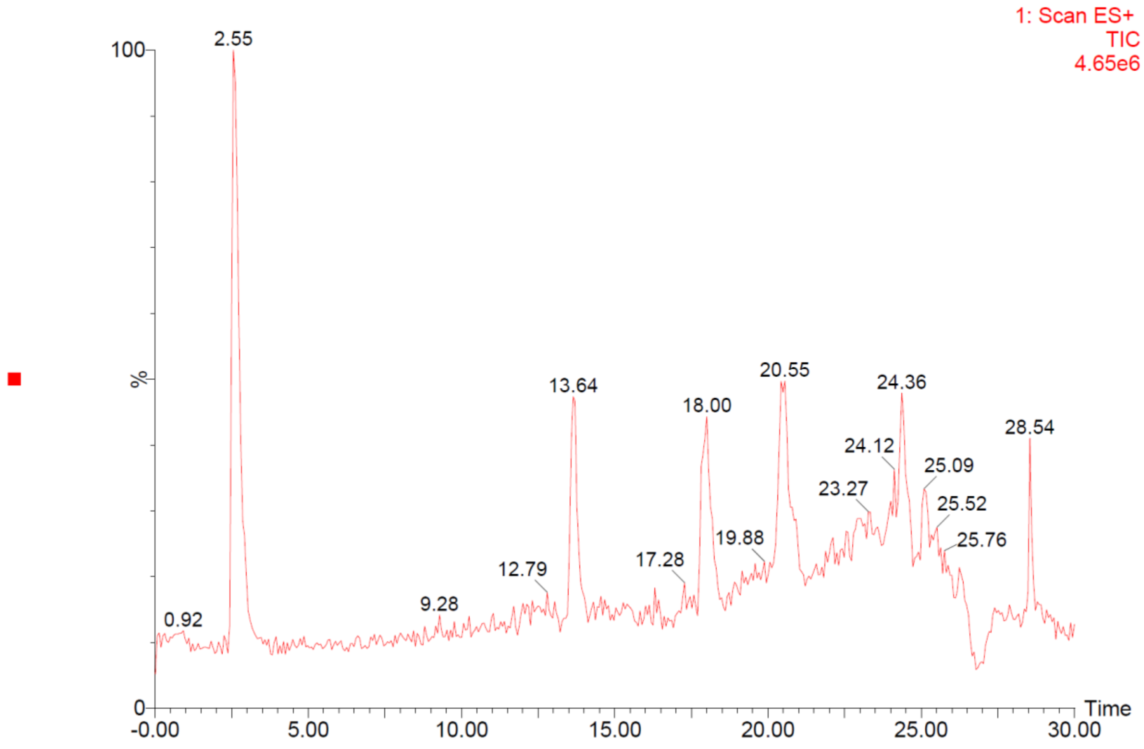

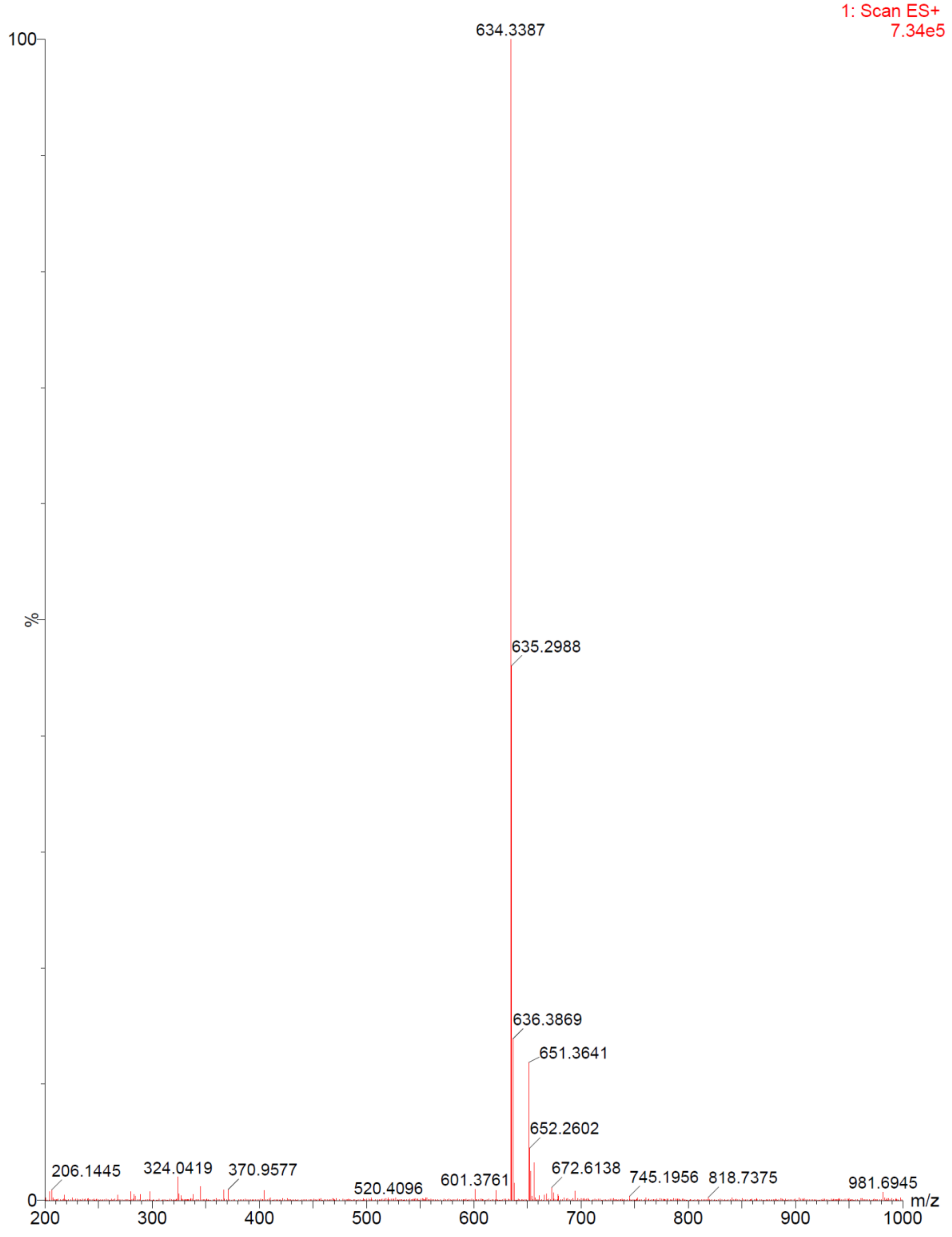

1: Scan ES+
7.34e5
634.3387
635.2988
636.3869
651.3641
652.2602
206.1445
324.0419
370.9577
520.4096
601.3761
672.6138
745.1956
818.7375
981.6945
100
%
0
200
300
400
500
600
700
800
900
1000
m/z

HPLC trace of *exo*-69. Conditions used in the chromatogram shown: Scharlau, C18 Kromaphase 100, 5 µm, 250 x 4.6 mm, gradient from CH_3CN/H_2O 30% to CH_3CN 100% in 20 min, 1.0 mL/min.

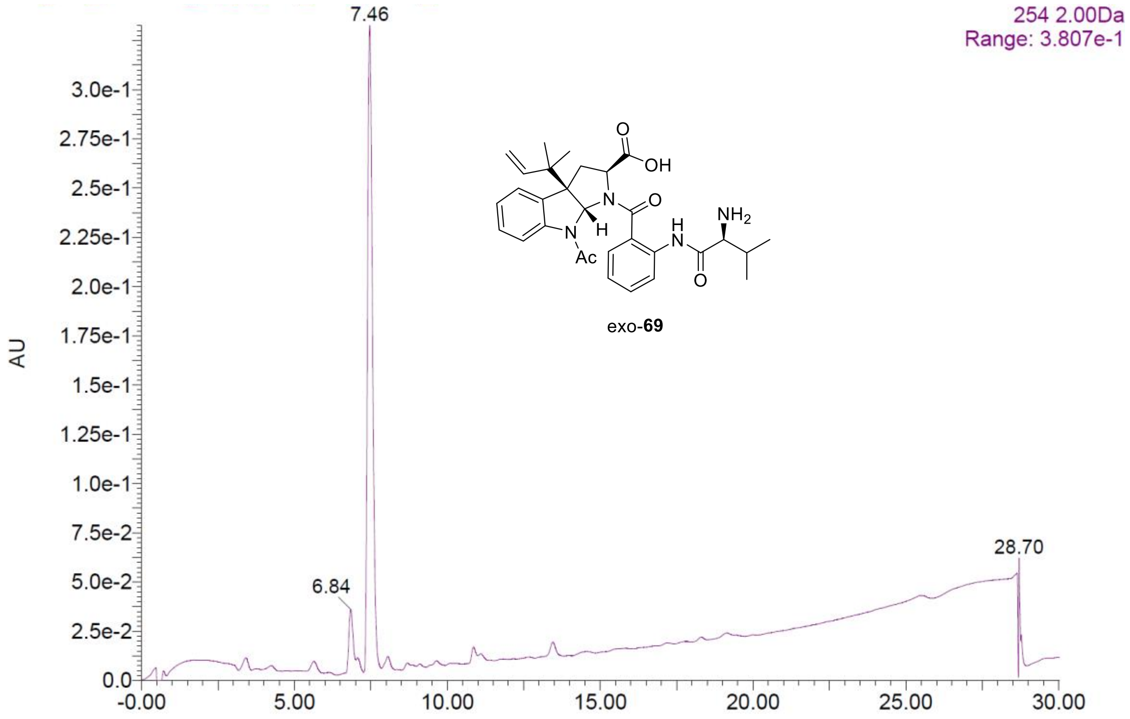

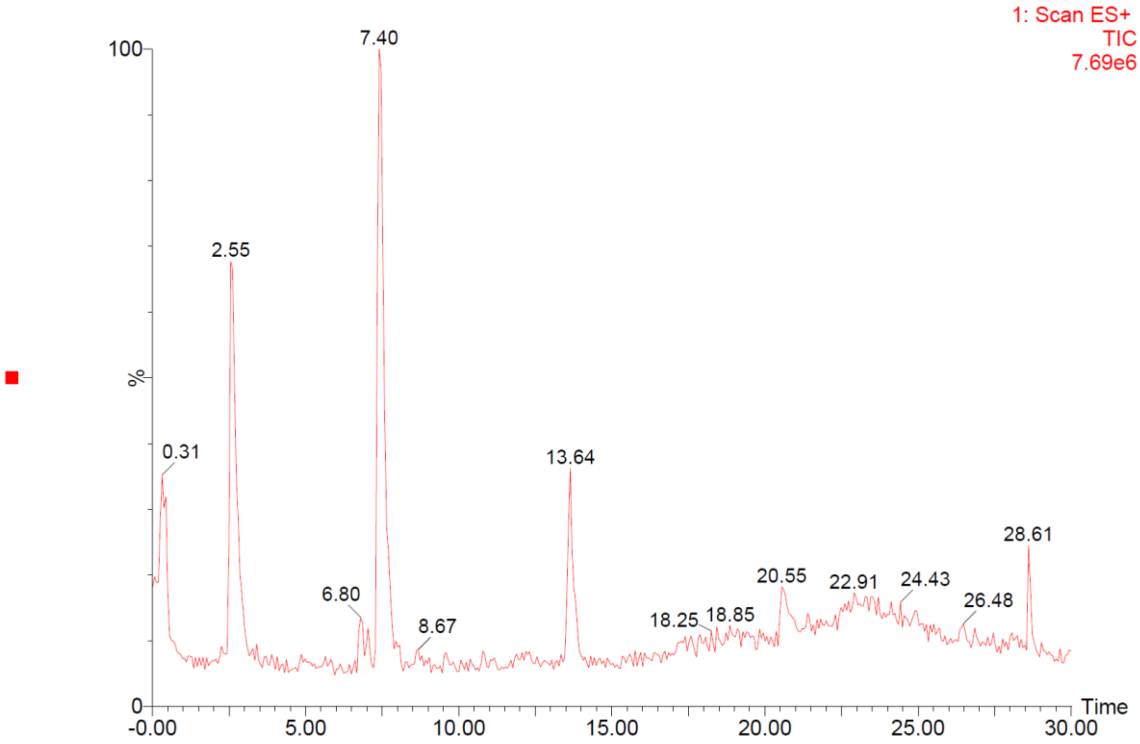

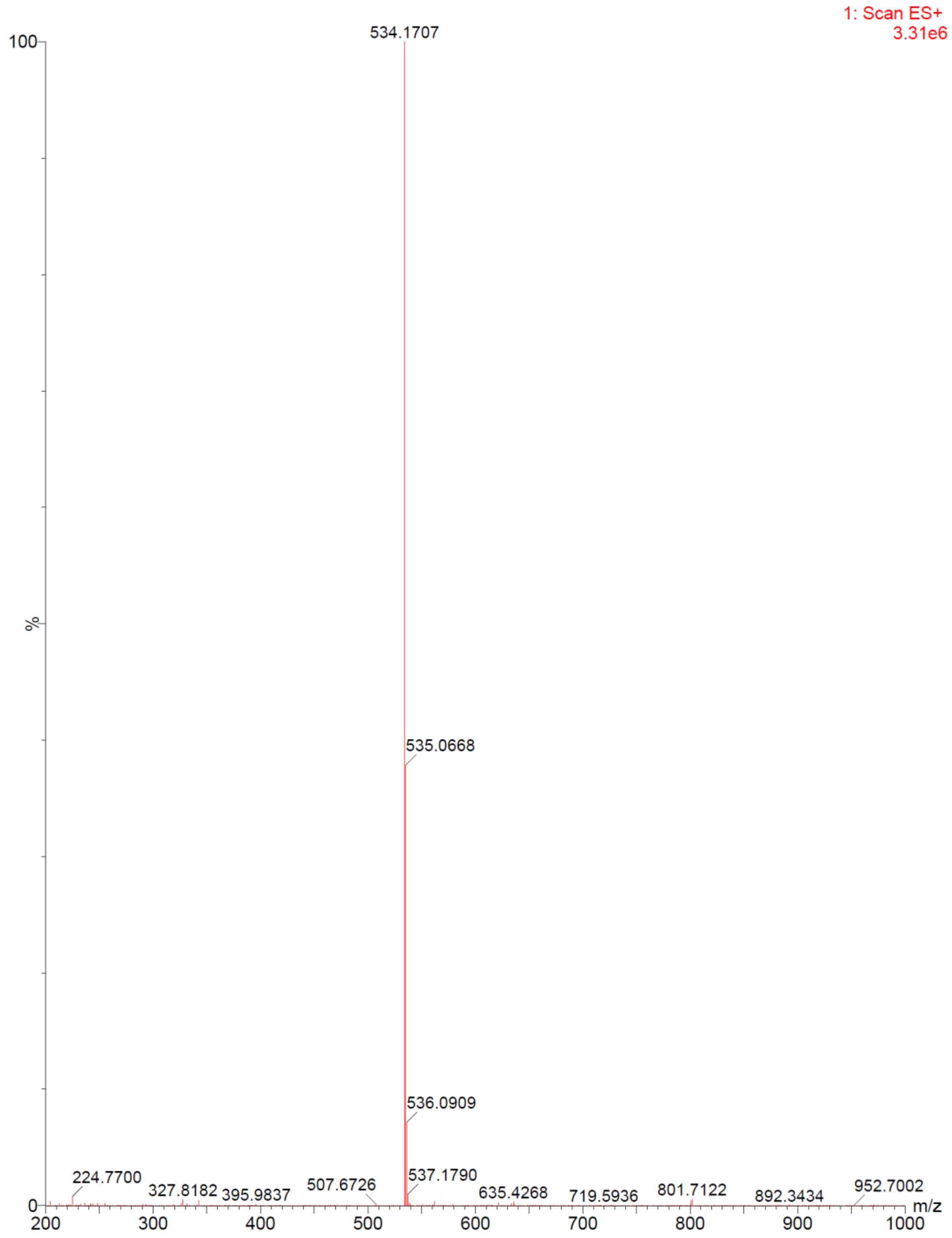
1: Scan ES+
3.31e6
534.1707
535.0668
536.0909
537.1790
224.7700
327.8182
395.9837
507.6726
635.4268
719.5936
801.7122
892.3434
952.7002
100
%
0
200
300
400
500
600
700
800
900
1000
m/z

^{1}H NMR (400 MHz, DMSO-d_6, 343 K)

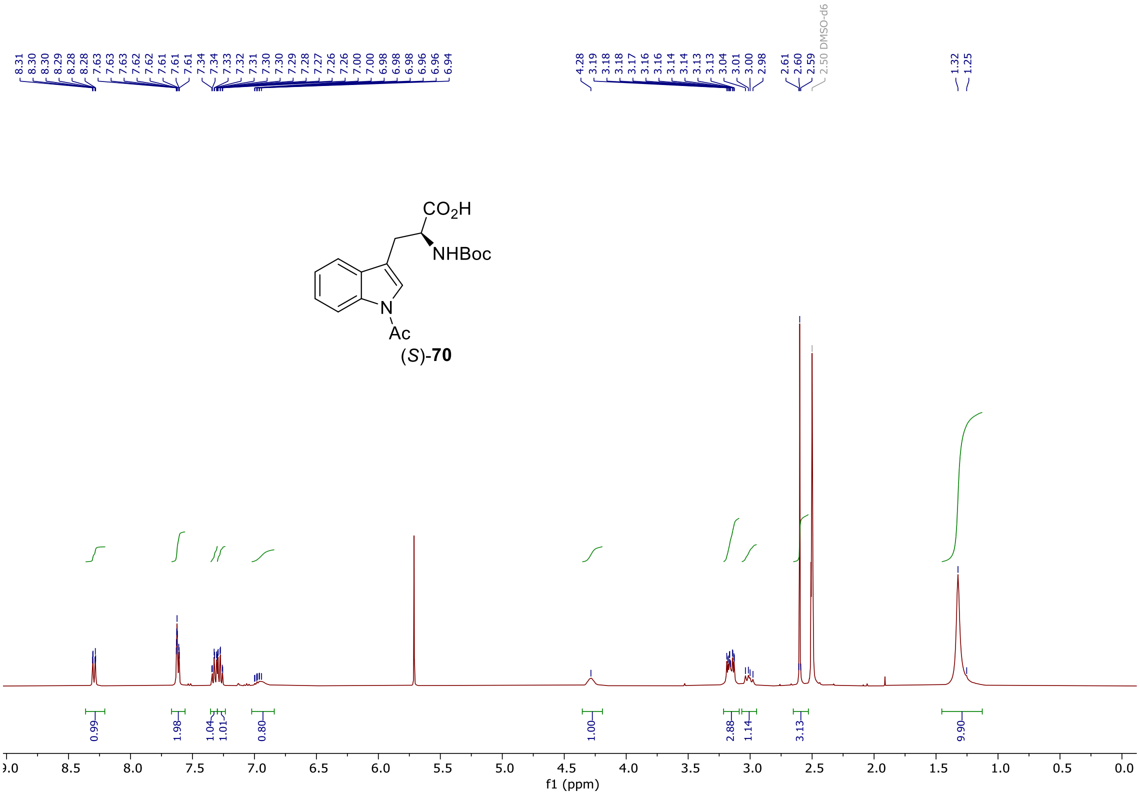

^{1}H NMR (400 MHz, DMSO-d_6, 343 K)

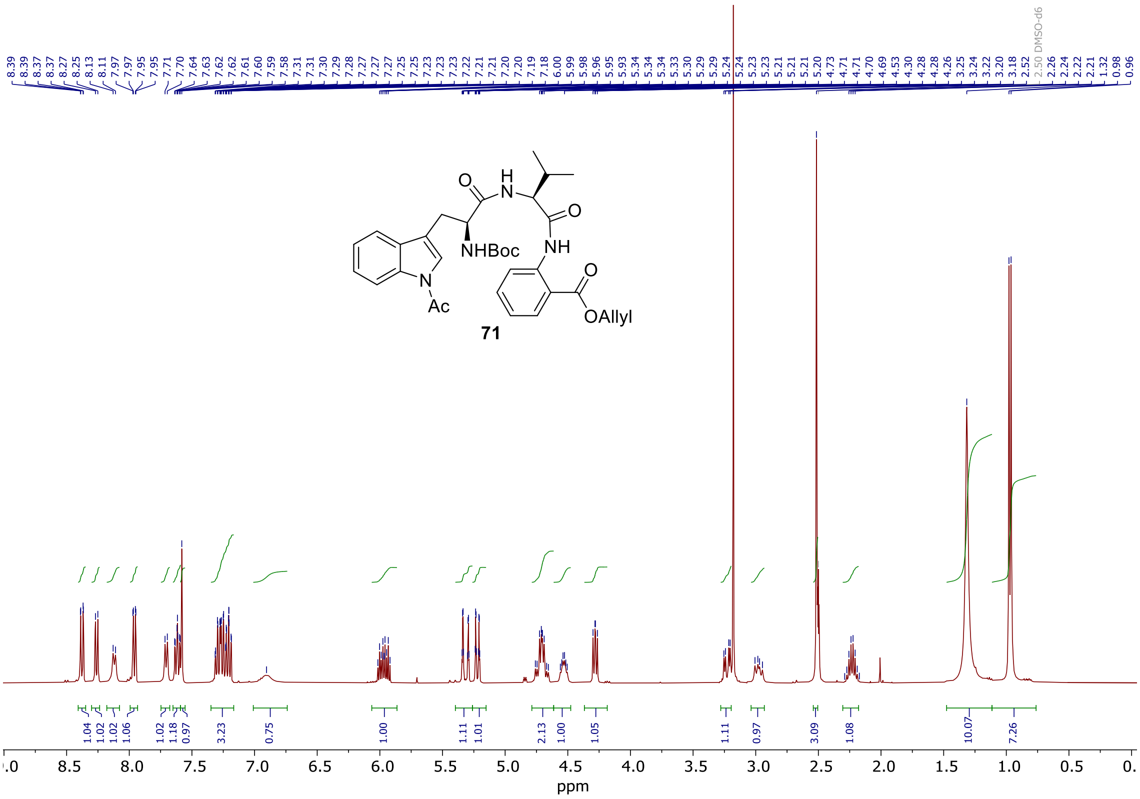

^{13}C{^{1}H} NMR (101 MHz, DMSO-d_6, 343 K)

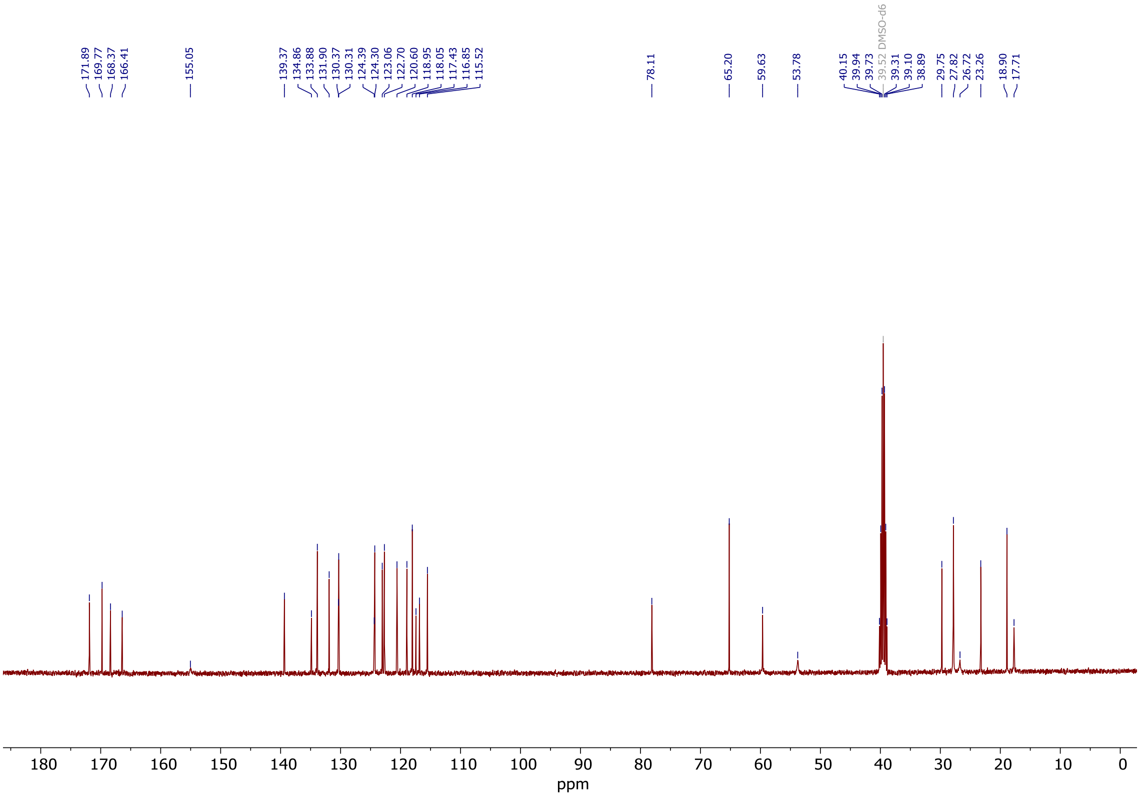

^{1}H NMR (400 MHz, DMSO-d_6, 343 K)

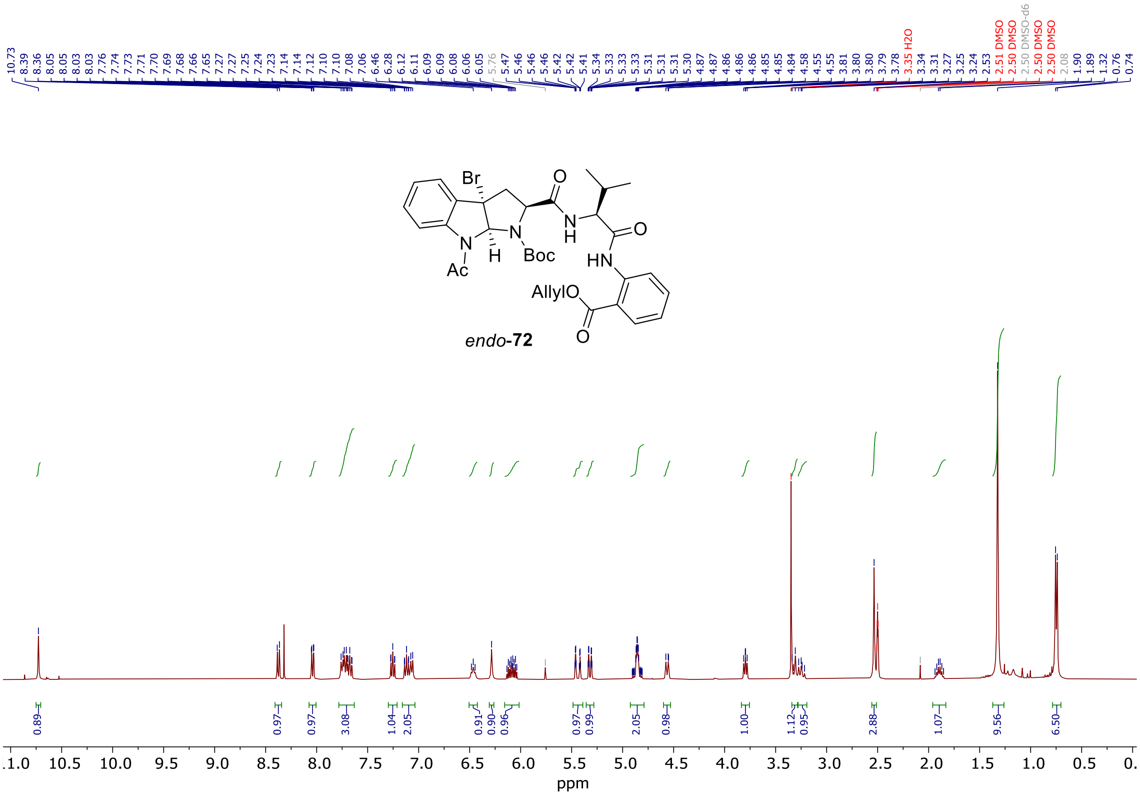

^{13}C{^{1}H} NMR (101 MHz, DMSO-d_6, 343 K)

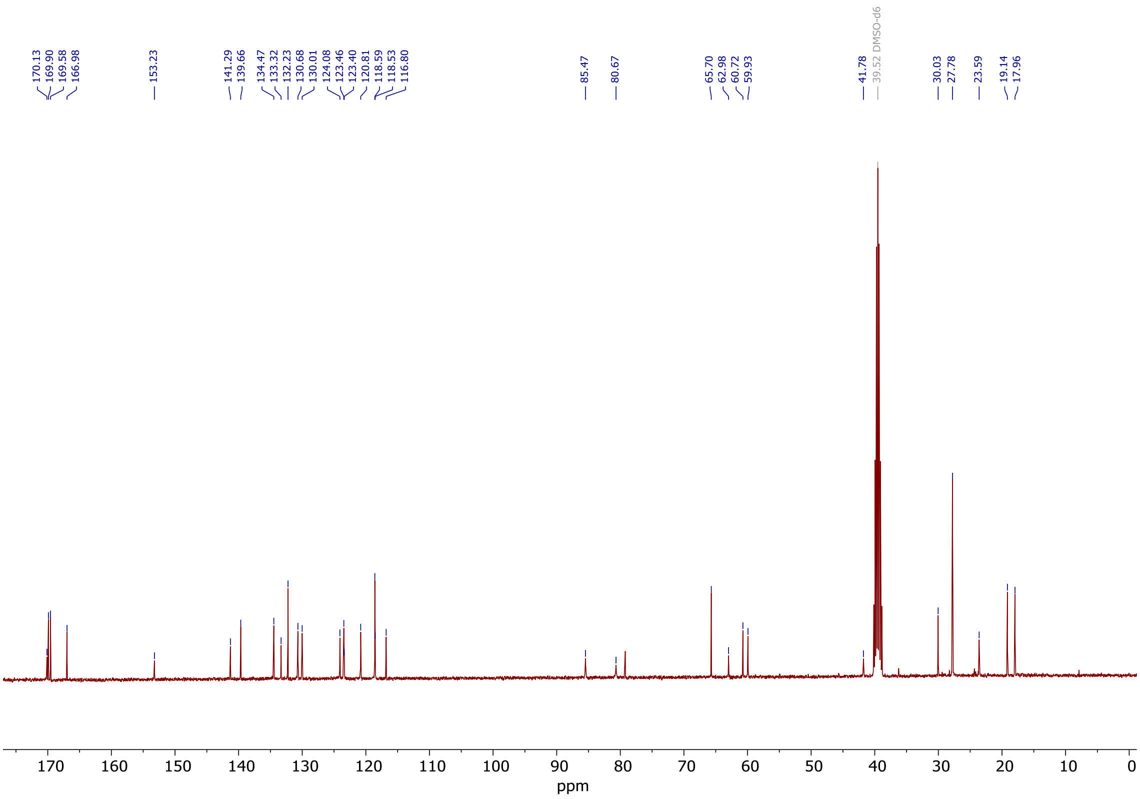

^{1}H NMR (400 MHz, DMSO-d_6, 343 K)

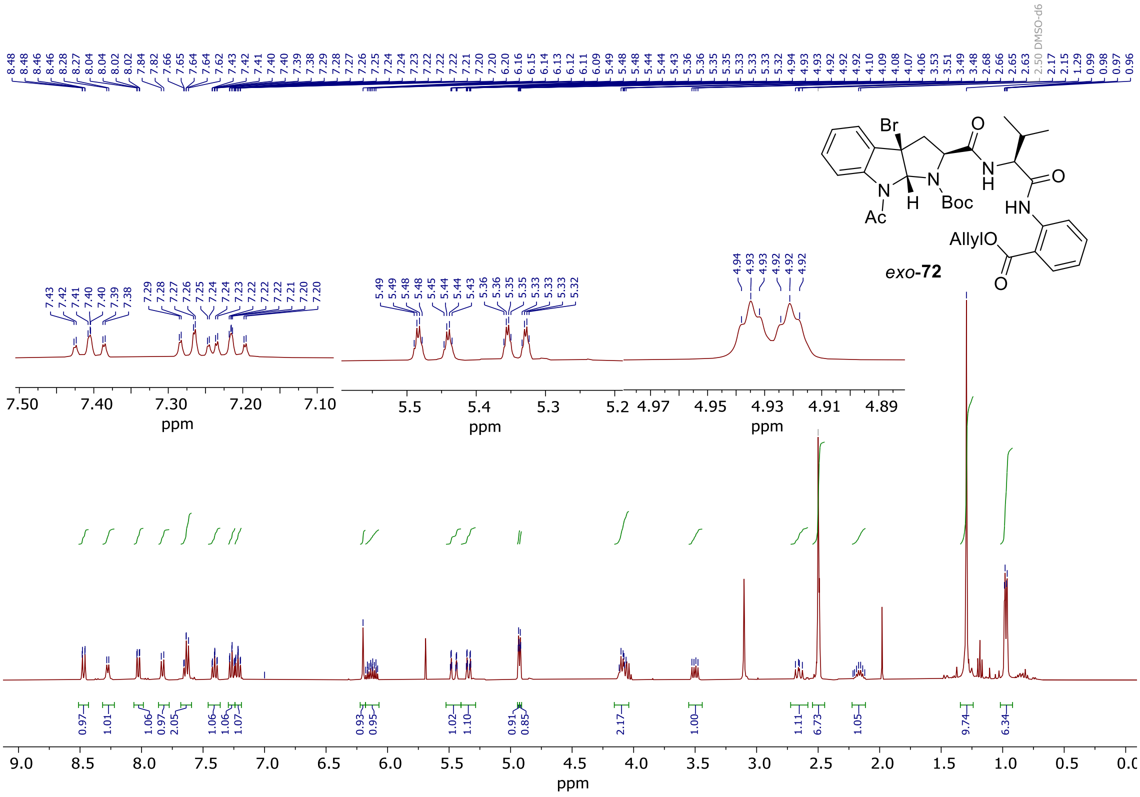

^{13}C{^{1}H} NMR (101 MHz, DMSO-d_6, 343 K)

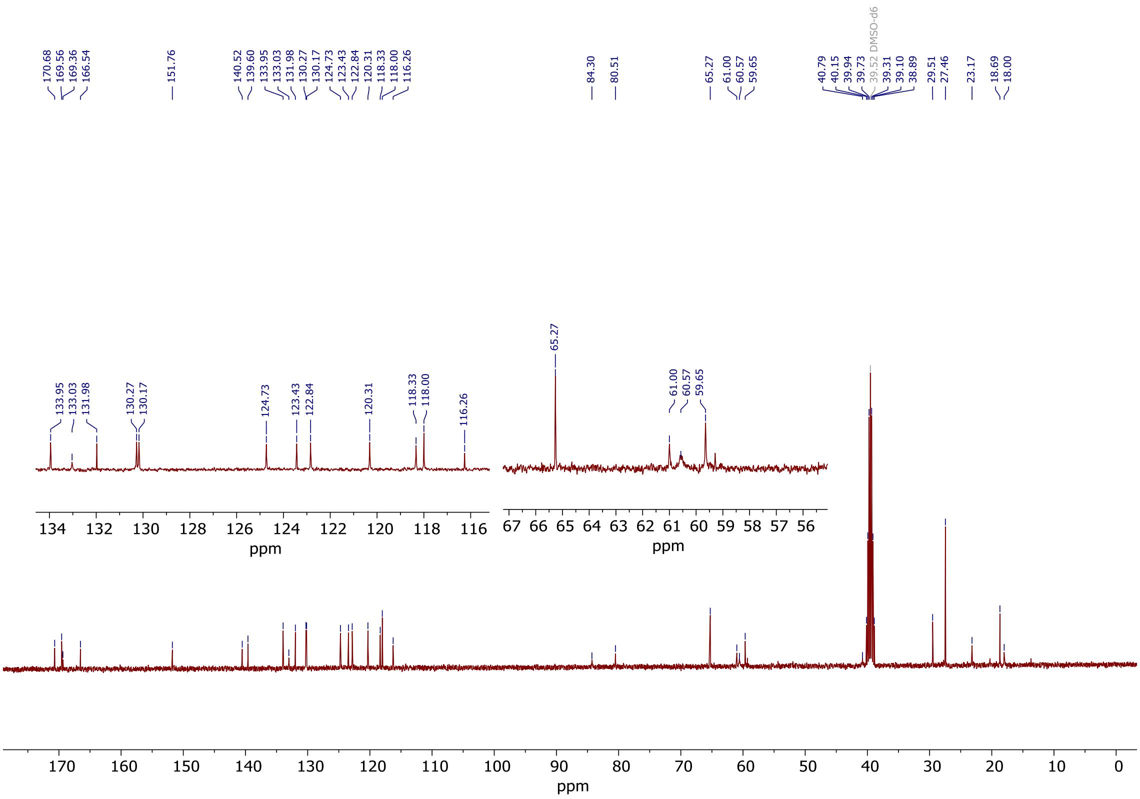

^{1}H NMR (400 MHz, DMSO-d_6, 343 K)

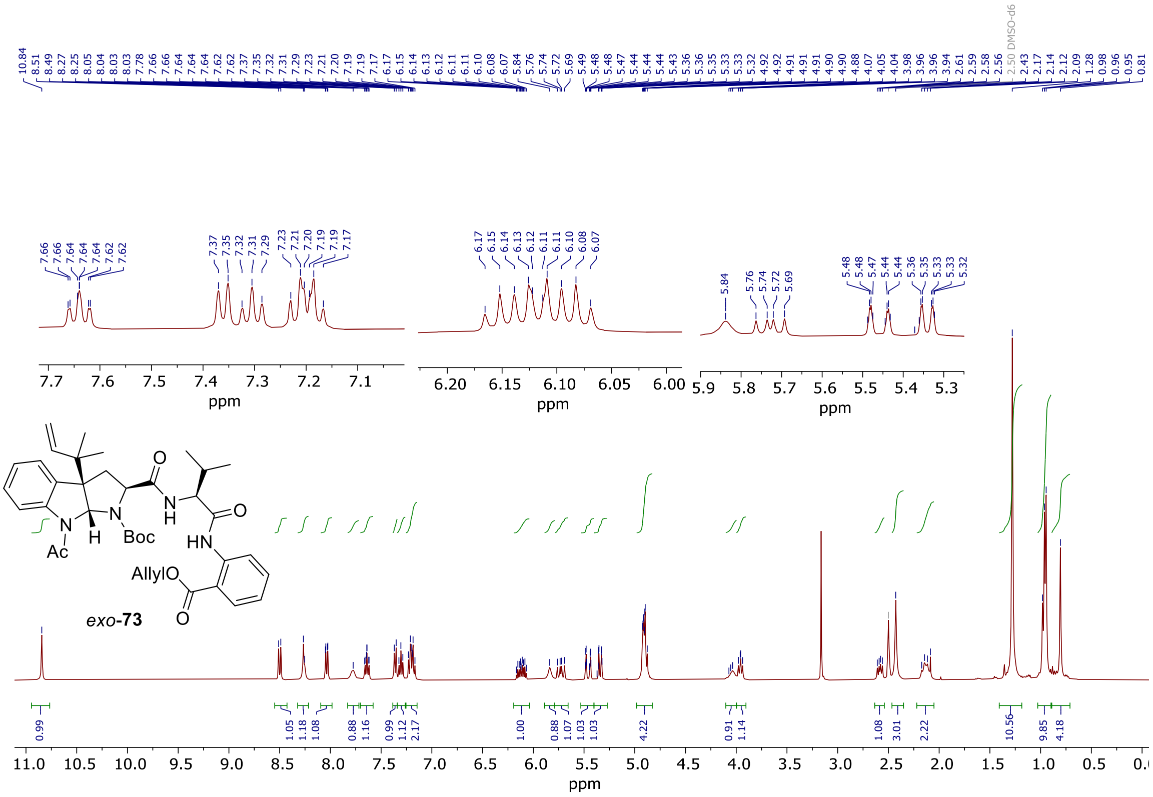

^{13}C{^{1}H} NMR (101 MHz, DMSO-d_6, 343 K)

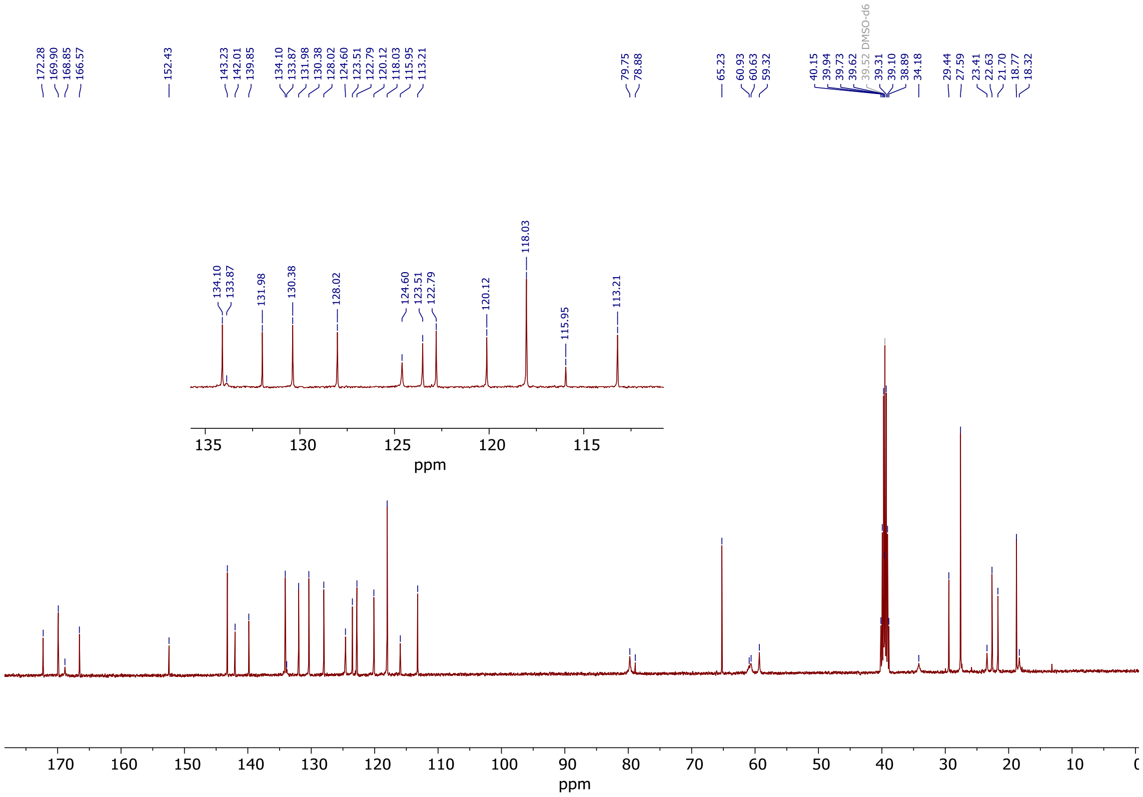

^{1}H NMR (400 MHz, DMSO-d_6, 343 K)

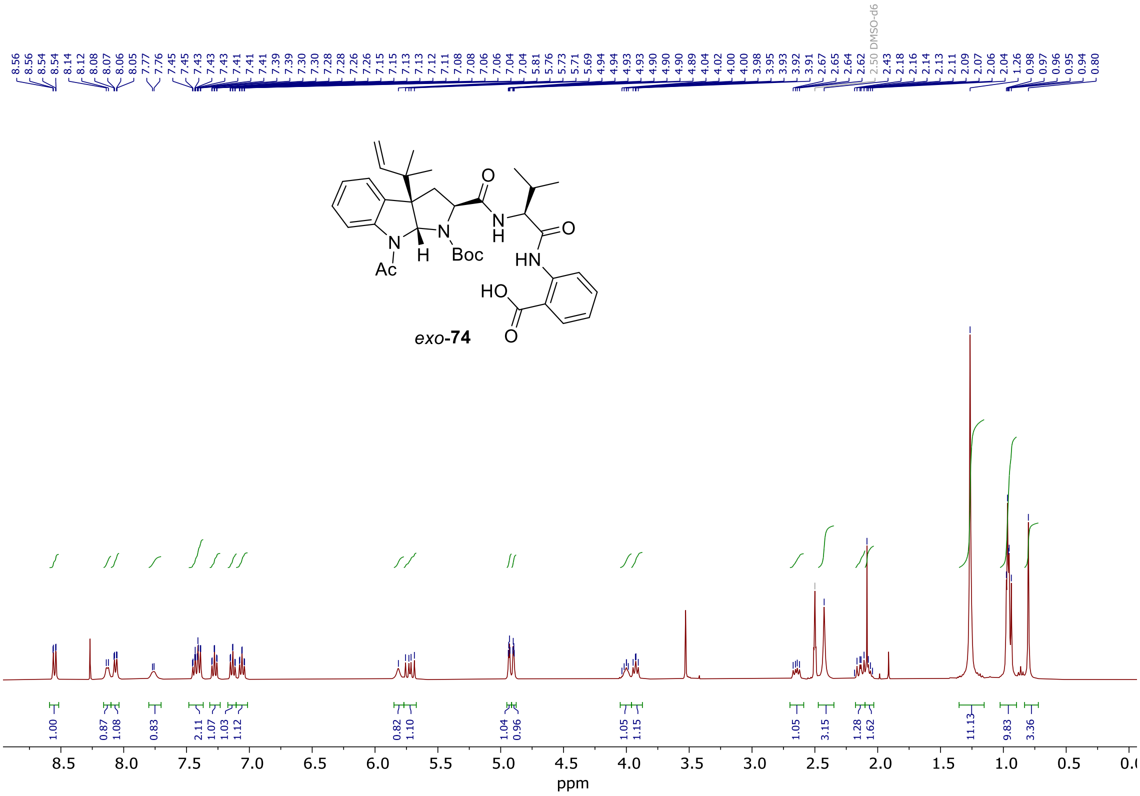

^{13}C{^{1}H} NMR (101 MHz, DMSO-d_6, 343 K)

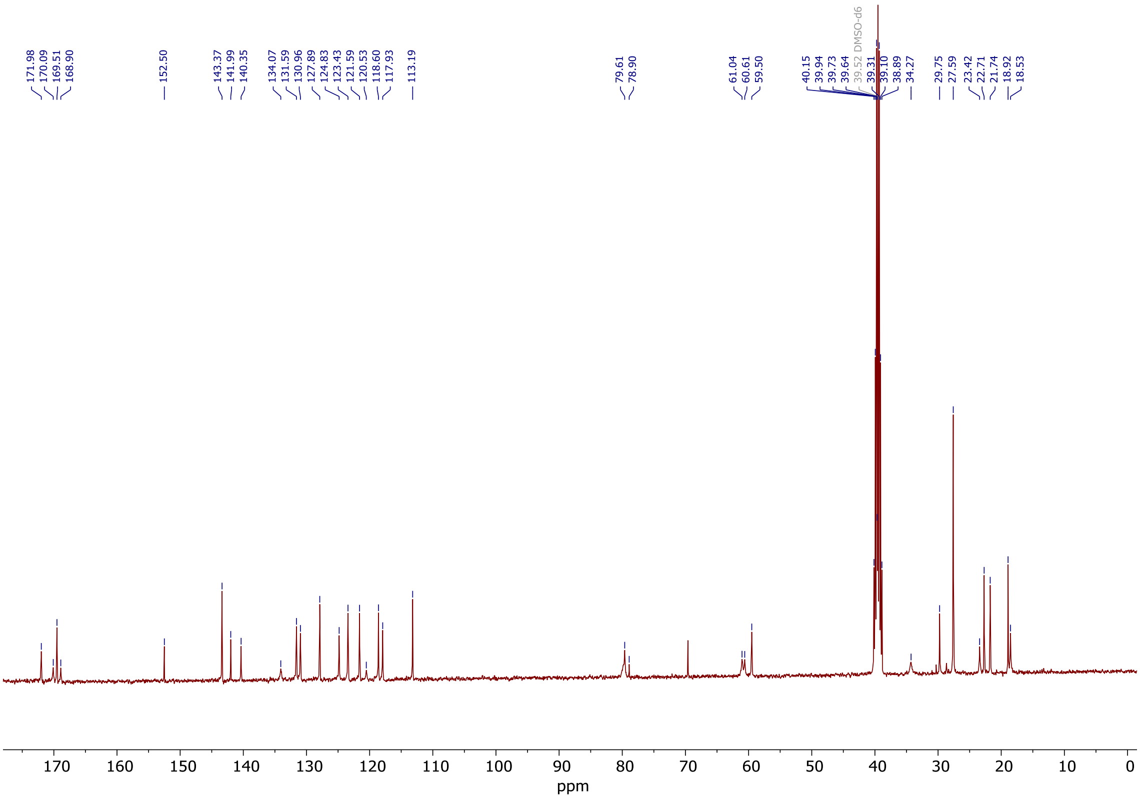

^{1}H NMR (400 MHz, $CDCl_3$)

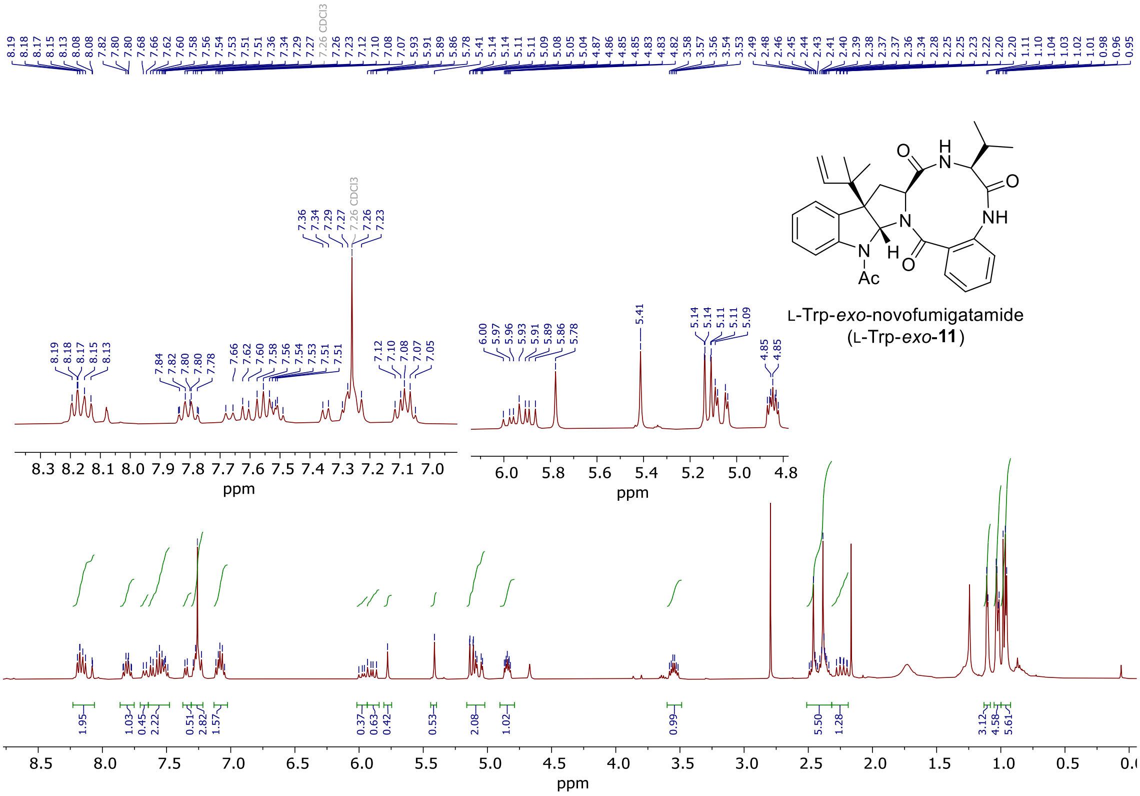

L-Trp-*exo*-novofumigatamide
(L-Trp-*exo*-**11**)

^{13}C{^{1}H} NMR (101 MHz, $CDCl_3$)

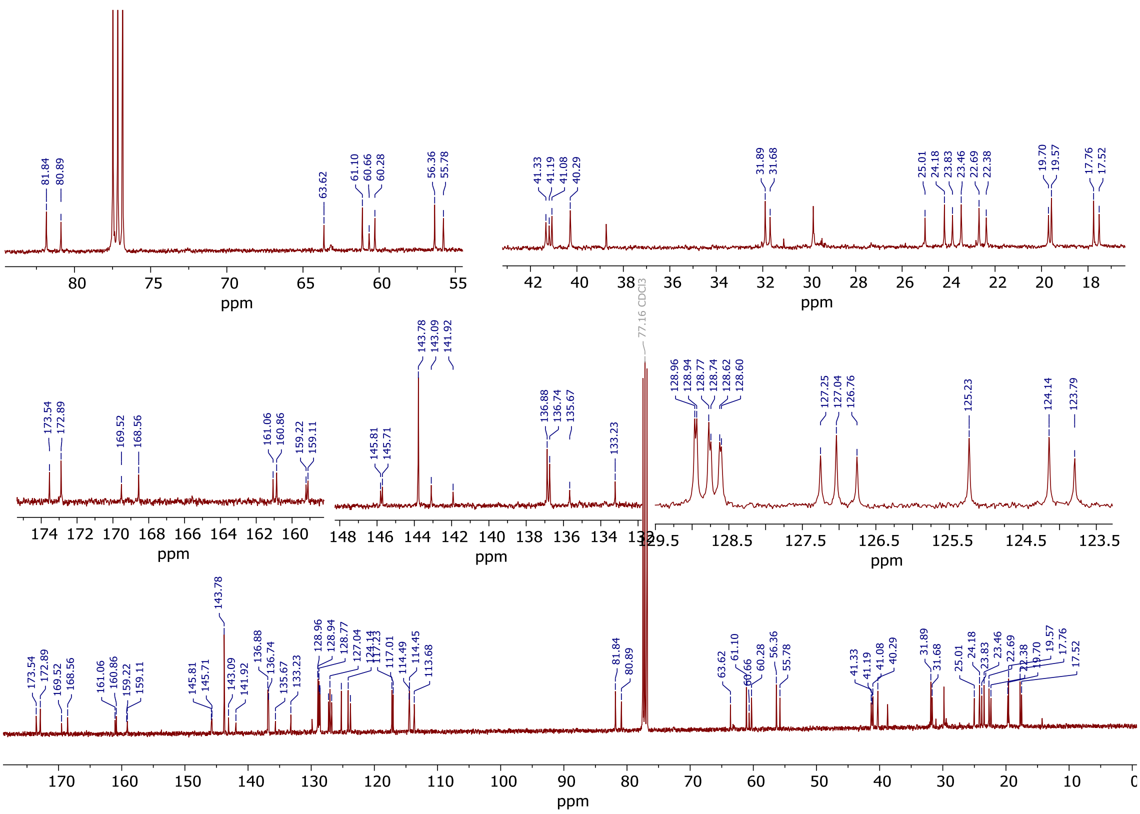

DEPT-135

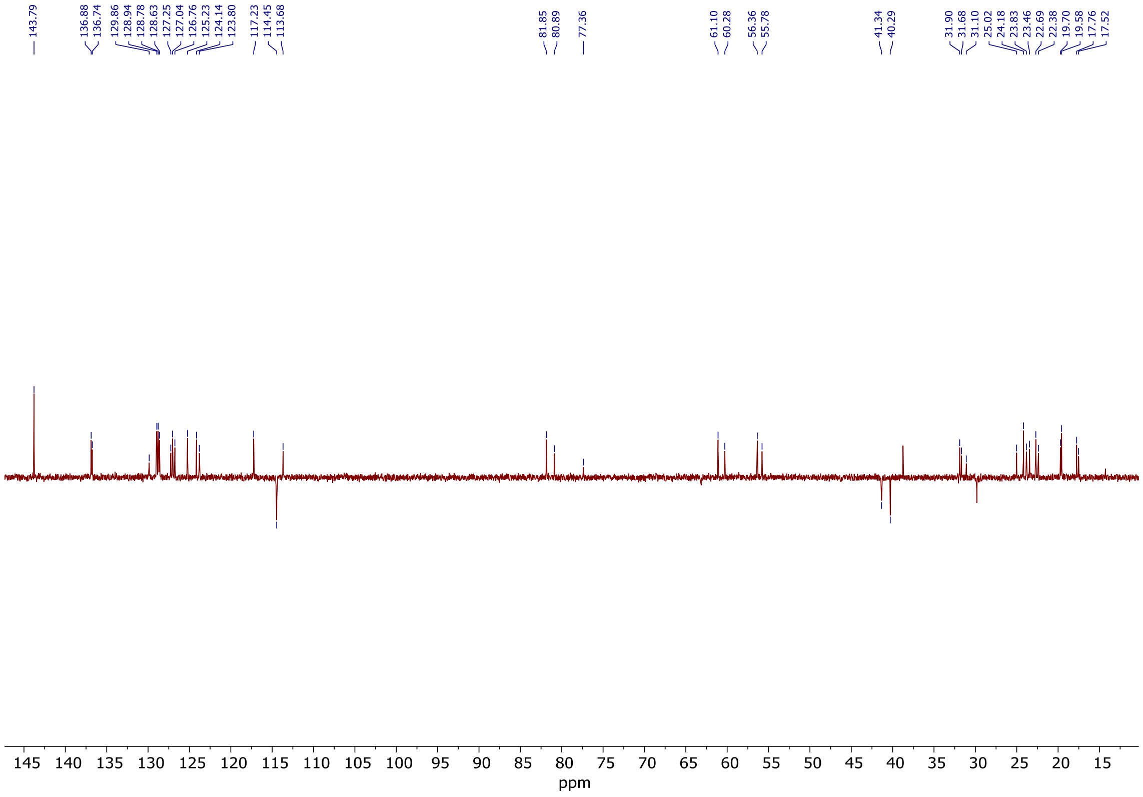

COSY

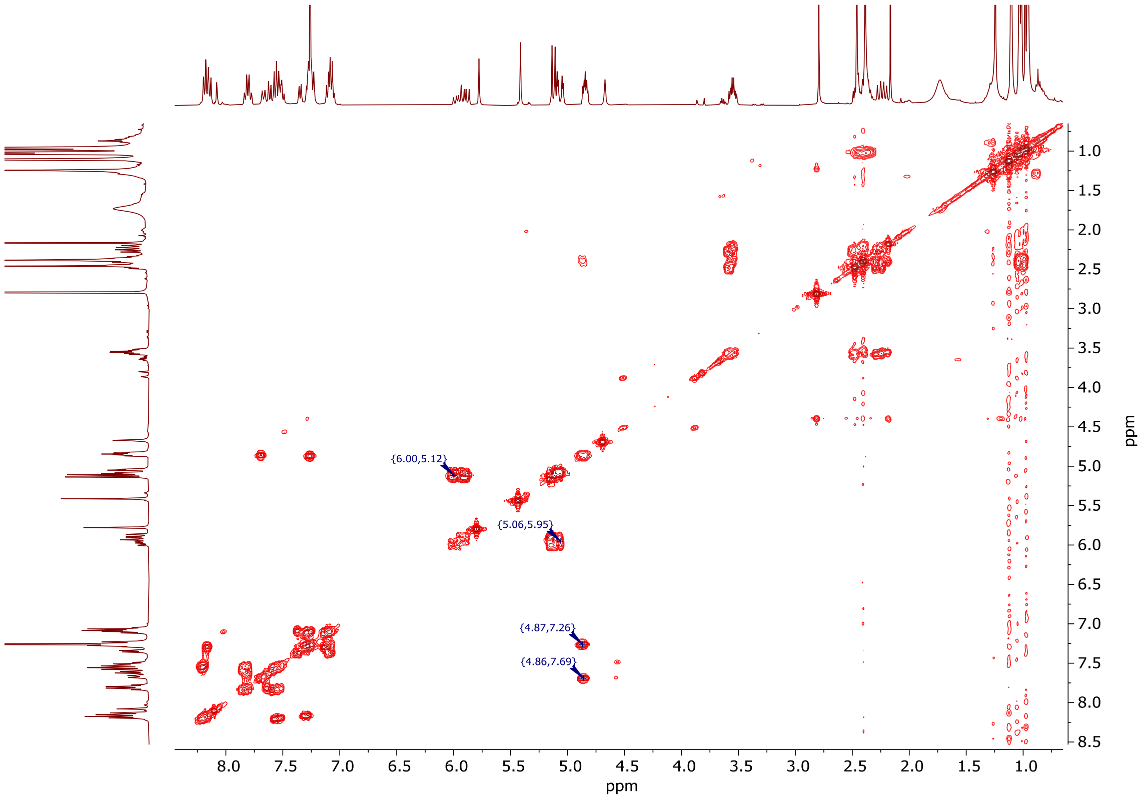

HSQC

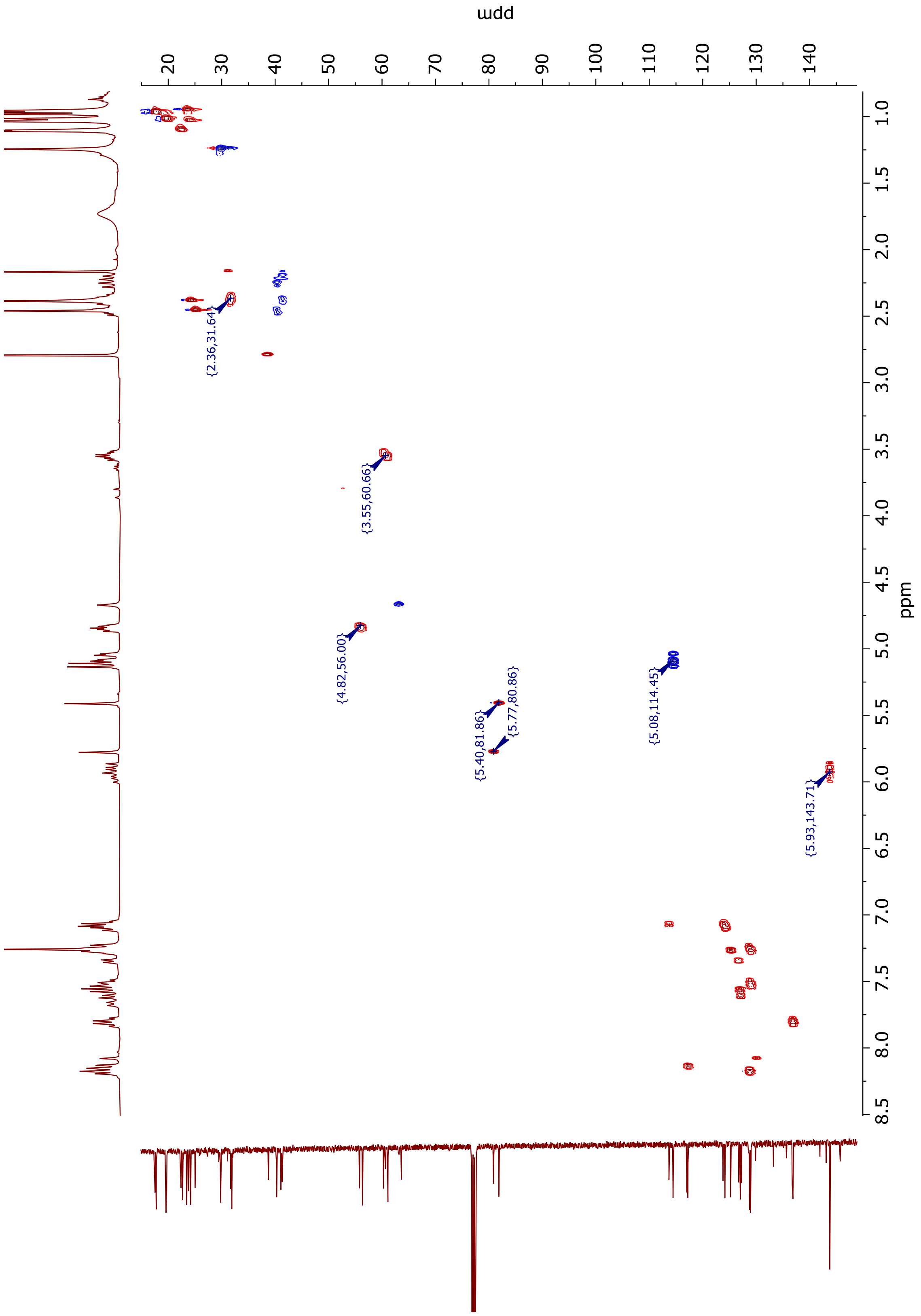

ppm
ppm
{2.36,31.64}
{3.55,60.66}
{4.82,56.00}
{5.40,81.86}
{5.77,80.86}
{5.08,114.45}
{5.93,143.71}

HMBC

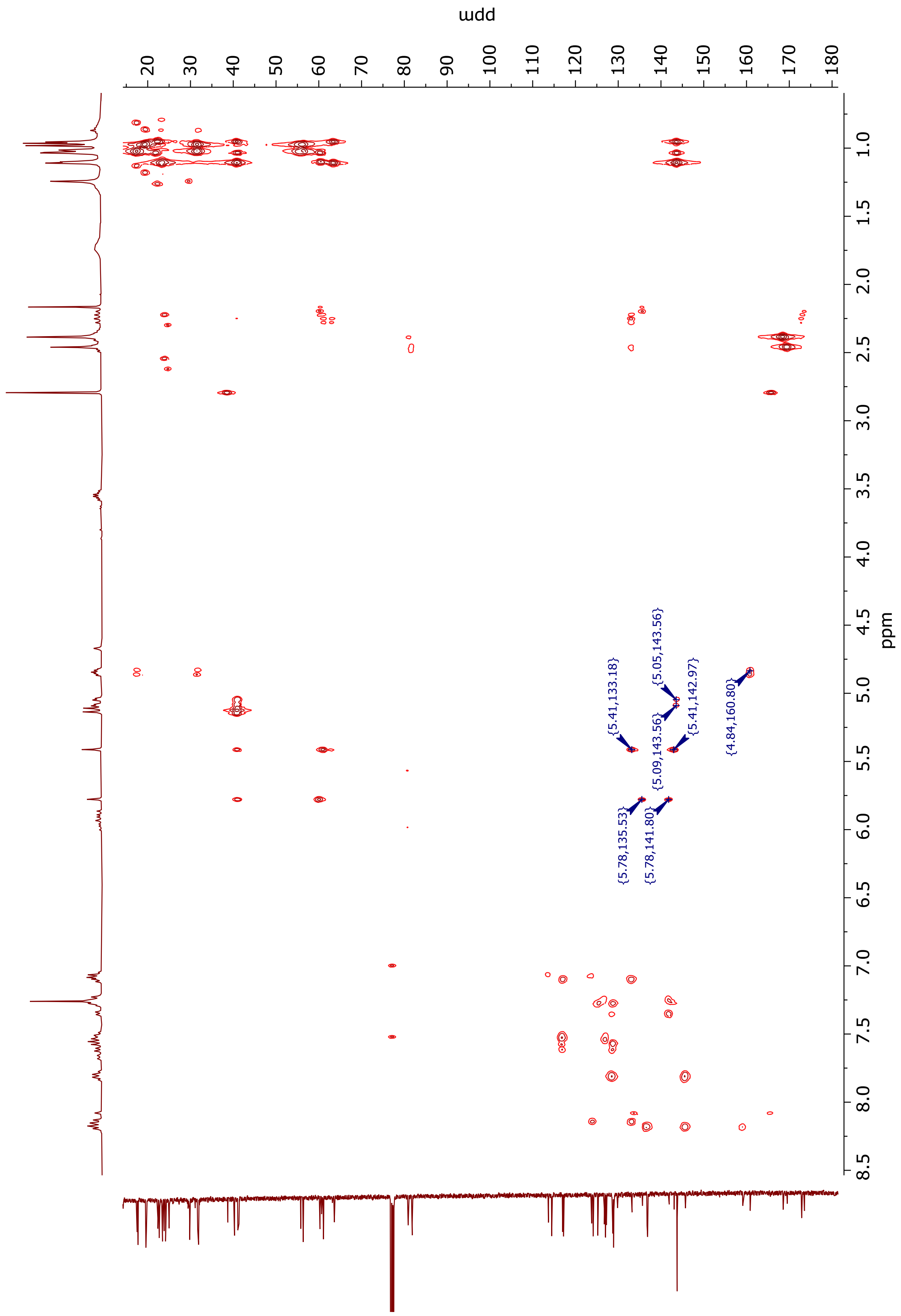
ppm
ppm
{5.41,133.18}
{5.05,143.56}
{5.41,142.97}
{4.84,160.80}
{5.09,143.56}
{5.78,135.53}
{5.78,141.80}

^{1}H NMR (400 MHz, DMSO-d_6, 343 K)

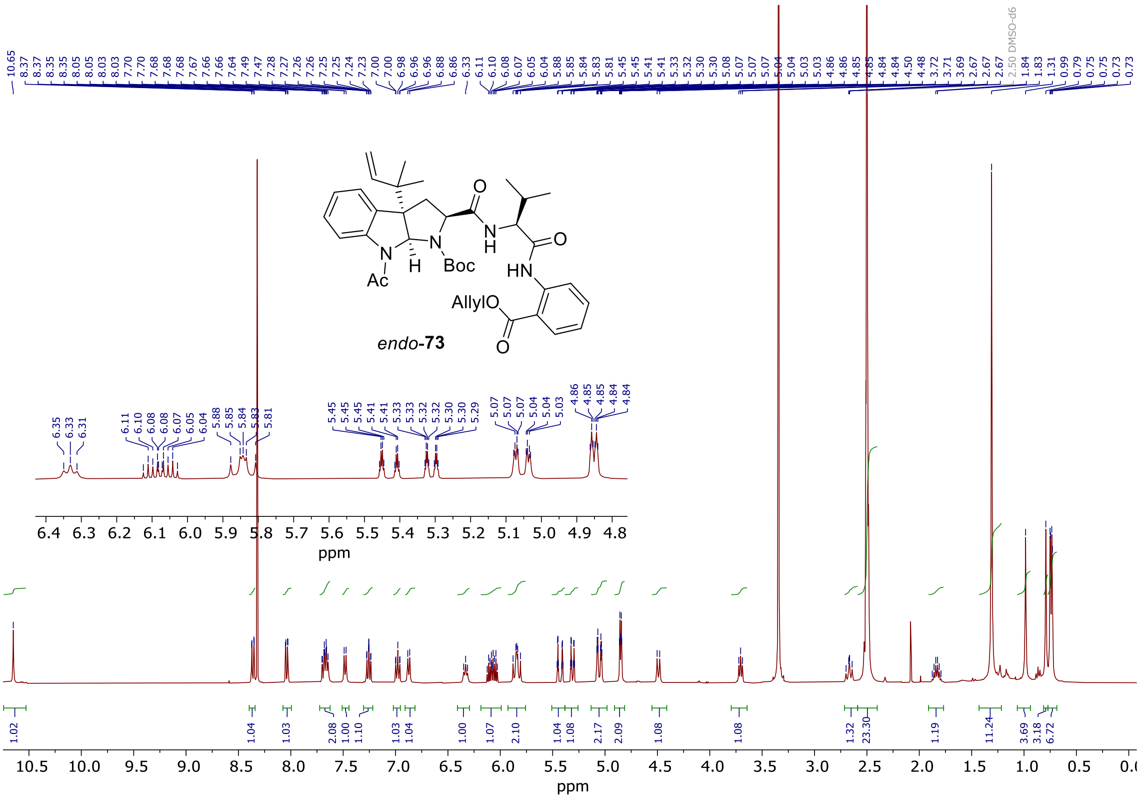

^{13}C{^{1}H} NMR (101 MHz, DMSO-d_6, 343 K)

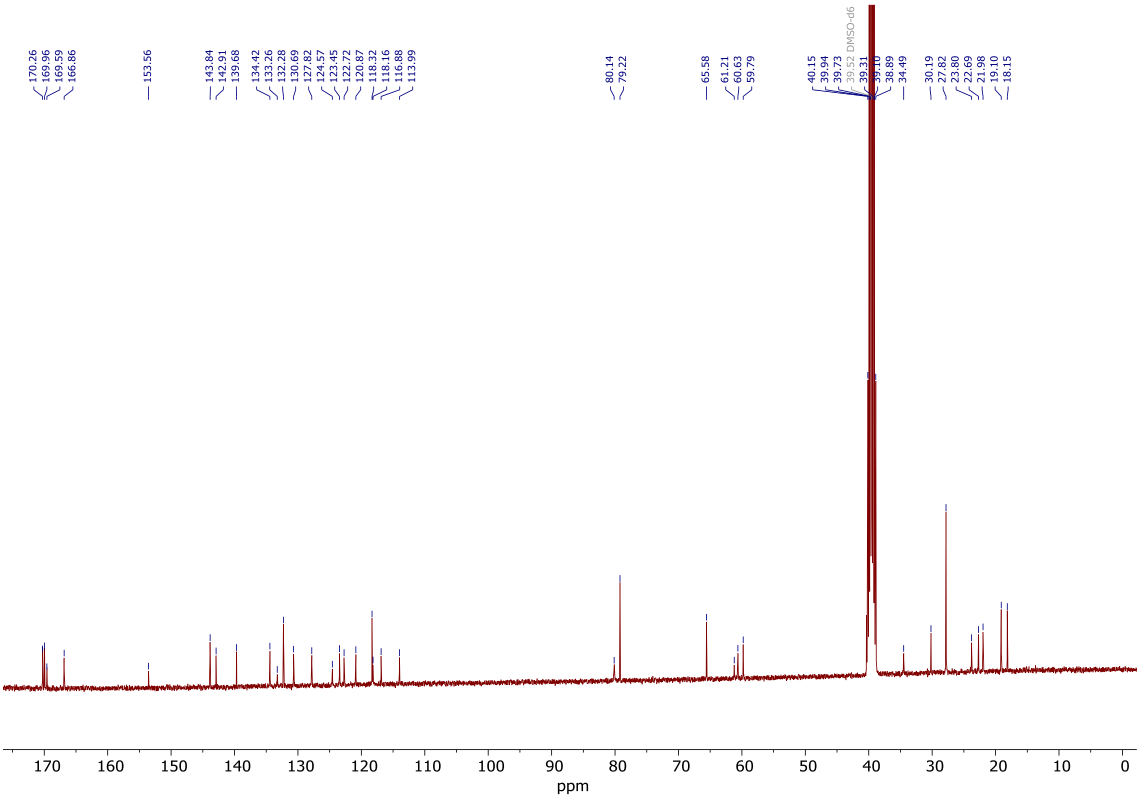

^{1}H NMR (400 MHz, DMSO-d_6, 343 K)

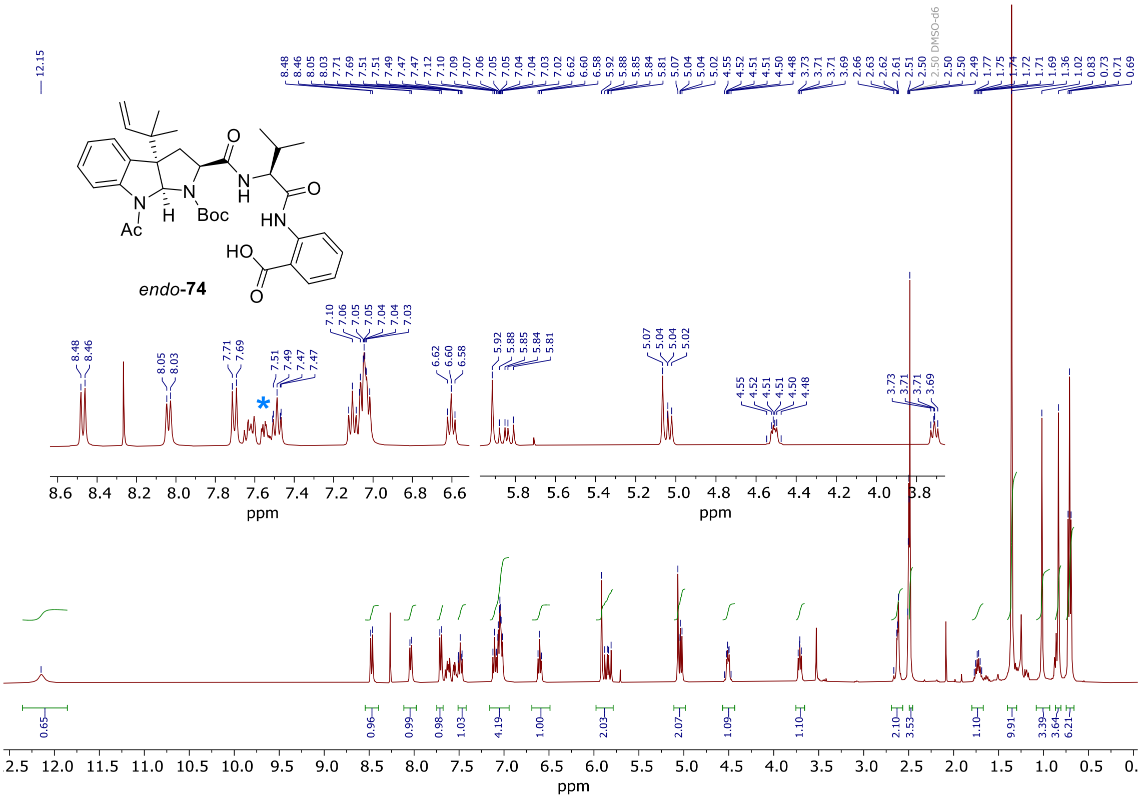

* ^{1}H NMR signals corresponding to remaining PPh_3

^{13}C{^{1}H} NMR (101 MHz, DMSO-d_6, 343 K)

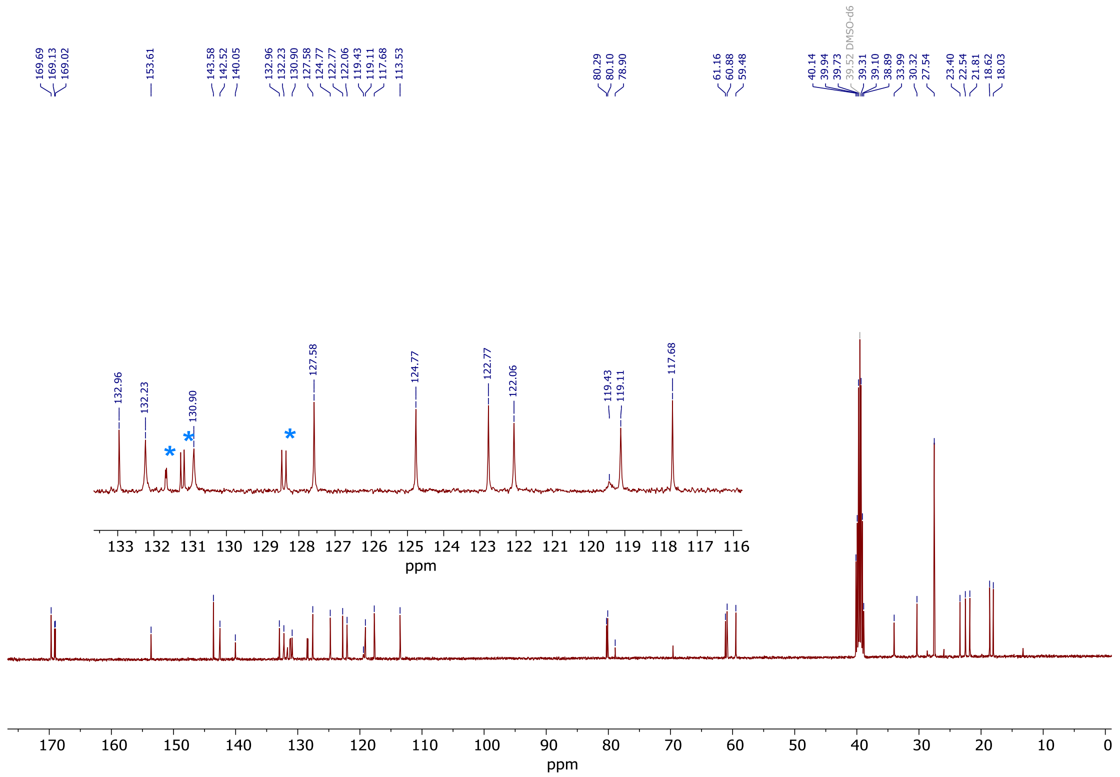

* ^{13}C{^{1}H} NMR signals corresponding to remaining PPh_3

^{1}H NMR (400 MHz, CDCl₃)

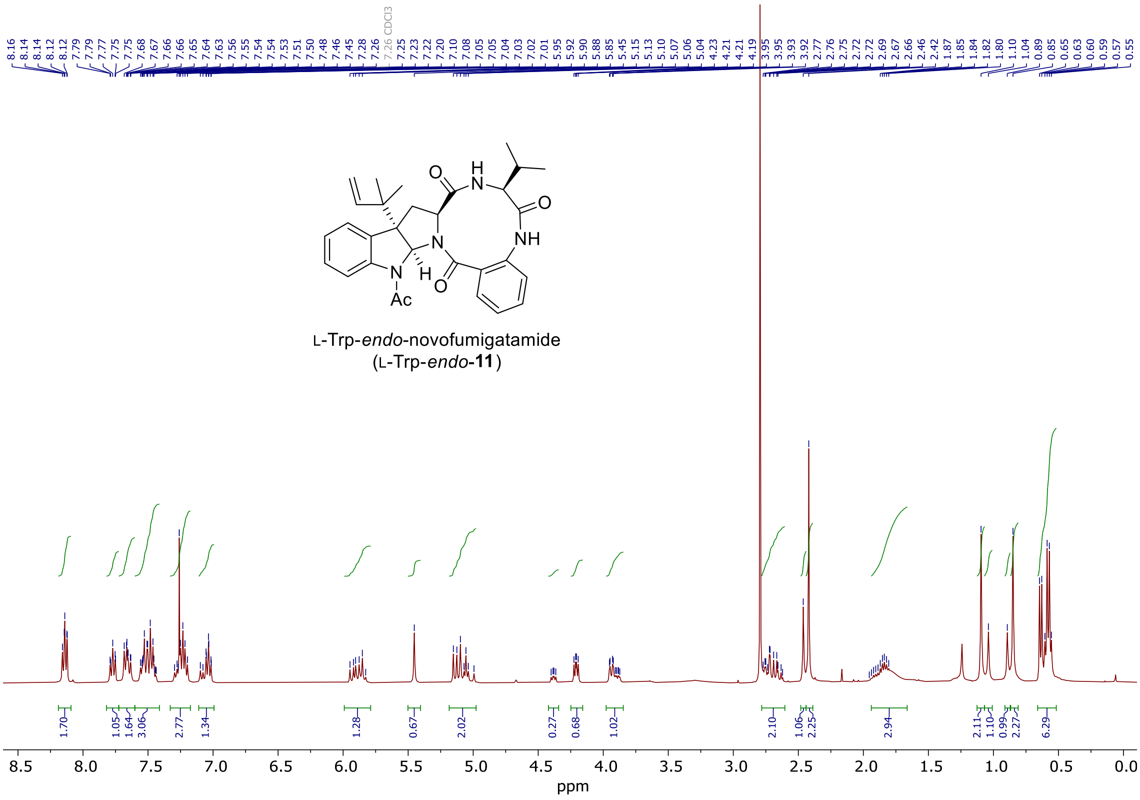

L-Trp-*endo*-novofumigatamide
(L-Trp-*endo*-**11**)

^{13}C{^{1}H} NMR (101 MHz, CDCl₃)

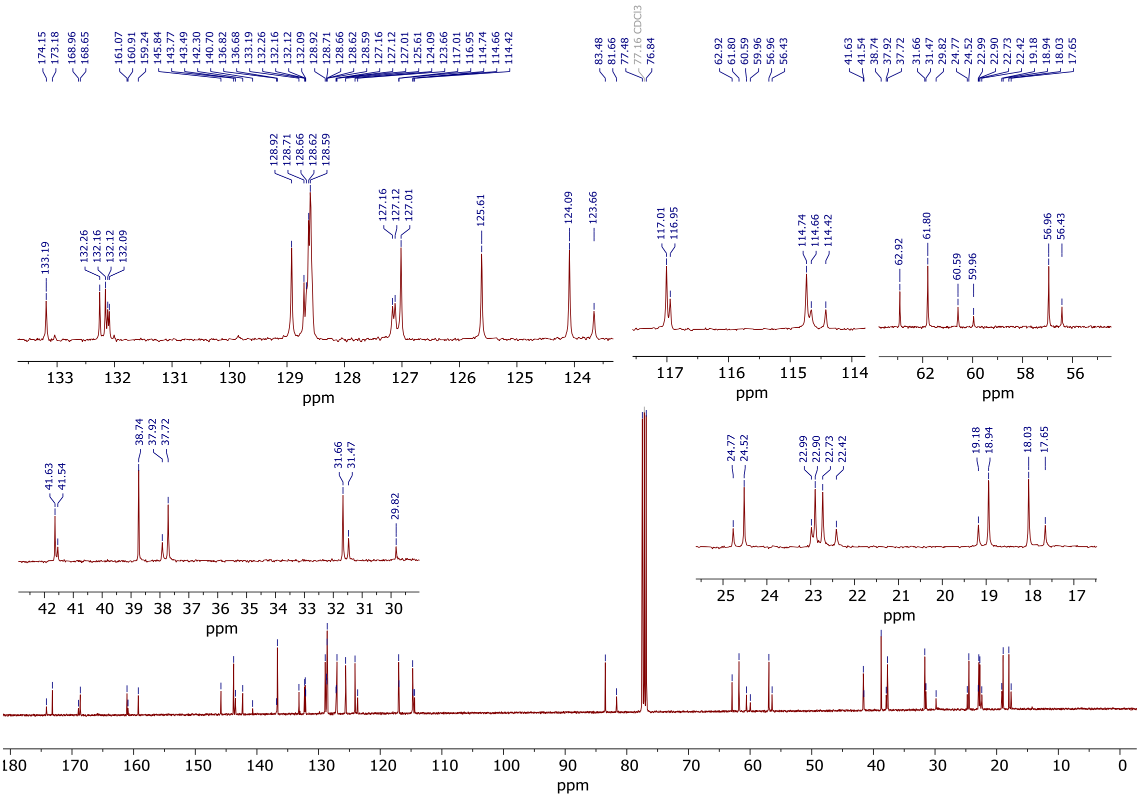

DEPT-135

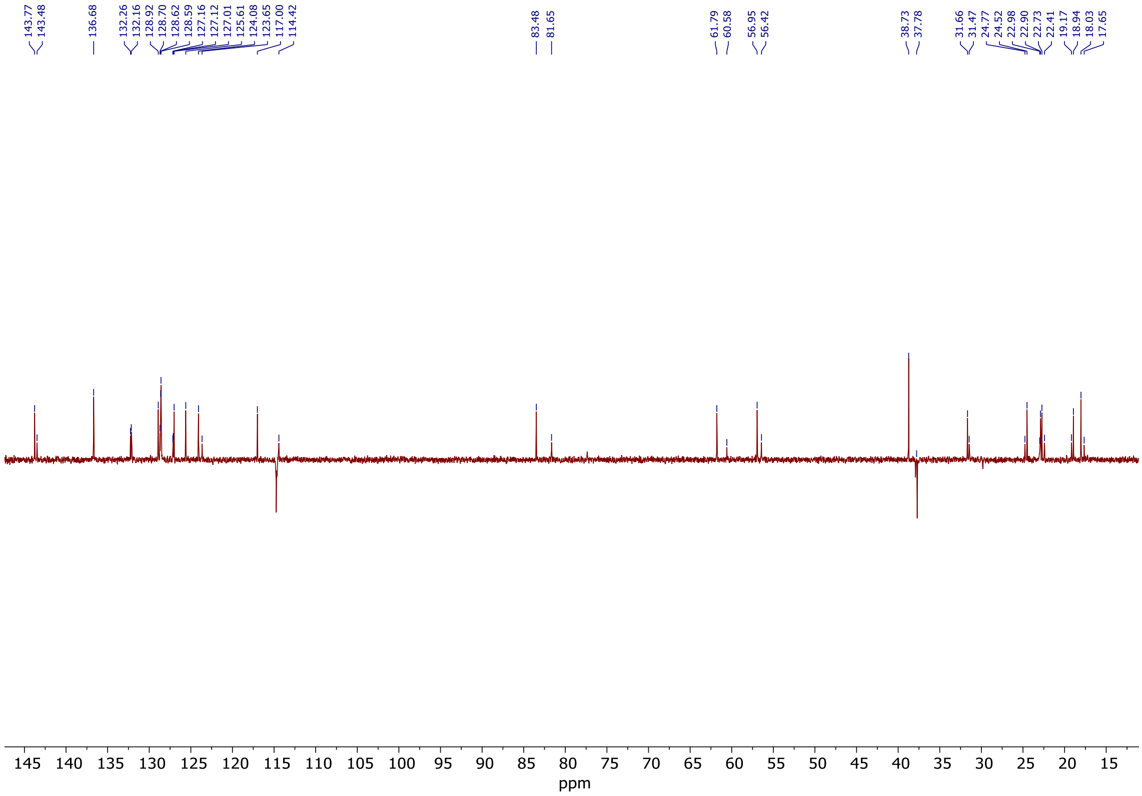

COSY

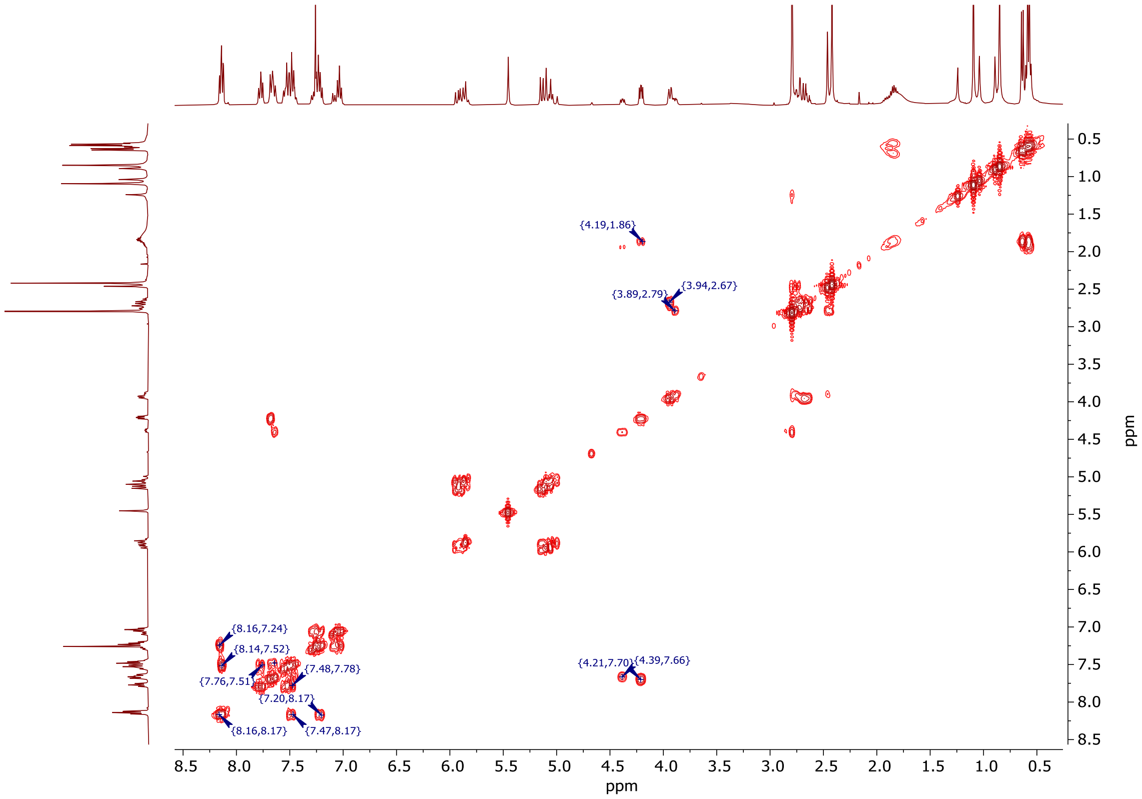

HSQC

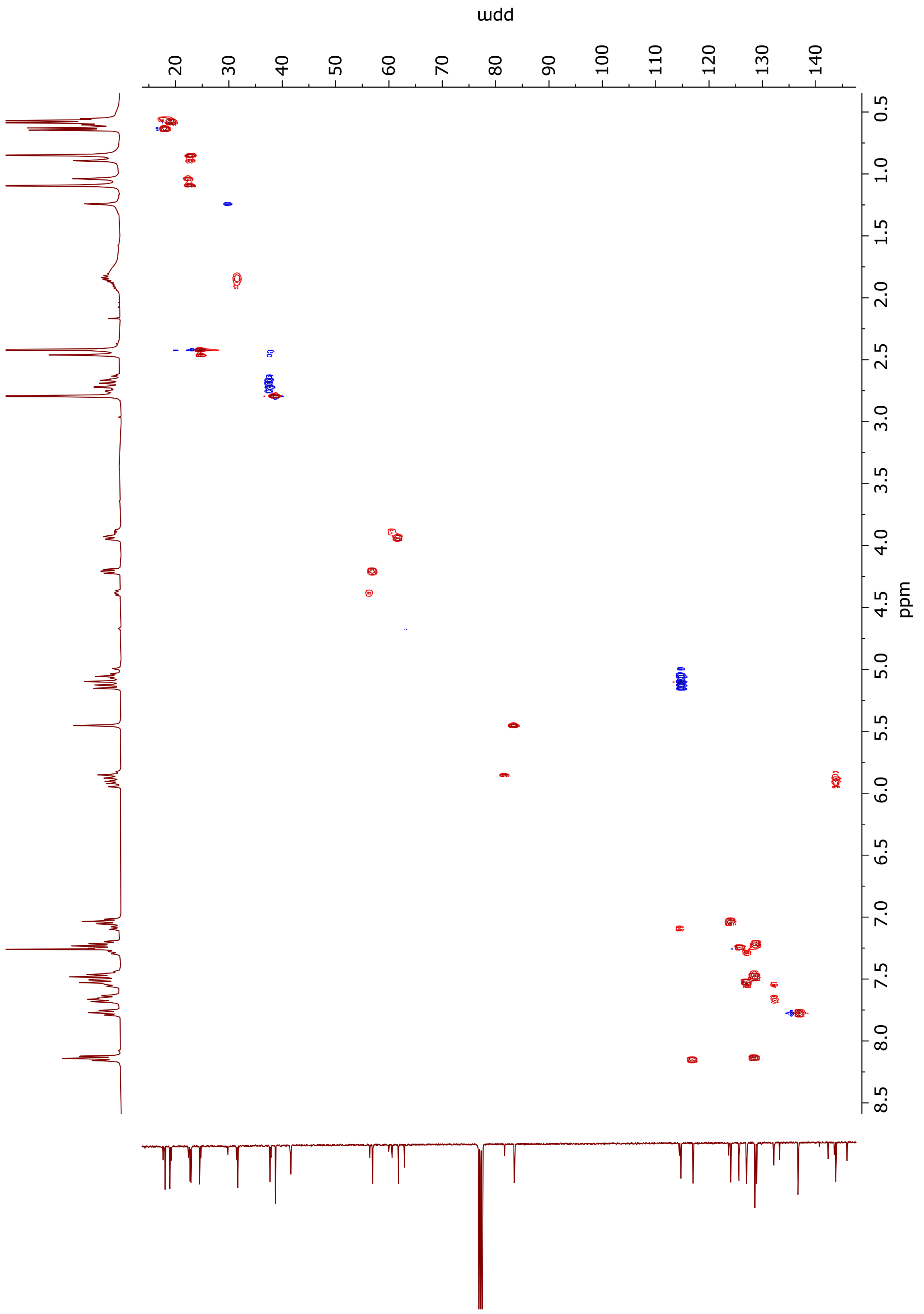

HMBC

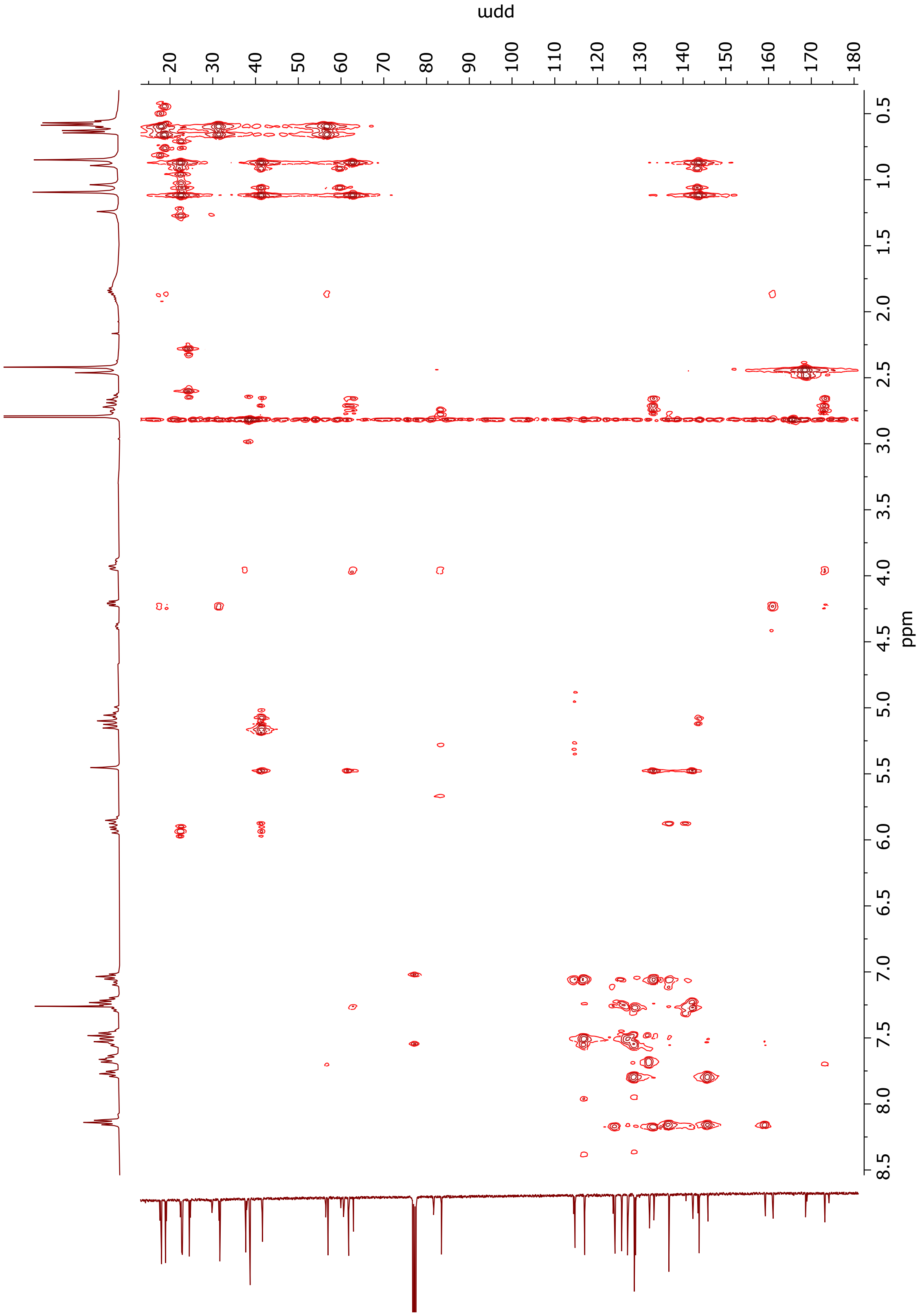
ppm
20 30 40 50 60 70 80 90 100 110 120 130 140 150 160 170 180
ppm
0.5 1.0 1.5 2.0 2.5 3.0 3.5 4.0 4.5 5.0 5.5 6.0 6.5 7.0 7.5 8.0 8.5

5. X-RAY Structures

Exo reverse-prenylated precursor of the synthetic novofumigatamide from L-Trp (*exo*-73)

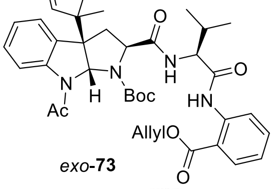

exo-**73**

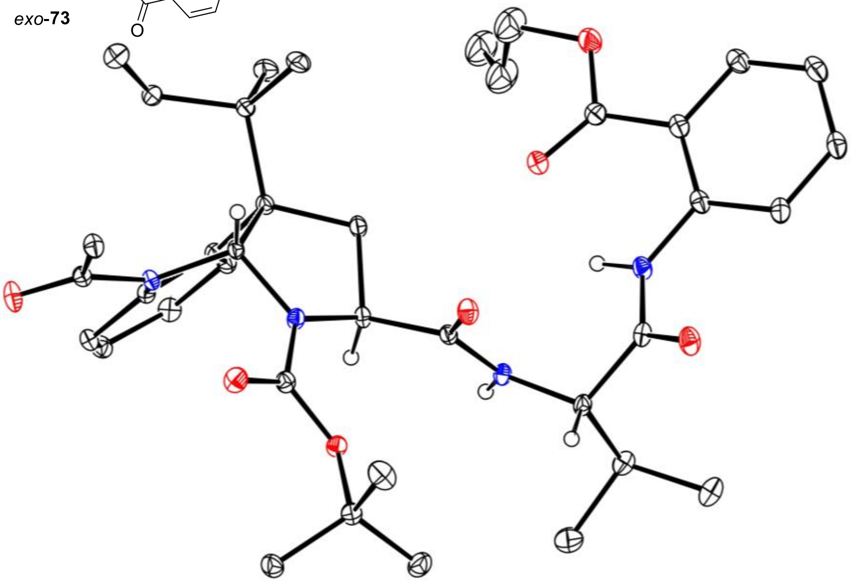

Table 1. Crystal data and structure refinement for *exo*-**73**

Empirical formula	$C_{38}H_{48}N_4O_7$	
Formula weight	672.80	
Temperature	100.0 K	
Wavelength	0.71073 Å	
Crystal system	Orthorhombic	
Space group	$P2_12_12_1$	
Unit cell dimensions	a = 11.8260(4) Å	α= 90°.
	b = 14.1094(5) Å	β= 90°.
	c = 21.7370(7) Å	γ = 90°.
Volume	3627.0(2) $Å^3$	
Z	4	
Density (calculated)	1.232 Mg/m^3	
Absorption coefficient	0.085 mm^{-1}	
F(000)	1440	
Crystal size	0.216 x 0.203 x 0.111 mm^3	
Theta range for data collection	2.365 to 28.304°.	
Index ranges	-15<=h<=15, -18<=k<=18, -25<=l<=28	
Reflections collected	43614	
Independent reflections	8986 [R(int) = 0.0341]	

Completeness to theta = 25.242°	99.8 %
Absorption correction	Semi-empirical from equivalents
Max. and min. transmission	0.7457 and 0.6626
Refinement method	Full-matrix least-squares on F^2
Data / restraints / parameters	8986 / 2 / 452
Goodness-of-fit on F^2	1.048
Final R indices [I>2sigma(I)]	R1 = 0.0400, wR2 = 0.1087
R indices (all data)	R1 = 0.0430, wR2 = 0.1110
Absolute structure parameter	-0.3(2)
Extinction coefficient	n/a
Largest diff. peak and hole	0.751 and -0.622 e.Å^{-3}

Experimental report

Crystallization was achieved in DMSO. A single crystal of *exo*-**73** was analysed by X-ray diffraction and a summary of the crystallographic data and the structure refinement parameters is reported in Table 1. Crystallographic data were collected at 100 K using a Bruker D8 Venture diffractometer with a Photon II CMOS detector and Mo-Kα radiation (λ = 0.71073 Å) generated by an Incoatec high brillance microfocus source equipped with Incoatec Helios multilayer optics. The software $APEX_3$[1] was used for collecting frames of data, indexing reflections, and determination of lattice parameters, SAINT[2] for integration of intensity of reflections, and SADABS[3] for scaling and empirical absorption correction. The structure was solved by dualspace algorithm using the program SHELXT.[4] All non-hydrogen atoms were refined with anisotropic displacement parameters by full-matrix least-squares calculations on F^2 using the program SHELXL[5] with $OLEX_2$.[6] Hydrogen atoms were inserted at calculated positions and constrained with isotropic displacement; except for the hydrogen atoms of the –NH groups, which were located from a Fourier-difference map and refined isotropically restraining the N-H distances. The sample crystallized in the chiral space group $P2_12_12_1$; the absolute configuration was determined by anomalous dispersion effects in diffraction measurements on the crystal using Bayesian statistics on Bijvoet differences[7] [P2(true) = 1.000, P3(true) = 0.999, P3(rac-twin) = 0.7 x 10^{-3} and P_3(false) = 0.4 x 10^{-9}]. Drawings were produced with PLATON.[8]

[1] $APEX_3$ Version 2019.11-0 (Bruker AXS Inc., 2019).
[2] SAINT Version 8.40A (Bruker AXS Inc., 2019).
[3] SADABS Version 2016/2 (Krause, L.; Herbst-Irmer, R.; Sheldrick, G. M.; Stalke, D. *J. Appl. Cryst.* **2015**, *48*, 3).
[4] SHELXT Version 2018/2 (Sheldrick, G. M. *Acta Cryst.* **2015**, *A71*, 3).
[5] SHELXL Version 2018/3 (Sheldrick, G. M. *Acta Cryst.* **2015**, *C71*, 3).
[6] $OLEX_2$: A complete structure solution, refinement and analysis program (Dolomanov, O.V.; Bourhis, L. J.; Gildea, R. J.; Howard, J. A. K.; Puschmann, H. *J. Appl. Cryst.* **2009**, *42*, 339).
[7] Hooft, R. W. W.; Straver, L. H.; Spek, A. L. *J. Appl. Cryst.* **2008**, *41*, 96.
[8] PLATON, *A Multipurpose Crystallographic Tool* (Spek, A. L. *J. Appl. Cryst.*, **2003**, *36*, 7).

6. References

1. Alqahtani, N.; Porwal, S. K.; James, E. D.; Bis, D. M.; Karty, J. A.; Lane, A. L.; Viswanathan, R. Synergism between genome sequencing, tandem mass spectrometry and bio-inspired synthesis reveals insights into nocardioazine B biogenesis. *Org. Biomol. Chem.* **2015**, *13*, 7177-7192.
2. Hewitt, P. R.; Cleator, E.; Ley, S. V. A concise total synthesis of (+)-okaramine C. *Org. Biomol. Chem.* **2004**, *2*, 2415-2417.
3. Xiao, J.; Wong, Z. Z.; Lu, Y. P.; Loh, T. P. Hexahydropyrrolo[2,3-*b*]indoles: A New Class of Structurally Rigid Tricyclic Skeleton for Oxazaborolidine-Catalyzed Asymmetric Borane Reduction. *Adv. Synth. Catal.* **2010**, *352*, 1107-1112.
4. Coste, A.; Toumi, M.; Wright, K.; Razafimahaleo, V.; Couty, F.; Marrot, J.; Evano, G. Copper-catalyzed cyclization of iodo-tryptophans: a straightforward synthesis of pyrroloindoles. *Org. Lett.* **2008**, *10*, 3841-3844.
5. Ishikawa, K.; Hosoe, T.; Itabashi, T.; Kayoko, T.; Takashi, Y.; Ken-ichi, K. A novofumigatamide, new cyclic tripeptide from *Aspergillus novofumigatus*. *Heterocycles* **2010**, *81*, 2143-2148.
6. Iwasa, E.; Hamashima, Y.; Fujishiro, S.; Hashizume, D.; Sodeoka, M. Total syntheses of chaetocin and *ent*-chaetocin. *Tetrahedron* **2011**, *67*, 6587-6599.
7. Boyer, N.; Movassaghi, M. Concise total synthesis of (+)-gliocladins B and C. *Chem. Sci.* **2012**, *3*, 1798-1803.
8. Sato, S.; Hirayama, A.; Ueda, H.; Tokuyama, H. Total Syntheses of (+)-T988 B and (+)-T988 C through the $AgNTf_2$-Mediated Coupling of Bromopyrroloindoline with Indole. *Asian J. Org. Chem.* **2017**, *6*, 54-58.
9. Engler, T. A.; Reddy, J. P.; Combrink, K. D.; Vander Velde, D. Formal 2 + 2 and 3 + 2 cycloaddition reactions of 2*H*-chromenes with 2-alkoxy-1,4-benzoquinones: regioselective synthesis of substituted pterocarpans. *J. Org. Chem.* **1990**, *55*, 1248-1254.
10. Naruta, Y.; Nishigaichi, Y.; Maruyama, K. Tributyl(3-methyl-2-butenyl)tin [Stannane, tributyl(3-methyl-2-butenyl)-]. *Org. Synth.* **1993**, *71*, 118.
11. Gualandi, A.; Canestrari, P.; Emer, E.; Cozzi, P. G. A Straightforward Organocatalytic Alkylation of 2-Arylacetaldehydes: An Approach towards Bisabolanes. *Adv. Synth. Catal.* **2014**, *356*, 528-536.
12. Robertson, J.; Hall, M. J.; Stafford, P. M.; Green, S. P. Ene cyclisations of α-(prenyl)dialkylsilyloxy aldehydes: formation and oxidative cleavage of oxasilacyclohexanols. *Org. Biomol. Chem.* **2003**, *1*, 3758-3767.
13. Soderquist, J. A.; Rivera, I.; Negron, A. 1- and 2-(trialkylsilyl)ethanols: new silyl reagents from tin, lithium, and boron chemistry. *J. Org. Chem.* **1989**, *54*, 4051-4055.
14. Williams, R. M.; Sinclair, P. J.; Zhai, D.; Chen, D. Practical asymmetric syntheses of .alpha.-amino acids through carbon-carbon bond constructions on electrophilic glycine templates. *J. Am. Chem. Soc.* **1988**, *110*, 1547-1557.
15. Downing, S. V.; Aguilar, E.; Meyers, A. I. Total Synthesis of Bistratamide D. *J. Org. Chem.* **1999**, *64*, 826-831.
16. Behloul, C.; Guijarro, D.; Yus, M. Deallyloxy- and debenzyloxycarbonylation of protected alcohols, amines and thiols via a naphthalene-catalysed lithiation reaction. *Tetrahedron* **2005**, *61*, 9319-9324.
17. ElHady, A. K.; Shih, S.-P.; Chen, Y.-C.; Liu, Y.-C.; Ahmed, N. S.; Keeton, A. B.; Piazza, G. A.; Engel, M.; Abadi, A. H.; Abdel-Halim, M. Extending the use of tadalafil scaffold: Development of novel selective phosphodiesterase 5 inhibitors and histone deacetylase inhibitors. *Bioorg. Chem.* **2020**, *98*, 103742.
18. Qing, G.-y.; He, Y.-b.; Wang, F.; Qin, H.-j.; Hu, C.-g.; Yang, X. Enantioselective Fluorescent Sensors for Chiral Carboxylates Based on Calix[4]arenes Bearing an L-Tryptophan Unit. *Eur. J. Org. Chem.* **2007**, *2007*, 1768-1778.
19. Wada, M.; Suzuki, H.; Kato, M.; Oikawa, H.; Tsubouchi, A.; Oguri, H. Stereodivergent Synthesis of Bispyrrolidinoindoline Alkaloidal Scaffolds and Generation of a Lead Candidate with Stereospecific Antiproliferative Activity. *ChemBioChem* **2019**, *20*, 1273-1281.

20. Loach, R. P.; Fenton, O. S.; Movassaghi, M. Concise Total Synthesis of (+)-Asperazine, (+)-Pestalazine A, and (+)-*iso*-Pestalazine A. Structure Revision of (+)-Pestalazine A. *J. Am. Chem. Soc.* **2016**, *138*, 1057-1064.
21. Hakamata, H.; Ueda, H.; Tokuyama, H. Construction of Indole Structure on Pyrroloindolines via $AgNTf_2$-Mediated Amination/Cyclization Cascade: Application to Total Synthesis of (+)-Pestalazine B. *Org. Lett.* **2019**, *21*, 4205-4209.
22. Wu, J.; Abou-Hamdan, H.; Guillot, R.; Kouklovsky, C.; Vincent, G. Electrochemical synthesis of 3a-bromofuranoindolines and 3a-bromopyrroloindolines mediated by $MgBr_2$. *Chem. Commun.* **2020**, *56*, 1713-1716.
23. Pérez-Balado, C.; de Lera, Á. R. Expedient Total Syntheses of WIN 64745 and WIN 64821. *Org. Lett.* **2008**, *10*, 3701-3704.
24. Wang, M.-Z.; Si, T.-X.; Ku, C.-F.; Zhang, H.-J.; Li, Z.-M.; Chan, A. S. C. Synthesis of Javanicunines A and B, 9-Deoxy-PF1233s A and B, and Absolute Configuration Establishment of Javanicunine B. *J. Org. Chem.* **2019**, *84*, 831-839.
25. Hart, D. J.; Magomedov, N. A. Synthesis of *ent*-Alantrypinone. *J. Am. Chem. Soc.* **2001**, *123*, 5892-5899.
26. Wang, H.; Ganesan, A. Total Synthesis of the Quinazoline Alkaloids (−)-Fumiquinazoline G and (−)-Fiscalin B. *J. Org. Chem.* **1998**, *63*, 2432-2433.
27. Reay, A. J.; Williams, T. J.; Fairlamb, I. J. S. Unified mild reaction conditions for C2-selective Pd-catalysed tryptophan arylation, including tryptophan-containing peptides. *Org. Biomol. Chem.* **2015**, *13*, 8298-8309.
